# Supplementary figures and images for: An image processing technique for optimizing industrial defect detection using dehazing algorithms (part 2 of 2)
Source: PLoS One. 2025 May 2;20(5):e0322217. doi: 10.1371/journal.pone.0322217 (PMC12047806; doi:10.1371/journal.pone.0322217)

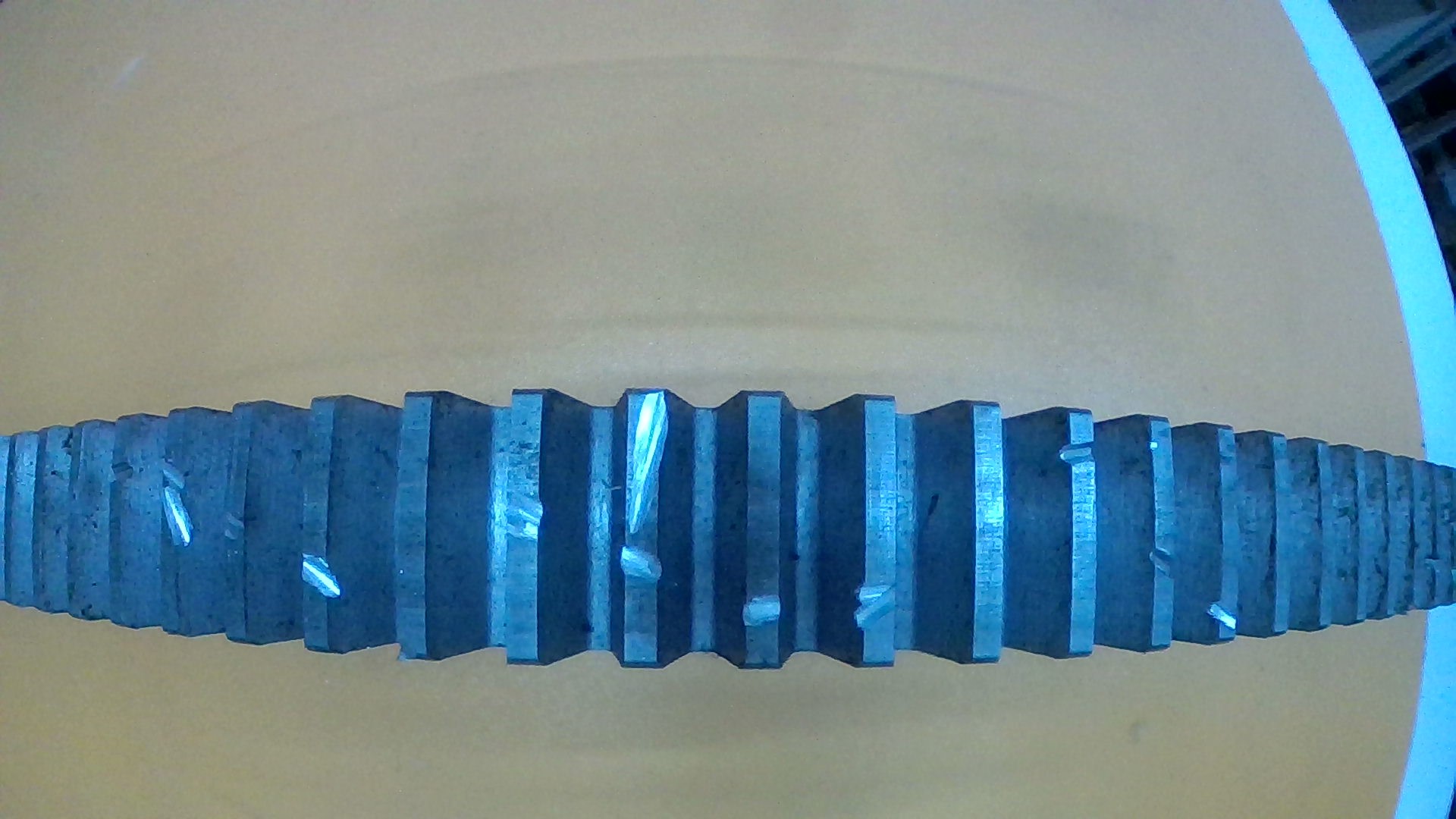

Supplement: S1 Data — (ZIP) [file pone.0322217.s001.zip › dataset/3/WIN_20250111_19_16_42_Pro.jpg]

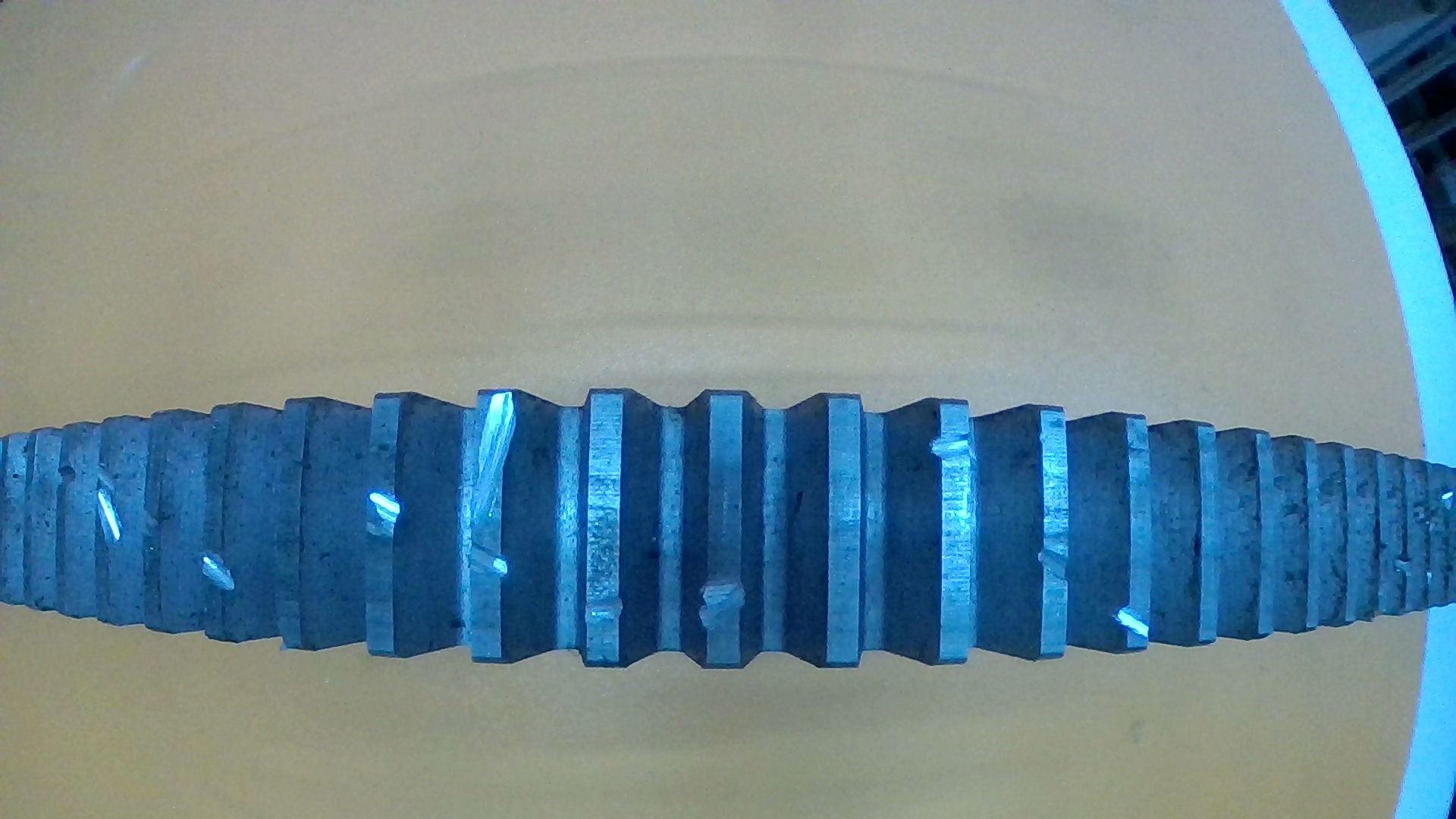

Supplement: S1 Data — (ZIP) [file pone.0322217.s001.zip › dataset/3/WIN_20250111_19_16_45_Pro.jpg]

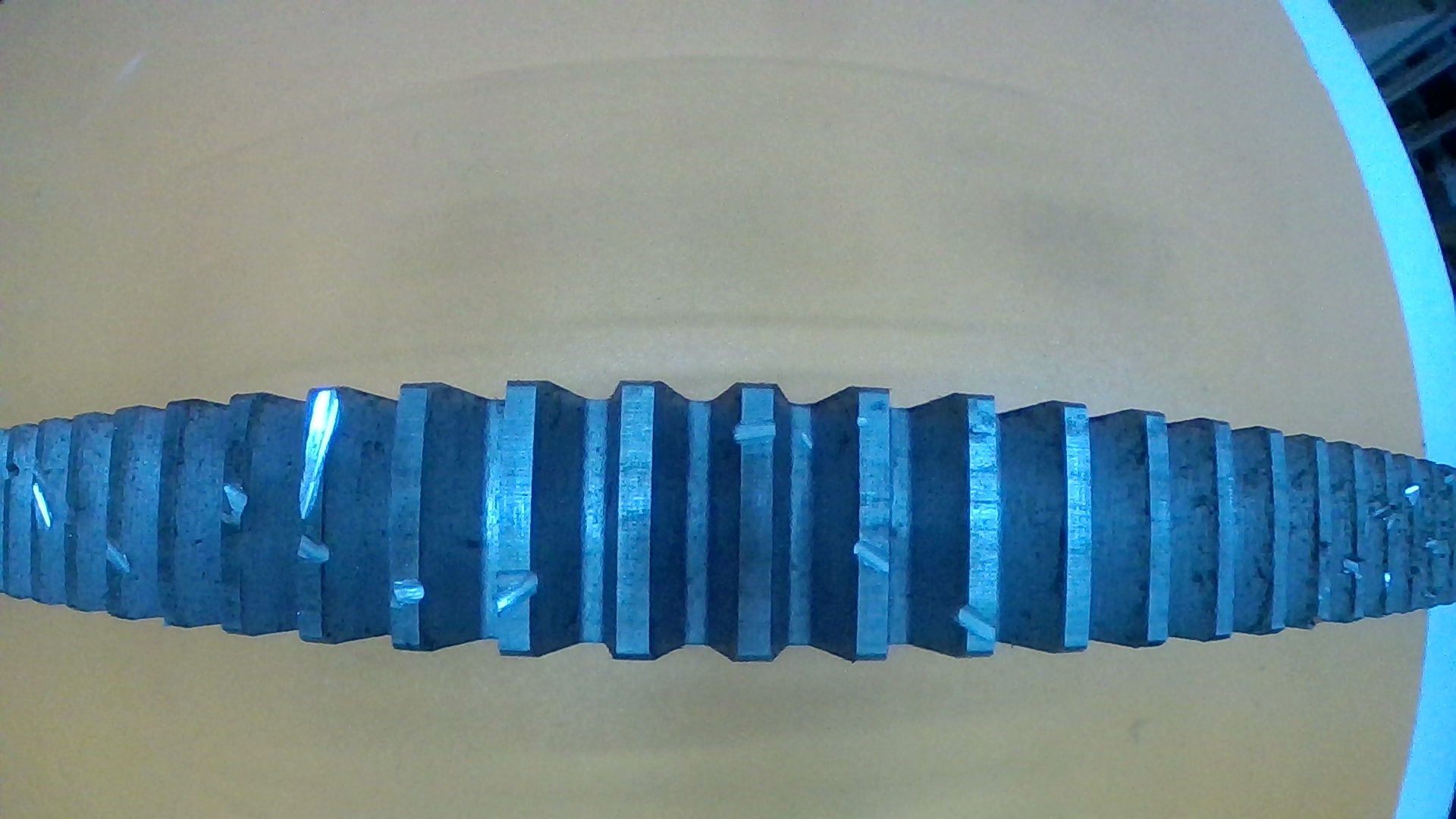

Supplement: S1 Data — (ZIP) [file pone.0322217.s001.zip › dataset/3/WIN_20250111_19_16_48_Pro.jpg]

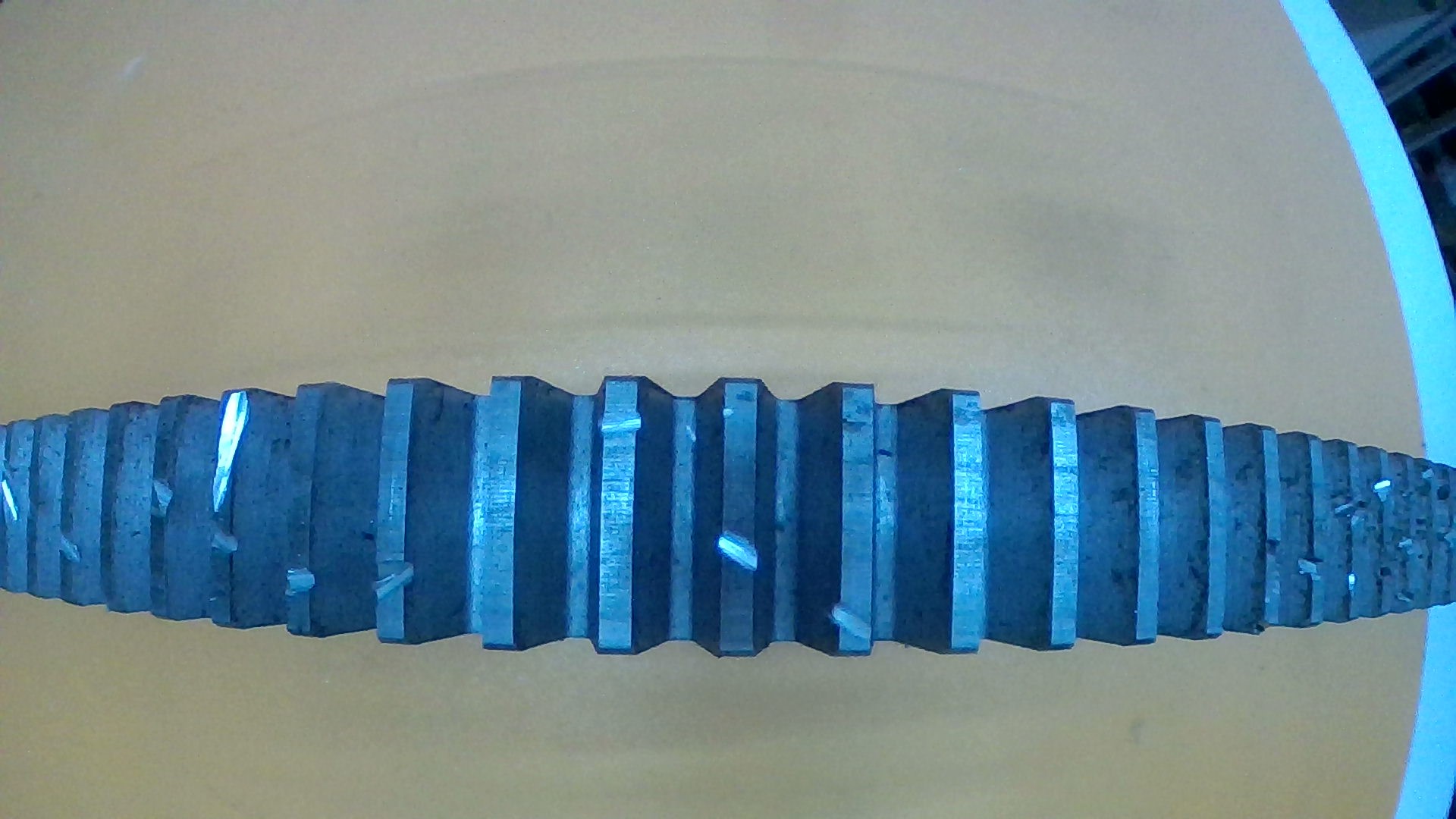

Supplement: S1 Data — (ZIP) [file pone.0322217.s001.zip › dataset/3/WIN_20250111_19_16_50_Pro.jpg]

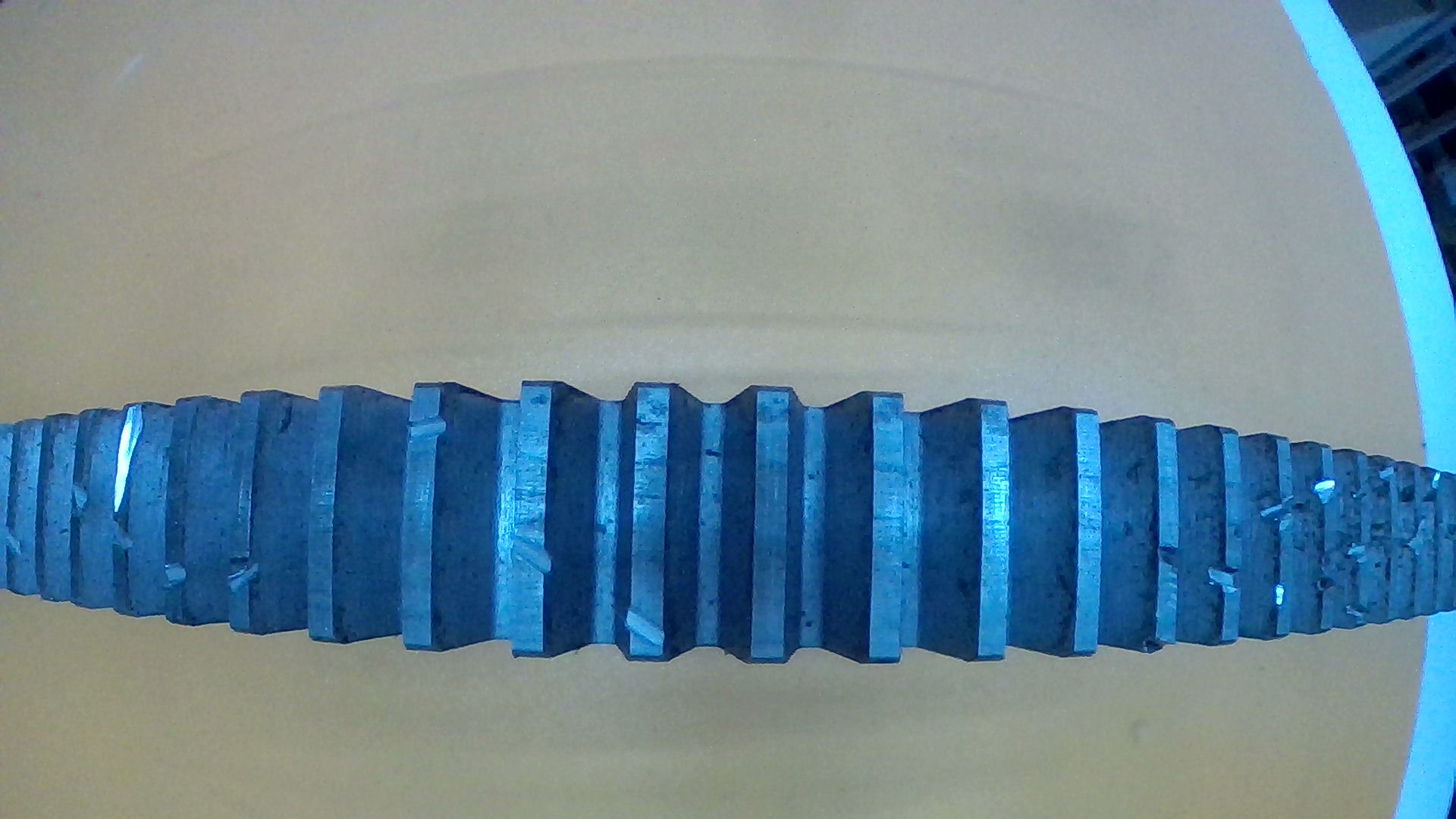

Supplement: S1 Data — (ZIP) [file pone.0322217.s001.zip › dataset/3/WIN_20250111_19_16_53_Pro.jpg]

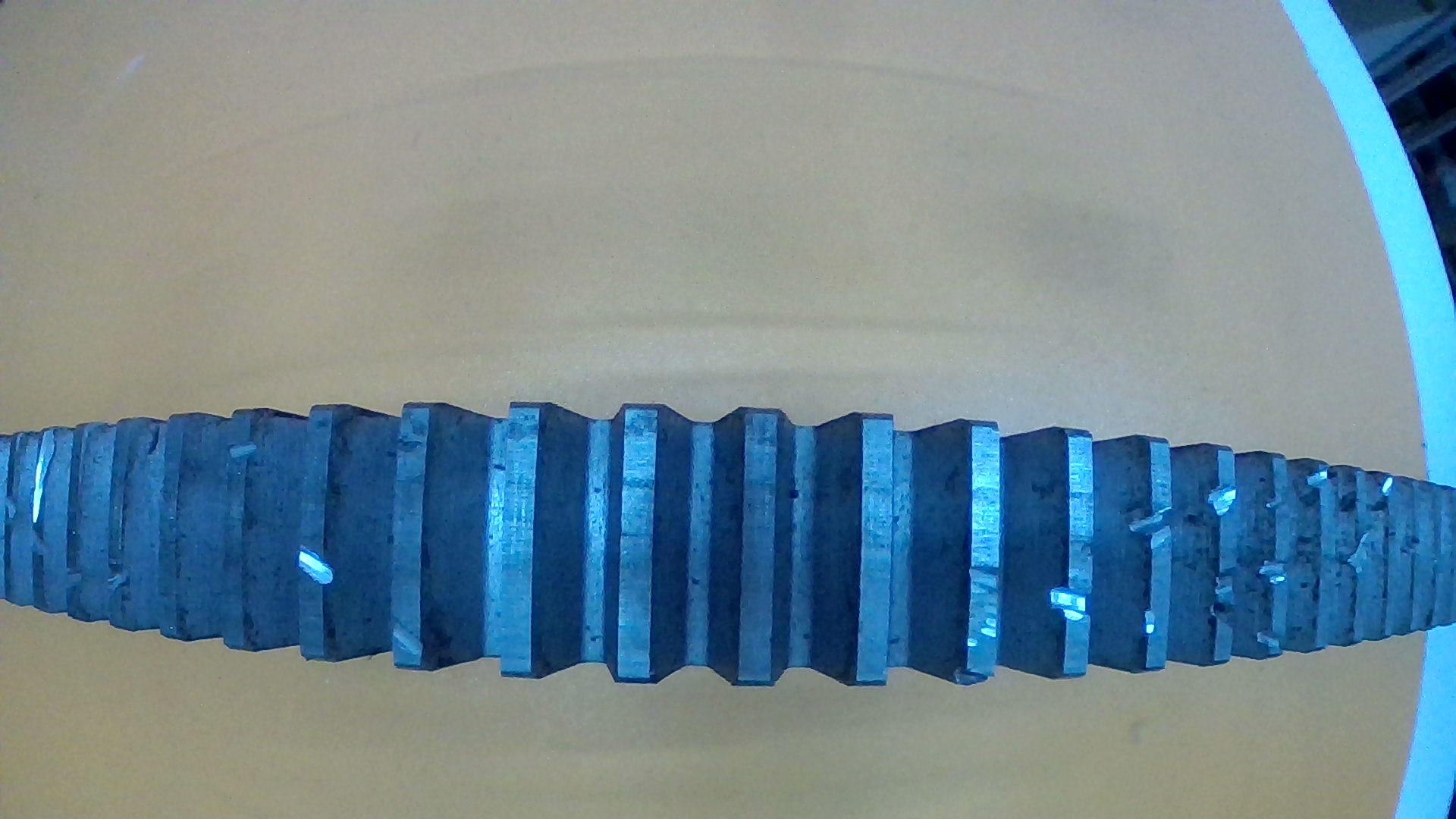

Supplement: S1 Data — (ZIP) [file pone.0322217.s001.zip › dataset/3/WIN_20250111_19_16_56_Pro.jpg]

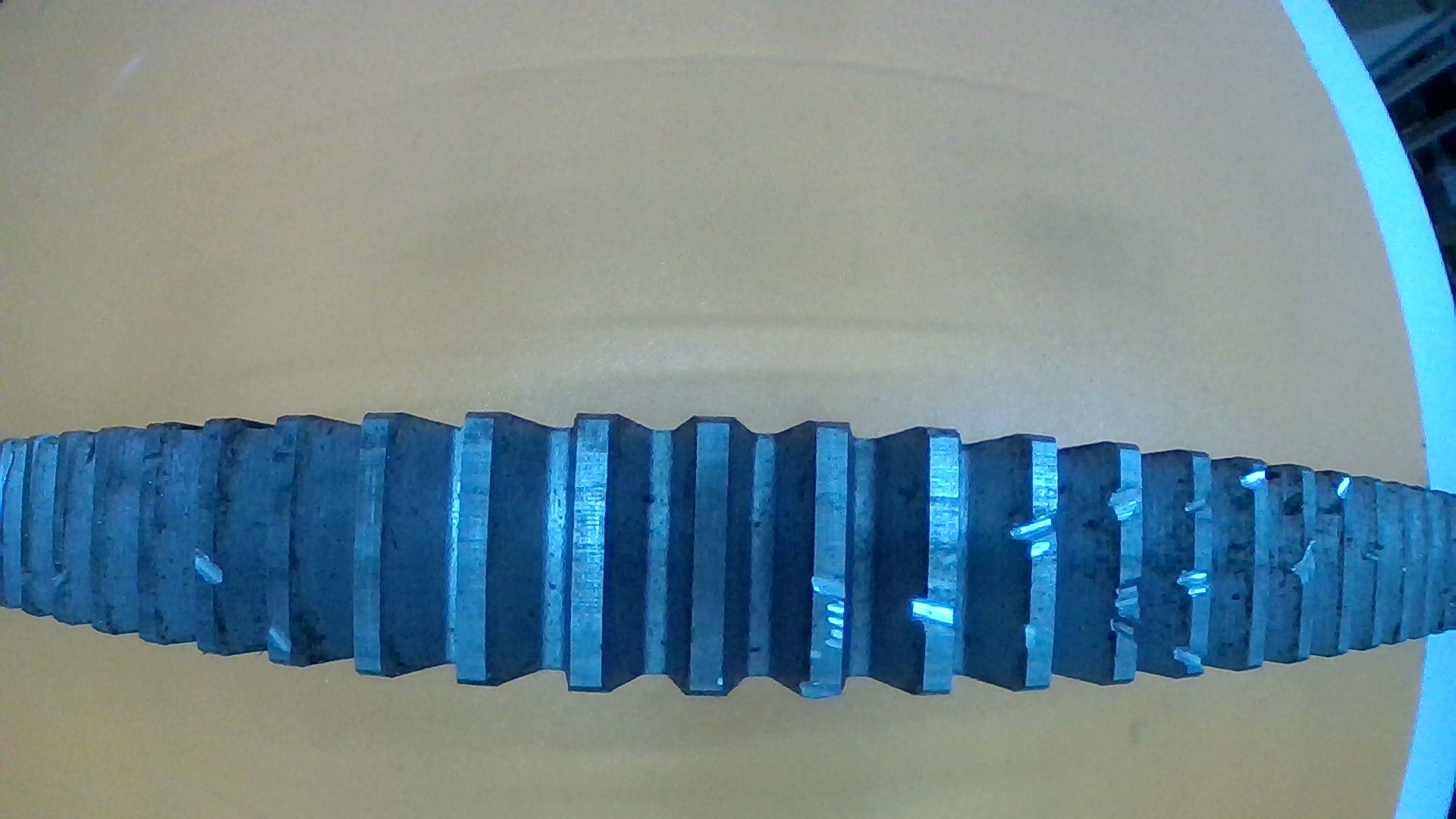

Supplement: S1 Data — (ZIP) [file pone.0322217.s001.zip › dataset/3/WIN_20250111_19_16_59_Pro.jpg]

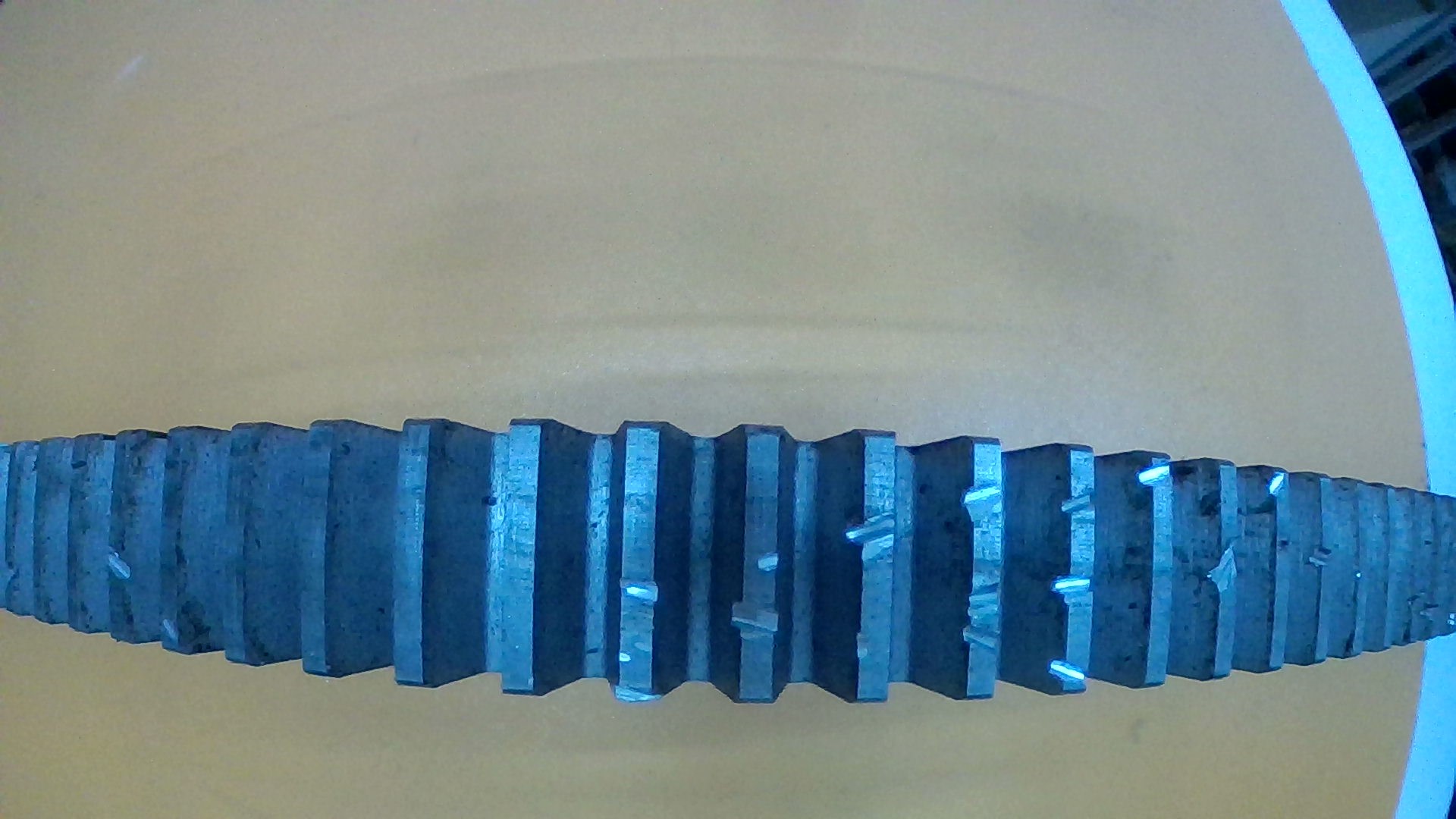

Supplement: S1 Data — (ZIP) [file pone.0322217.s001.zip › dataset/3/WIN_20250111_19_17_01_Pro.jpg]

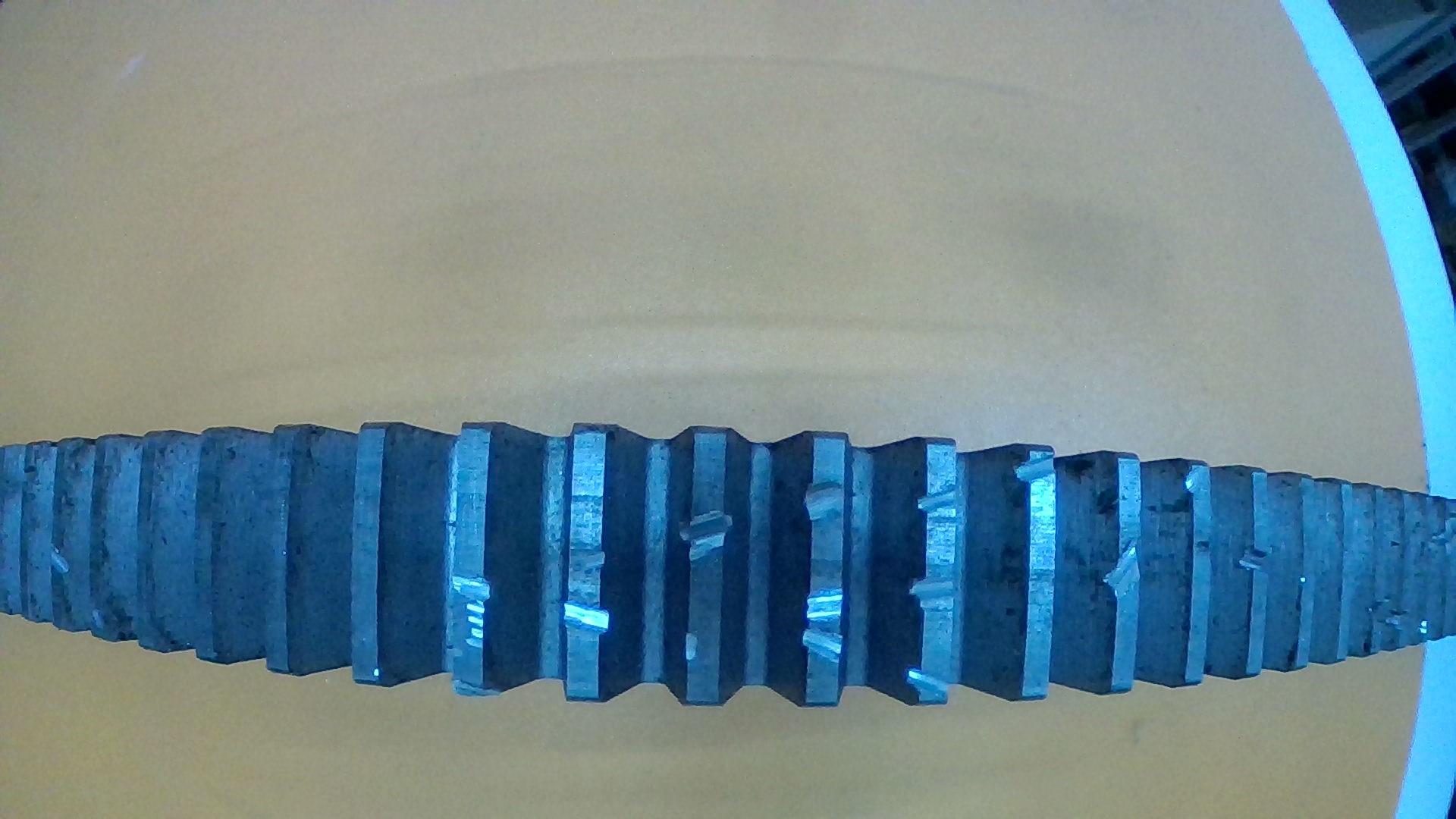

Supplement: S1 Data — (ZIP) [file pone.0322217.s001.zip › dataset/3/WIN_20250111_19_17_03_Pro.jpg]

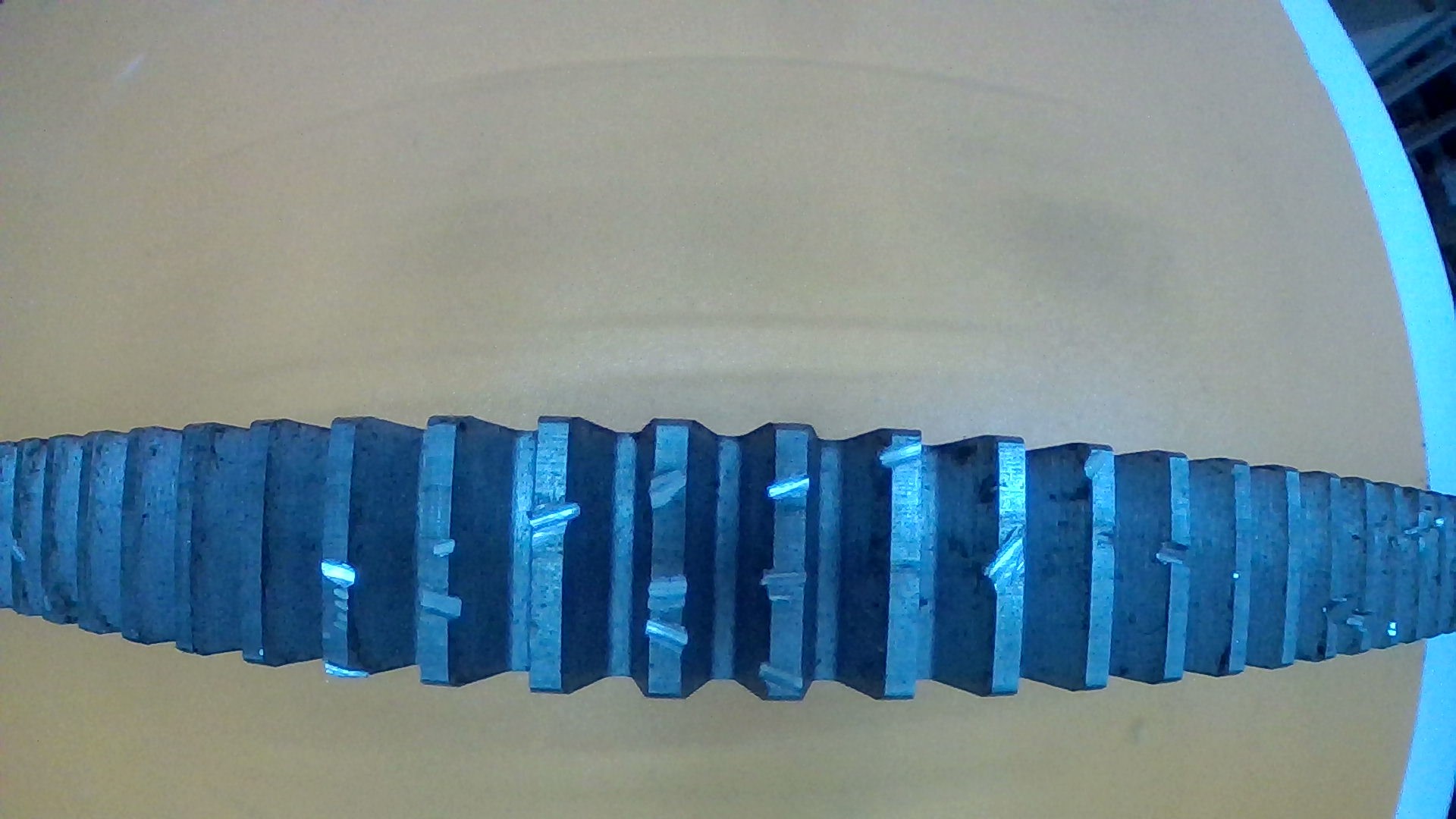

Supplement: S1 Data — (ZIP) [file pone.0322217.s001.zip › dataset/3/WIN_20250111_19_17_05_Pro.jpg]

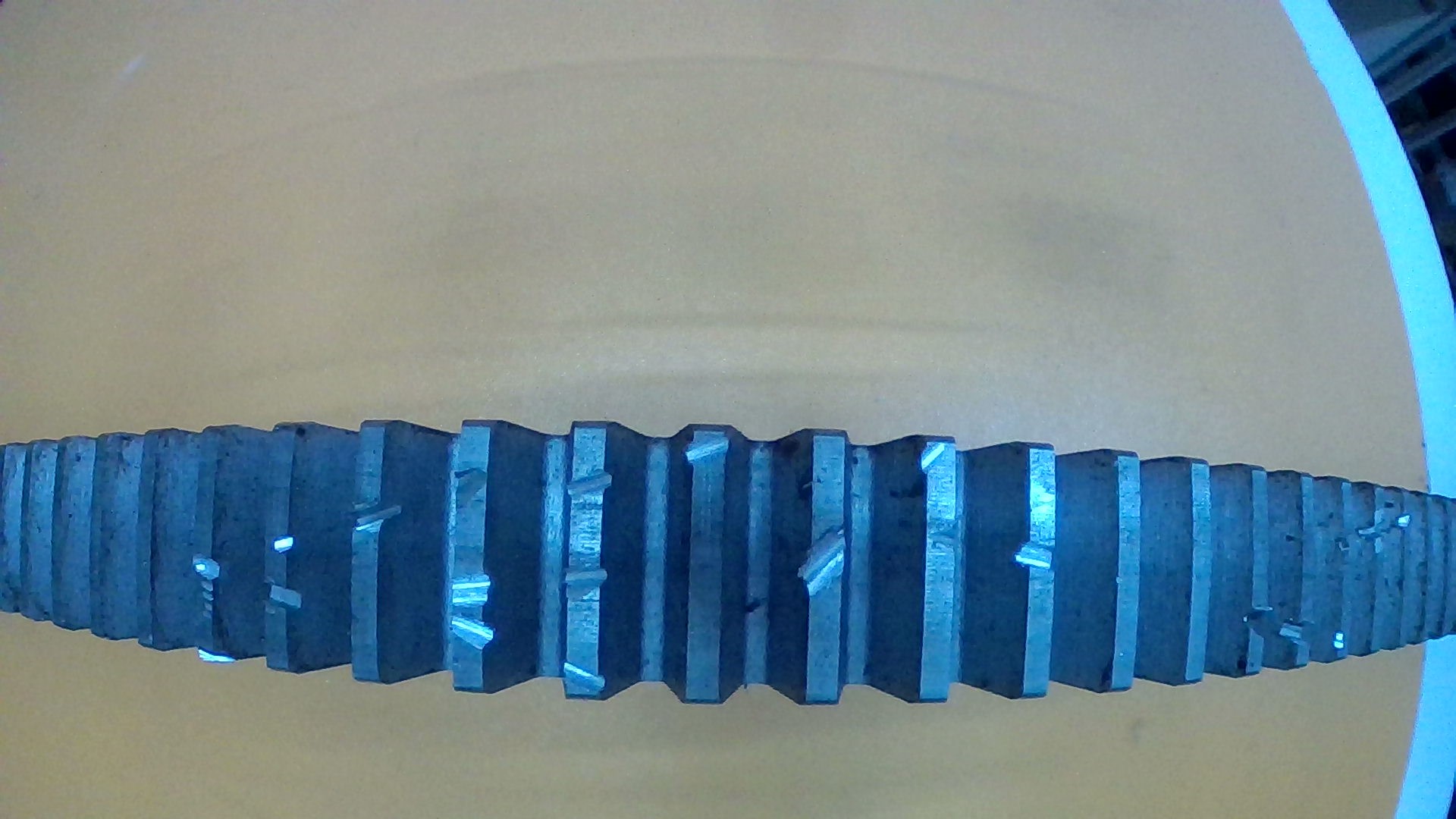

Supplement: S1 Data — (ZIP) [file pone.0322217.s001.zip › dataset/3/WIN_20250111_19_17_07_Pro.jpg]

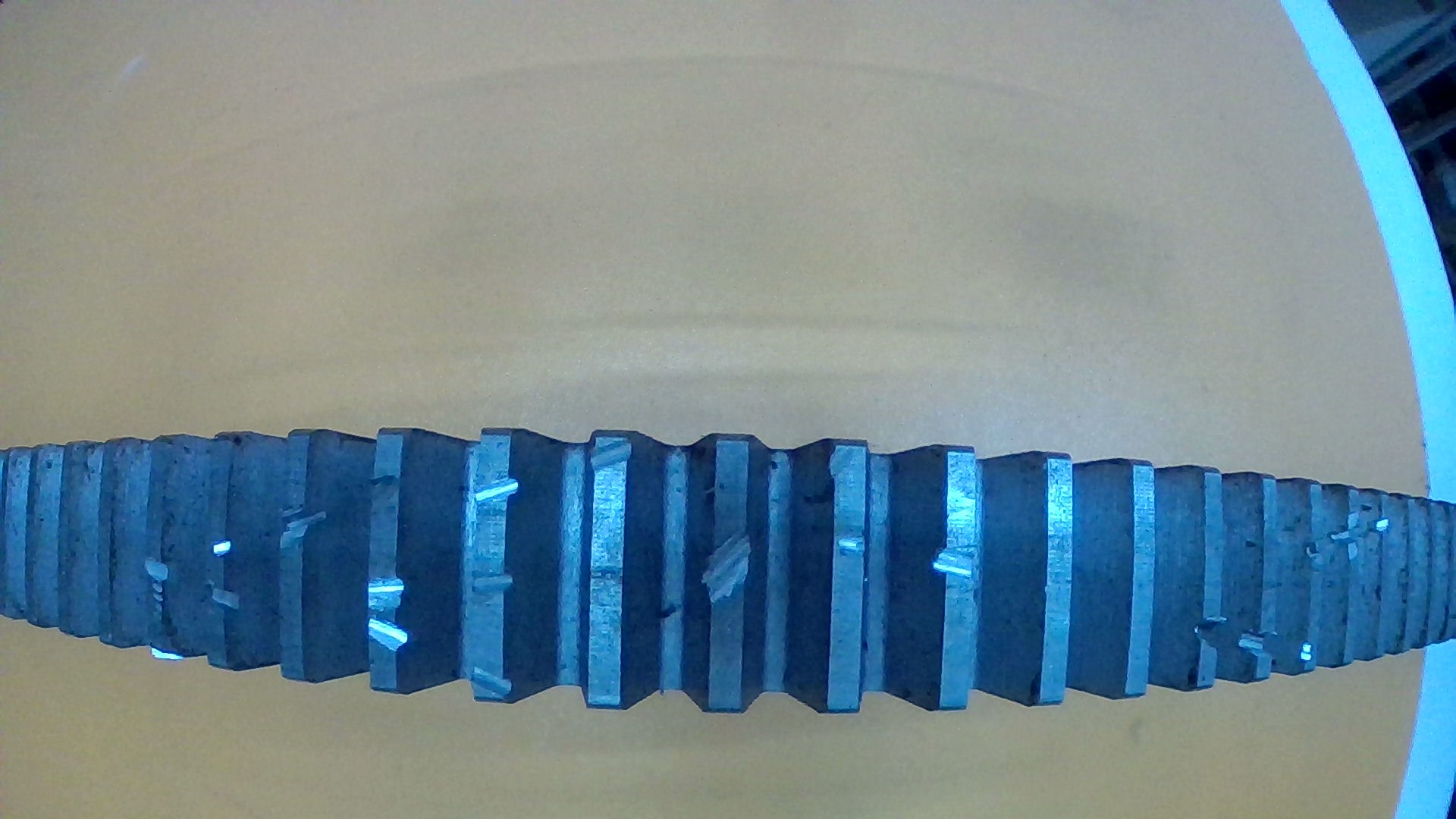

Supplement: S1 Data — (ZIP) [file pone.0322217.s001.zip › dataset/3/WIN_20250111_19_17_11_Pro.jpg]

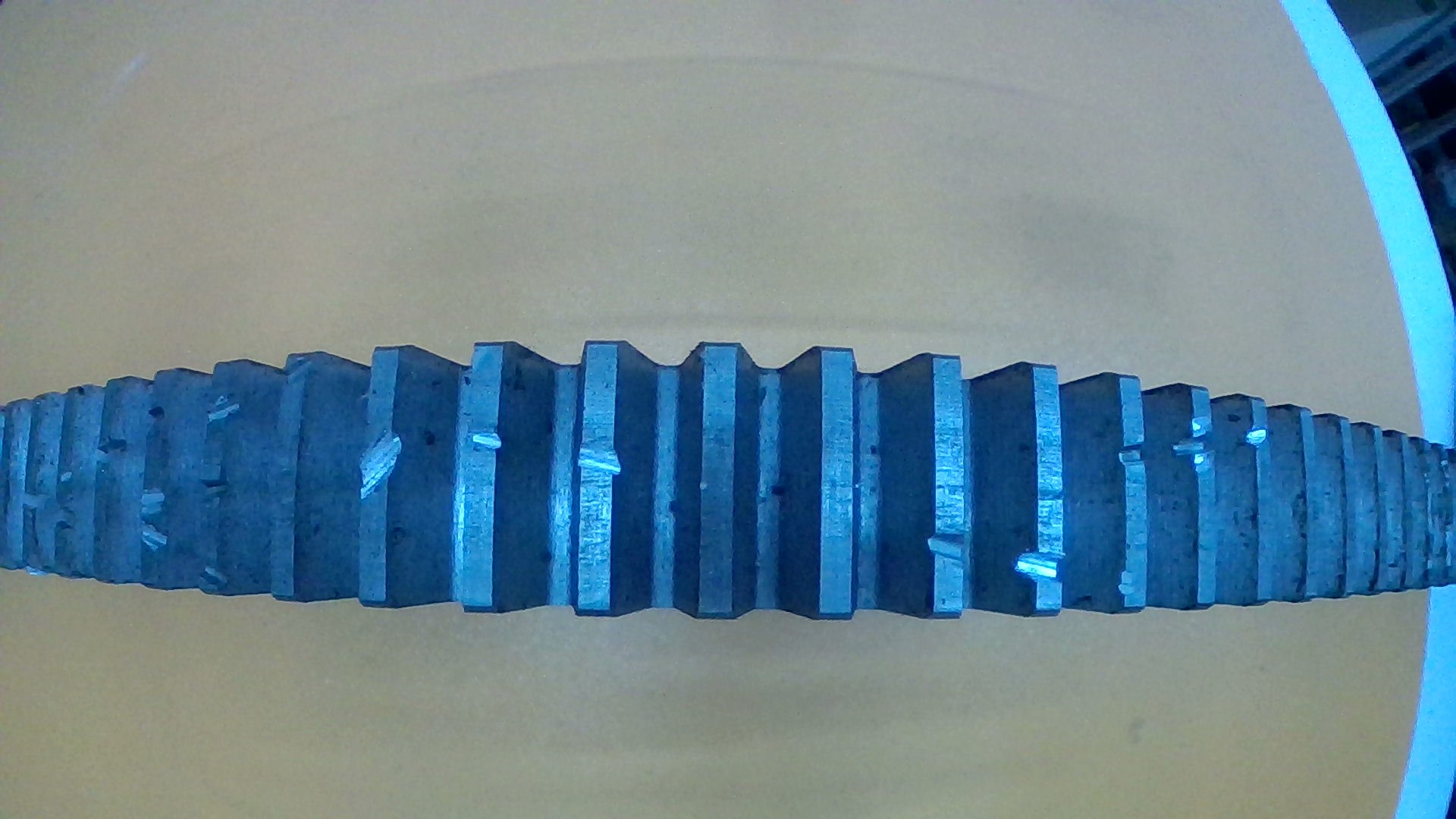

Supplement: S1 Data — (ZIP) [file pone.0322217.s001.zip › dataset/3/WIN_20250111_19_17_32_Pro.jpg]

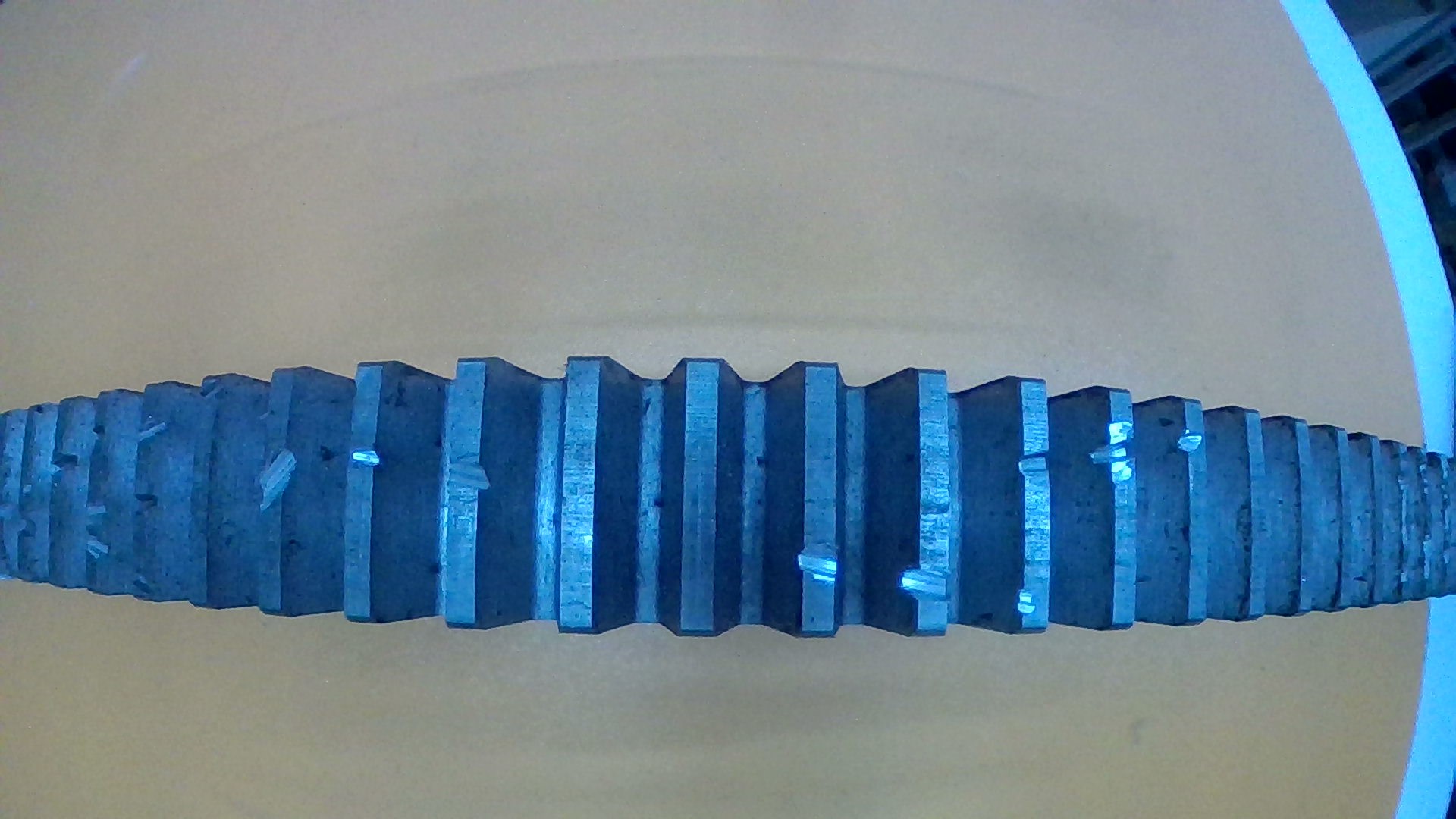

Supplement: S1 Data — (ZIP) [file pone.0322217.s001.zip › dataset/3/WIN_20250111_19_17_34_Pro.jpg]

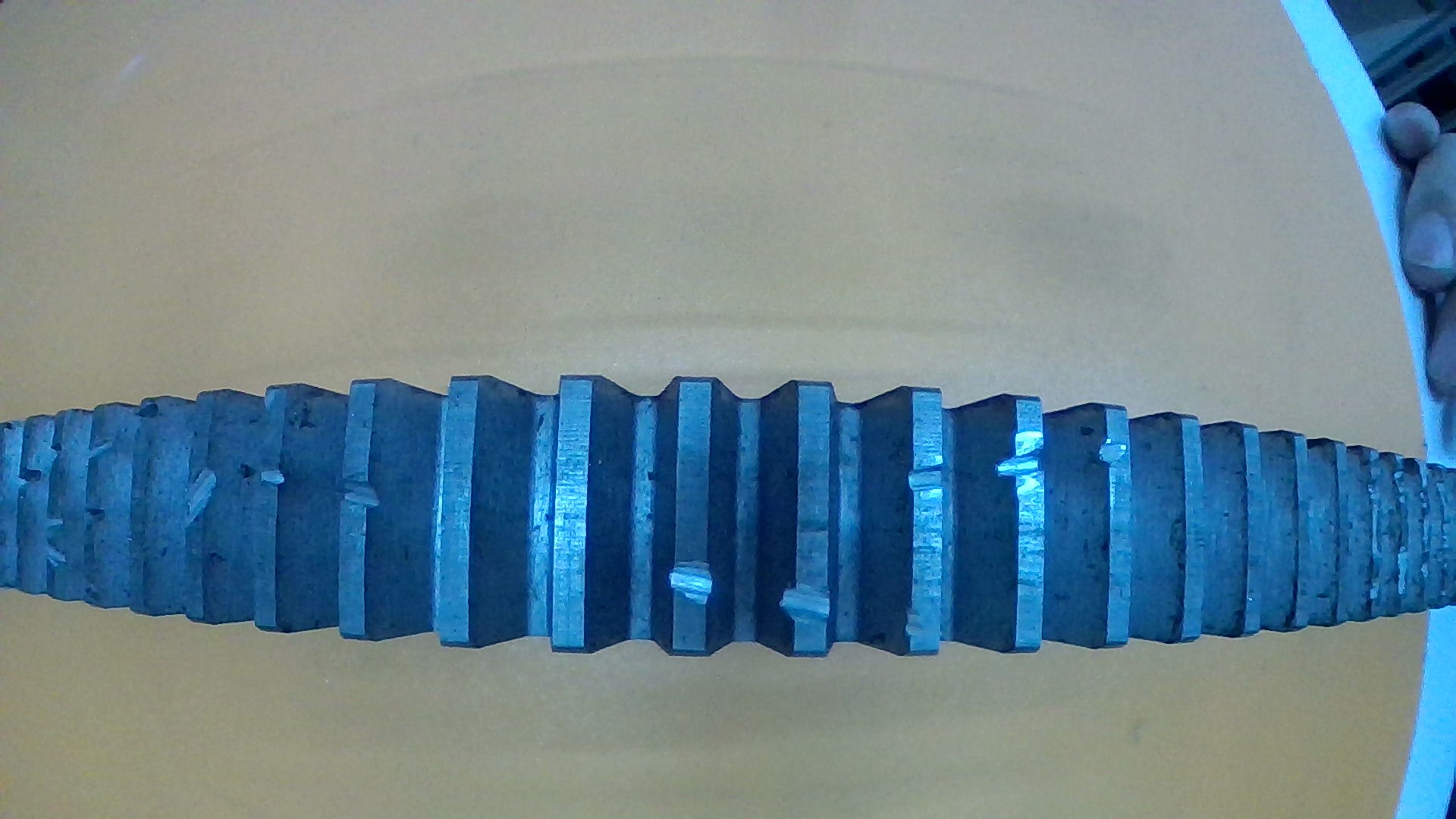

Supplement: S1 Data — (ZIP) [file pone.0322217.s001.zip › dataset/3/WIN_20250111_19_17_37_Pro.jpg]

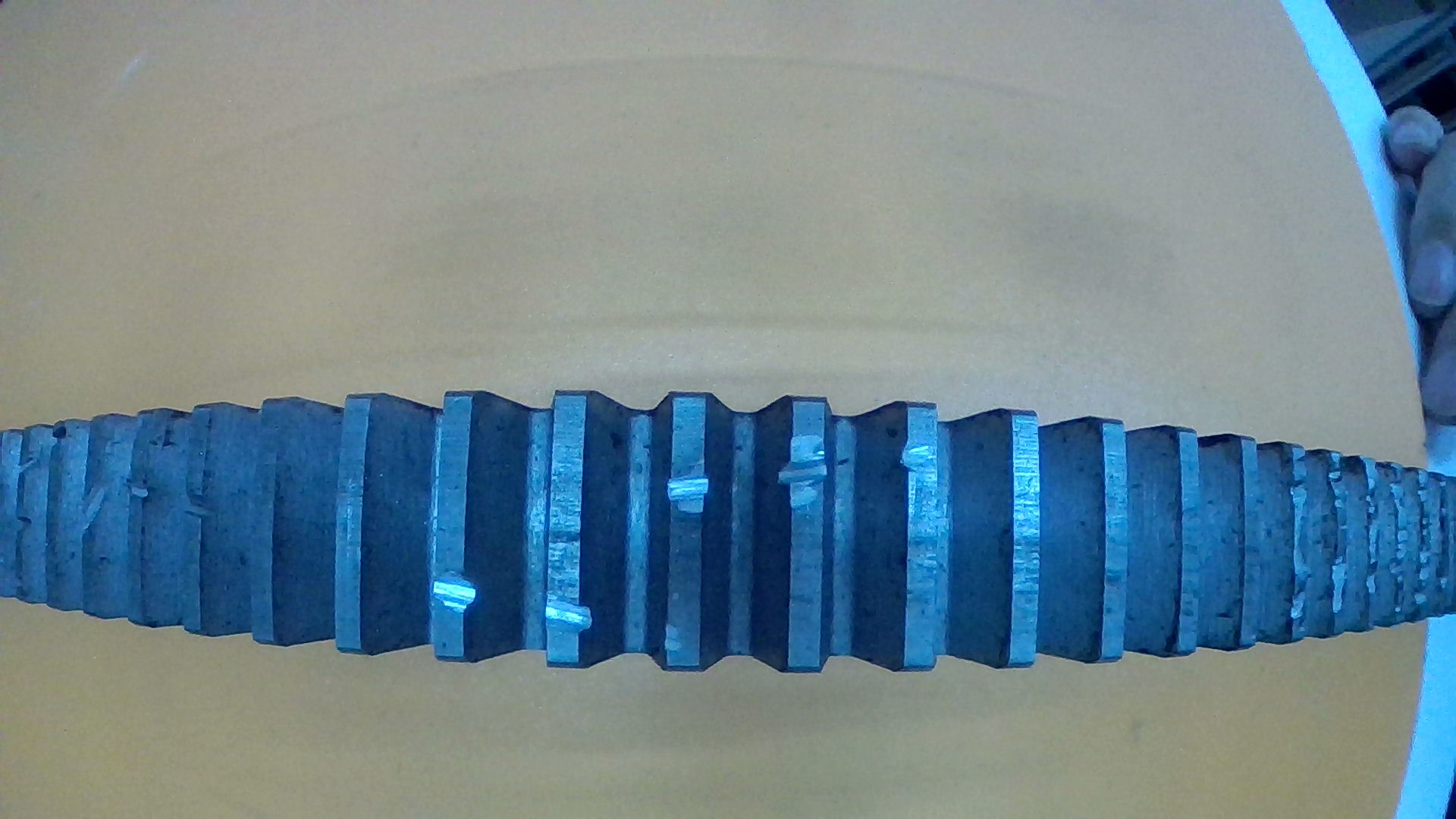

Supplement: S1 Data — (ZIP) [file pone.0322217.s001.zip › dataset/3/WIN_20250111_19_17_41_Pro.jpg]

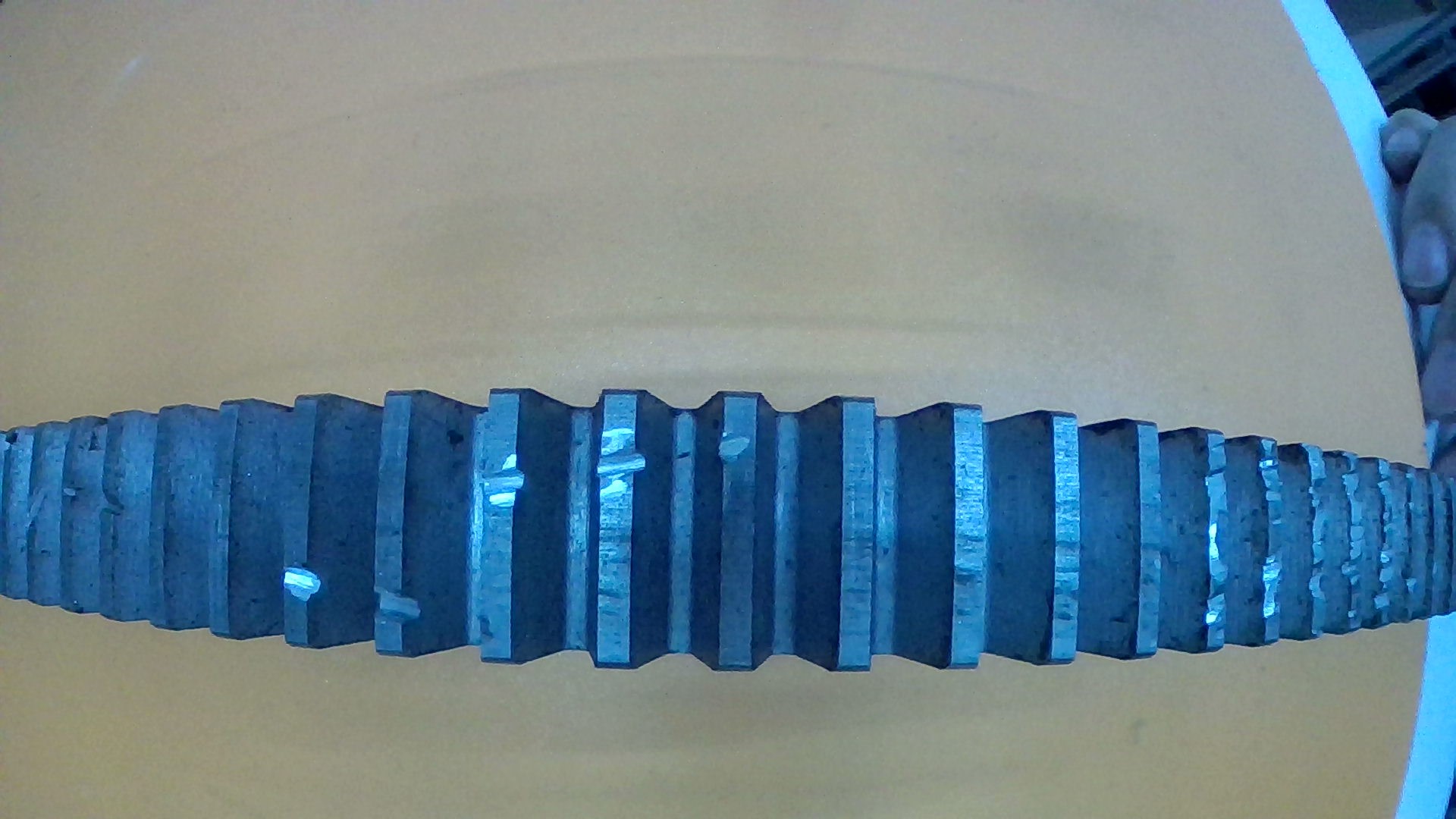

Supplement: S1 Data — (ZIP) [file pone.0322217.s001.zip › dataset/3/WIN_20250111_19_17_44_Pro.jpg]

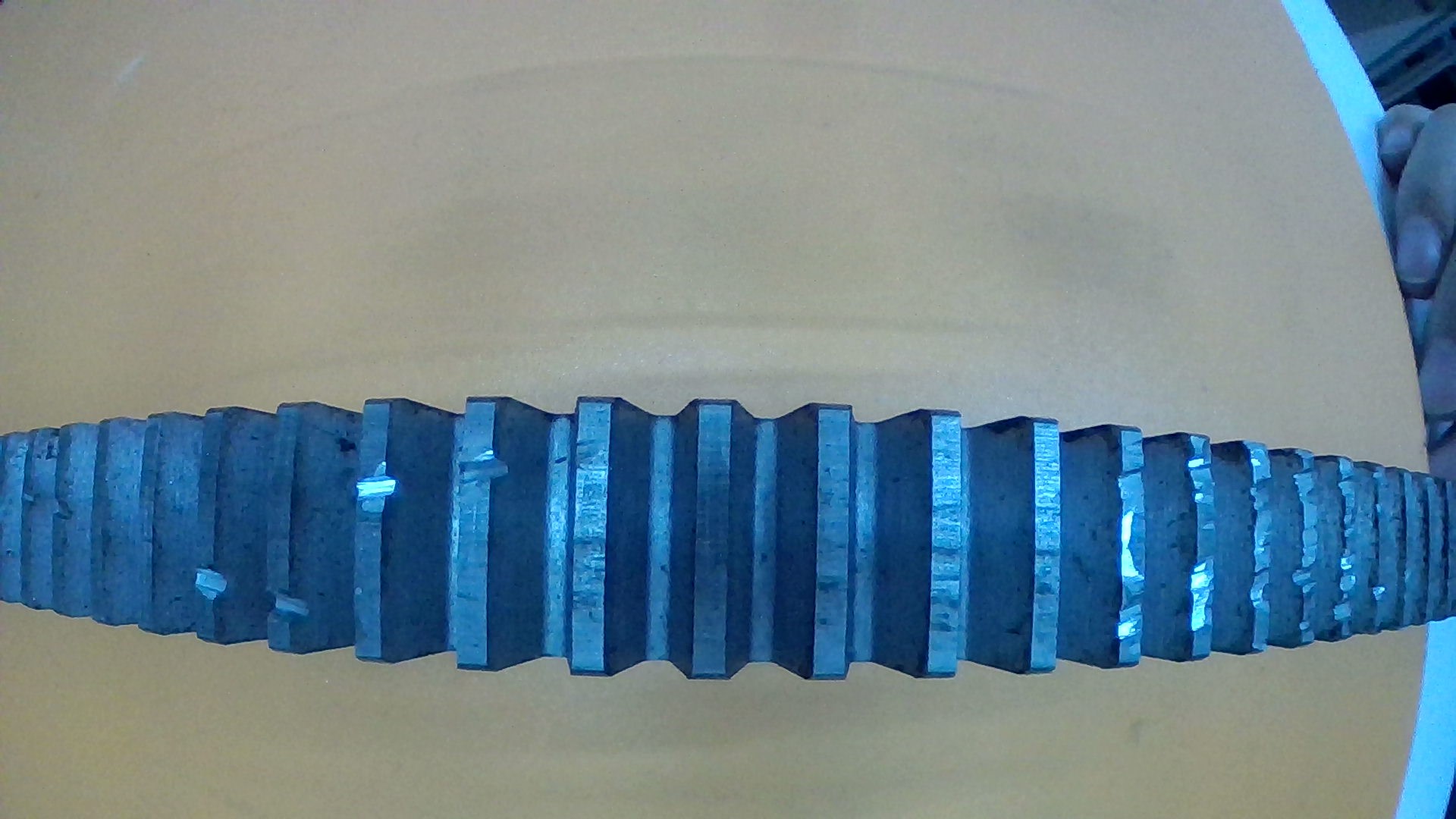

Supplement: S1 Data — (ZIP) [file pone.0322217.s001.zip › dataset/3/WIN_20250111_19_17_46_Pro.jpg]

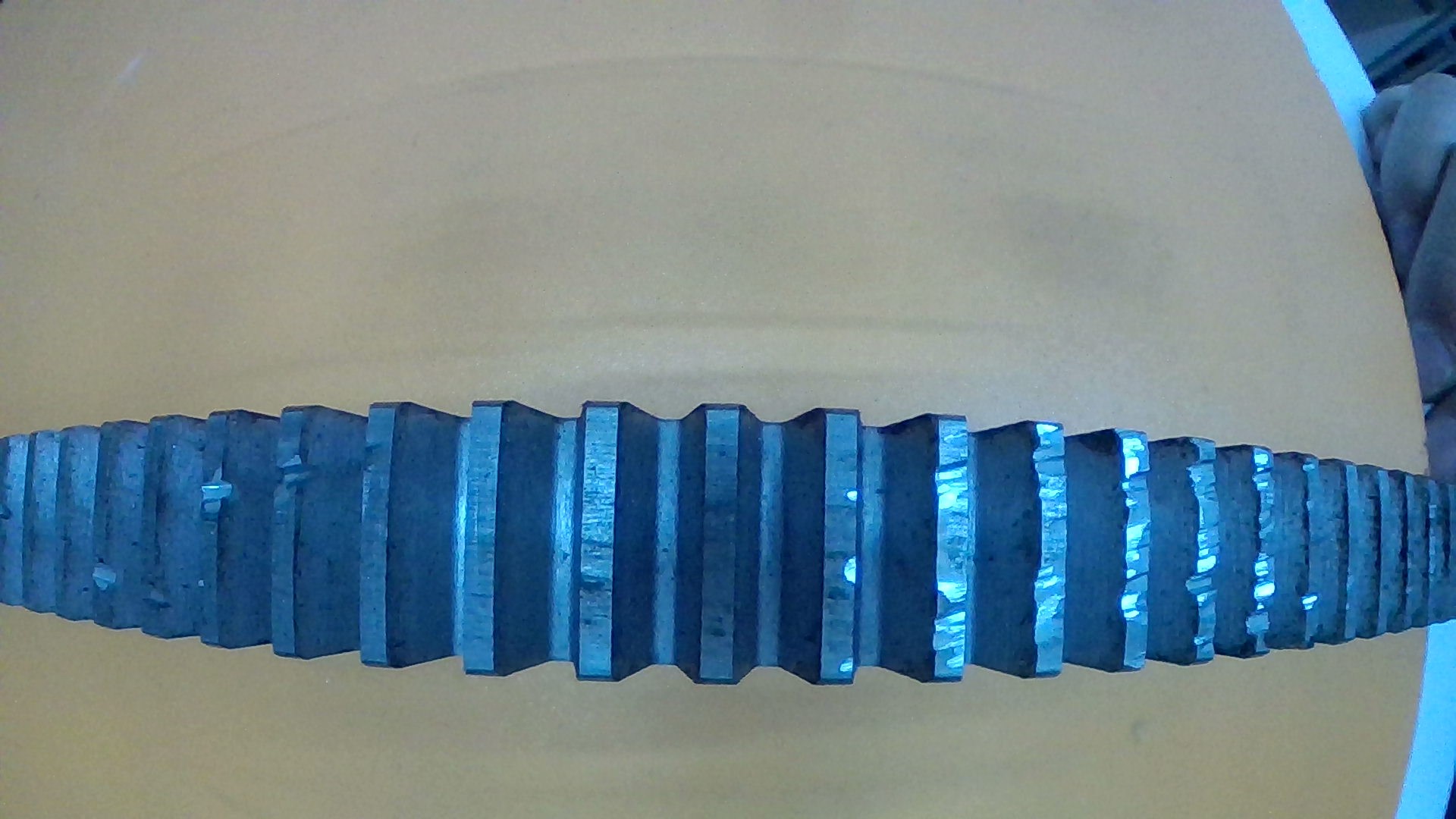

Supplement: S1 Data — (ZIP) [file pone.0322217.s001.zip › dataset/3/WIN_20250111_19_17_48_Pro.jpg]

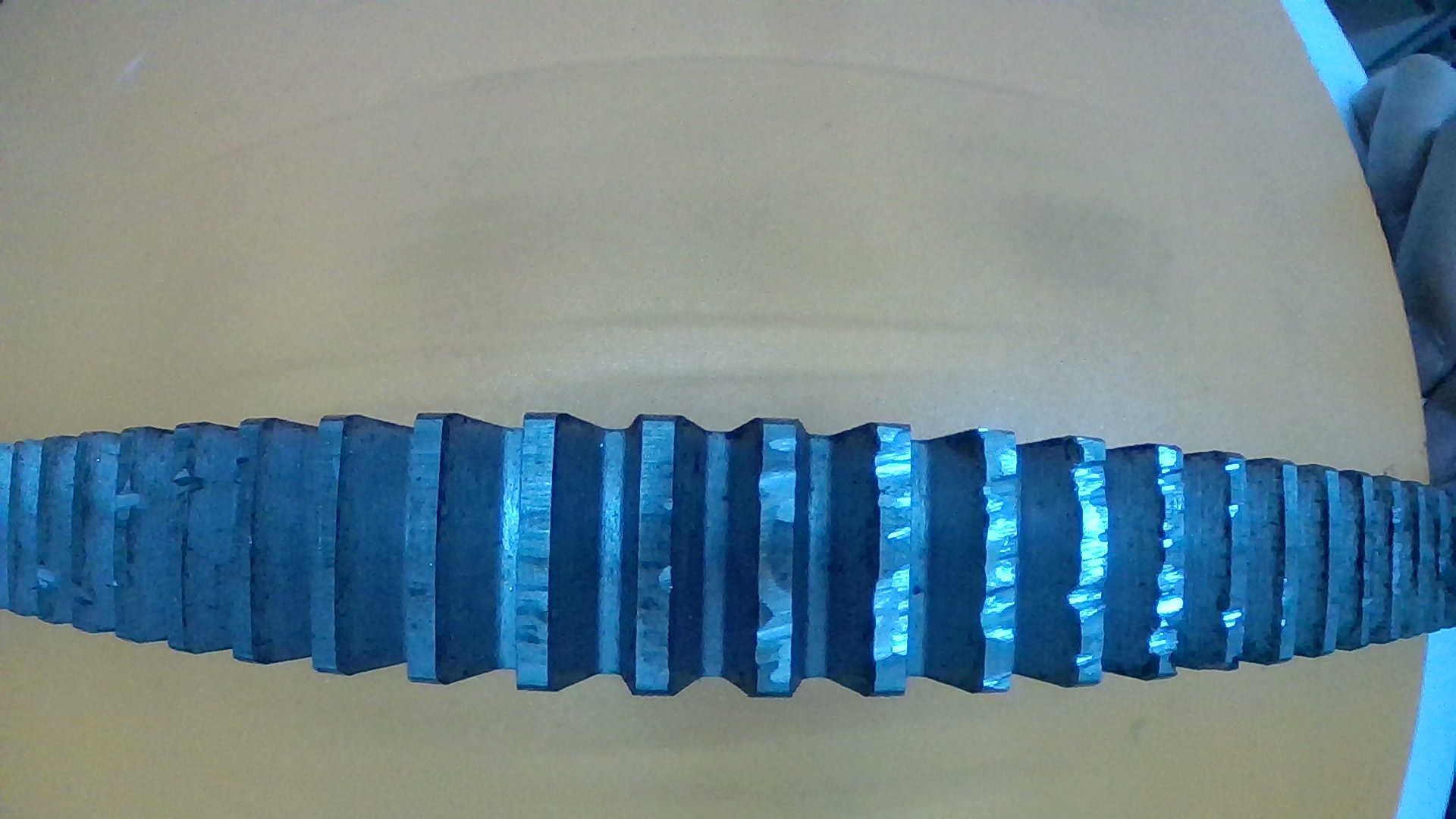

Supplement: S1 Data — (ZIP) [file pone.0322217.s001.zip › dataset/3/WIN_20250111_19_17_50_Pro.jpg]

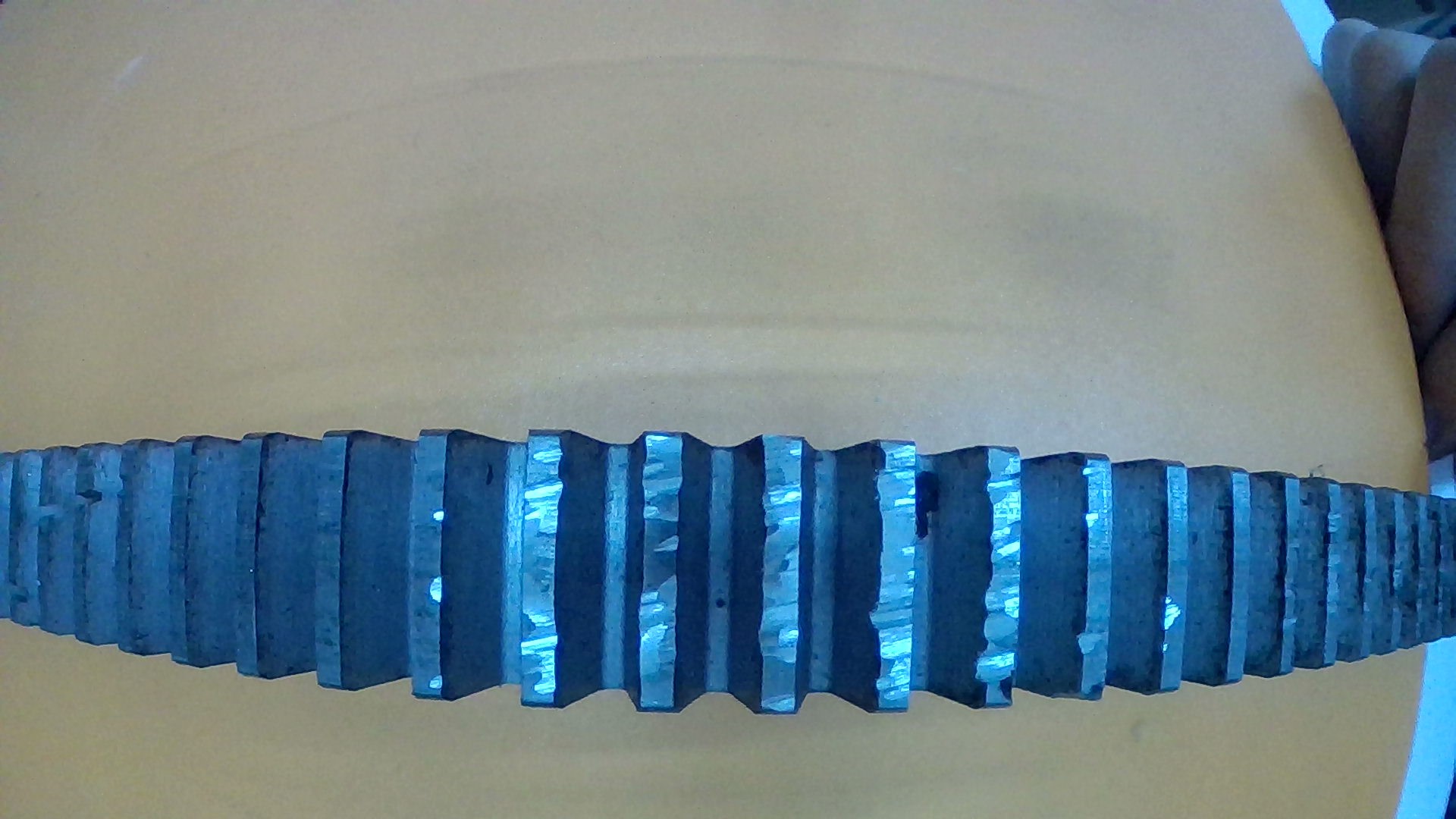

Supplement: S1 Data — (ZIP) [file pone.0322217.s001.zip › dataset/3/WIN_20250111_19_17_53_Pro.jpg]

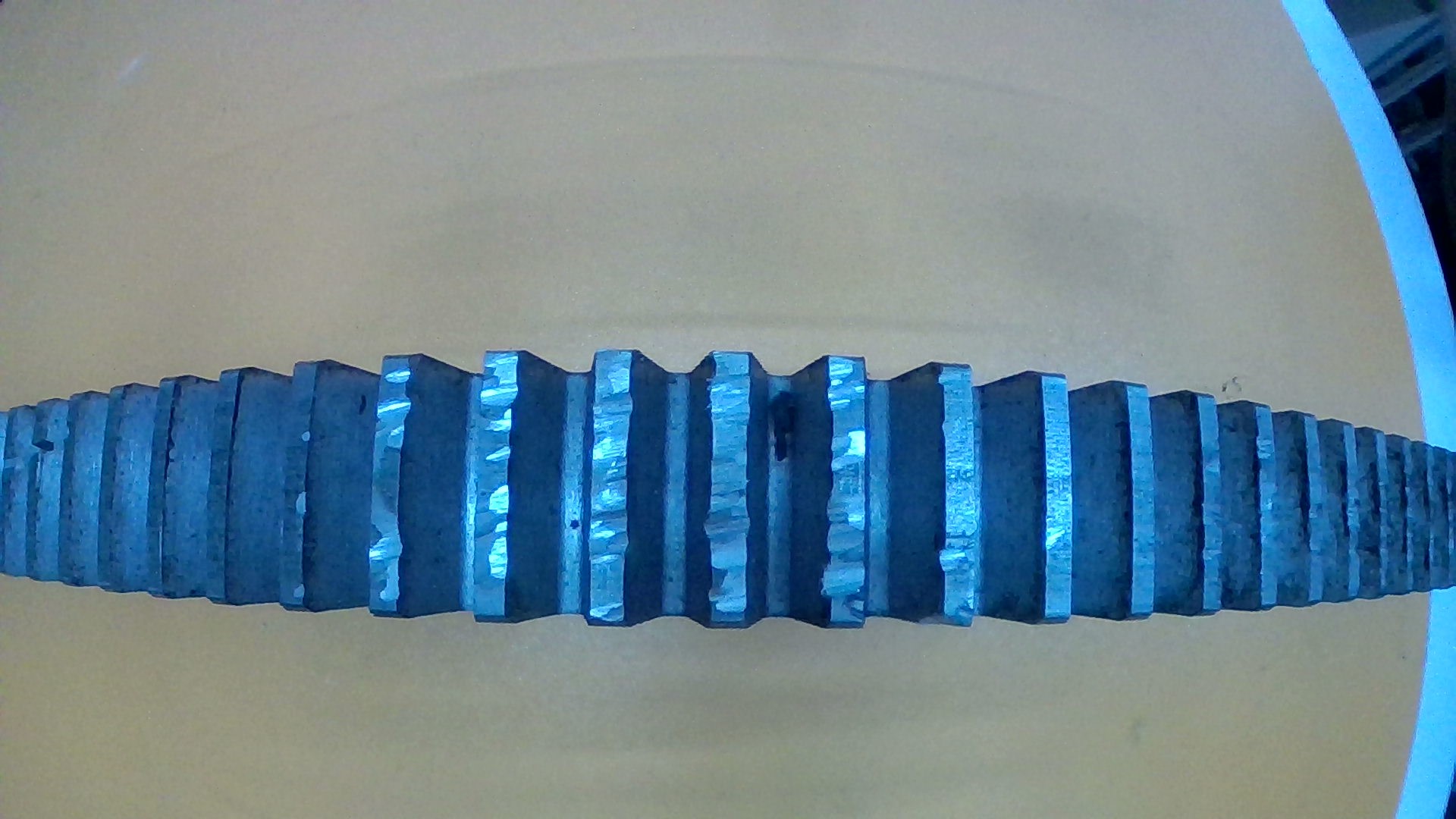

Supplement: S1 Data — (ZIP) [file pone.0322217.s001.zip › dataset/3/WIN_20250111_19_17_57_Pro.jpg]

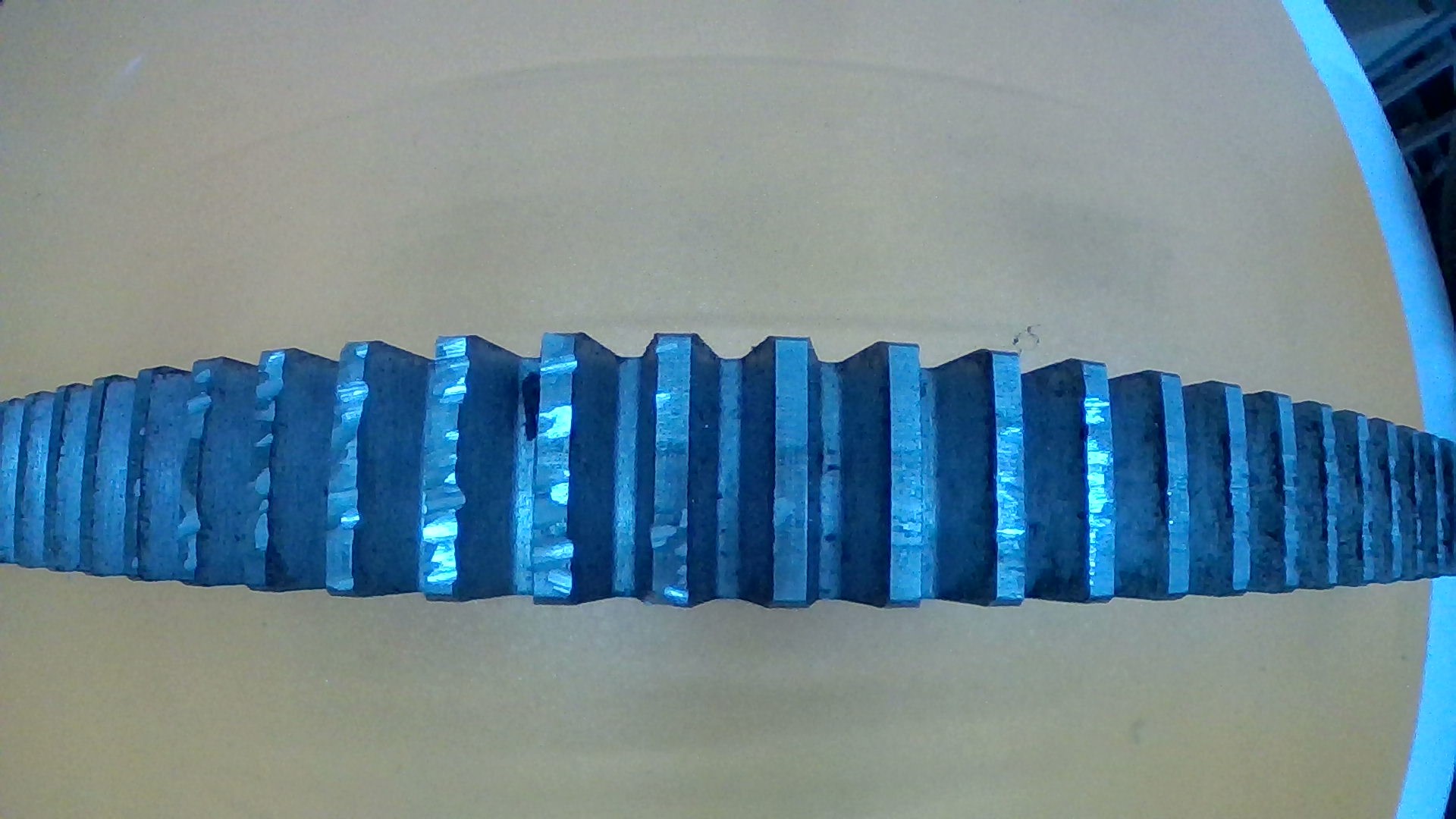

Supplement: S1 Data — (ZIP) [file pone.0322217.s001.zip › dataset/3/WIN_20250111_19_18_01_Pro.jpg]

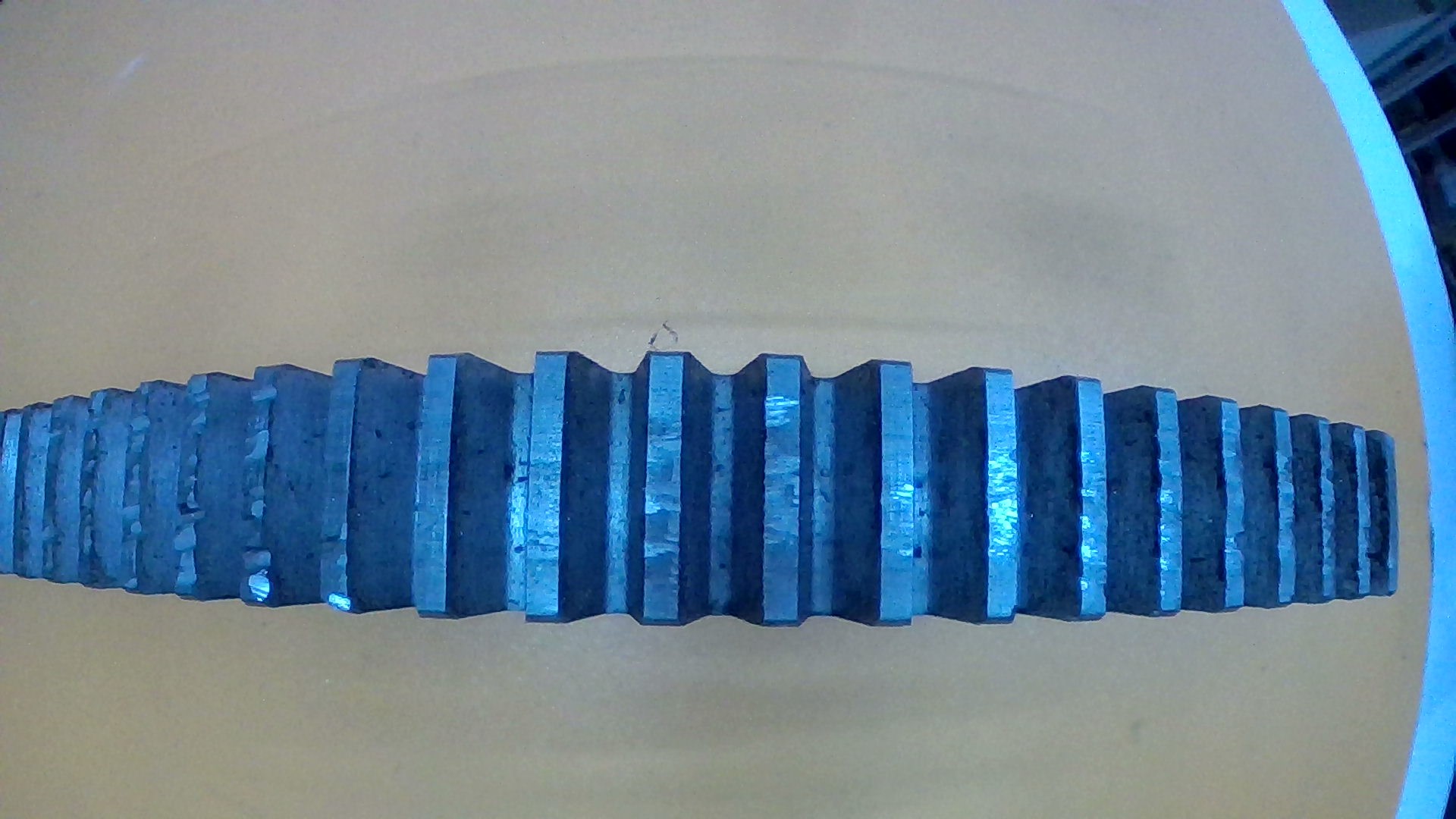

Supplement: S1 Data — (ZIP) [file pone.0322217.s001.zip › dataset/3/WIN_20250111_19_18_06_Pro.jpg]

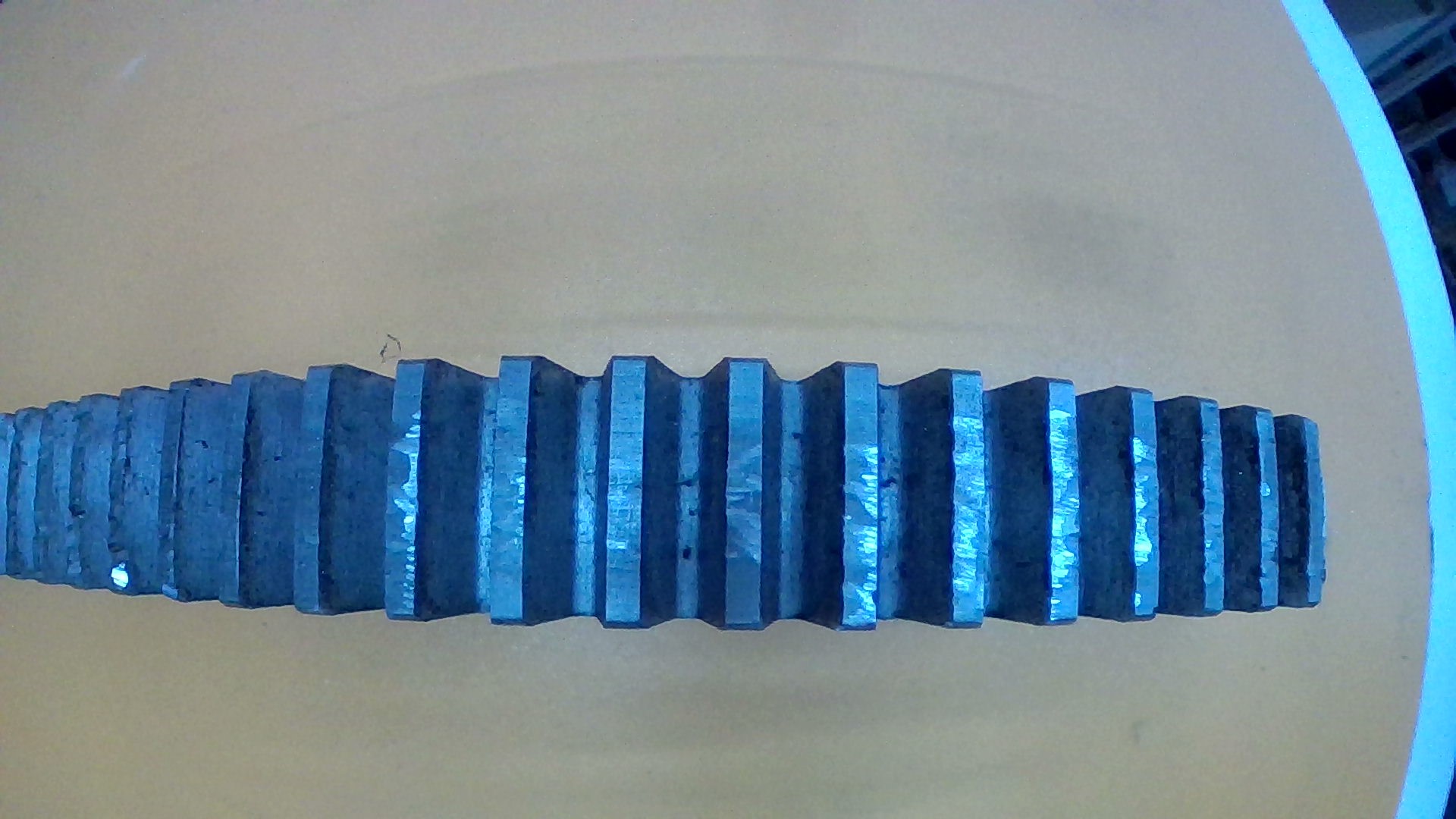

Supplement: S1 Data — (ZIP) [file pone.0322217.s001.zip › dataset/3/WIN_20250111_19_18_10_Pro.jpg]

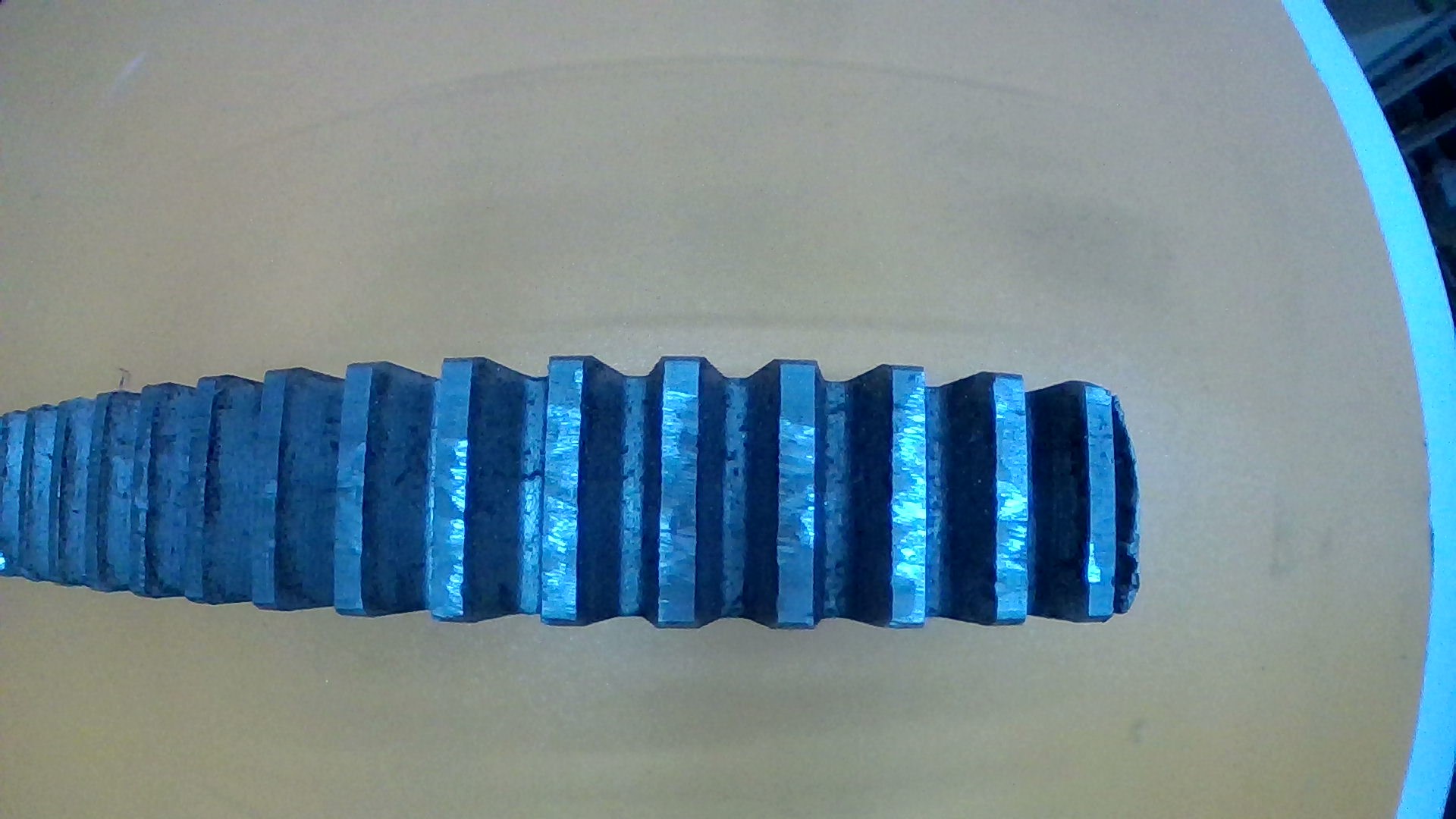

Supplement: S1 Data — (ZIP) [file pone.0322217.s001.zip › dataset/3/WIN_20250111_19_18_15_Pro.jpg]

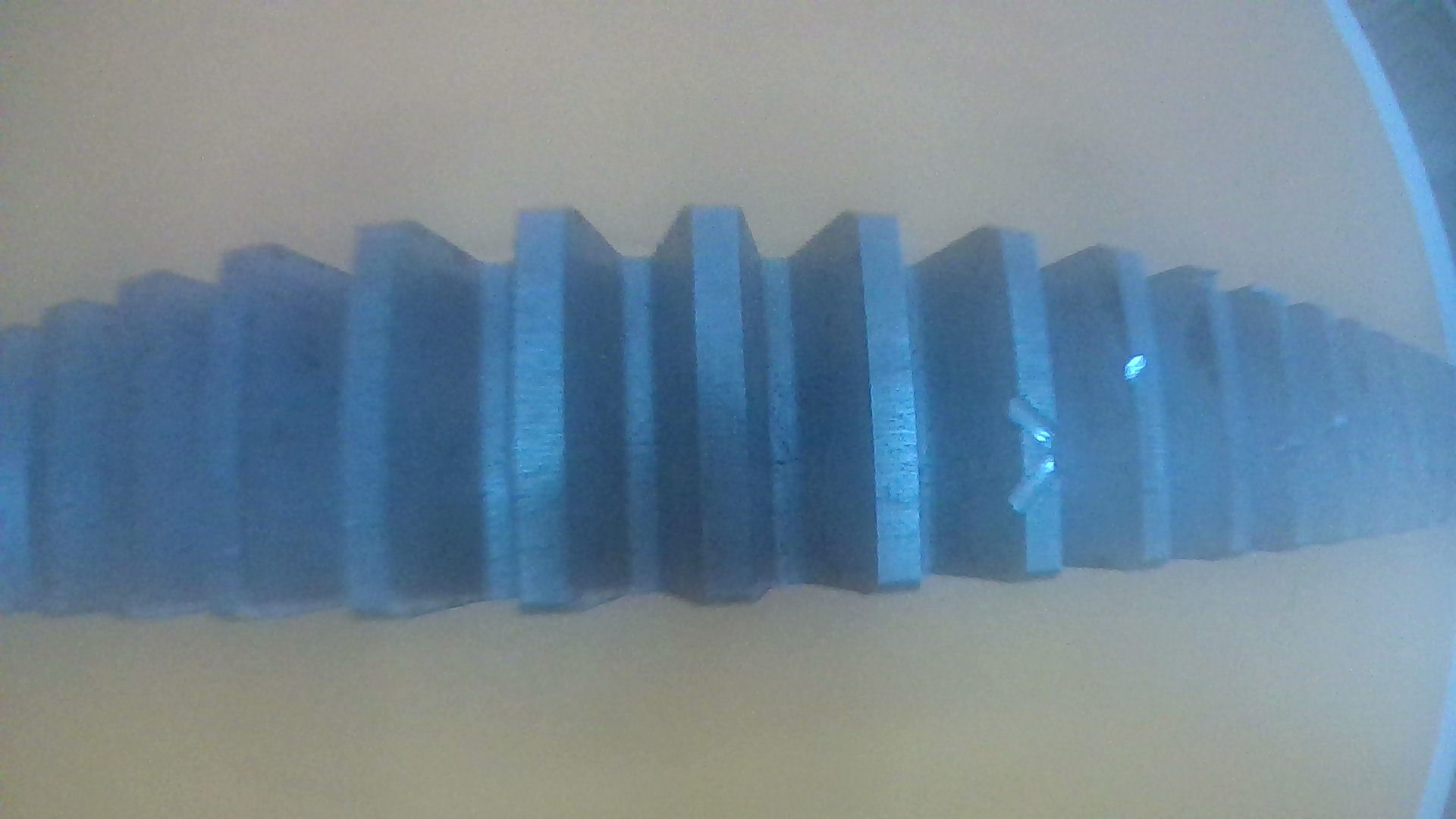

Supplement: S1 Data — (ZIP) [file pone.0322217.s001.zip › dataset/3/WIN_20250111_20_48_42_Pro.jpg]

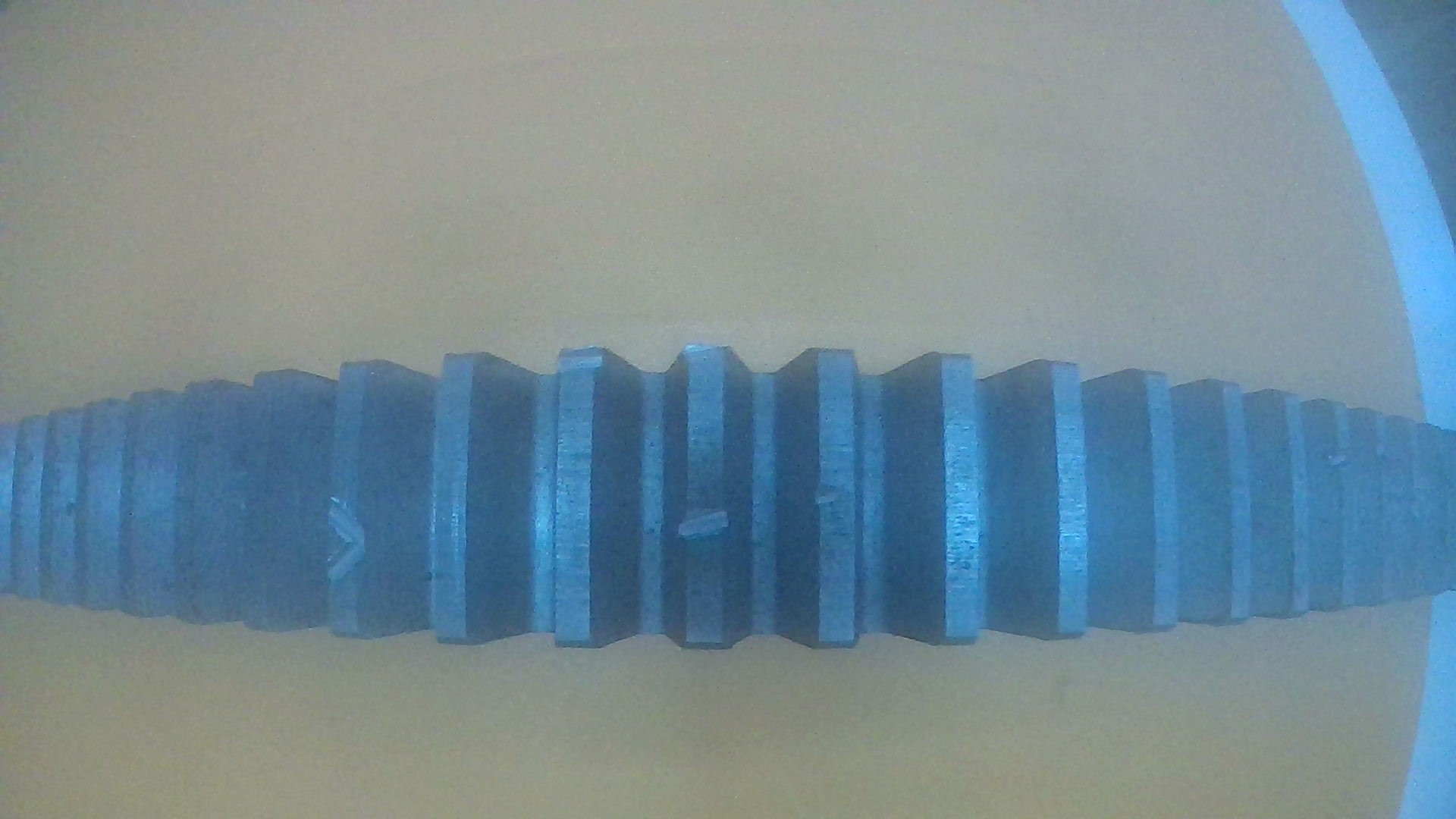

Supplement: S1 Data — (ZIP) [file pone.0322217.s001.zip › dataset/3/WIN_20250111_20_49_01_Pro.jpg]

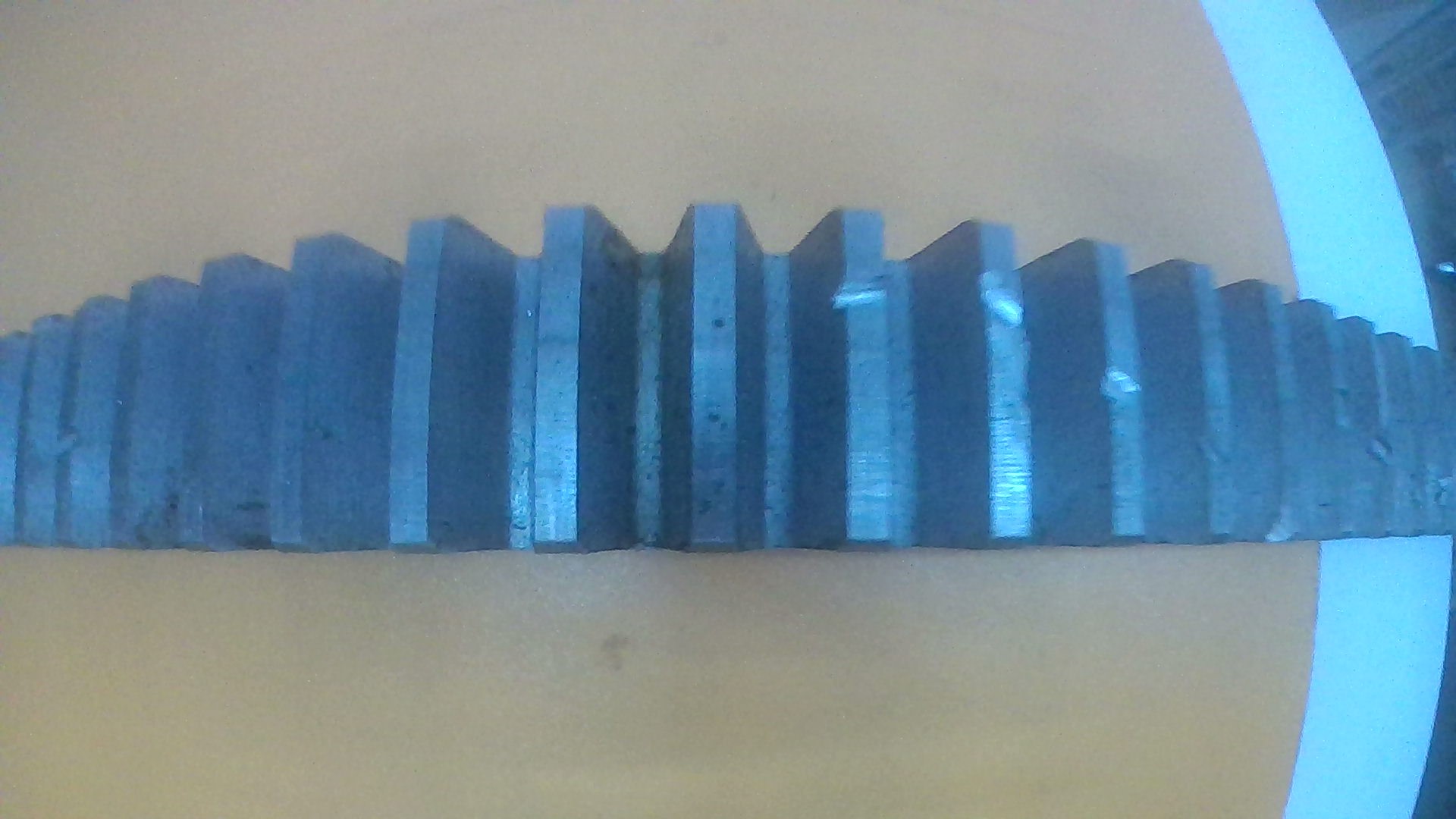

Supplement: S1 Data — (ZIP) [file pone.0322217.s001.zip › dataset/3/WIN_20250111_20_49_05_Pro.jpg]

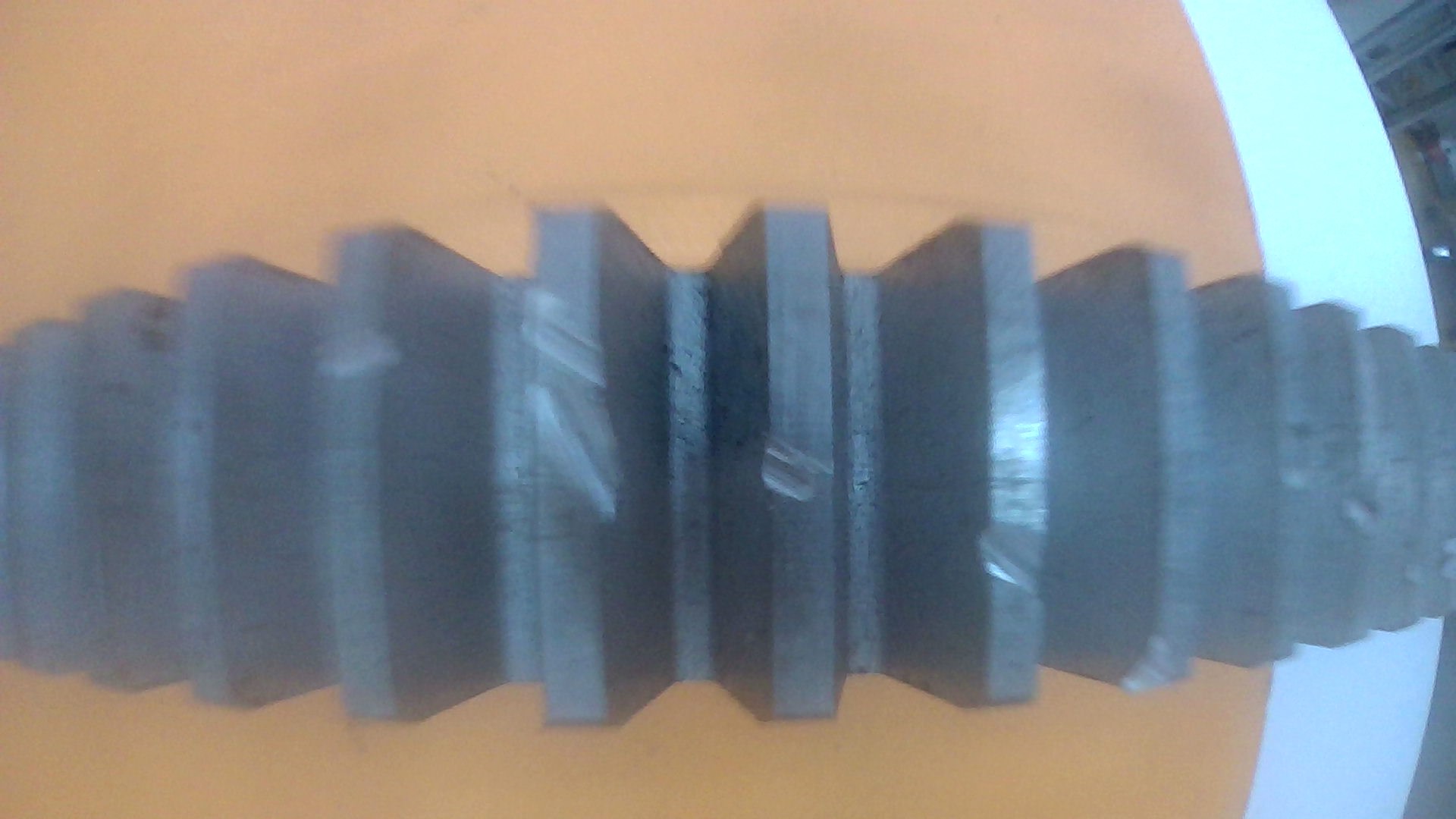

Supplement: S1 Data — (ZIP) [file pone.0322217.s001.zip › dataset/3/WIN_20250111_20_49_13_Pro.jpg]

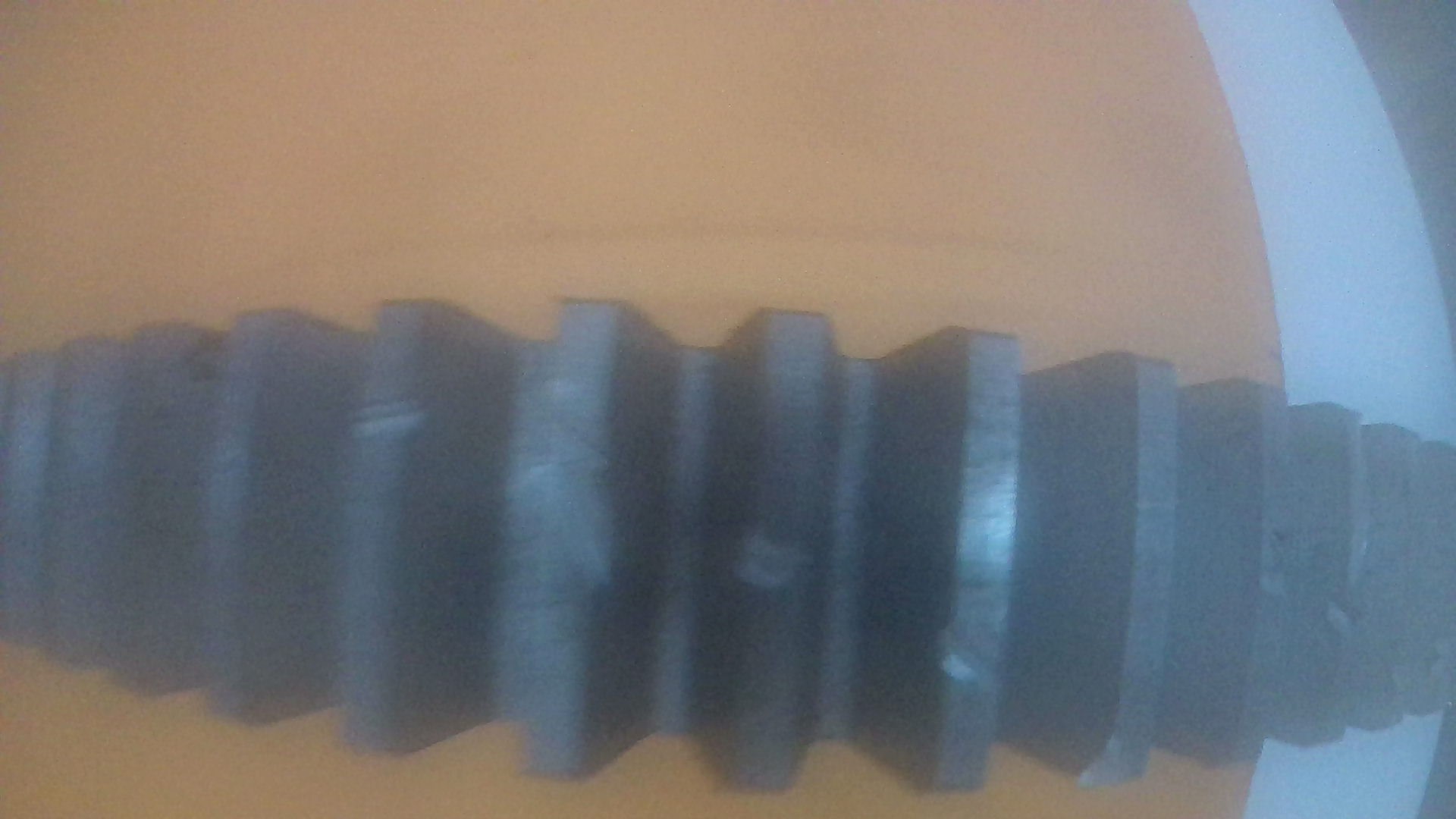

Supplement: S1 Data — (ZIP) [file pone.0322217.s001.zip › dataset/3/WIN_20250111_20_49_19_Pro.jpg]

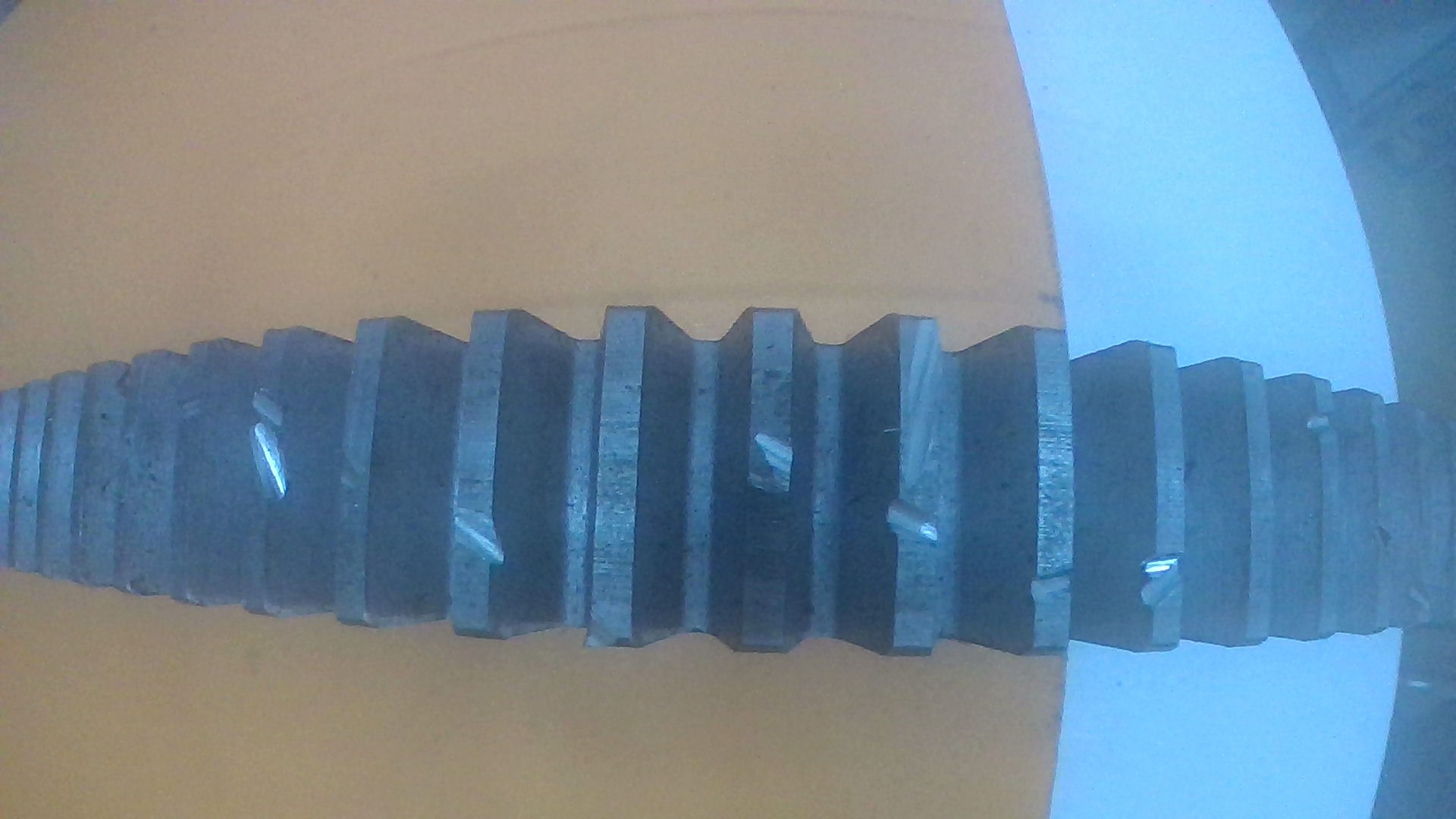

Supplement: S1 Data — (ZIP) [file pone.0322217.s001.zip › dataset/3/WIN_20250111_20_49_23_Pro.jpg]

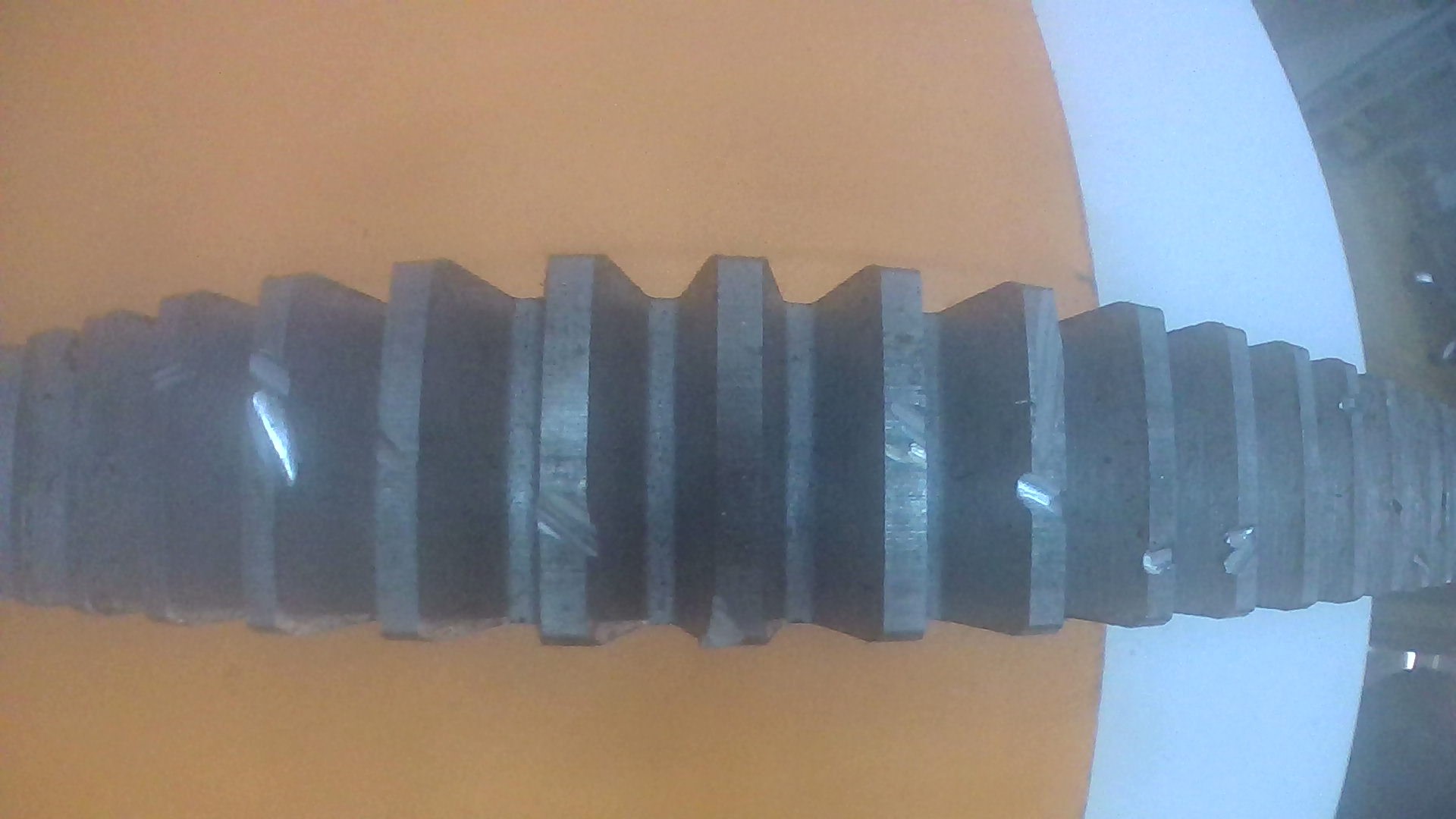

Supplement: S1 Data — (ZIP) [file pone.0322217.s001.zip › dataset/3/WIN_20250111_20_49_29_Pro.jpg]

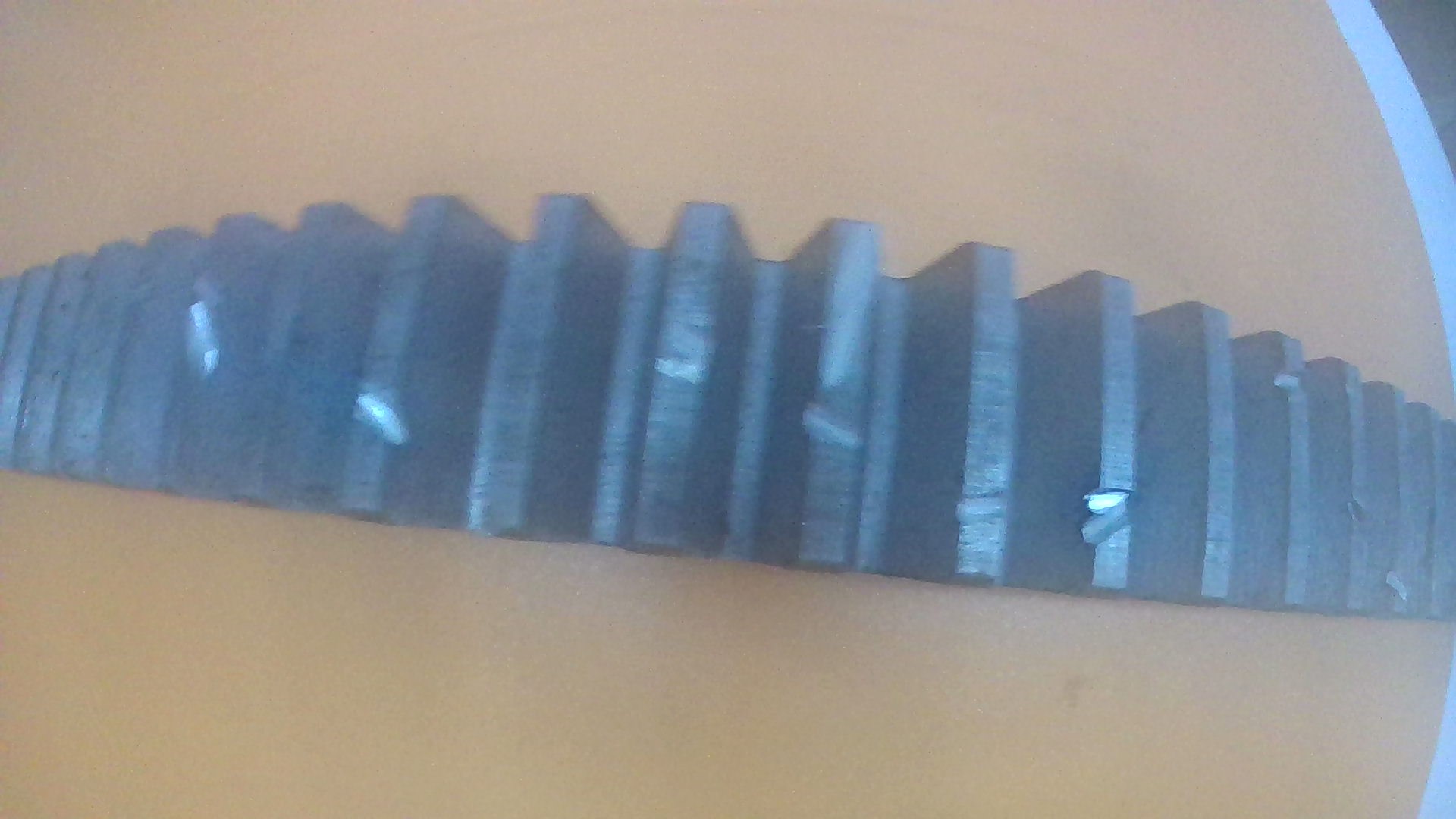

Supplement: S1 Data — (ZIP) [file pone.0322217.s001.zip › dataset/3/WIN_20250111_20_49_42_Pro.jpg]

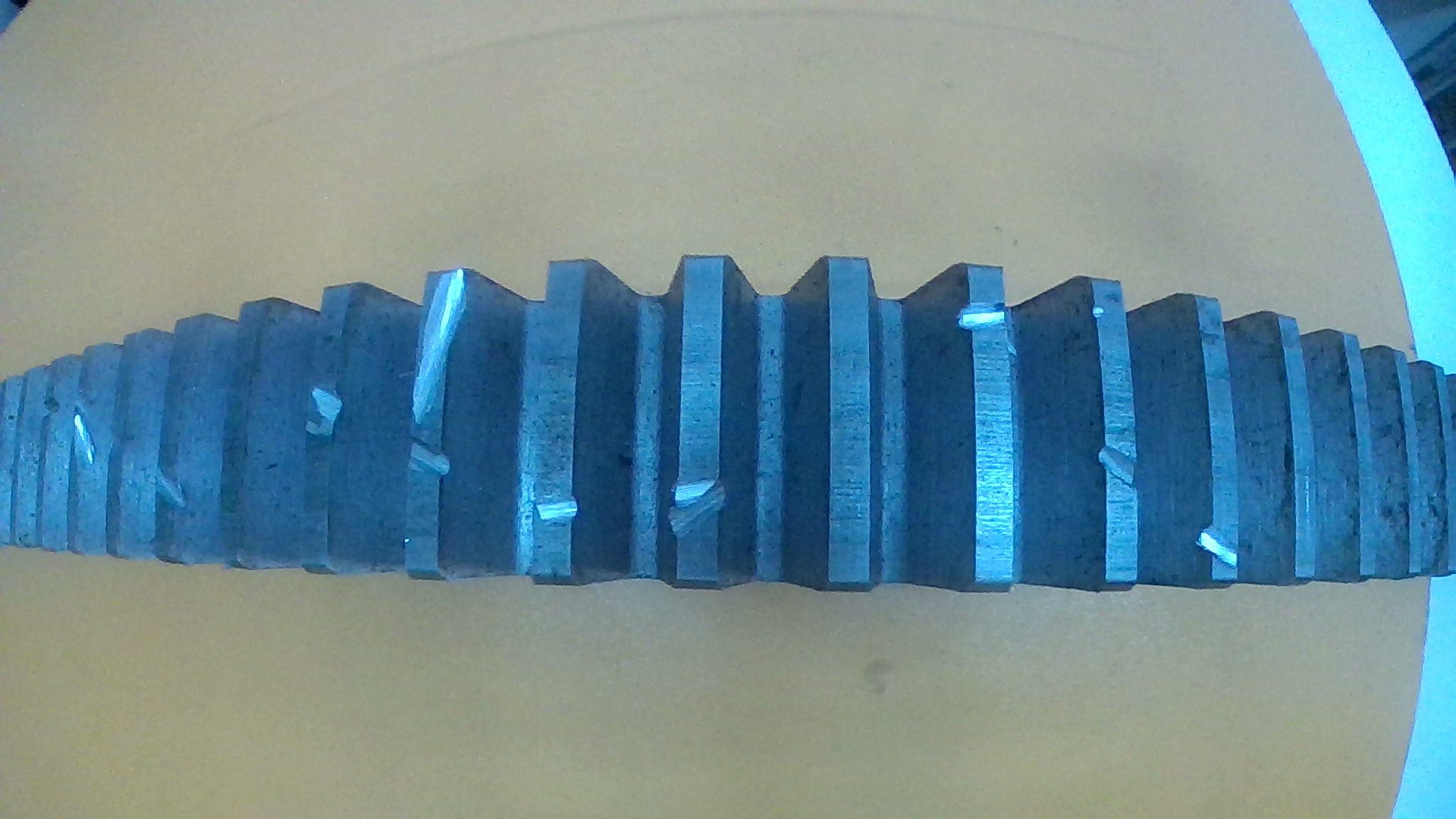

Supplement: S1 Data — (ZIP) [file pone.0322217.s001.zip › dataset/3/WIN_20250111_20_49_44_Pro.jpg]

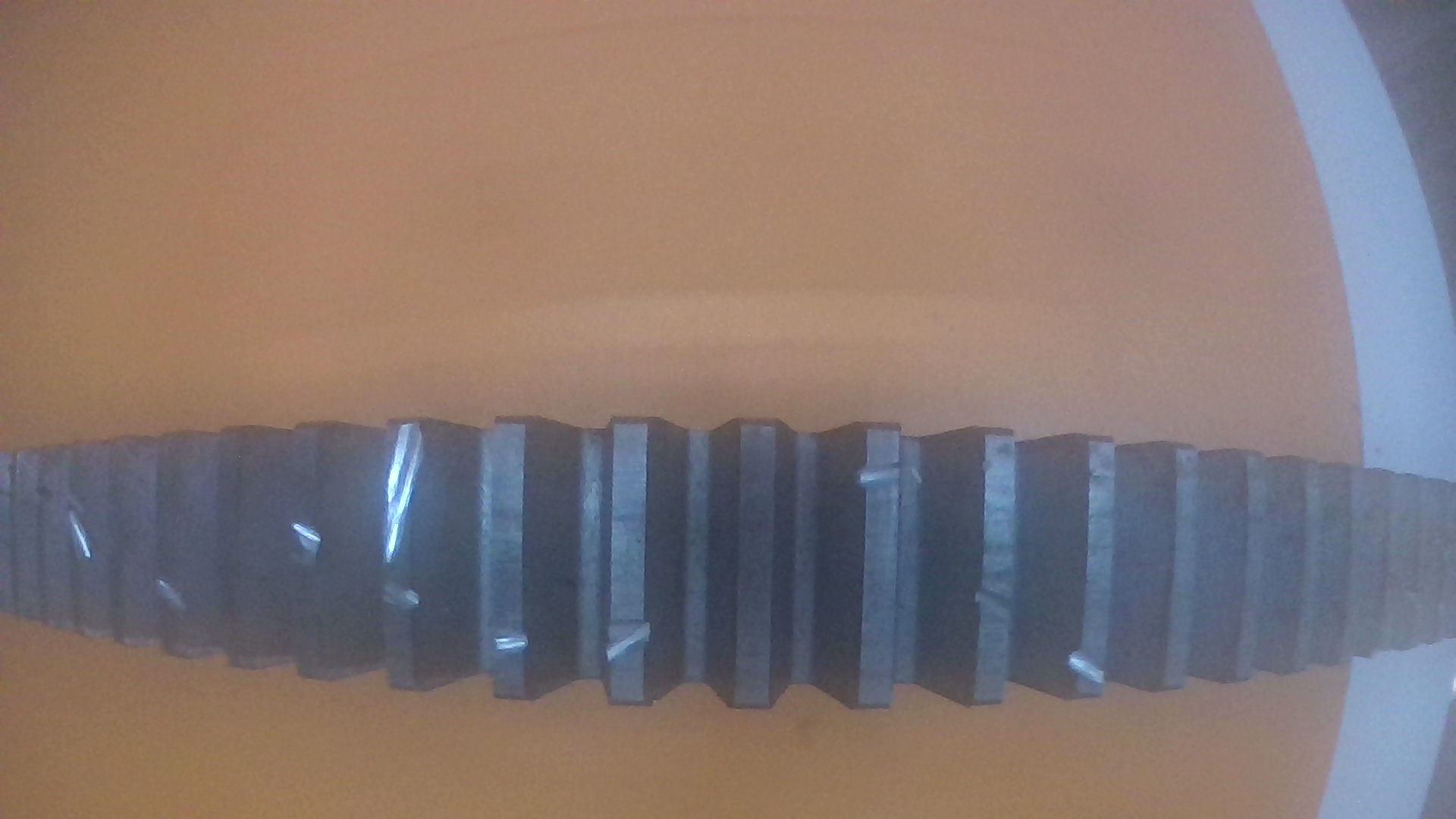

Supplement: S1 Data — (ZIP) [file pone.0322217.s001.zip › dataset/3/WIN_20250111_20_49_48_Pro.jpg]

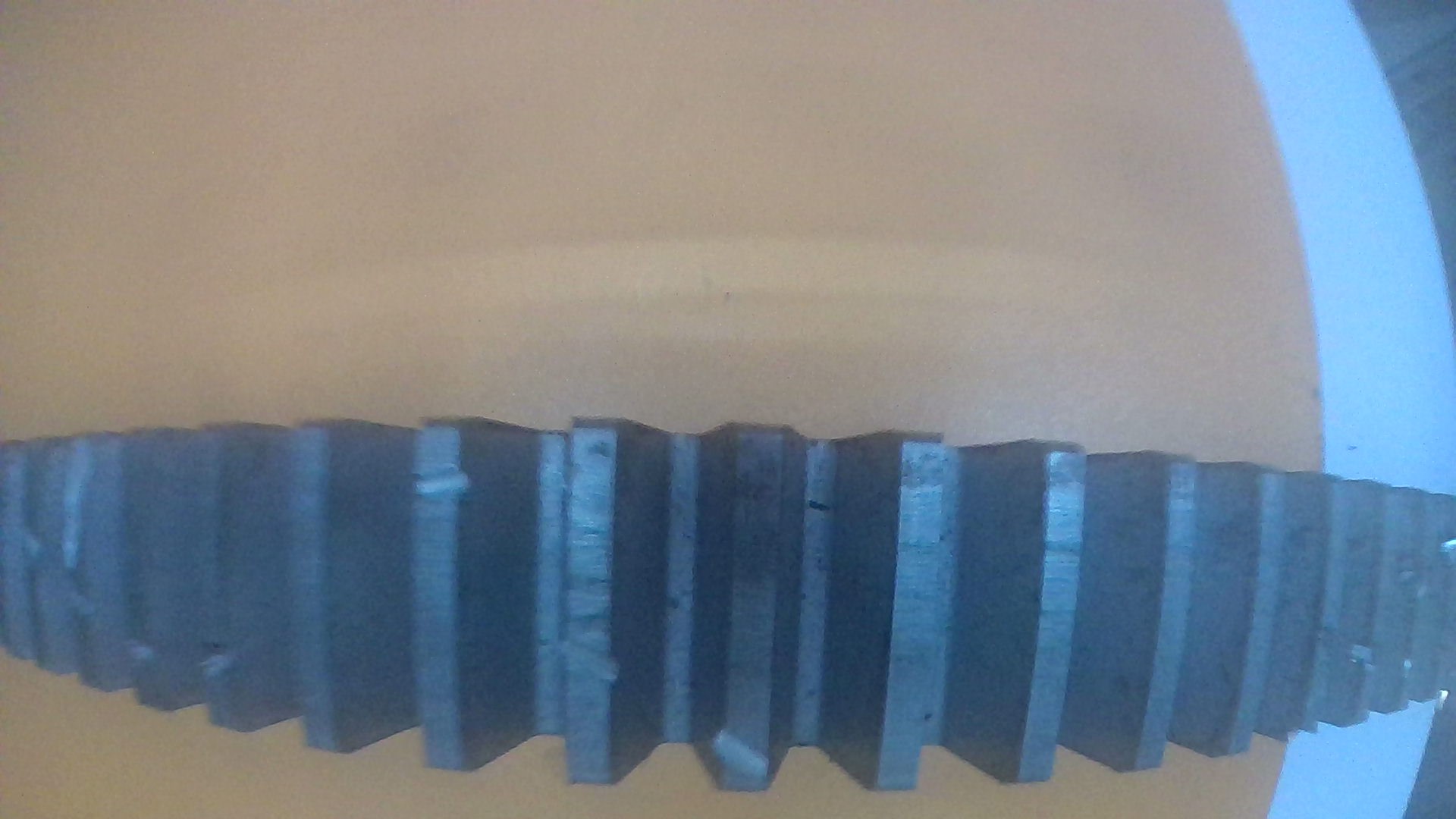

Supplement: S1 Data — (ZIP) [file pone.0322217.s001.zip › dataset/3/WIN_20250111_20_49_49_Pro.jpg]

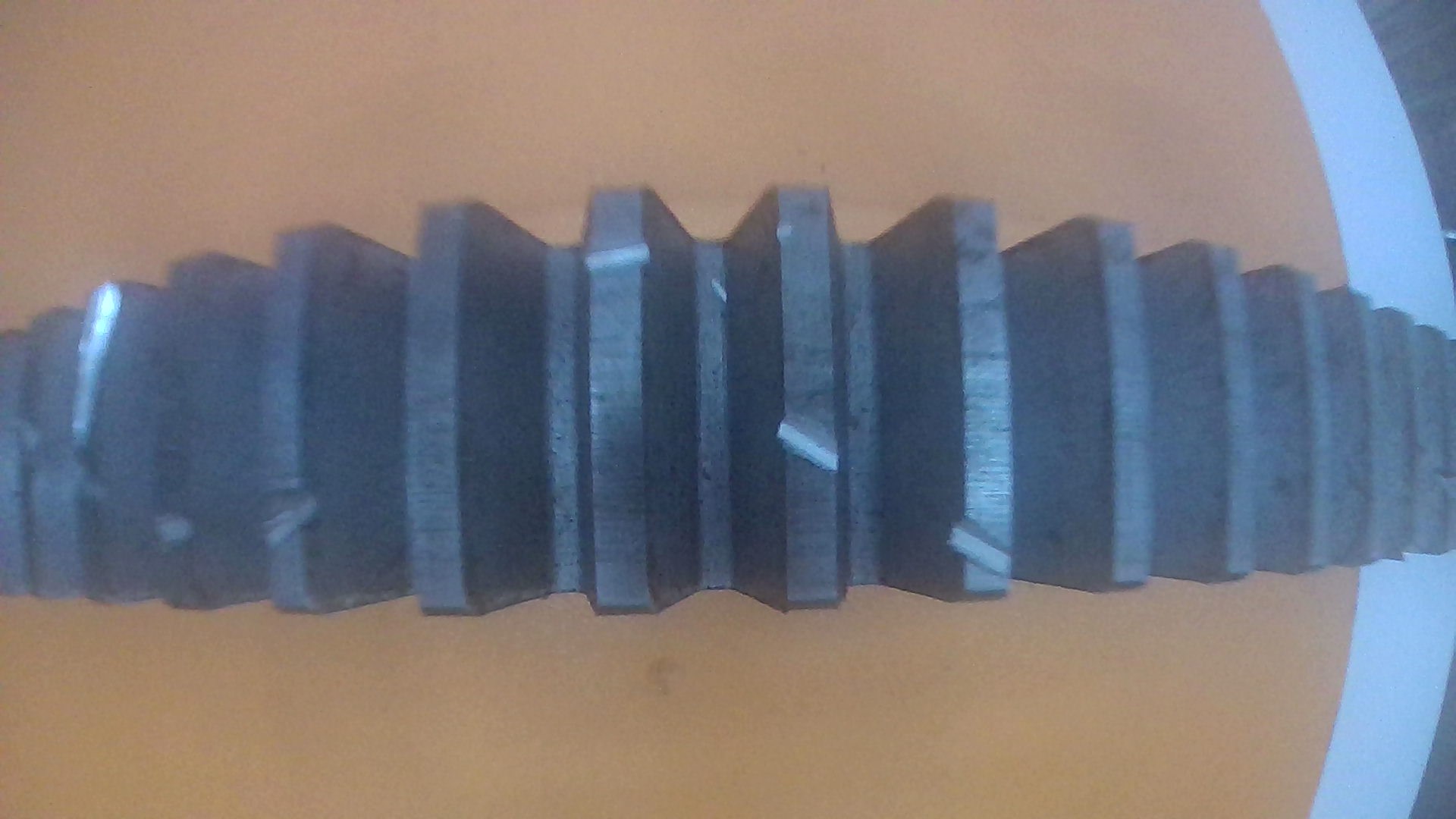

Supplement: S1 Data — (ZIP) [file pone.0322217.s001.zip › dataset/3/WIN_20250111_20_49_54_Pro.jpg]

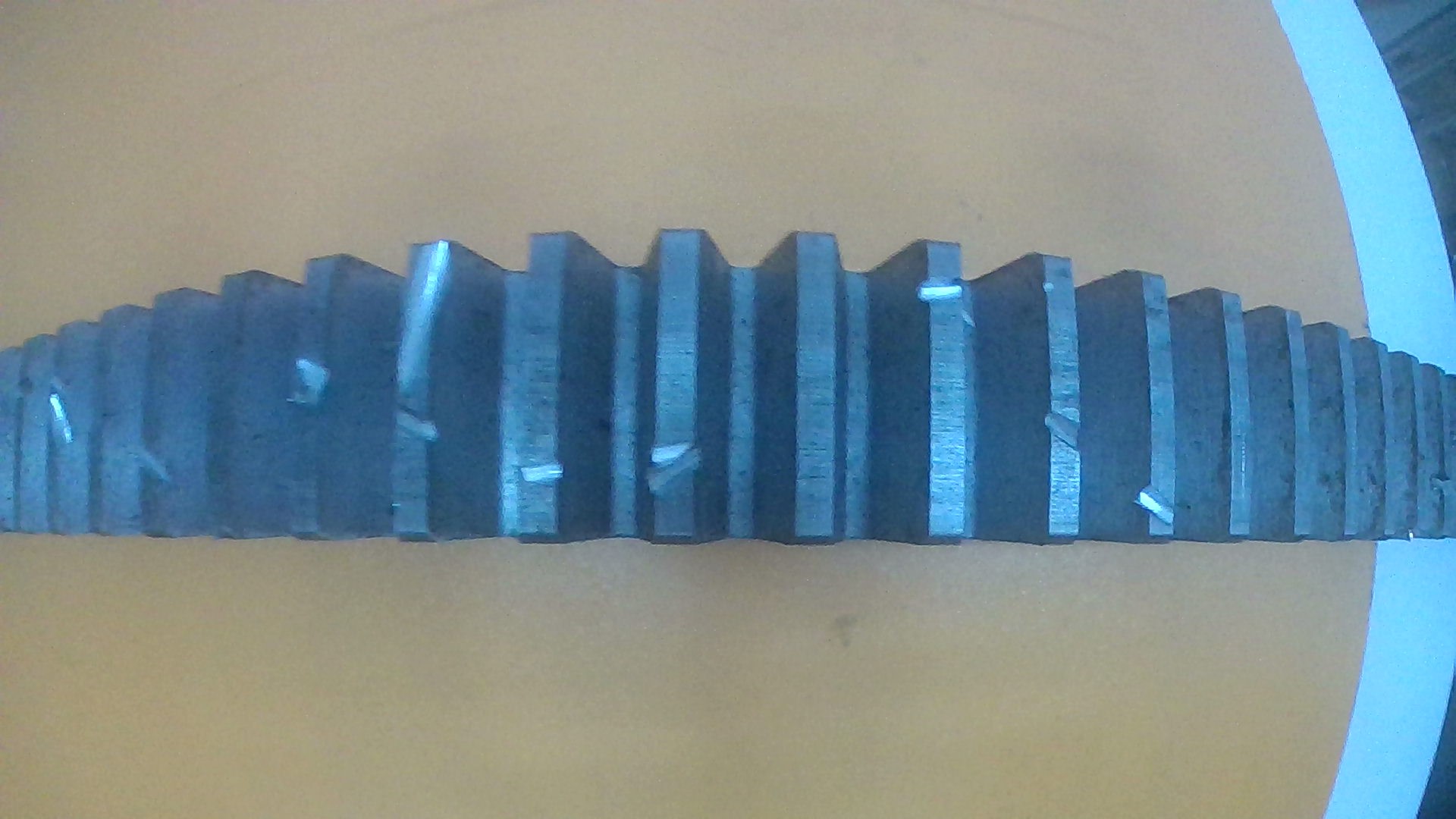

Supplement: S1 Data — (ZIP) [file pone.0322217.s001.zip › dataset/3/WIN_20250111_20_49_55_Pro.jpg]

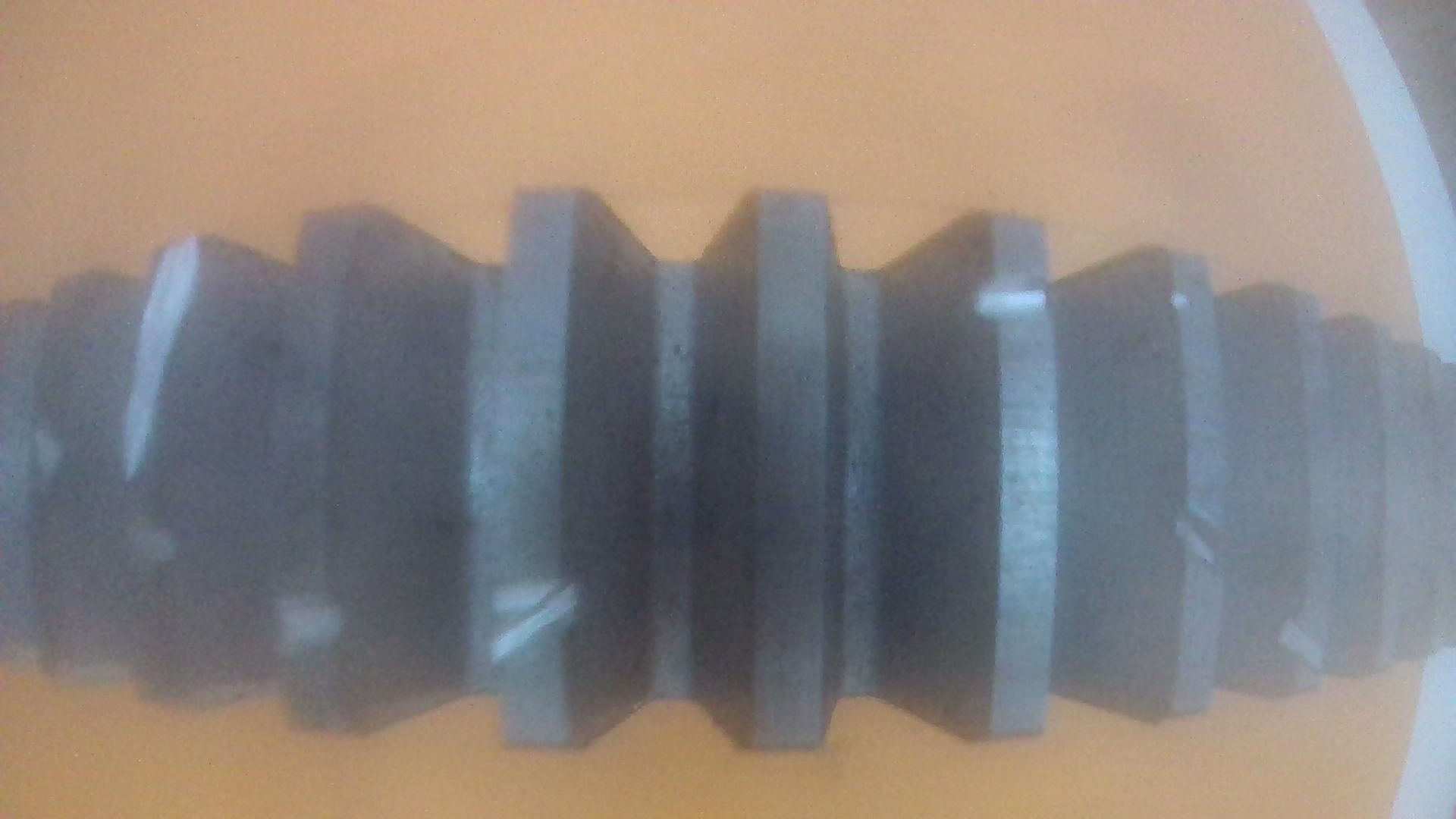

Supplement: S1 Data — (ZIP) [file pone.0322217.s001.zip › dataset/3/WIN_20250111_20_50_16_Pro.jpg]

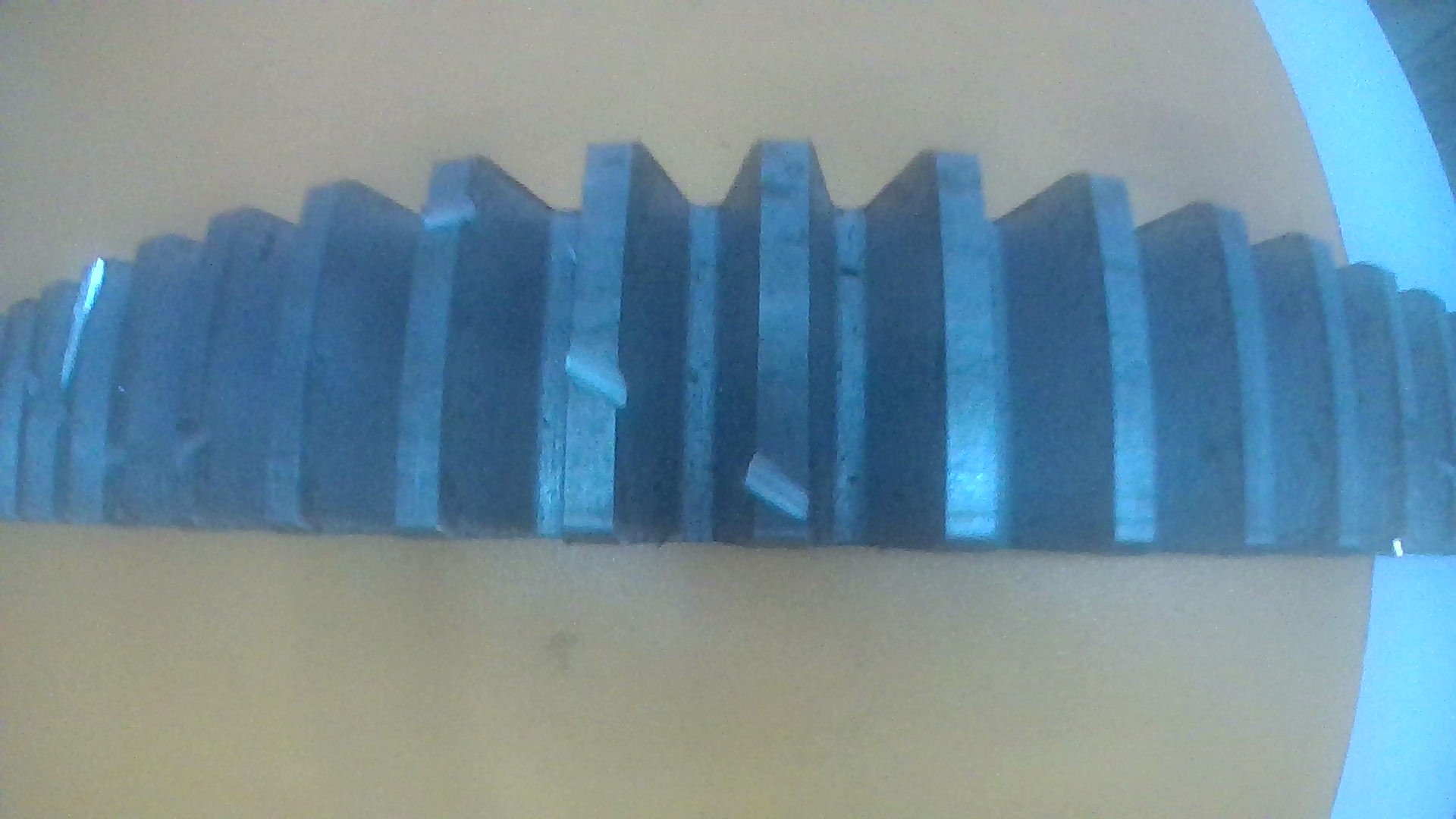

Supplement: S1 Data — (ZIP) [file pone.0322217.s001.zip › dataset/3/WIN_20250111_20_50_19_Pro.jpg]

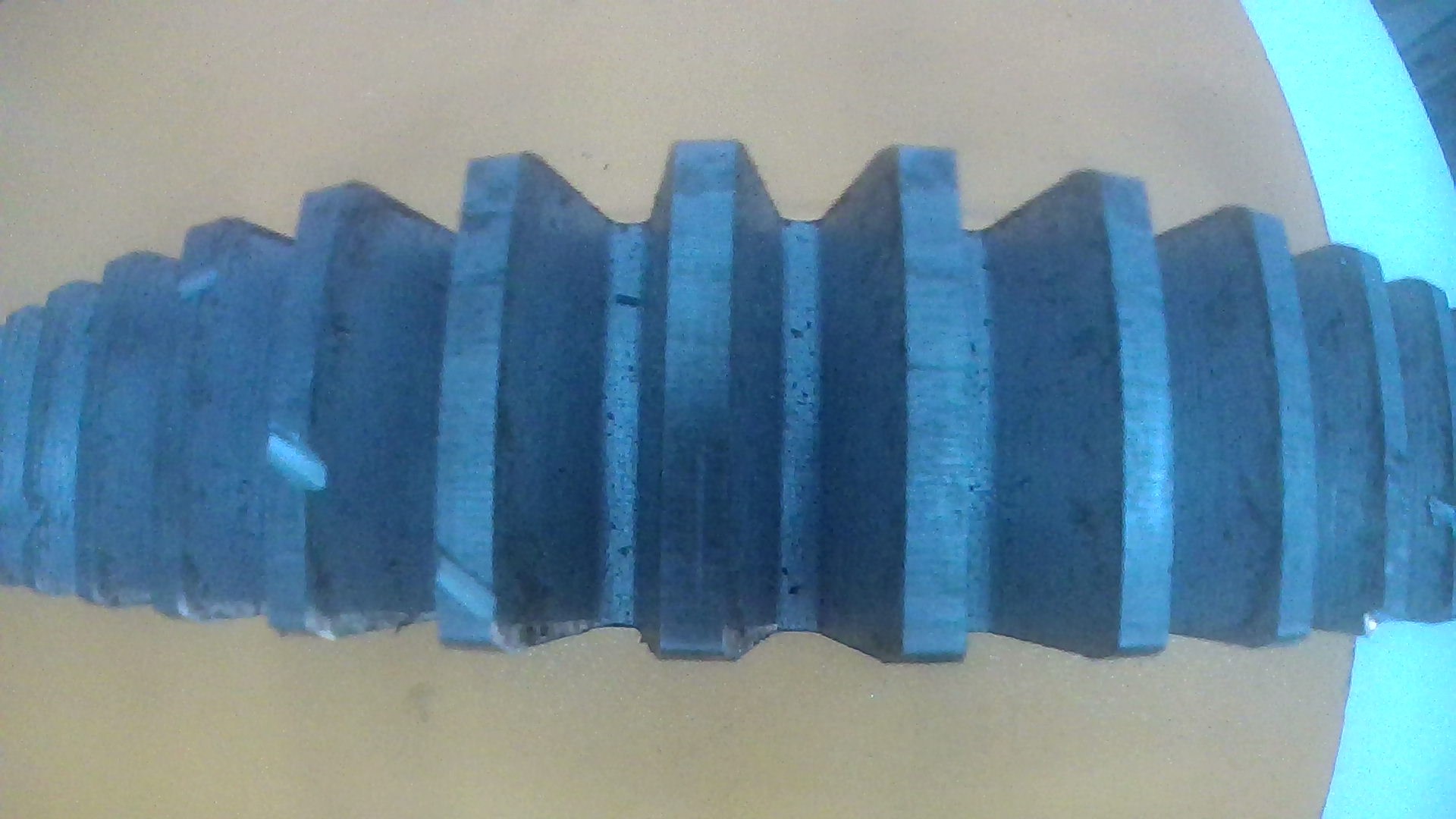

Supplement: S1 Data — (ZIP) [file pone.0322217.s001.zip › dataset/3/WIN_20250111_20_50_22_Pro.jpg]

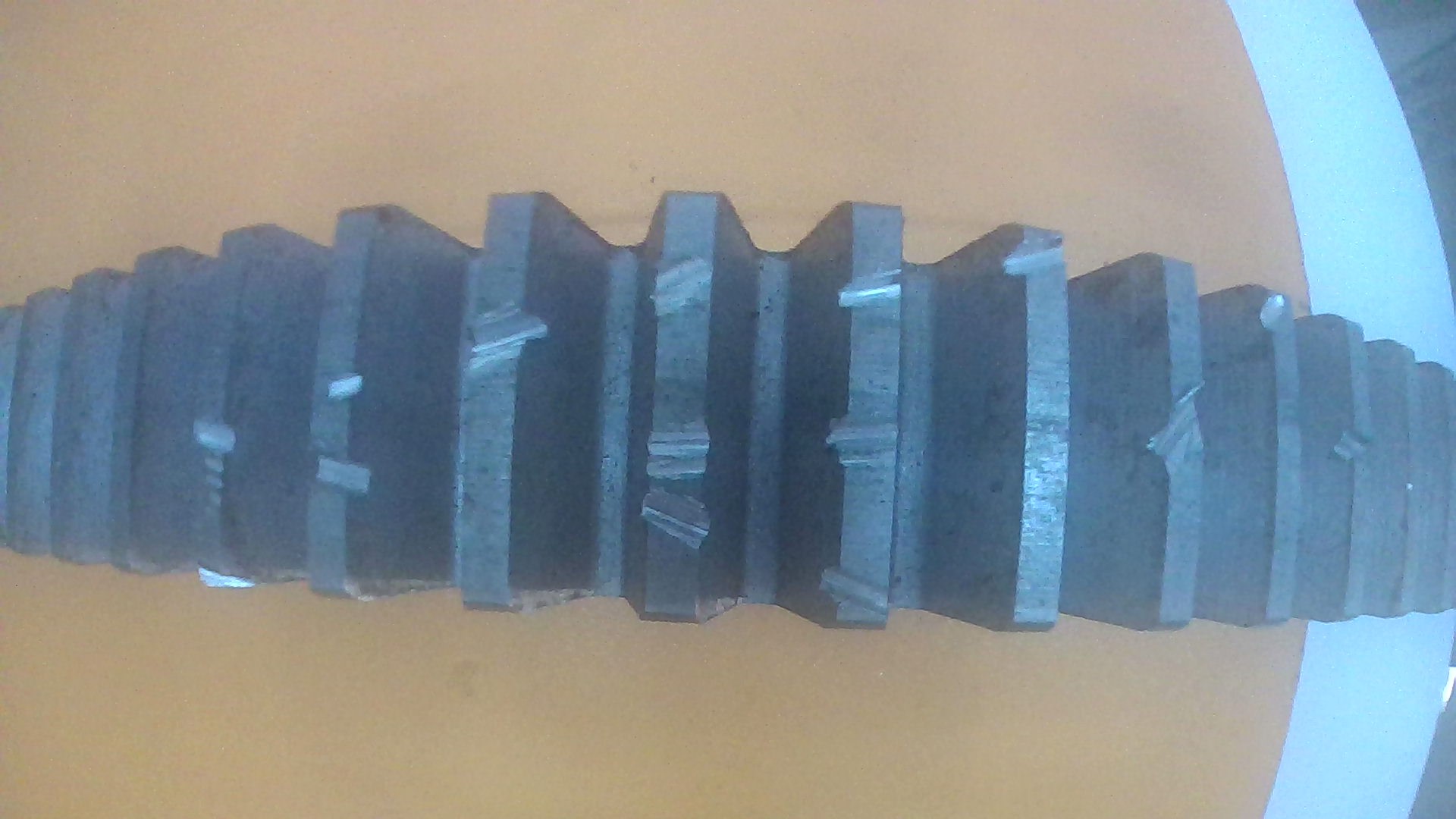

Supplement: S1 Data — (ZIP) [file pone.0322217.s001.zip › dataset/3/WIN_20250111_20_50_33_Pro.jpg]

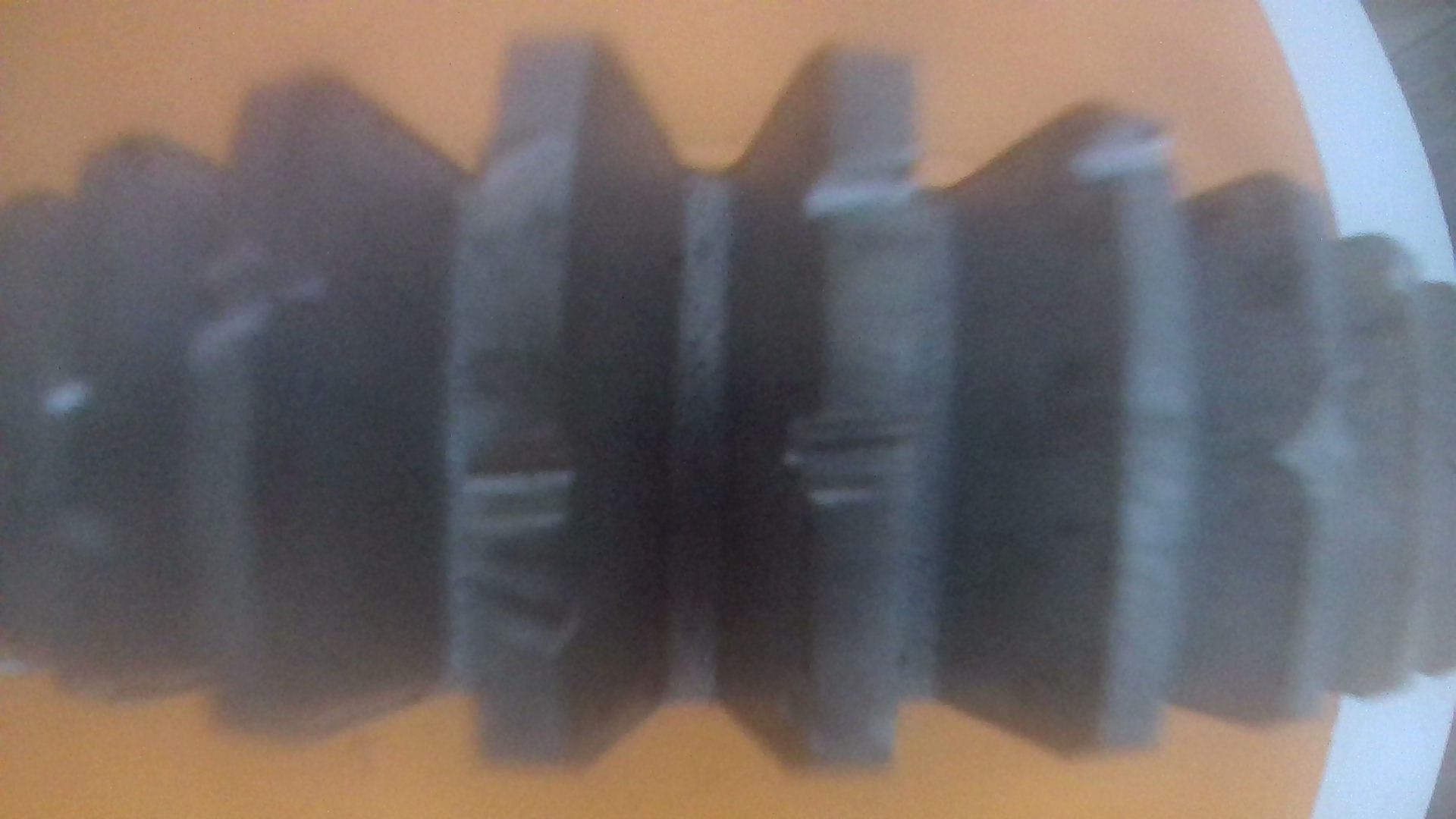

Supplement: S1 Data — (ZIP) [file pone.0322217.s001.zip › dataset/3/WIN_20250111_20_50_40_Pro.jpg]

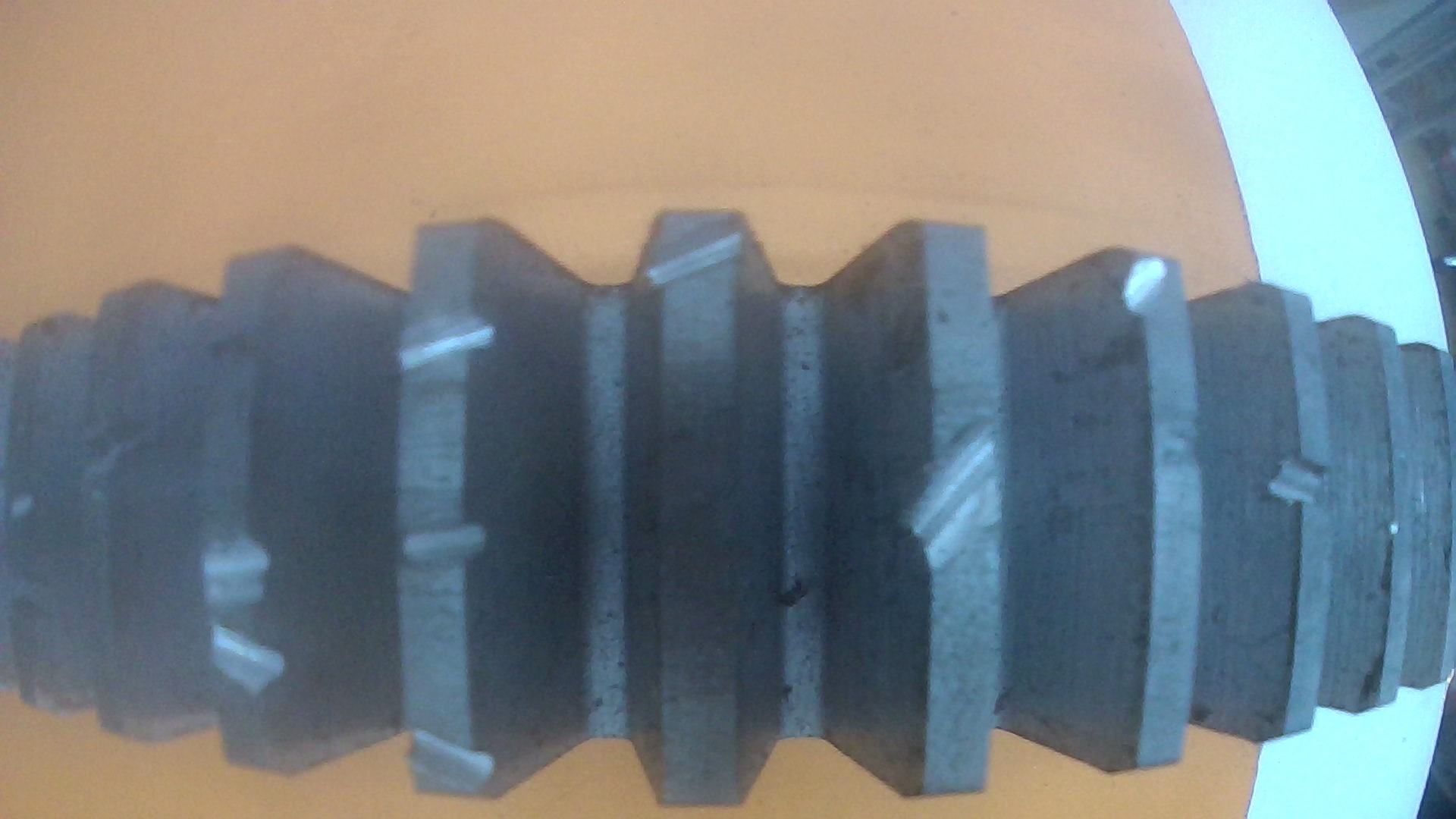

Supplement: S1 Data — (ZIP) [file pone.0322217.s001.zip › dataset/3/WIN_20250111_20_50_41_Pro.jpg]

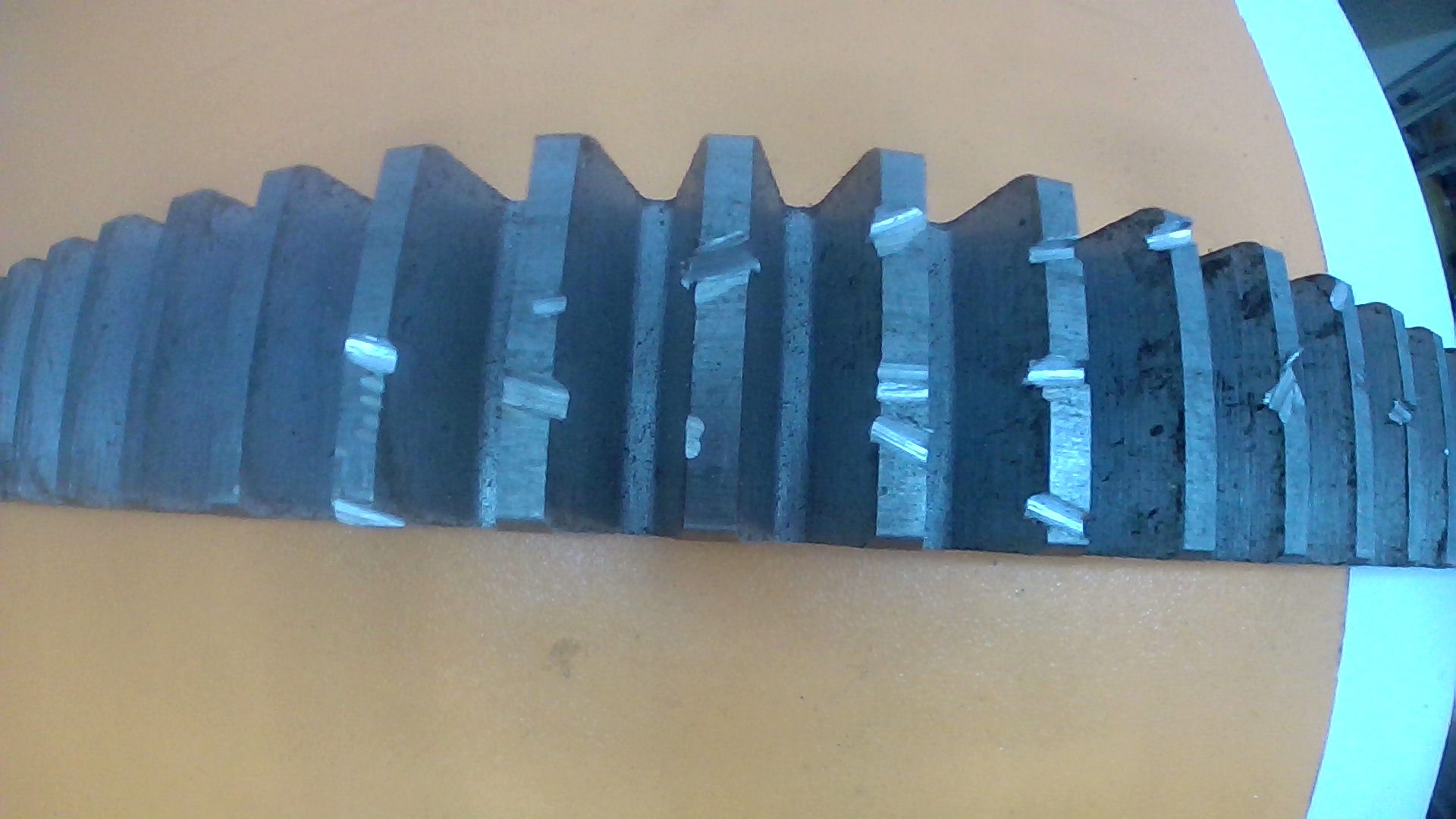

Supplement: S1 Data — (ZIP) [file pone.0322217.s001.zip › dataset/3/WIN_20250111_20_50_43_Pro.jpg]

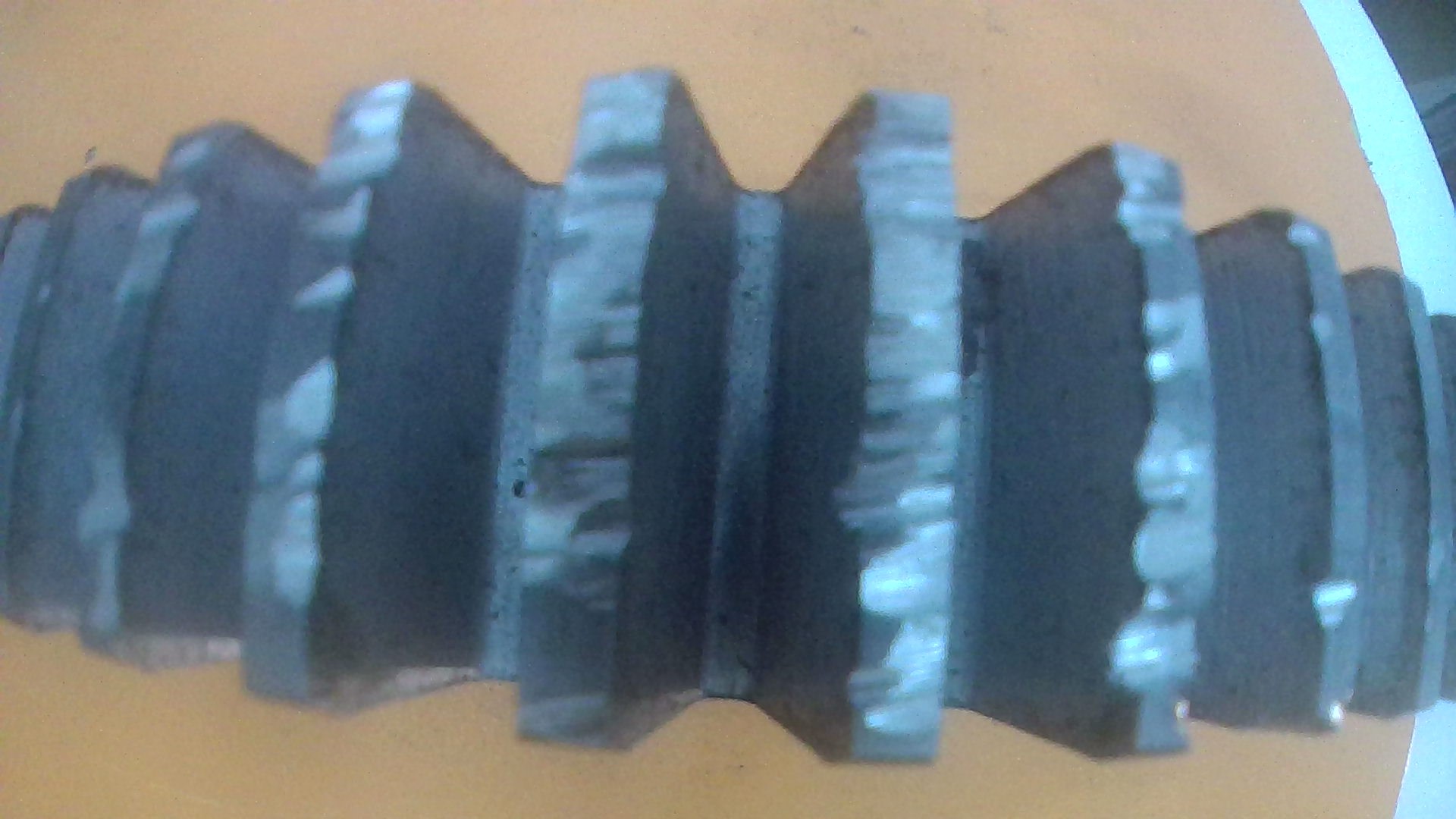

Supplement: S1 Data — (ZIP) [file pone.0322217.s001.zip › dataset/3/WIN_20250111_20_50_57_Pro.jpg]

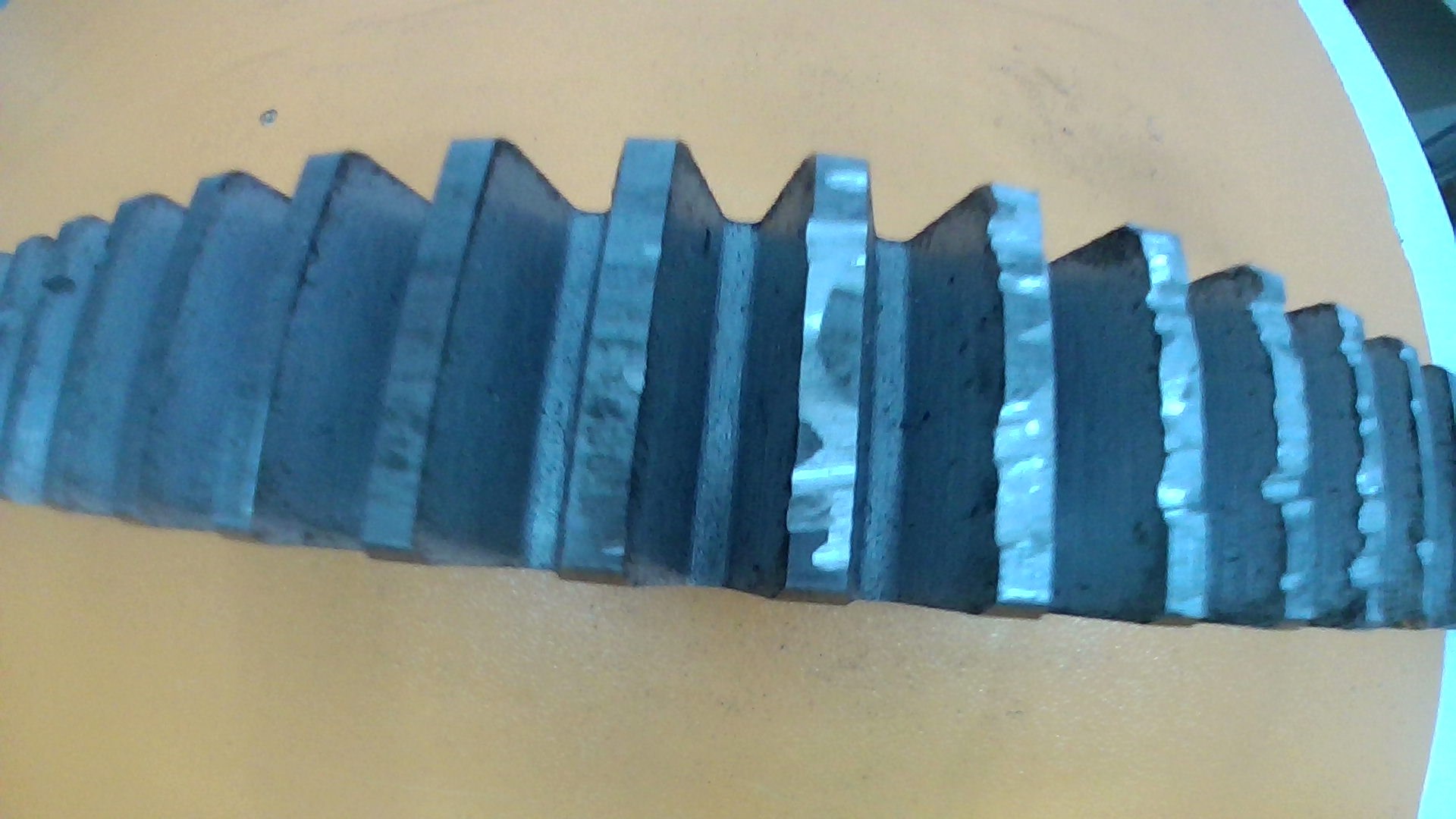

Supplement: S1 Data — (ZIP) [file pone.0322217.s001.zip › dataset/3/WIN_20250111_20_50_59_Pro.jpg]

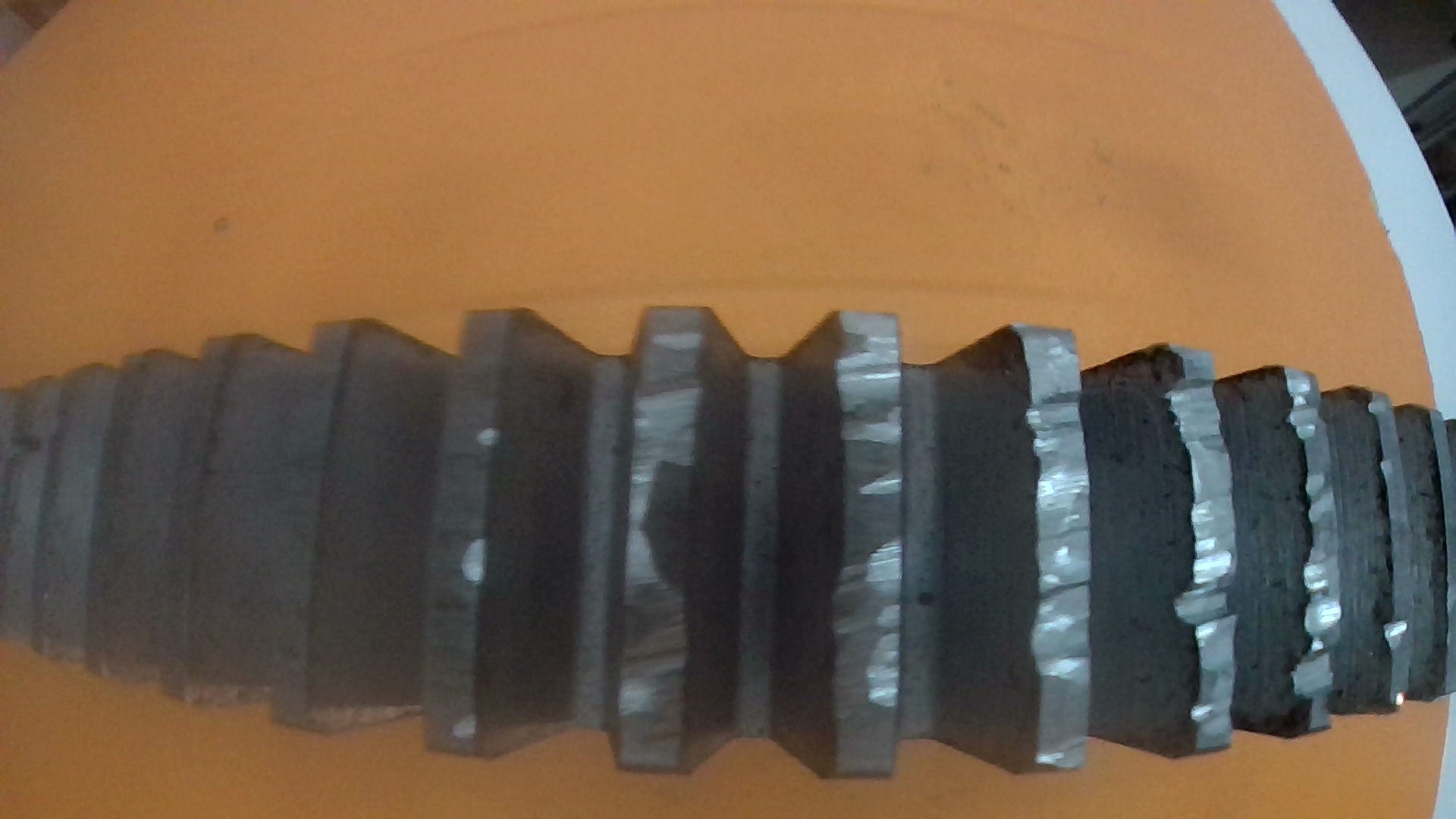

Supplement: S1 Data — (ZIP) [file pone.0322217.s001.zip › dataset/3/WIN_20250111_20_51_07_Pro.jpg]

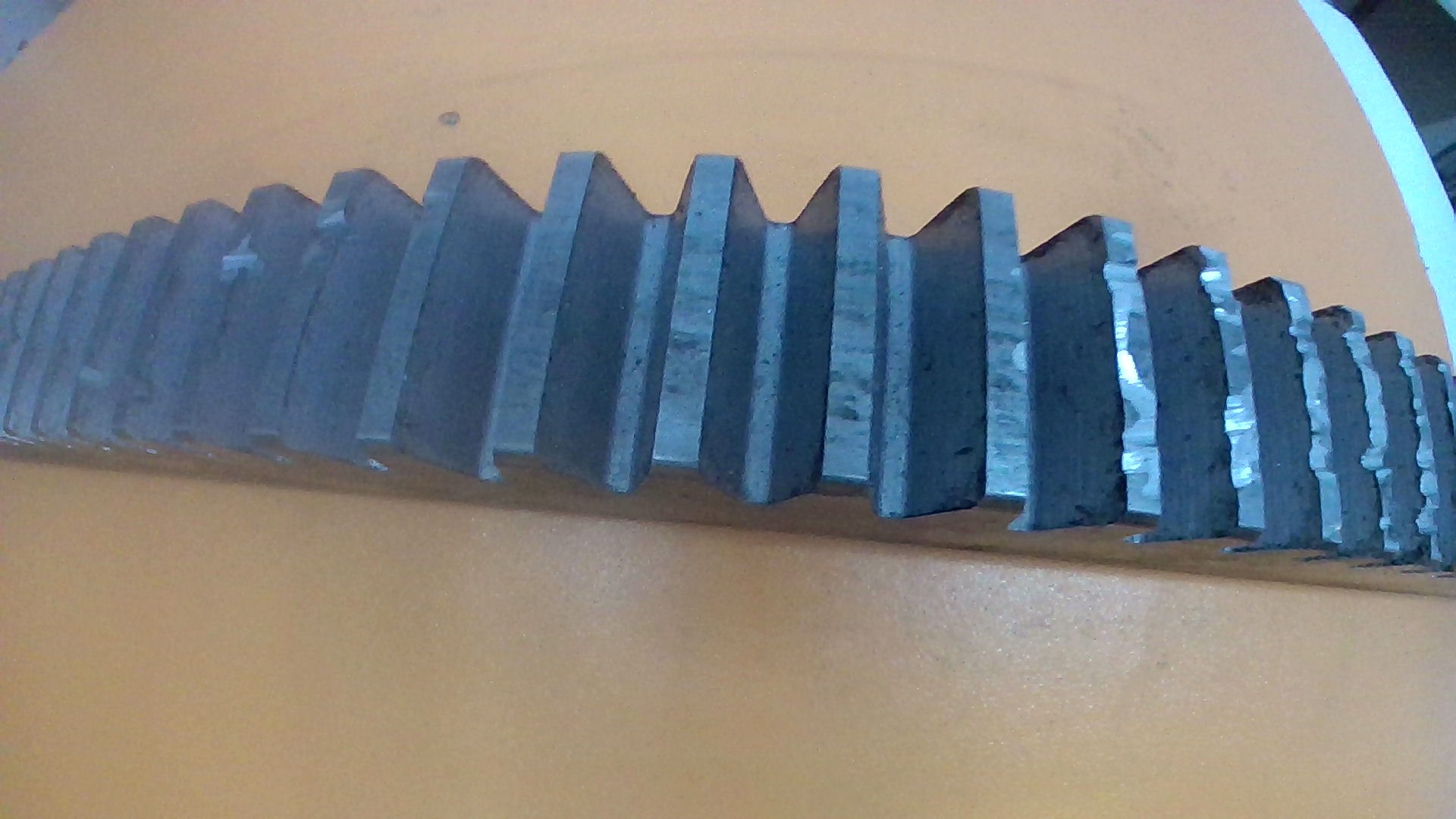

Supplement: S1 Data — (ZIP) [file pone.0322217.s001.zip › dataset/3/WIN_20250111_20_51_08_Pro.jpg]

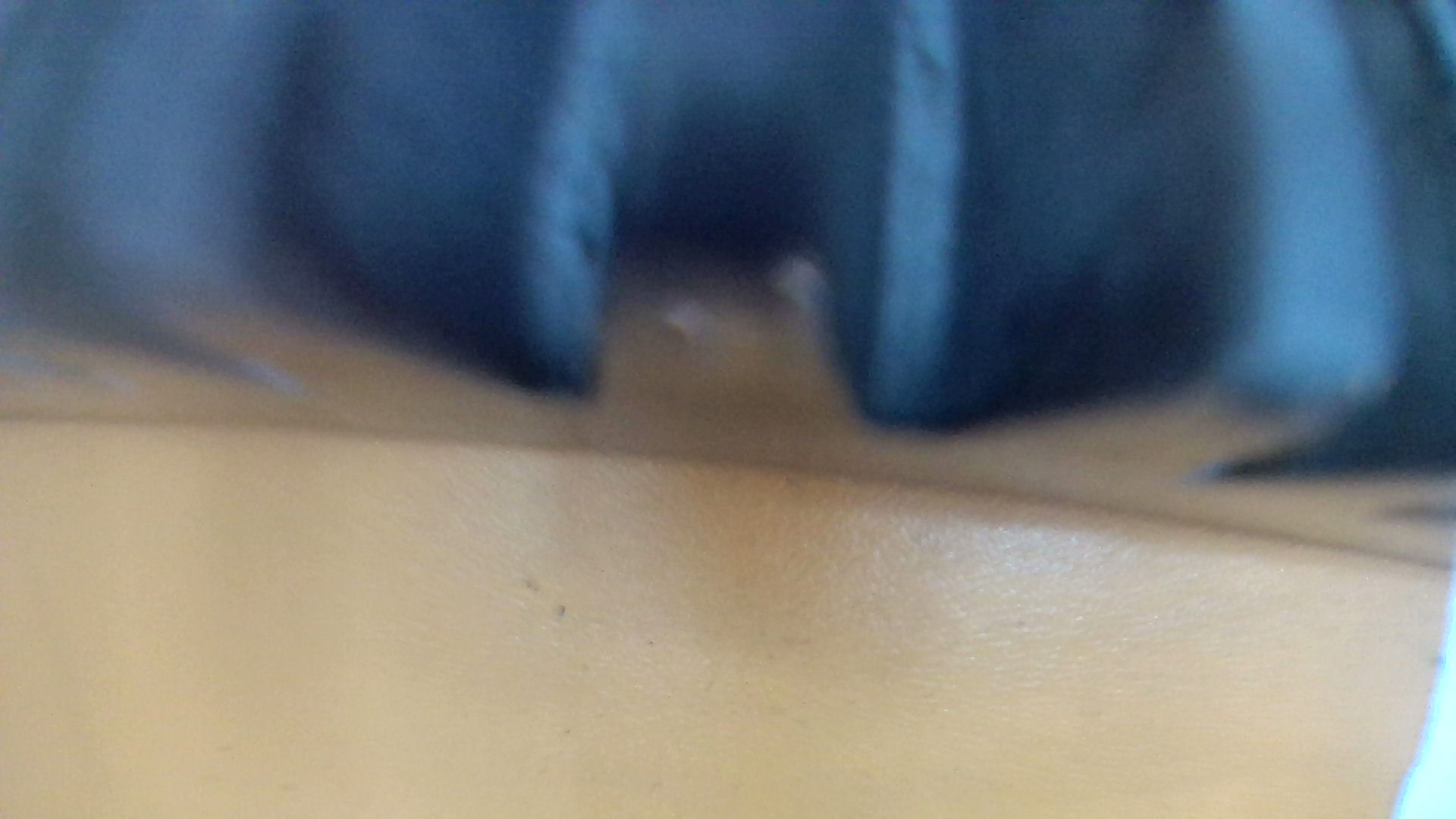

Supplement: S1 Data — (ZIP) [file pone.0322217.s001.zip › dataset/3/WIN_20250111_20_53_11_Pro.jpg]

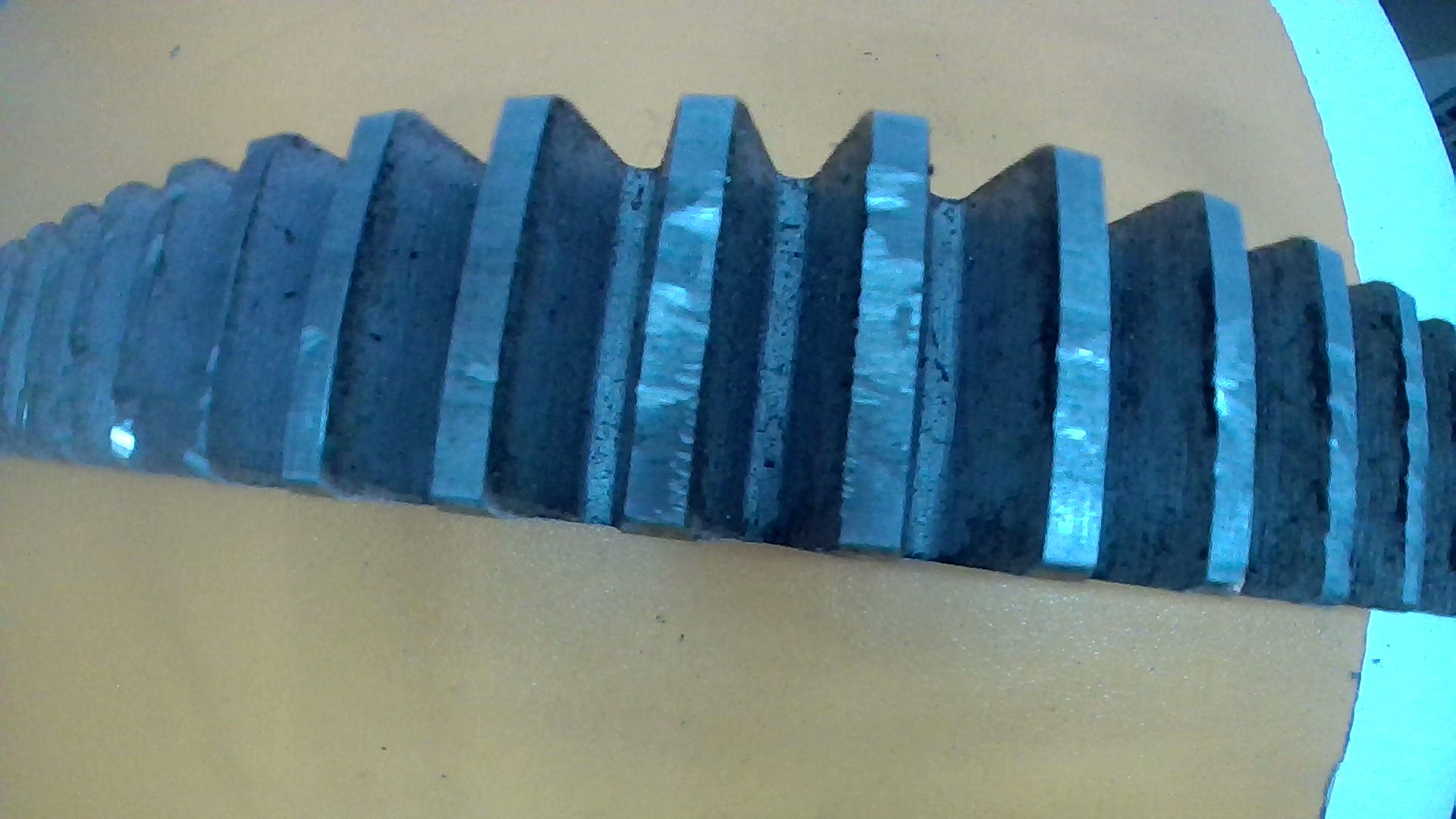

Supplement: S1 Data — (ZIP) [file pone.0322217.s001.zip › dataset/3/WIN_20250111_20_53_12_Pro.jpg]

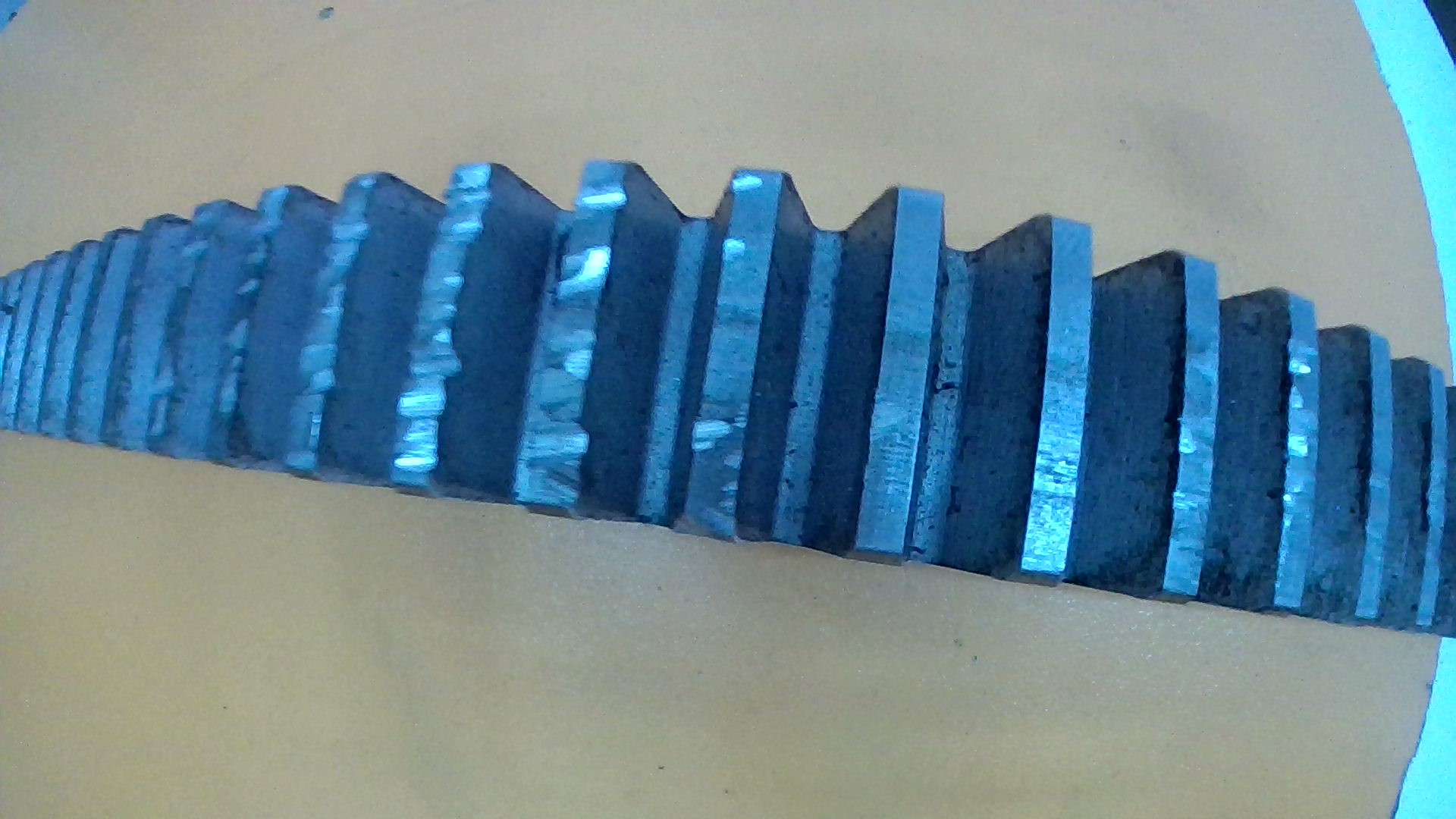

Supplement: S1 Data — (ZIP) [file pone.0322217.s001.zip › dataset/3/WIN_20250111_20_53_15_Pro.jpg]

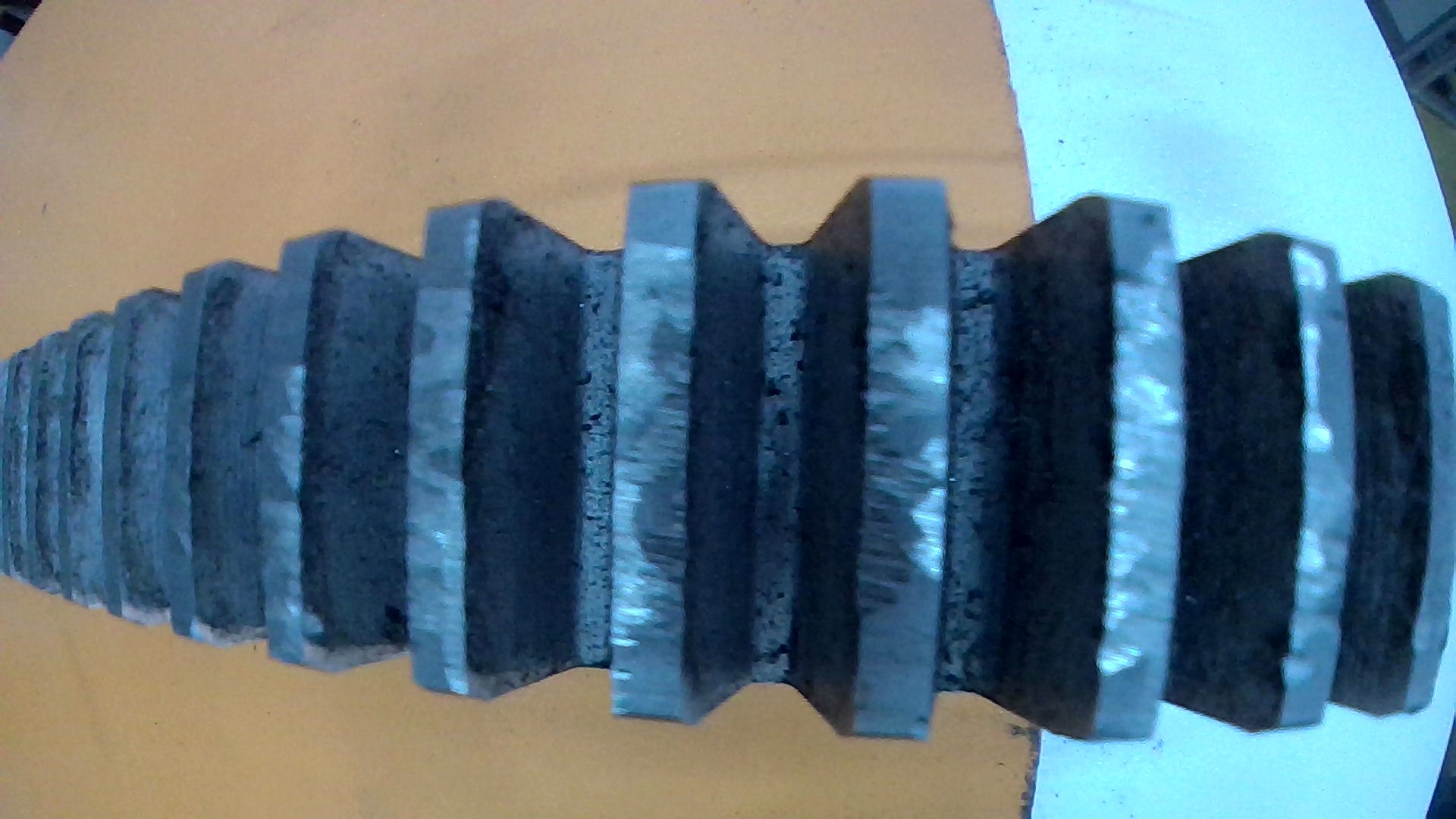

Supplement: S1 Data — (ZIP) [file pone.0322217.s001.zip › dataset/3/WIN_20250111_20_53_17_Pro.jpg]

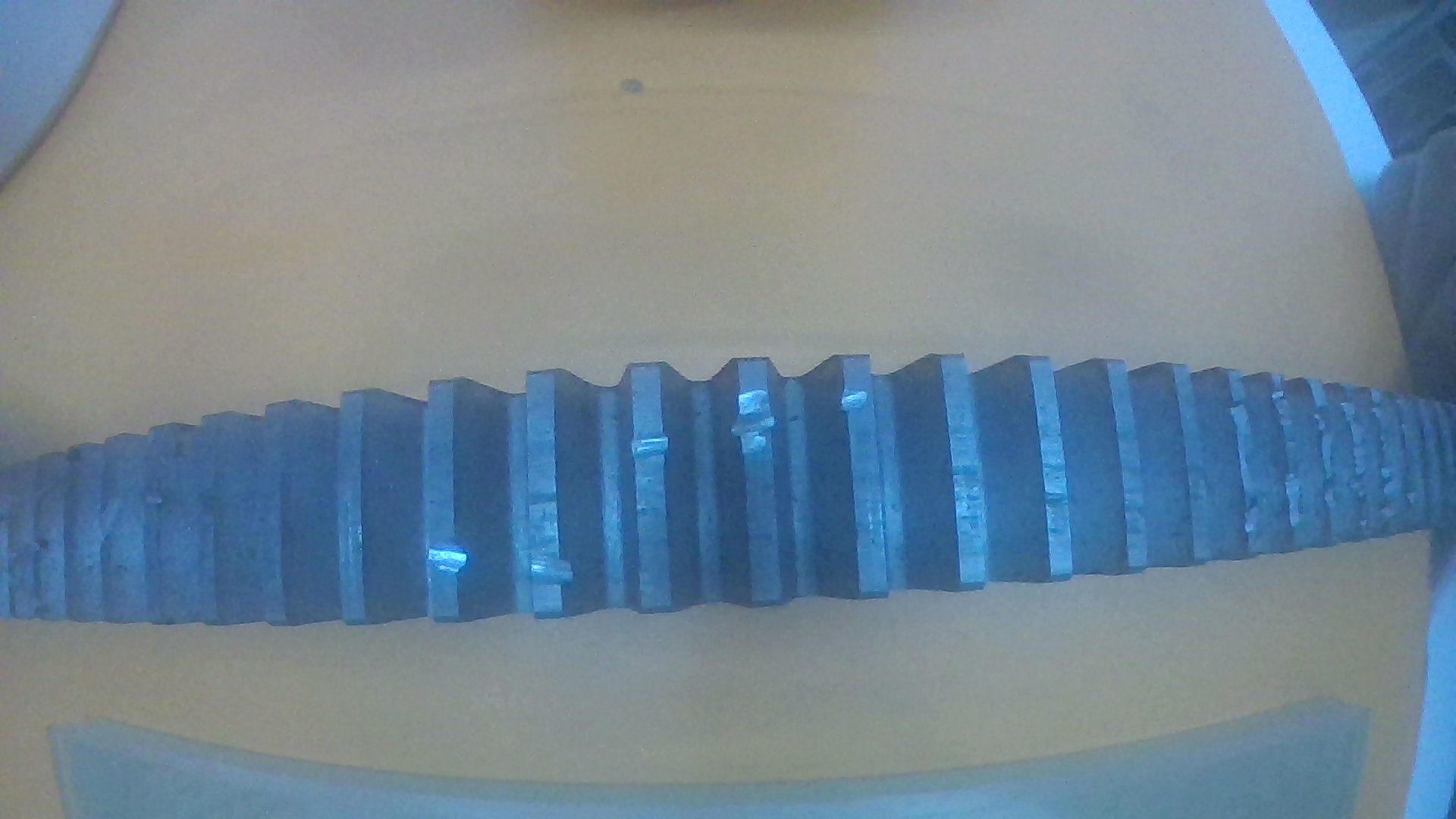

Supplement: S1 Data — (ZIP) [file pone.0322217.s001.zip › dataset/4/WIN_20250111_23_29_20_Pro.jpg]

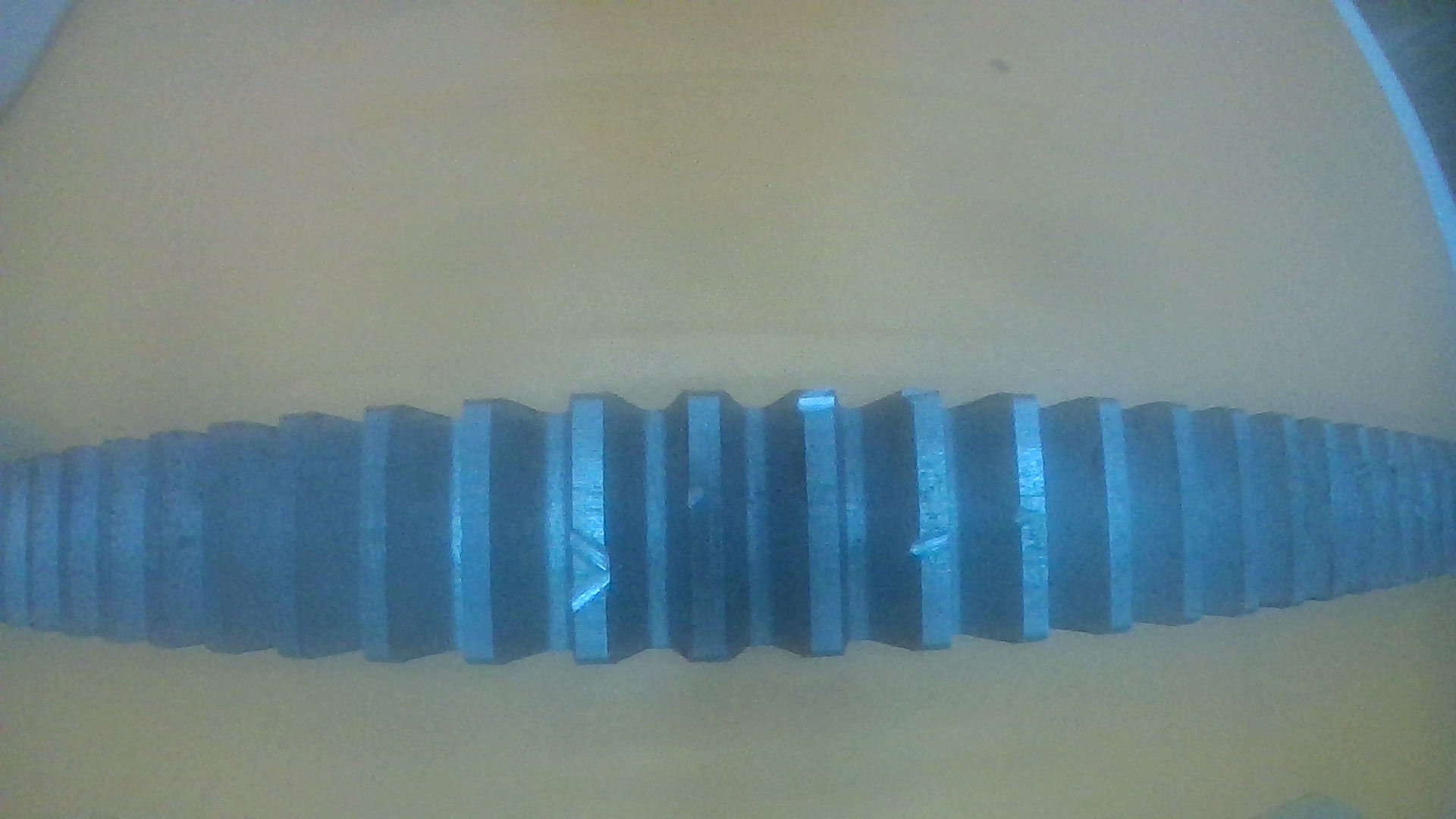

Supplement: S1 Data — (ZIP) [file pone.0322217.s001.zip › dataset/4/WIN_20250111_23_30_18_Pro.jpg]

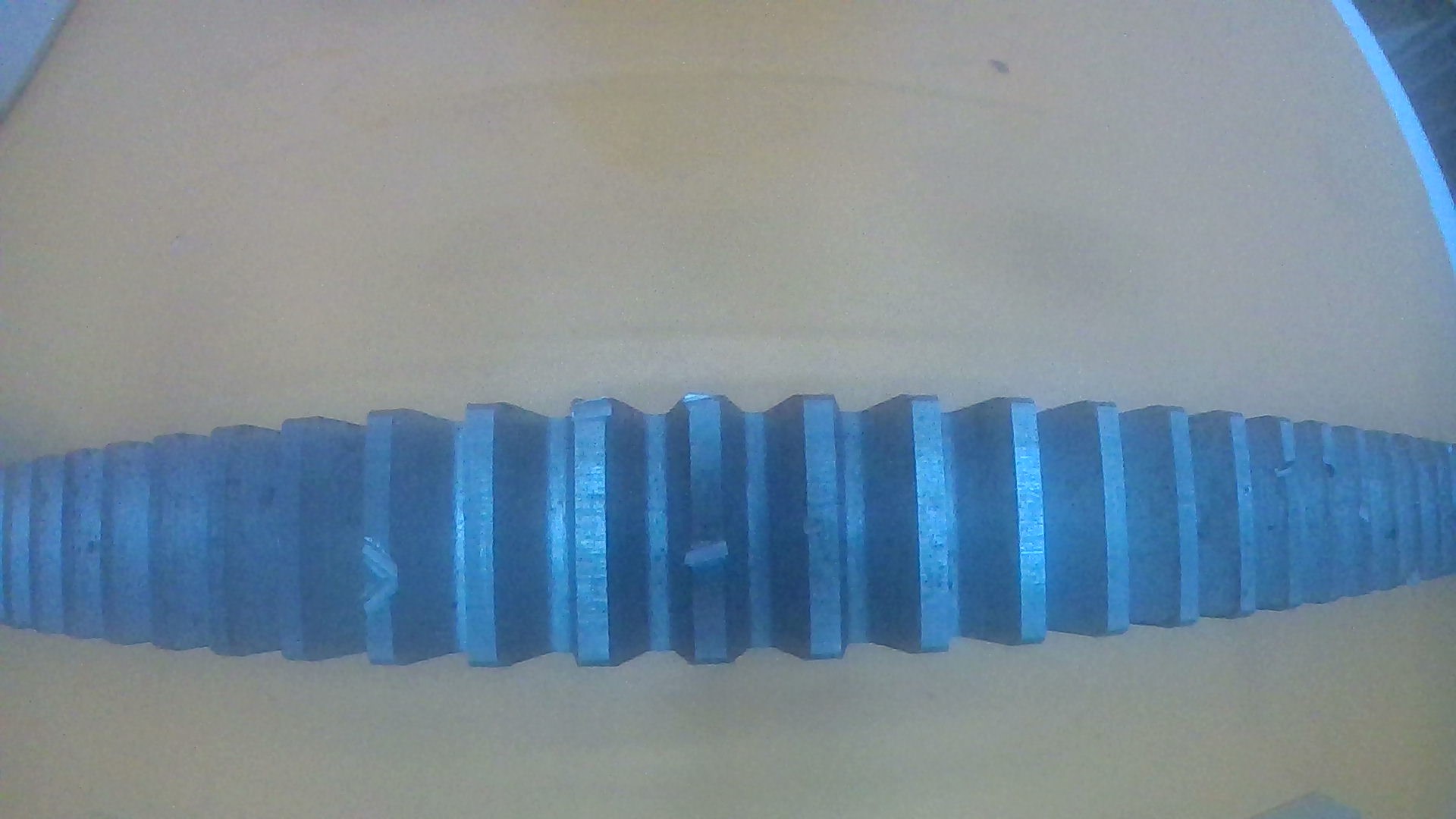

Supplement: S1 Data — (ZIP) [file pone.0322217.s001.zip › dataset/4/WIN_20250111_23_30_21_Pro.jpg]

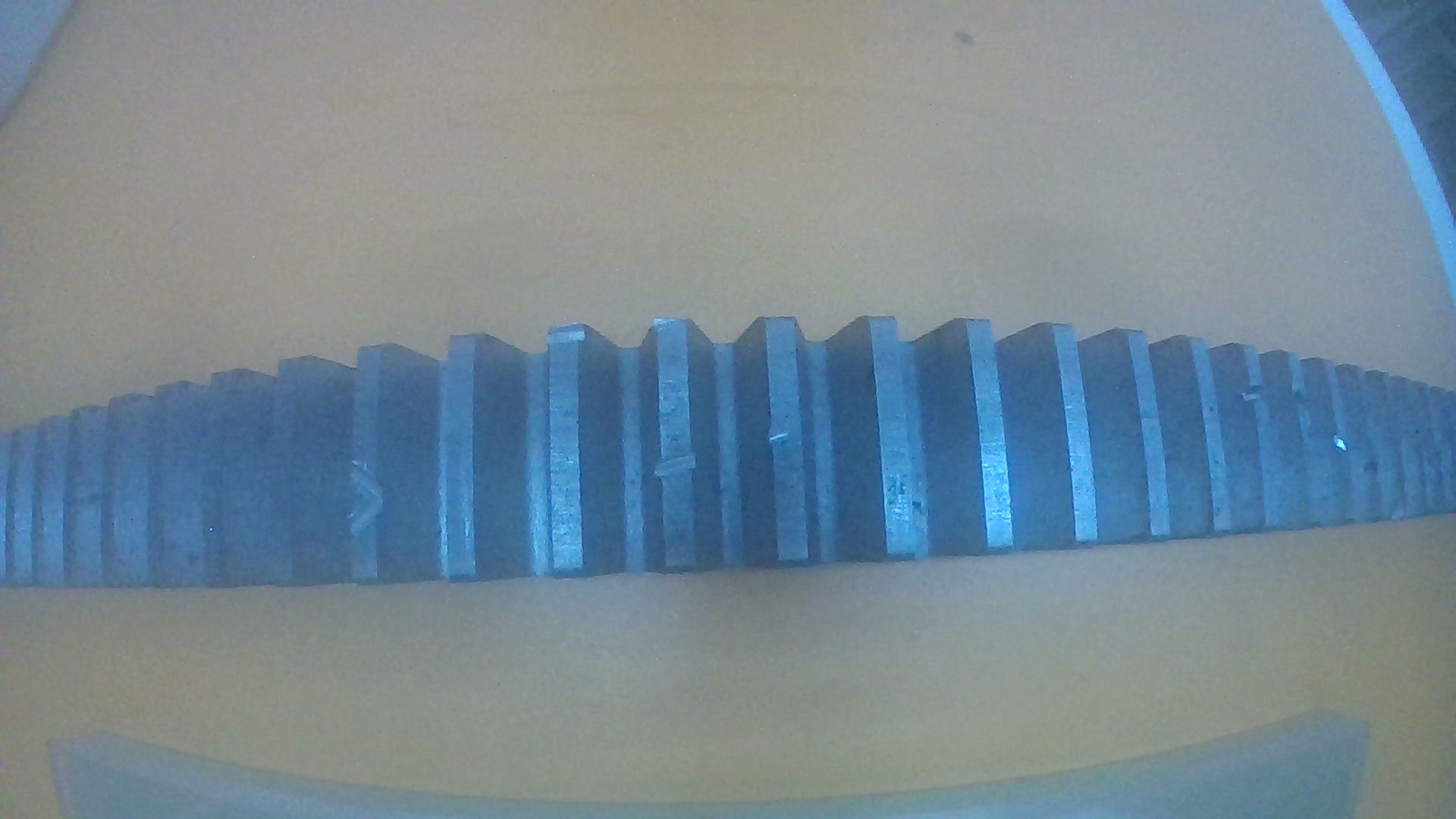

Supplement: S1 Data — (ZIP) [file pone.0322217.s001.zip › dataset/4/WIN_20250111_23_30_41_Pro.jpg]

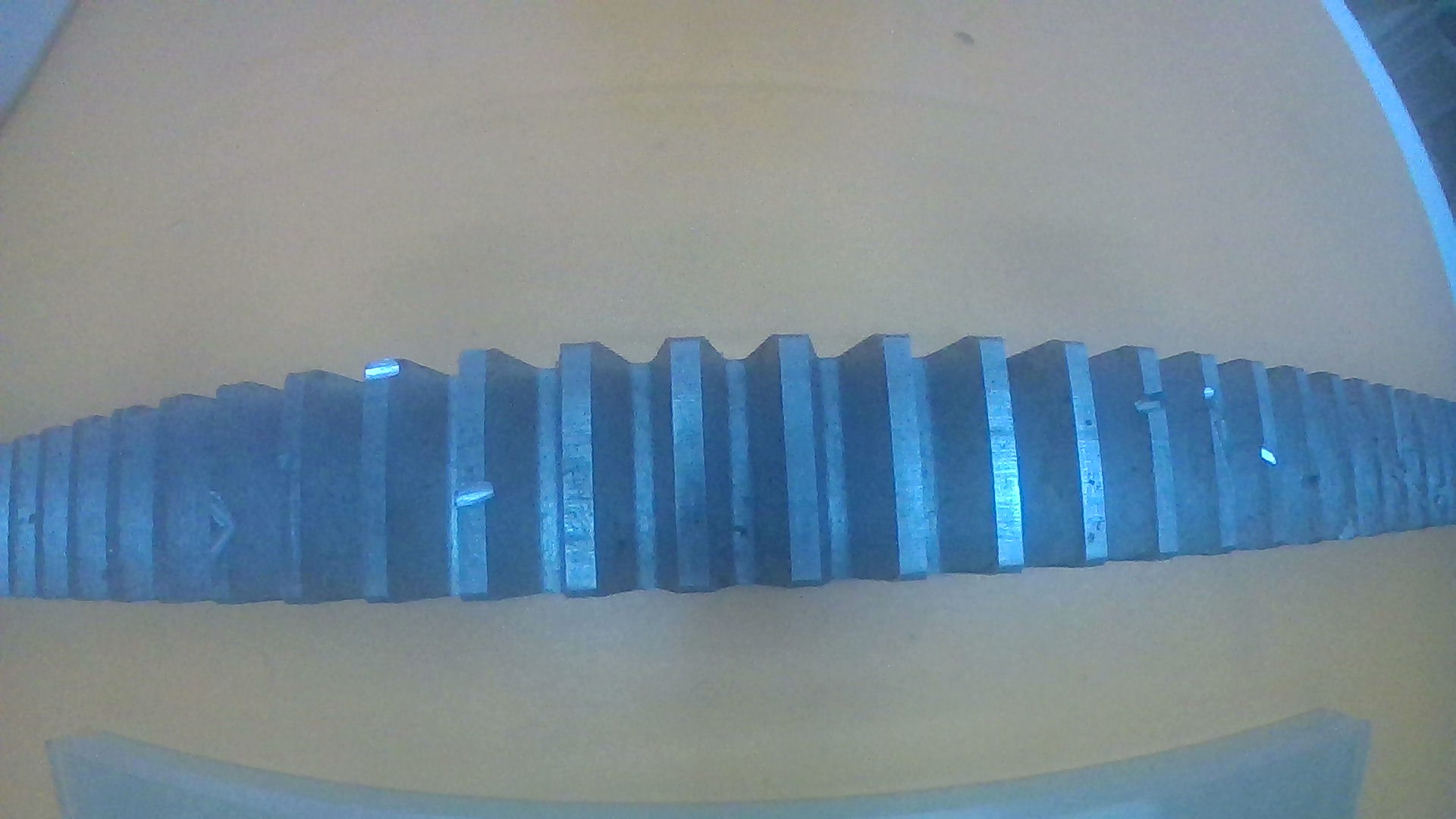

Supplement: S1 Data — (ZIP) [file pone.0322217.s001.zip › dataset/4/WIN_20250111_23_30_43_Pro.jpg]

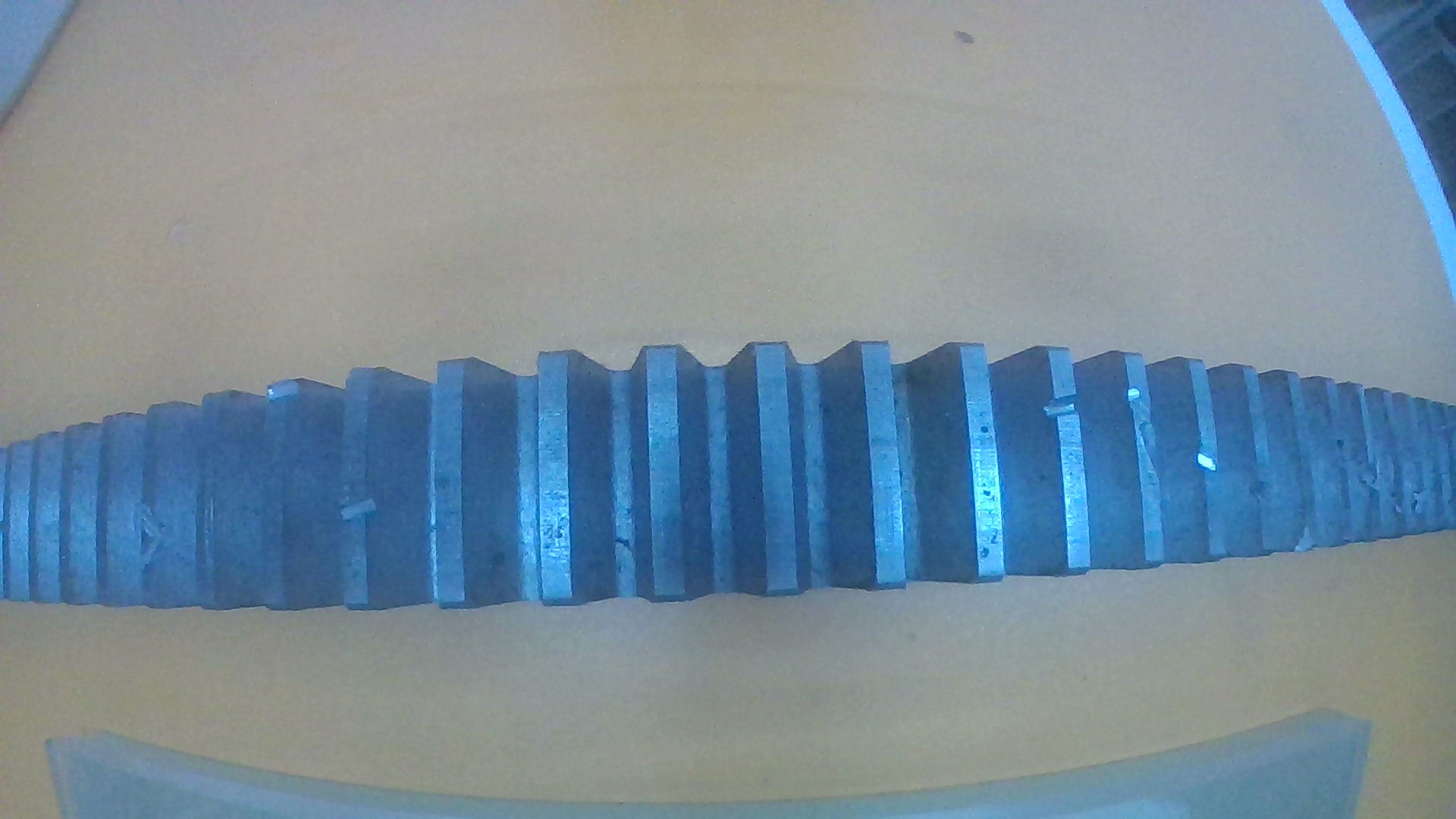

Supplement: S1 Data — (ZIP) [file pone.0322217.s001.zip › dataset/4/WIN_20250111_23_30_44_Pro.jpg]

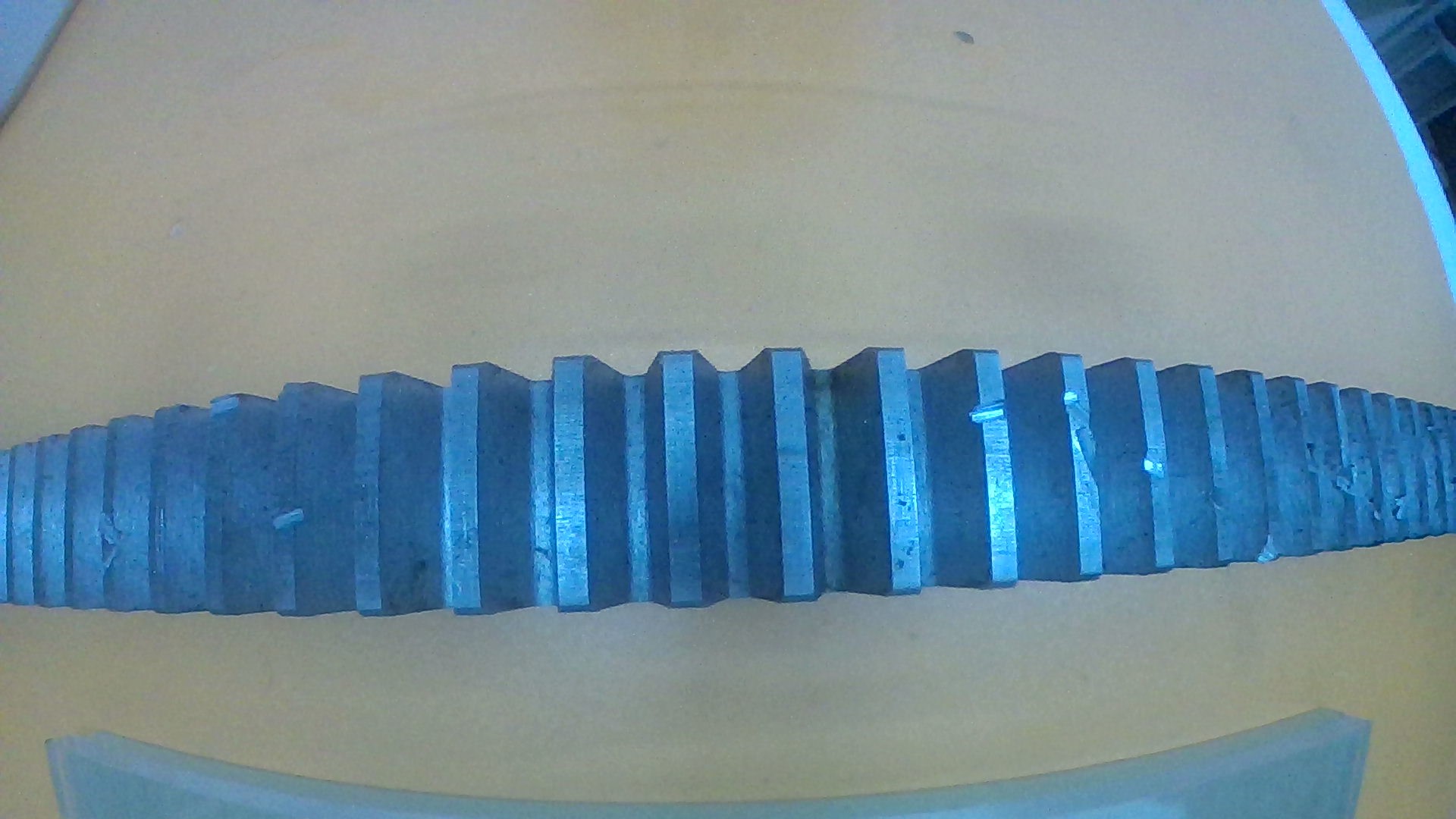

Supplement: S1 Data — (ZIP) [file pone.0322217.s001.zip › dataset/4/WIN_20250111_23_30_45_Pro.jpg]

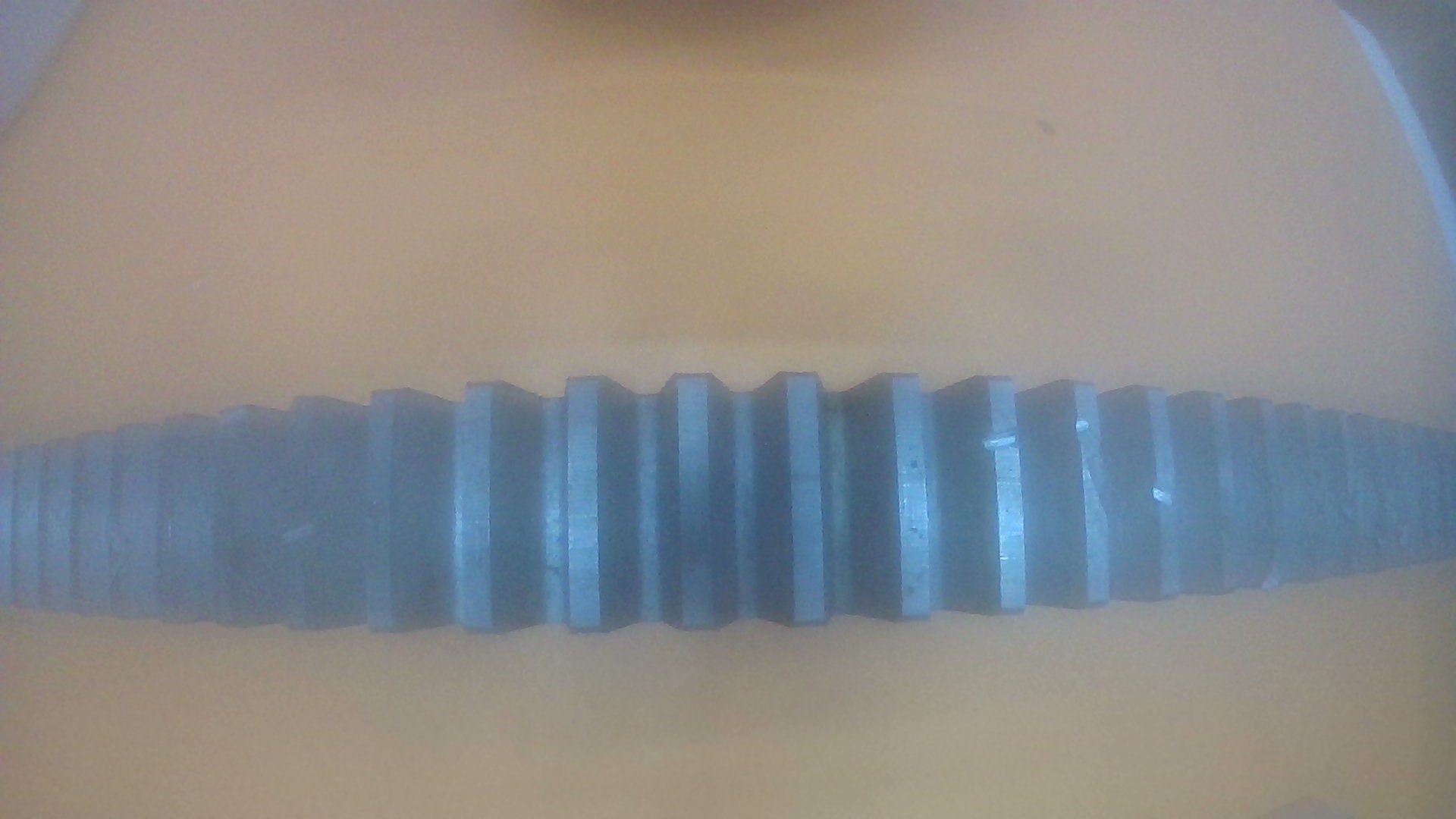

Supplement: S1 Data — (ZIP) [file pone.0322217.s001.zip › dataset/4/WIN_20250111_23_31_56_Pro.jpg]

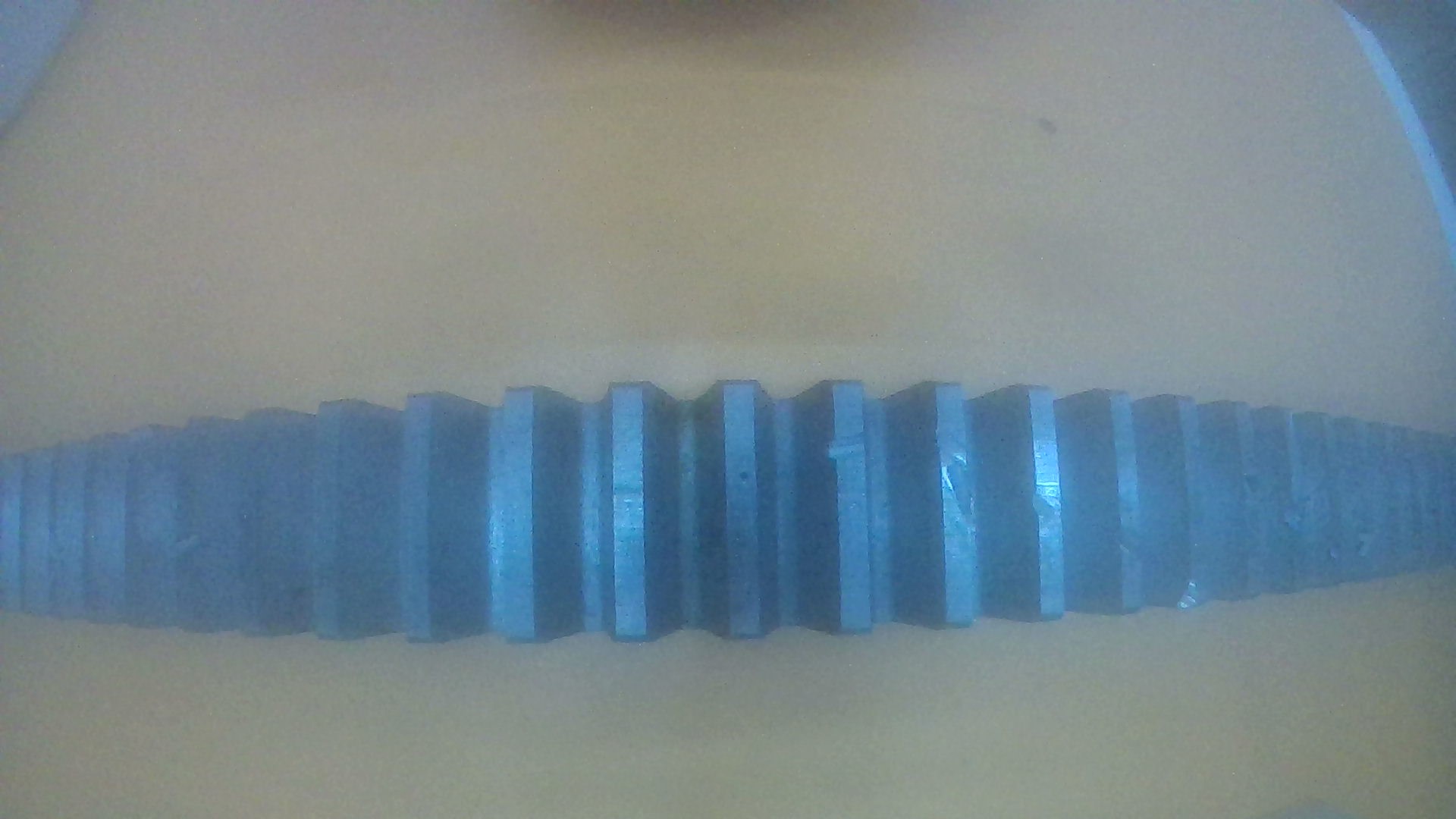

Supplement: S1 Data — (ZIP) [file pone.0322217.s001.zip › dataset/4/WIN_20250111_23_31_57_Pro.jpg]

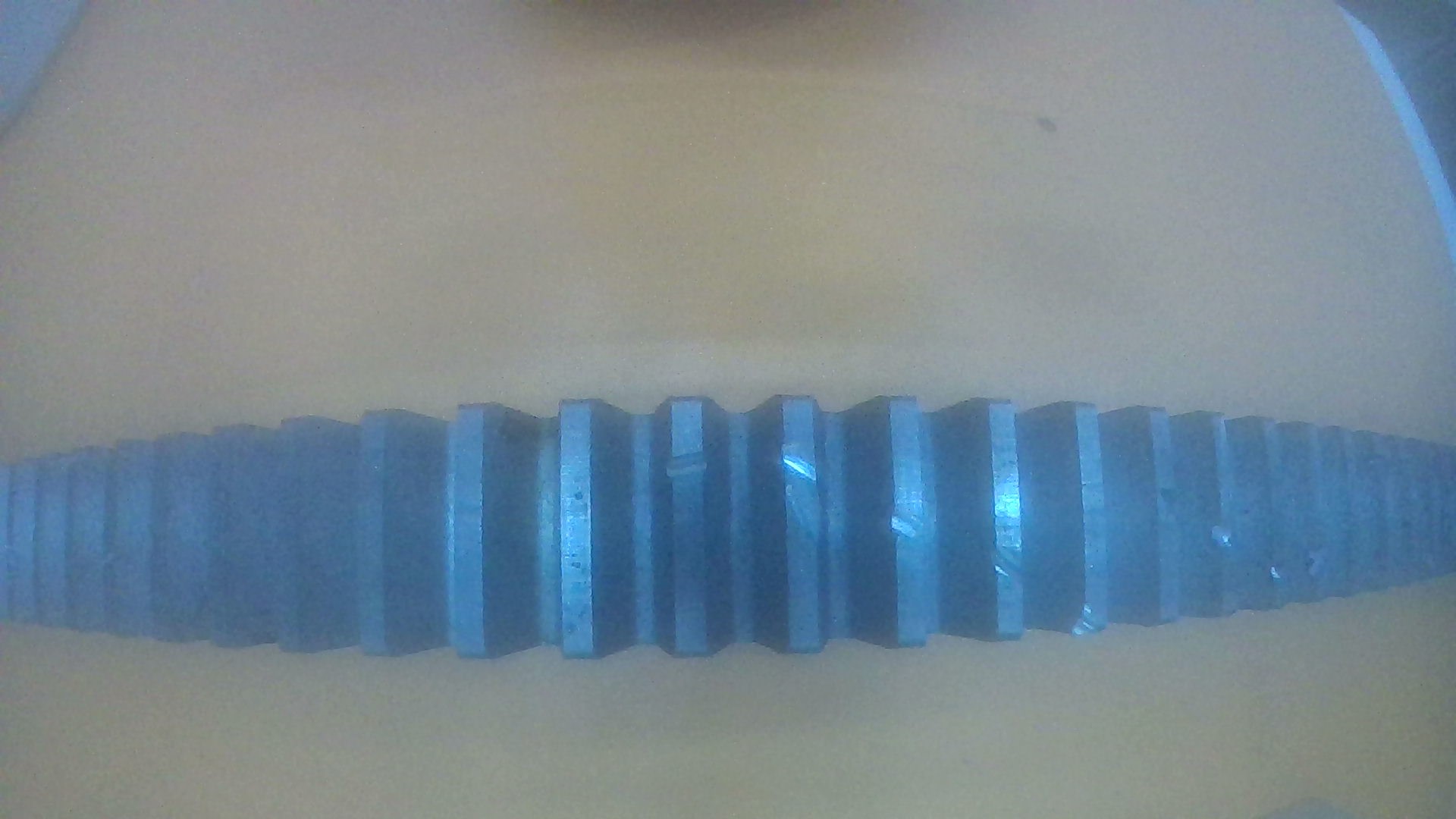

Supplement: S1 Data — (ZIP) [file pone.0322217.s001.zip › dataset/4/WIN_20250111_23_31_59_Pro.jpg]

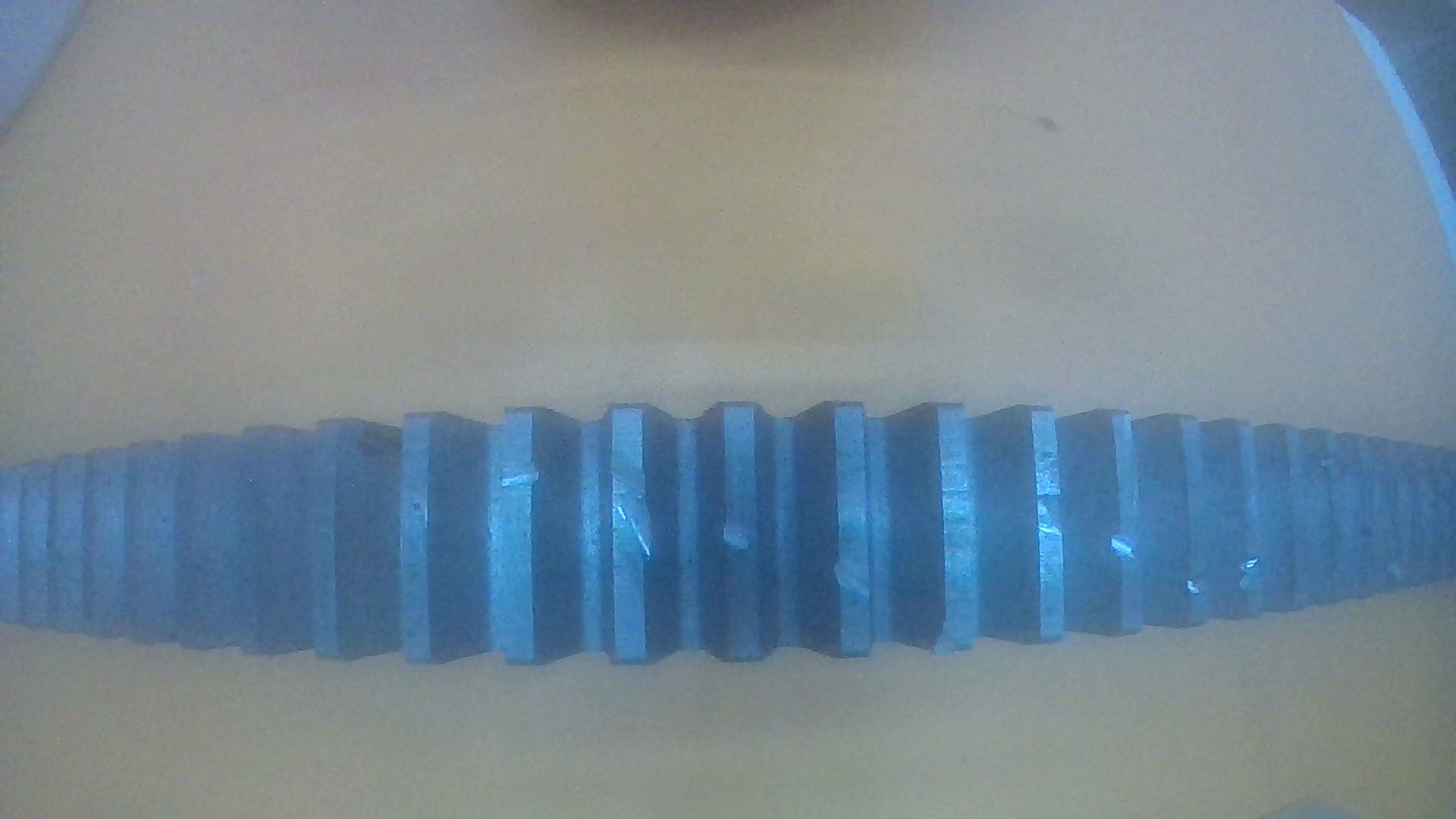

Supplement: S1 Data — (ZIP) [file pone.0322217.s001.zip › dataset/4/WIN_20250111_23_32_00_Pro.jpg]

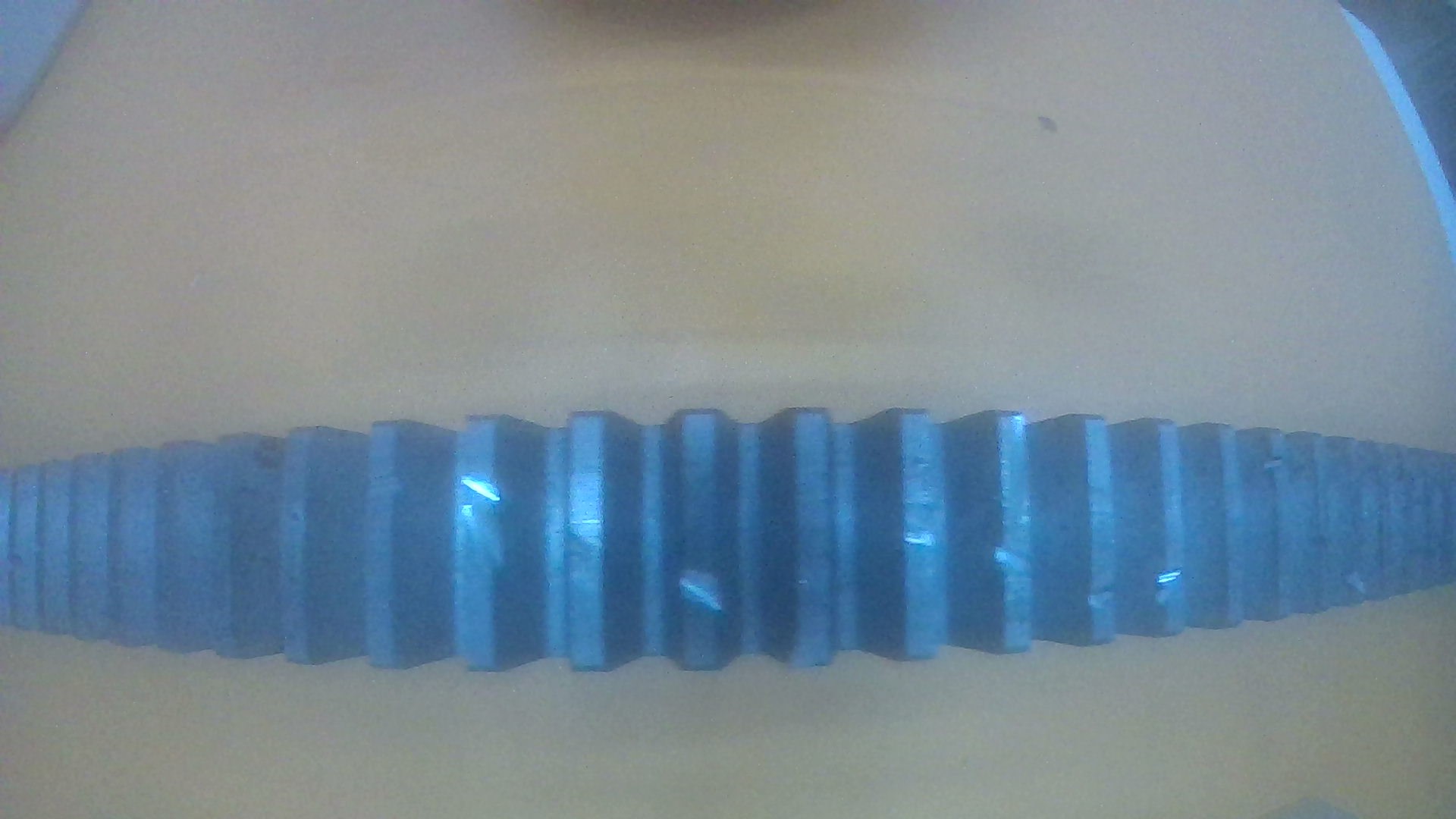

Supplement: S1 Data — (ZIP) [file pone.0322217.s001.zip › dataset/4/WIN_20250111_23_32_01_Pro.jpg]

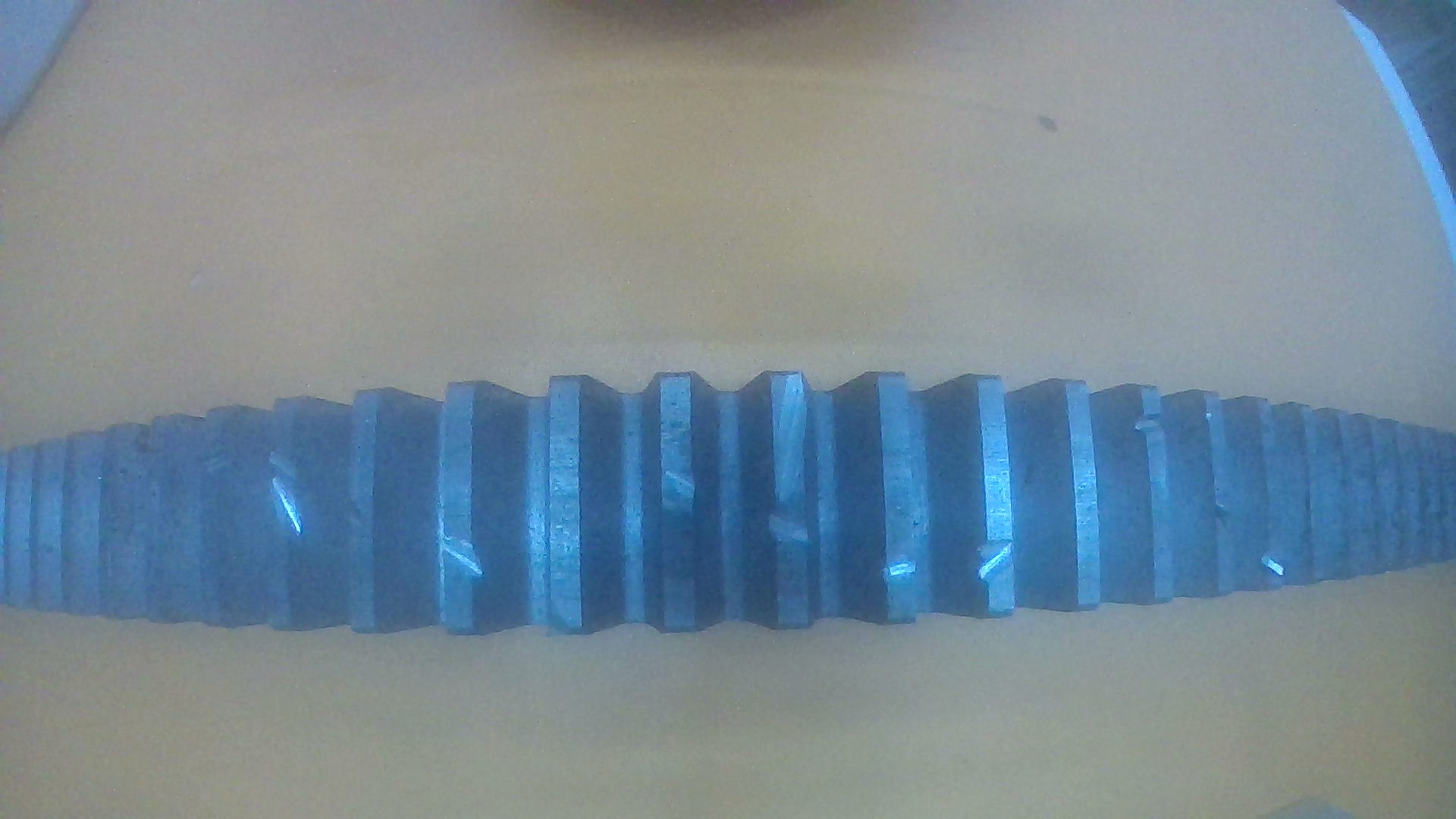

Supplement: S1 Data — (ZIP) [file pone.0322217.s001.zip › dataset/4/WIN_20250111_23_32_02_Pro.jpg]

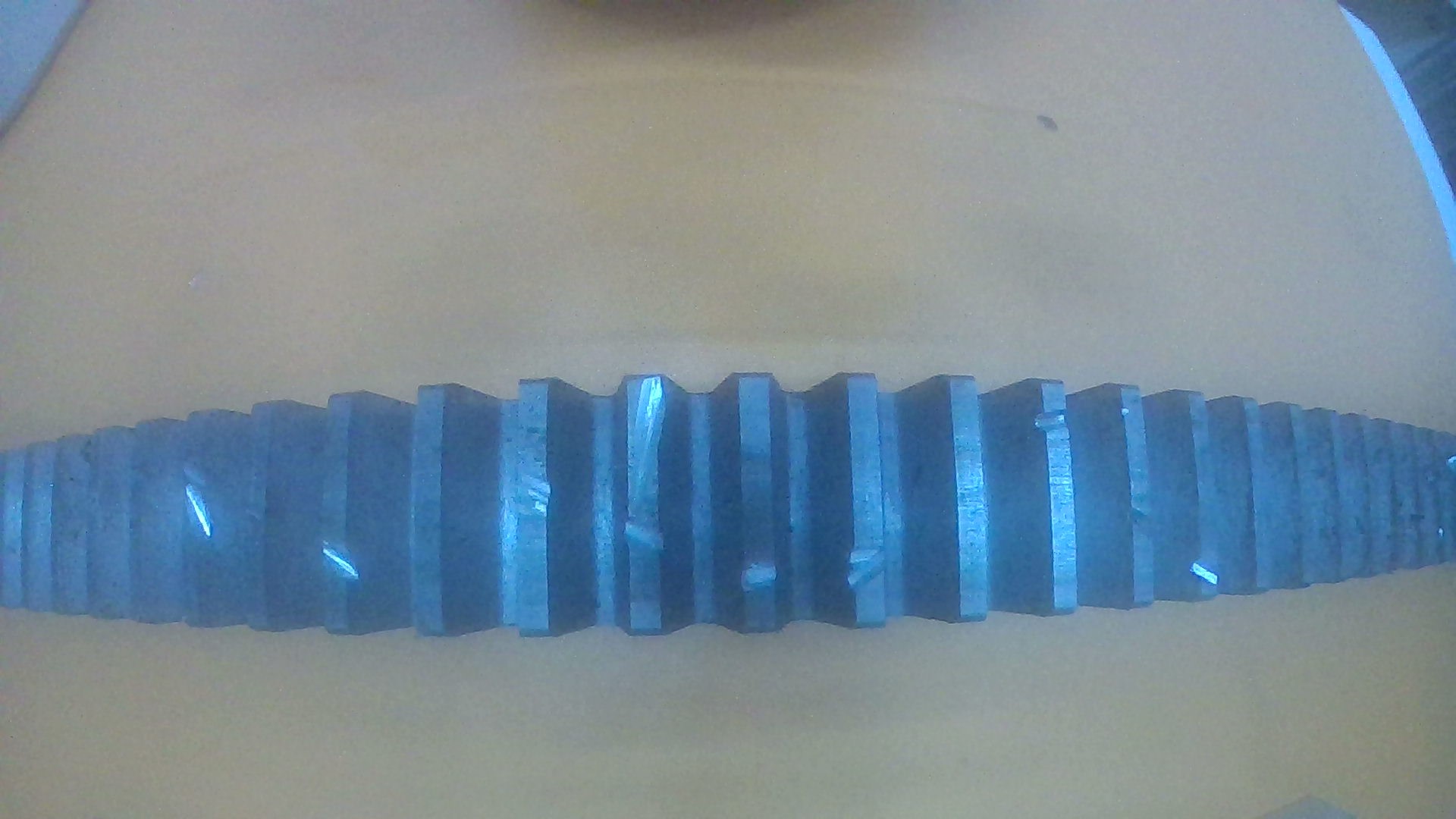

Supplement: S1 Data — (ZIP) [file pone.0322217.s001.zip › dataset/4/WIN_20250111_23_32_04_Pro.jpg]

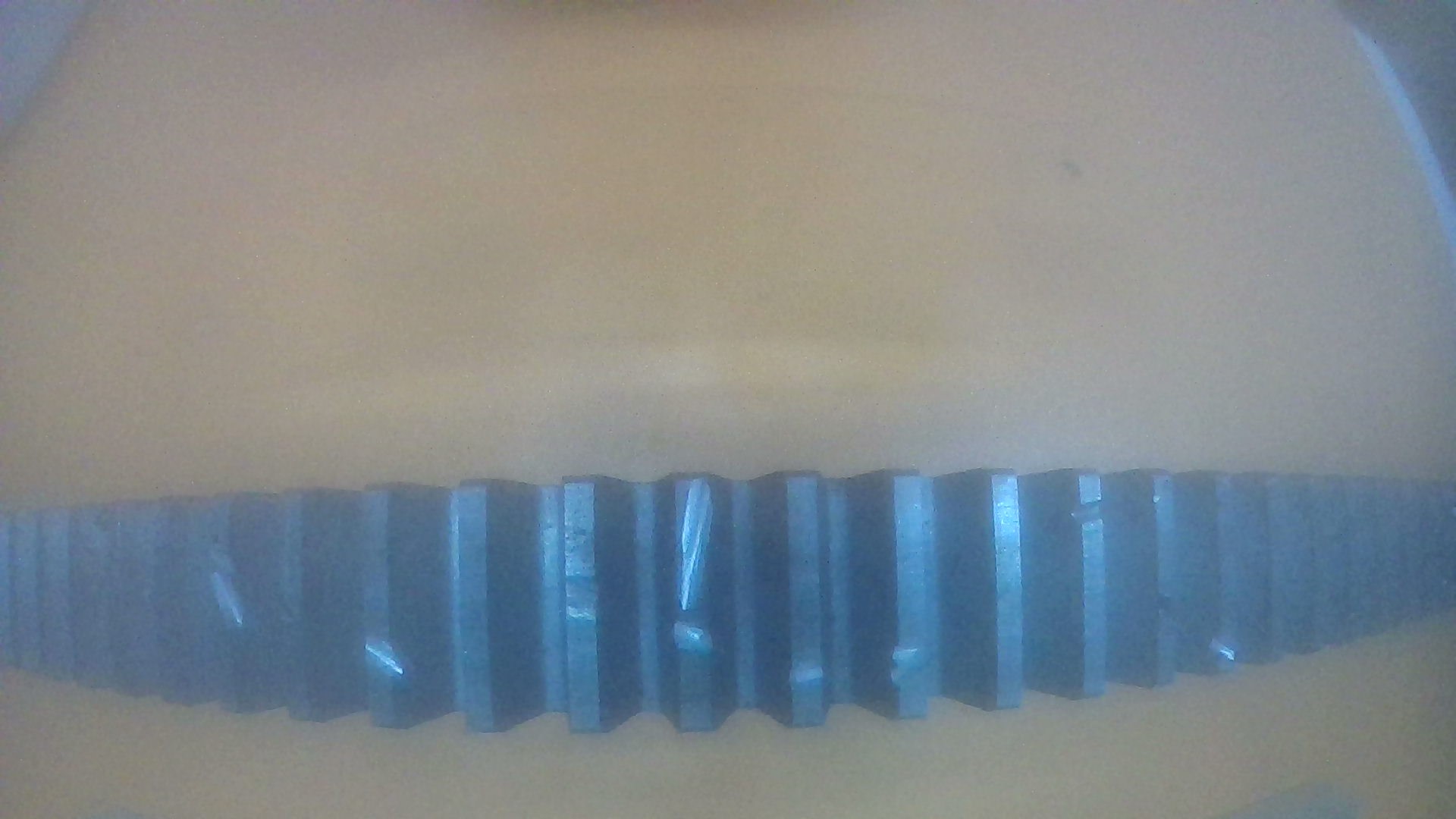

Supplement: S1 Data — (ZIP) [file pone.0322217.s001.zip › dataset/4/WIN_20250111_23_32_32_Pro.jpg]

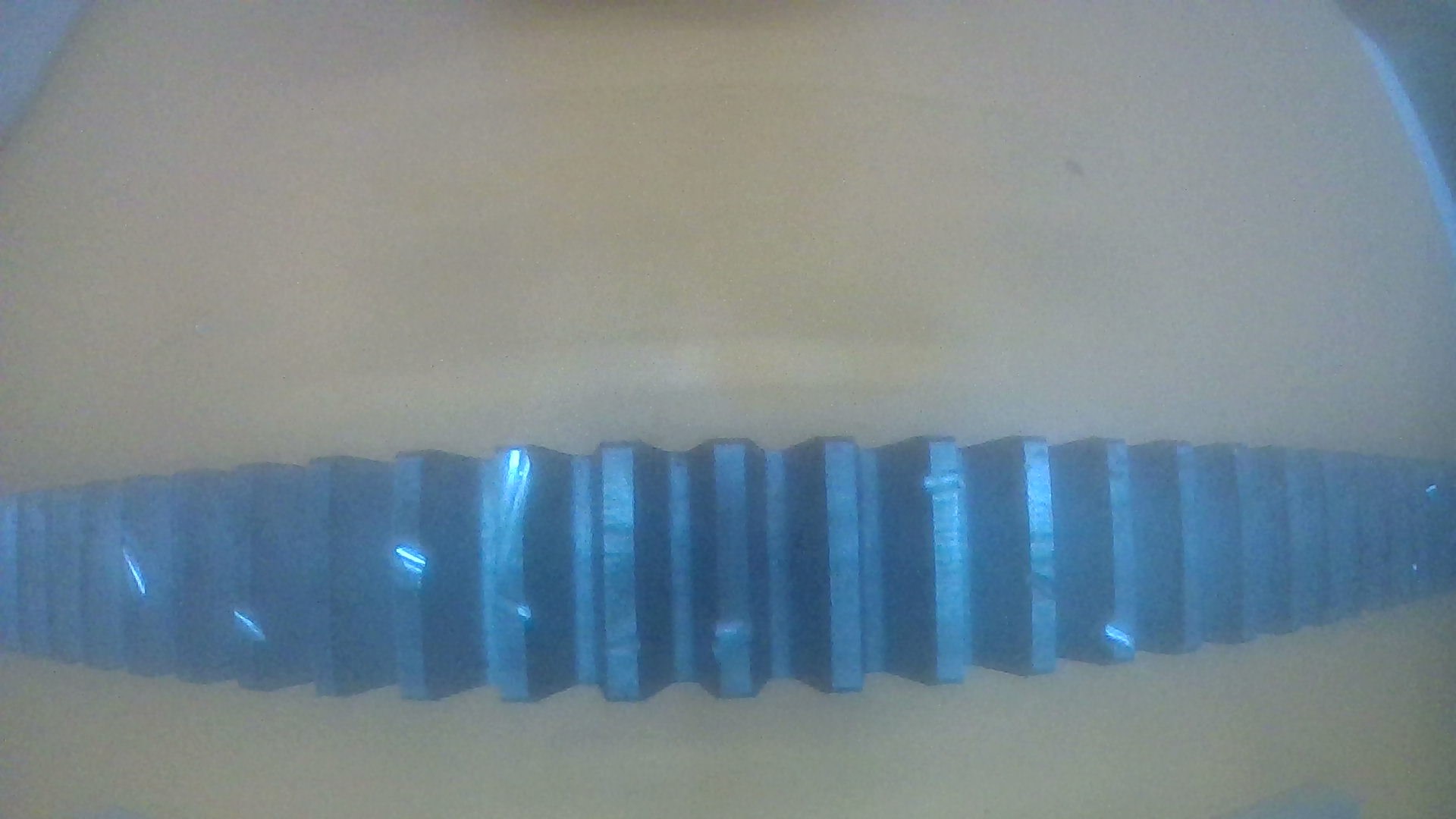

Supplement: S1 Data — (ZIP) [file pone.0322217.s001.zip › dataset/4/WIN_20250111_23_32_33_Pro.jpg]

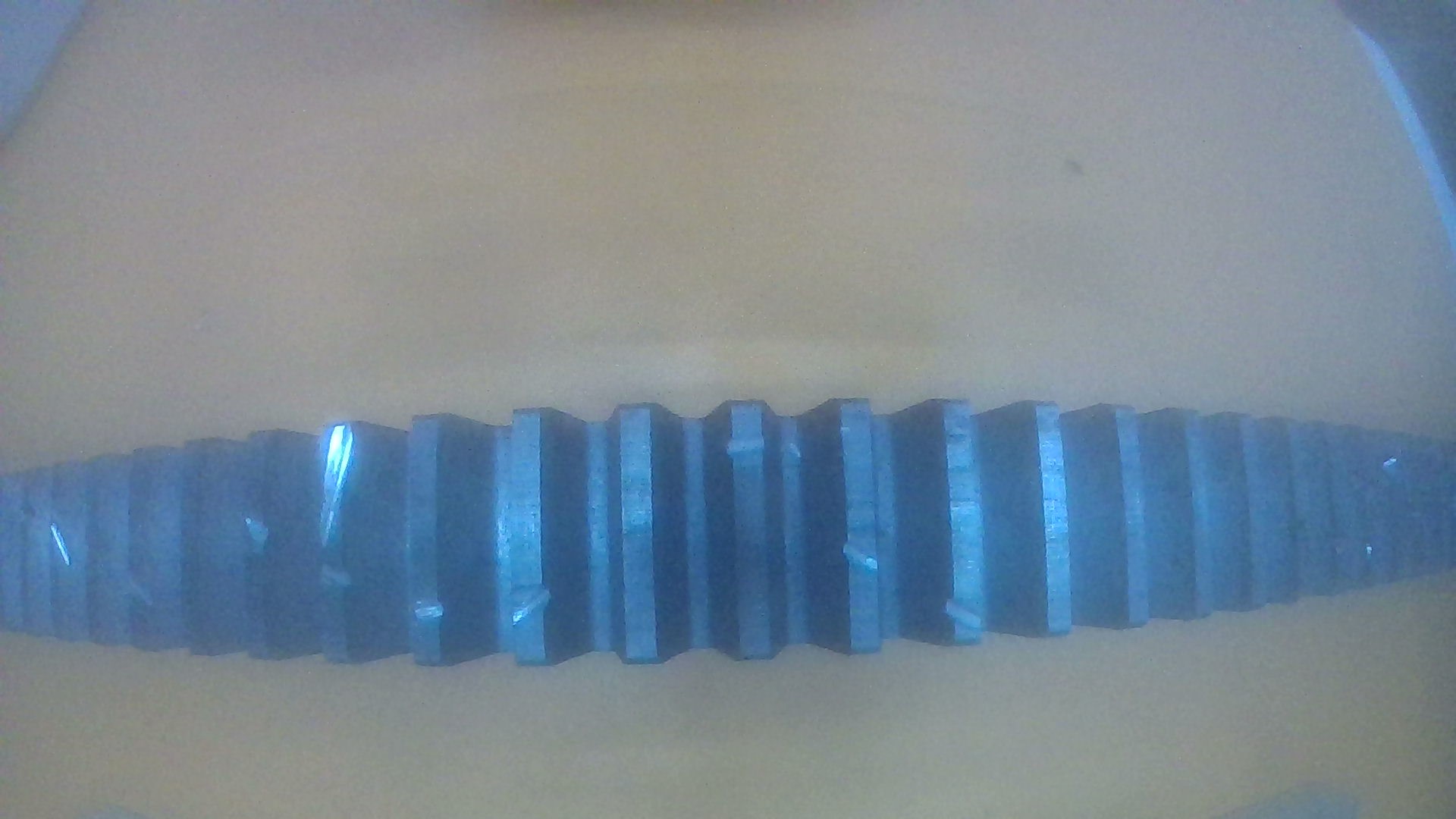

Supplement: S1 Data — (ZIP) [file pone.0322217.s001.zip › dataset/4/WIN_20250111_23_32_34_Pro.jpg]

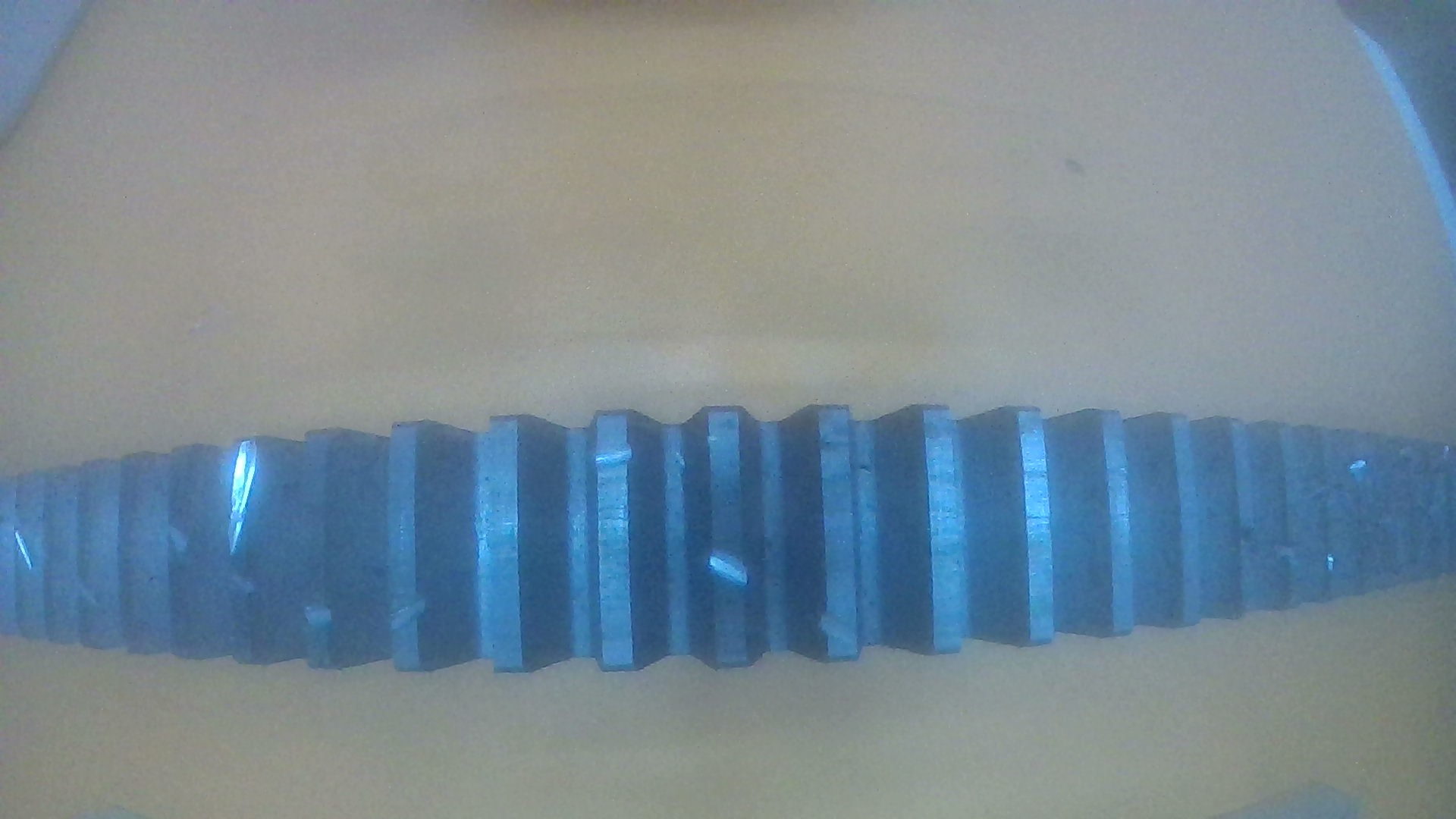

Supplement: S1 Data — (ZIP) [file pone.0322217.s001.zip › dataset/4/WIN_20250111_23_32_35_Pro.jpg]

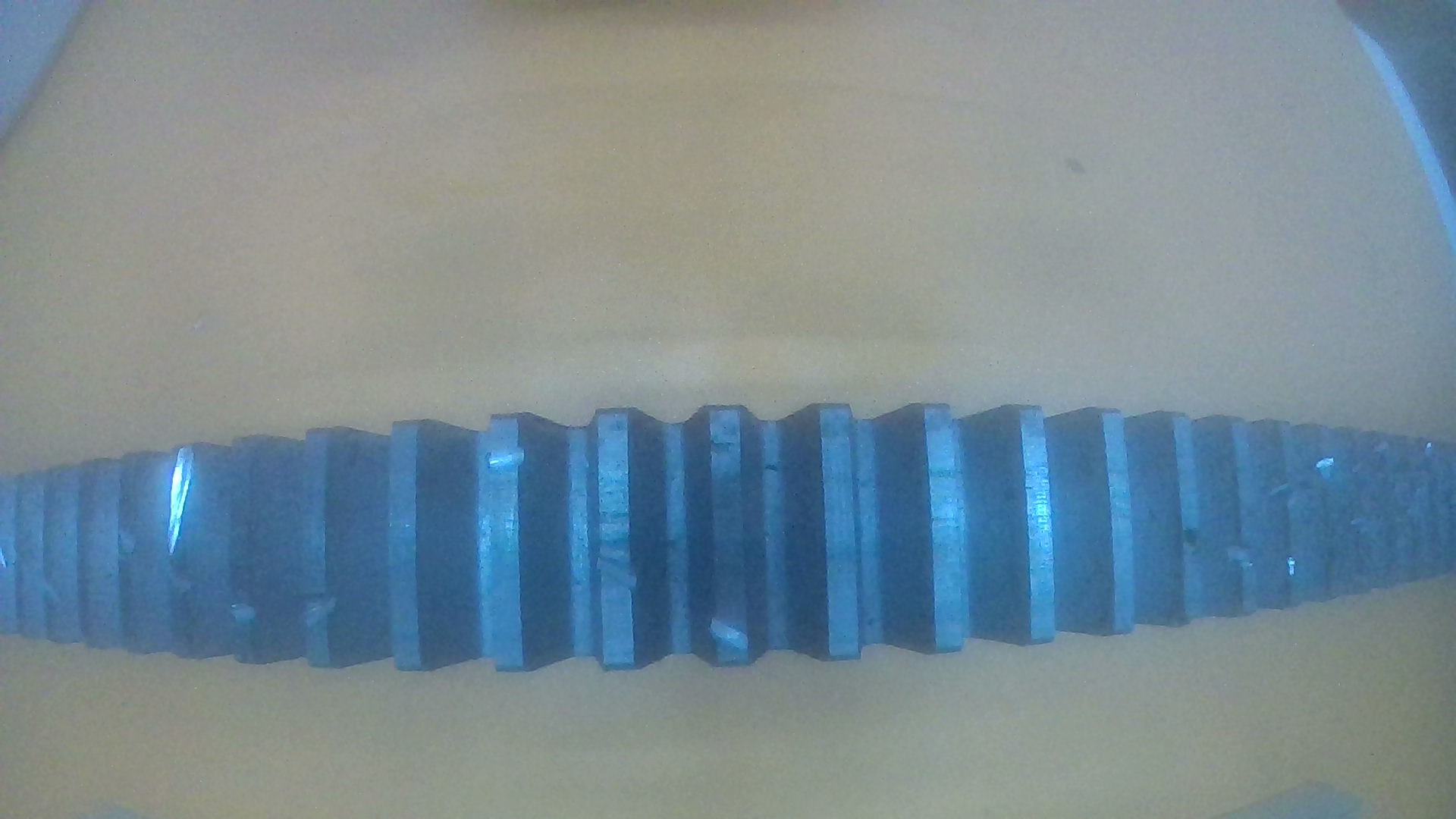

Supplement: S1 Data — (ZIP) [file pone.0322217.s001.zip › dataset/4/WIN_20250111_23_32_36_Pro.jpg]

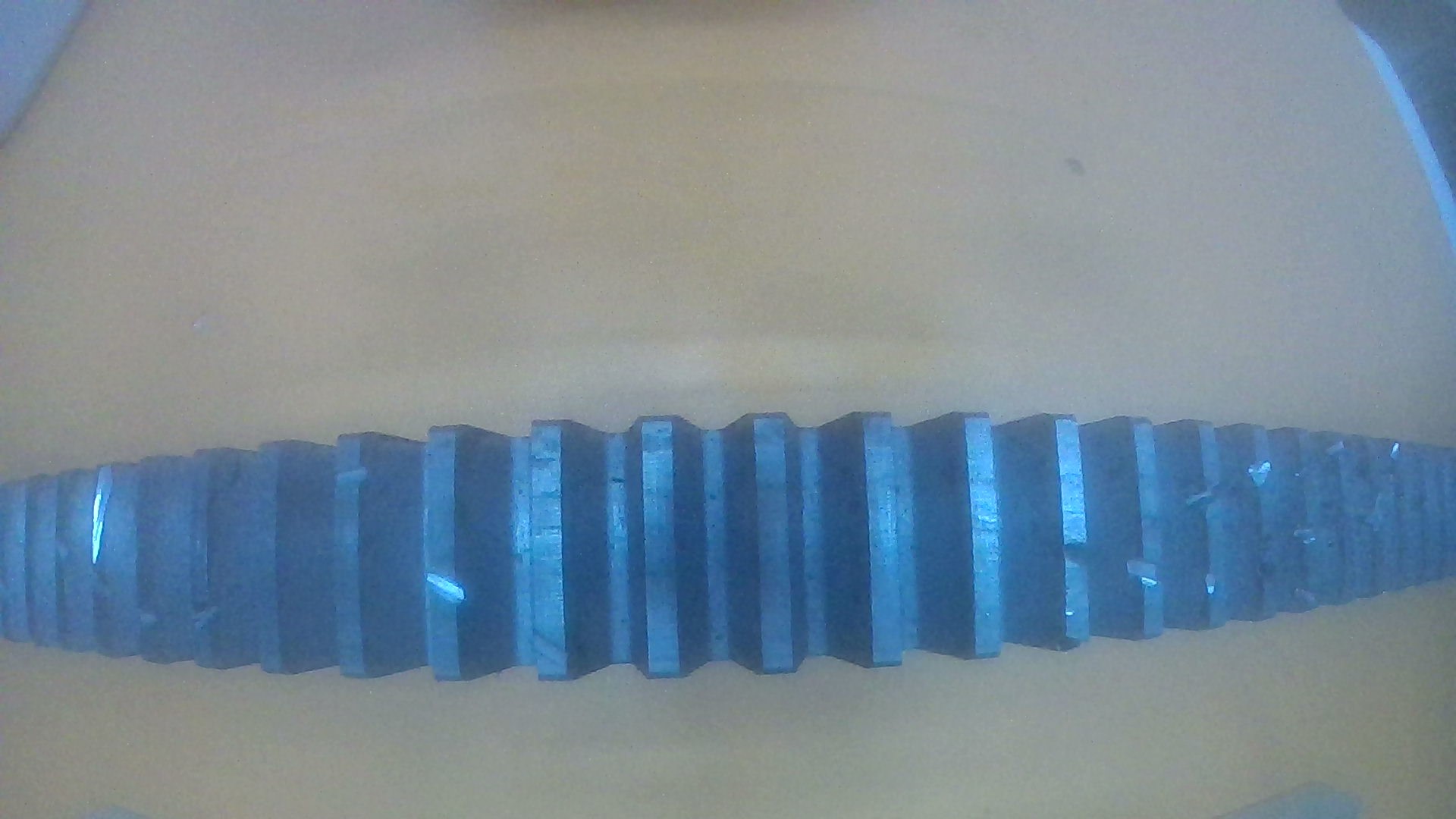

Supplement: S1 Data — (ZIP) [file pone.0322217.s001.zip › dataset/4/WIN_20250111_23_32_37_Pro.jpg]

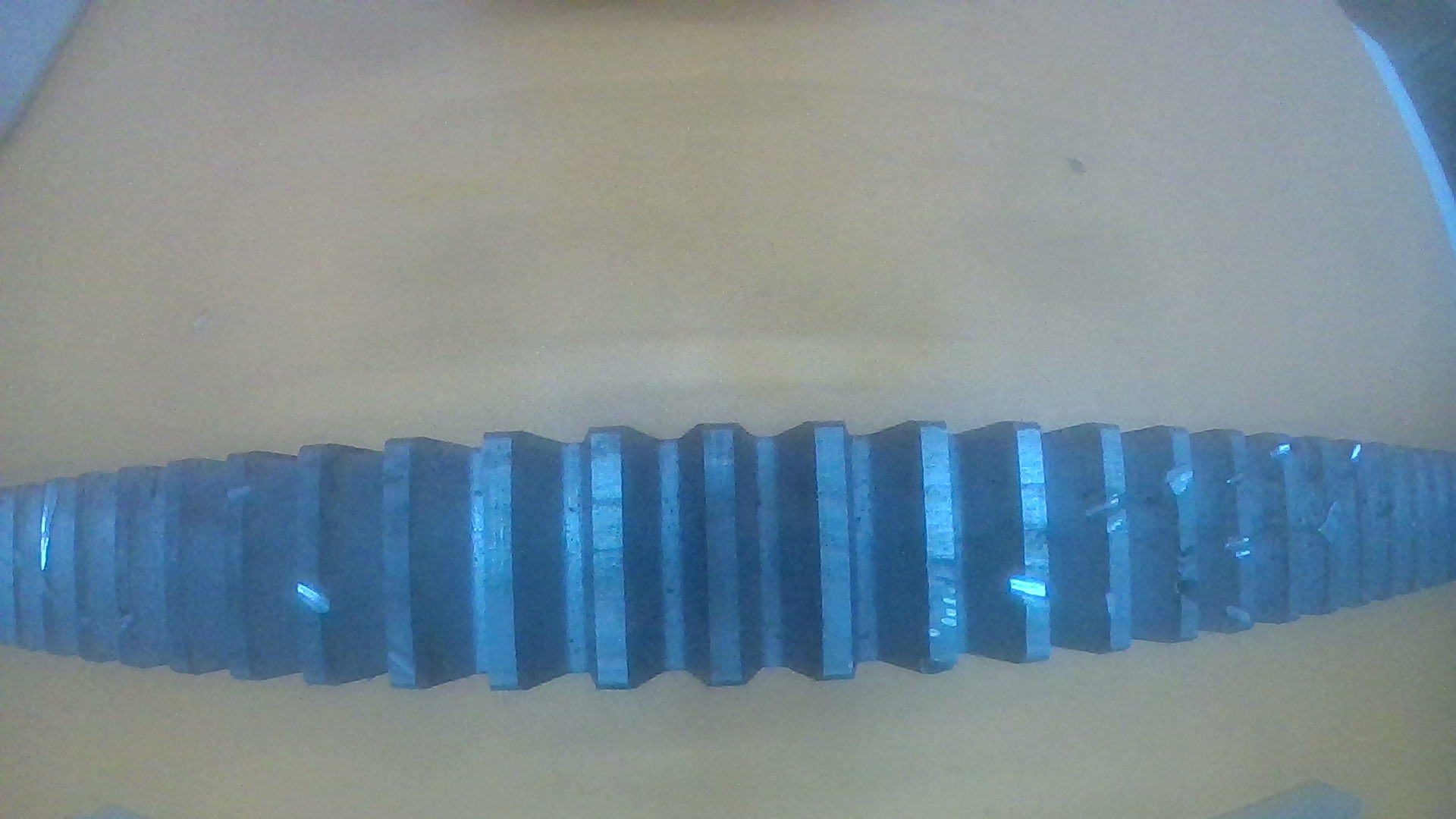

Supplement: S1 Data — (ZIP) [file pone.0322217.s001.zip › dataset/4/WIN_20250111_23_32_38_Pro.jpg]

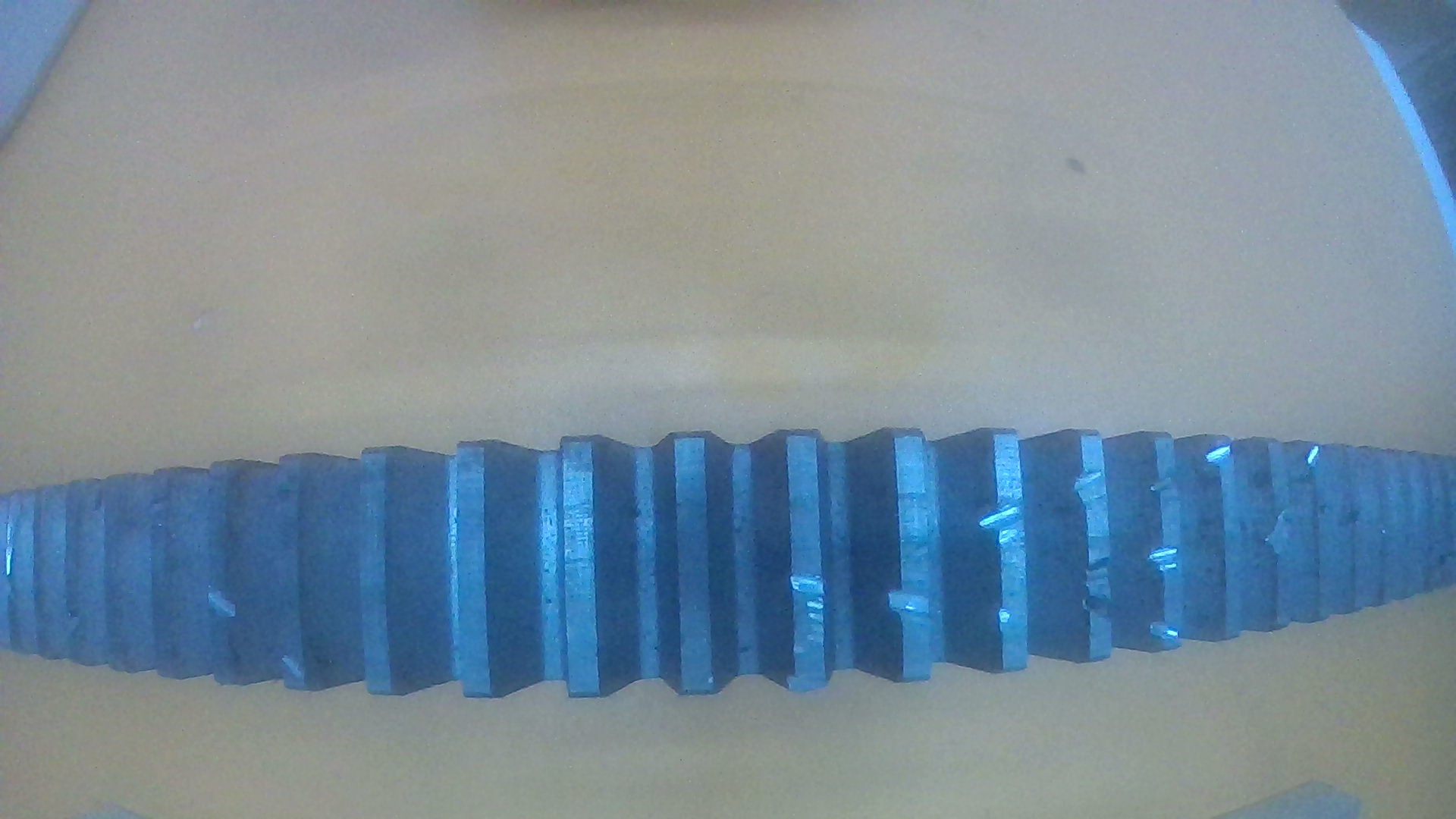

Supplement: S1 Data — (ZIP) [file pone.0322217.s001.zip › dataset/4/WIN_20250111_23_32_39_Pro.jpg]
