# Supplementary figures and images for: An image processing technique for optimizing industrial defect detection using dehazing algorithms (part 1 of 2)
Source: PLoS One. 2025 May 2;20(5):e0322217. doi: 10.1371/journal.pone.0322217 (PMC12047806; doi:10.1371/journal.pone.0322217)

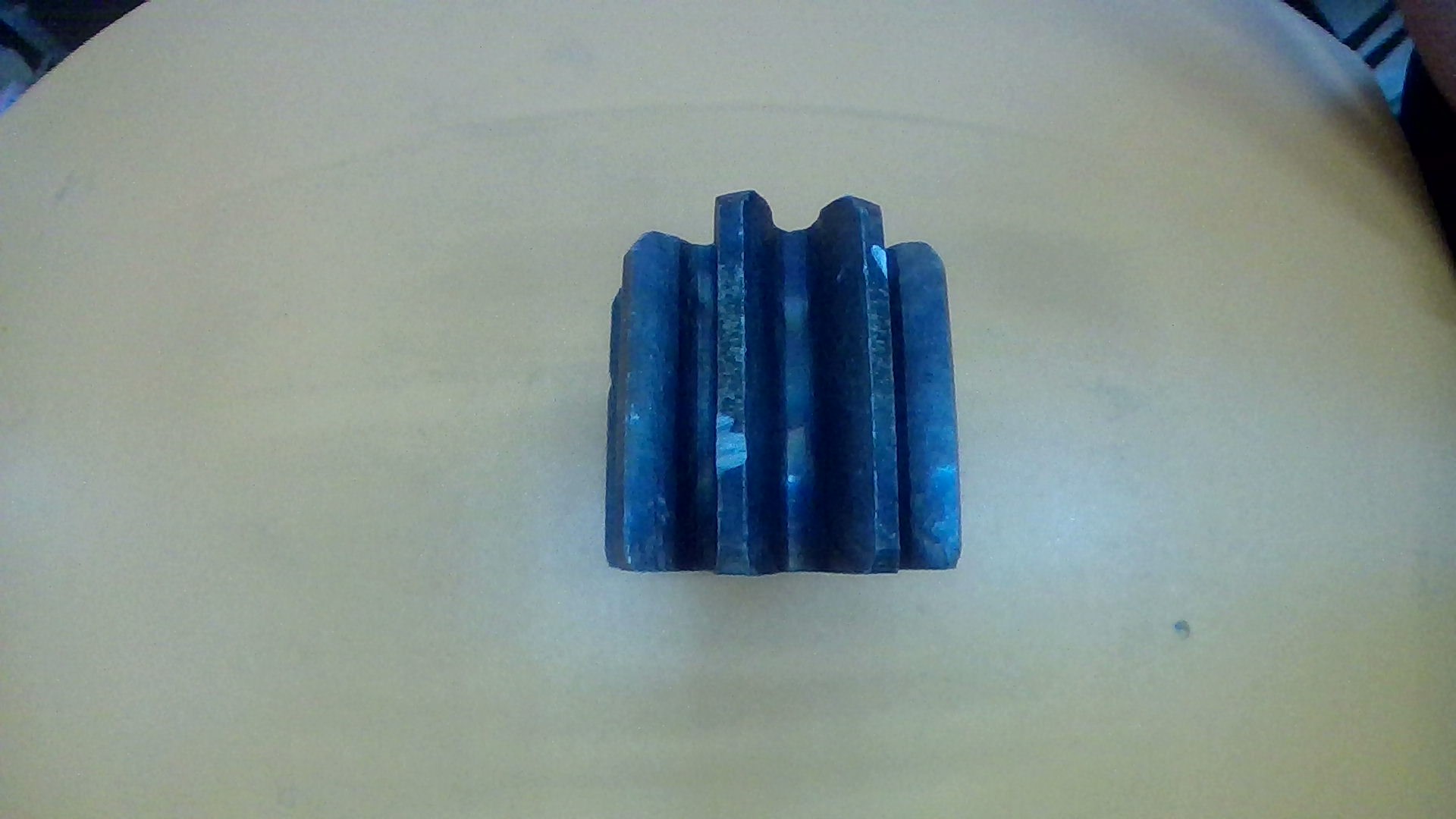

Supplement: S1 Data — (ZIP) [file pone.0322217.s001.zip › dataset/1/WIN_20250112_14_38_00_Pro.jpg]

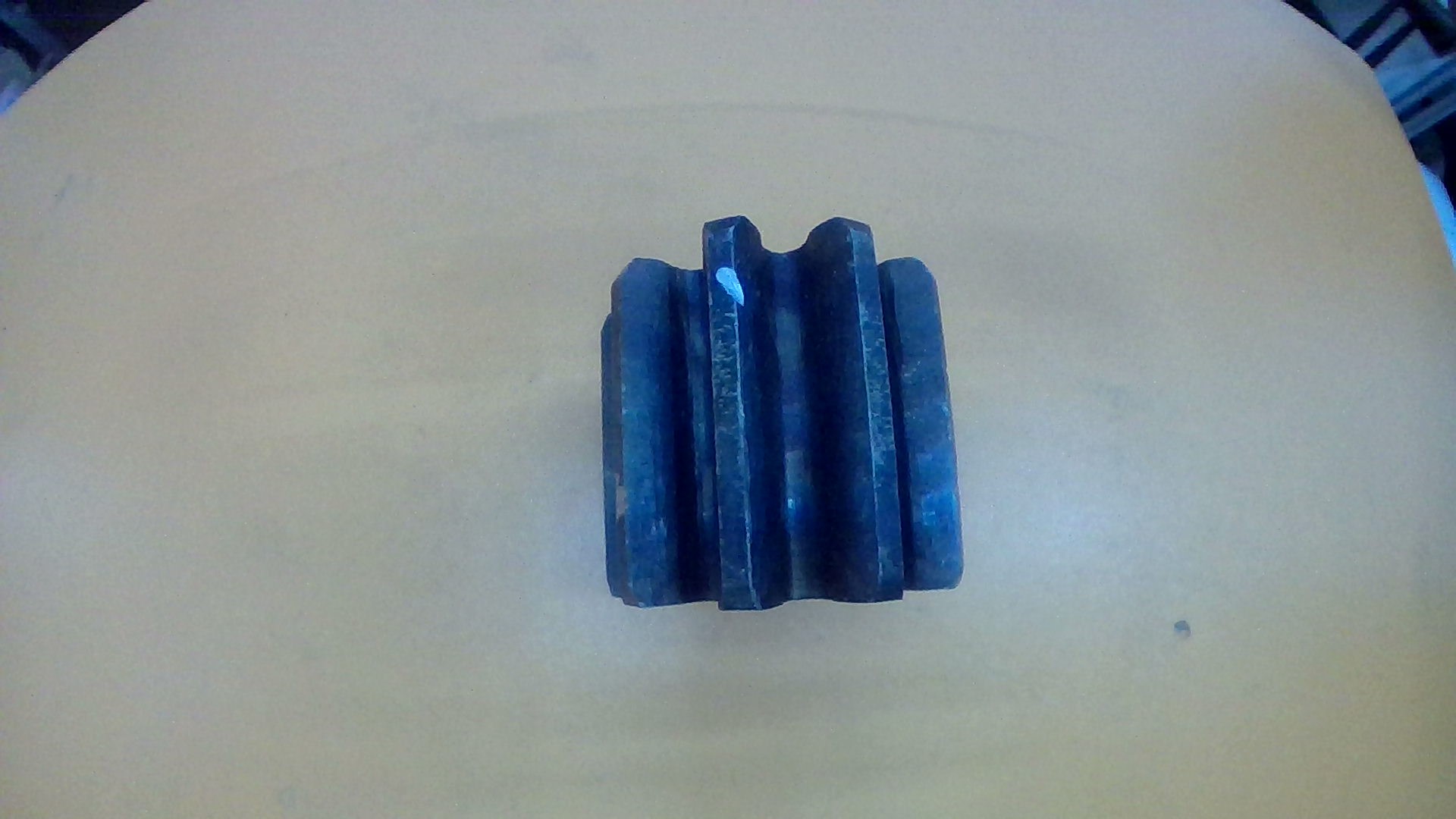

Supplement: S1 Data — (ZIP) [file pone.0322217.s001.zip › dataset/1/WIN_20250112_14_38_06_Pro.jpg]

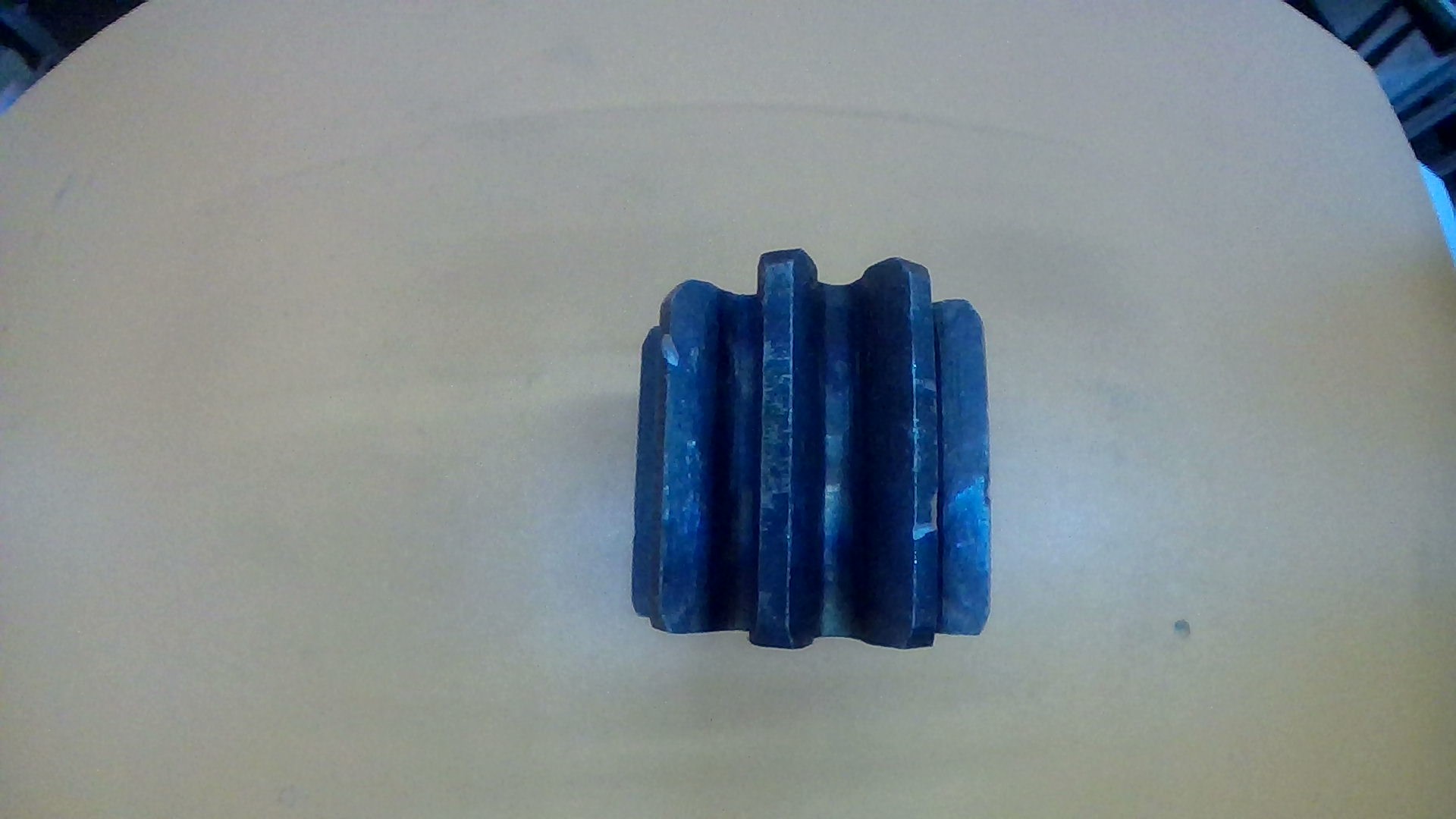

Supplement: S1 Data — (ZIP) [file pone.0322217.s001.zip › dataset/1/WIN_20250112_14_38_12_Pro.jpg]

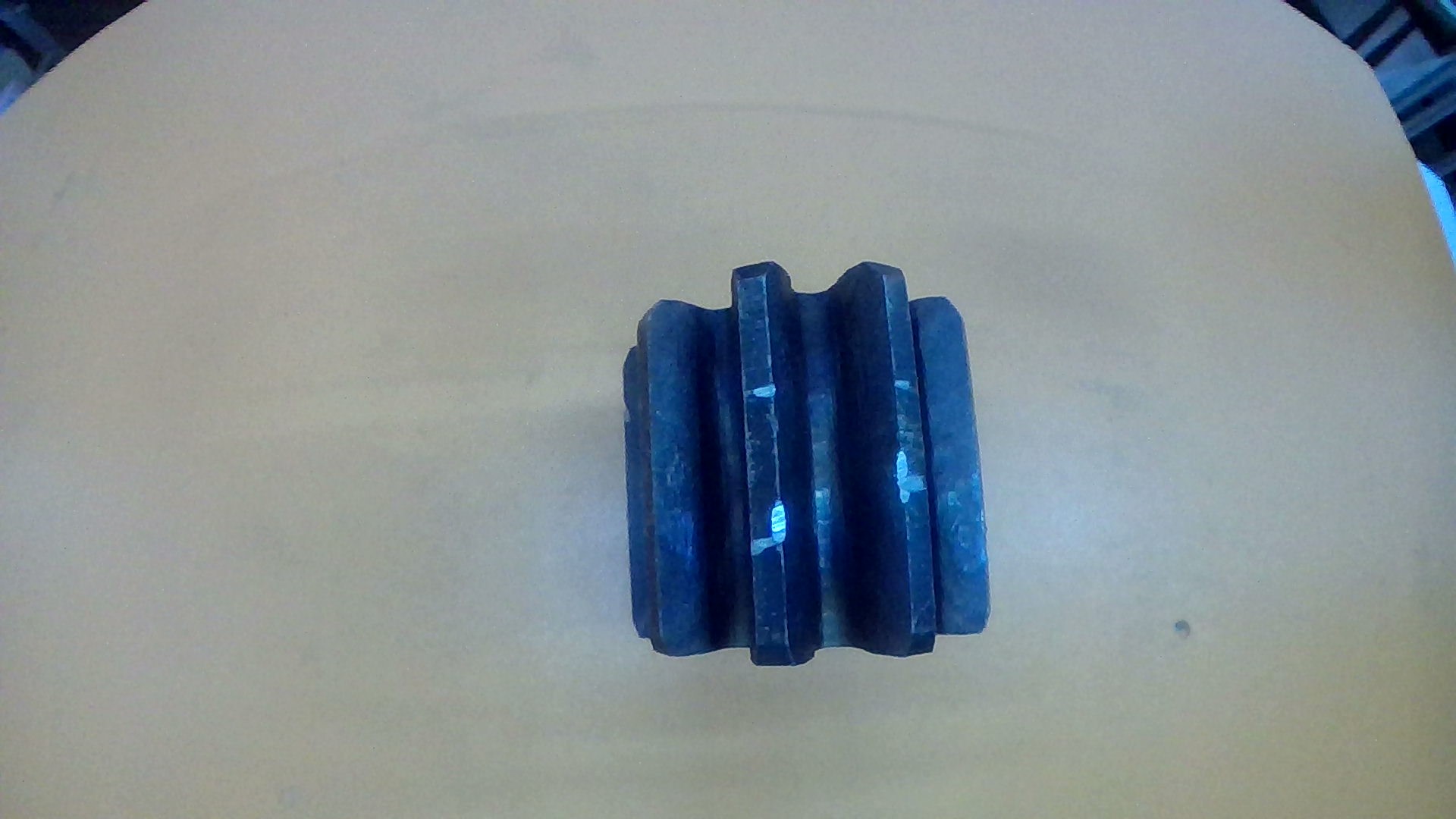

Supplement: S1 Data — (ZIP) [file pone.0322217.s001.zip › dataset/1/WIN_20250112_14_38_18_Pro.jpg]

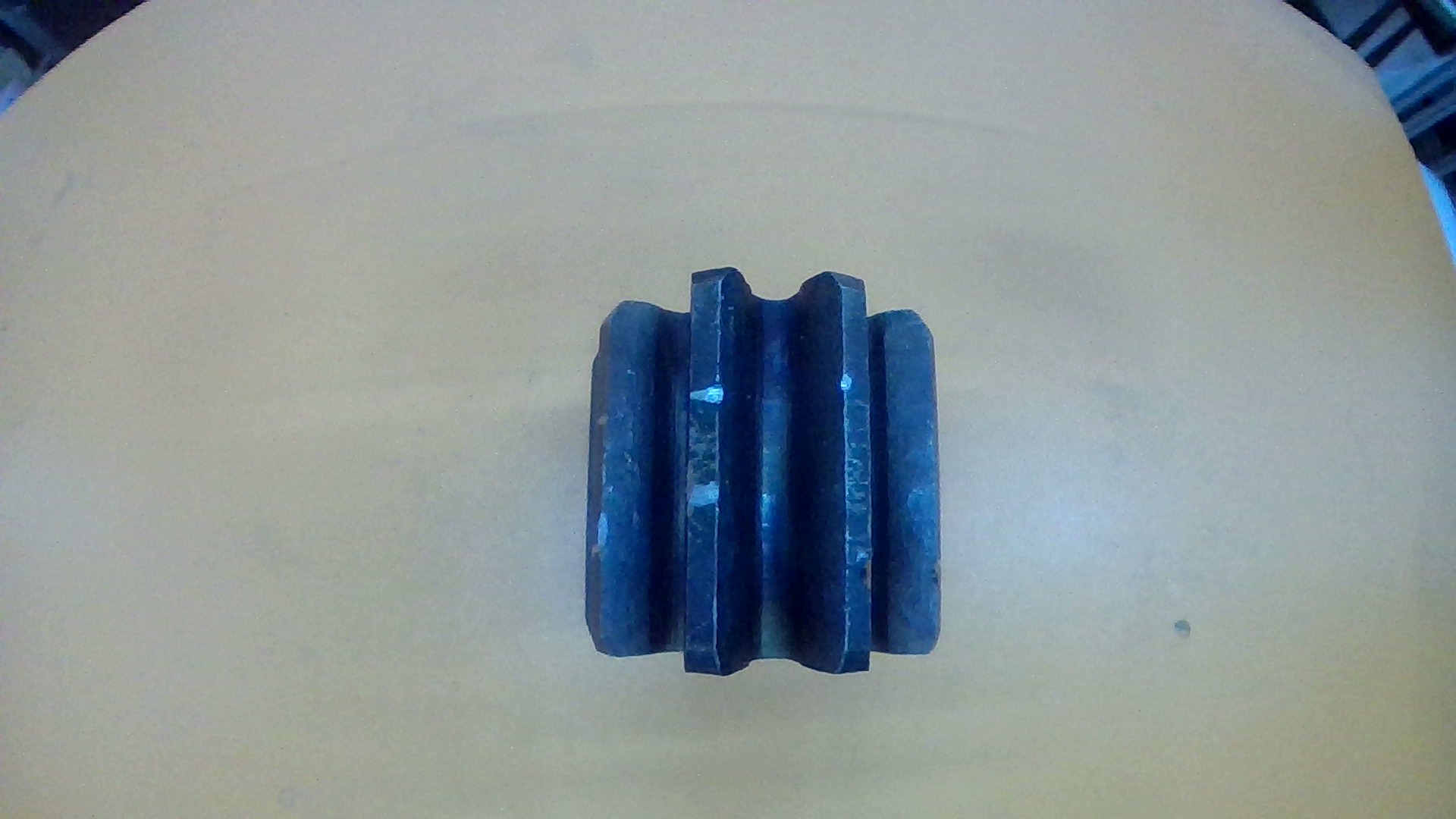

Supplement: S1 Data — (ZIP) [file pone.0322217.s001.zip › dataset/1/WIN_20250112_14_38_22_Pro.jpg]

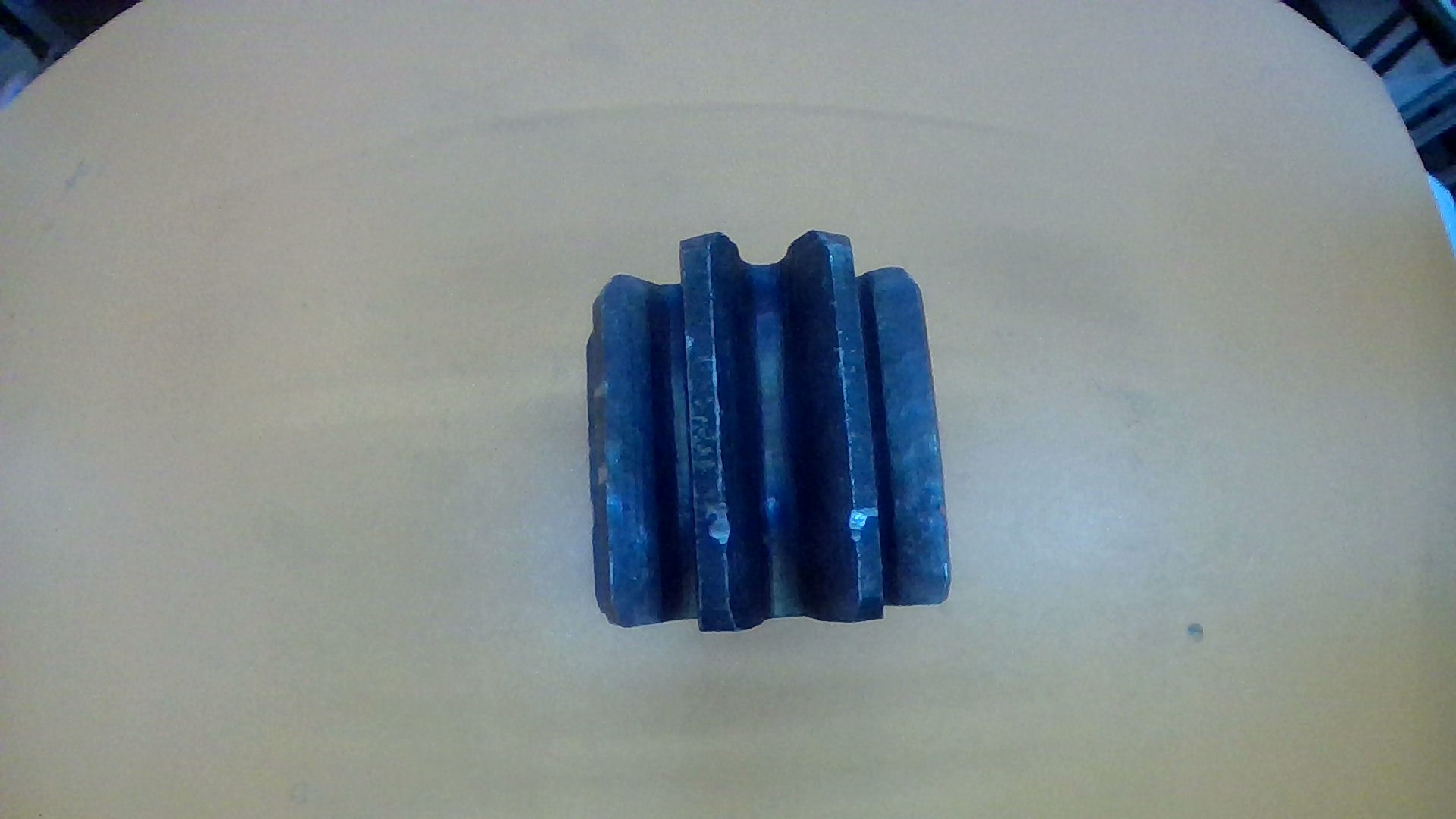

Supplement: S1 Data — (ZIP) [file pone.0322217.s001.zip › dataset/1/WIN_20250112_14_38_27_Pro.jpg]

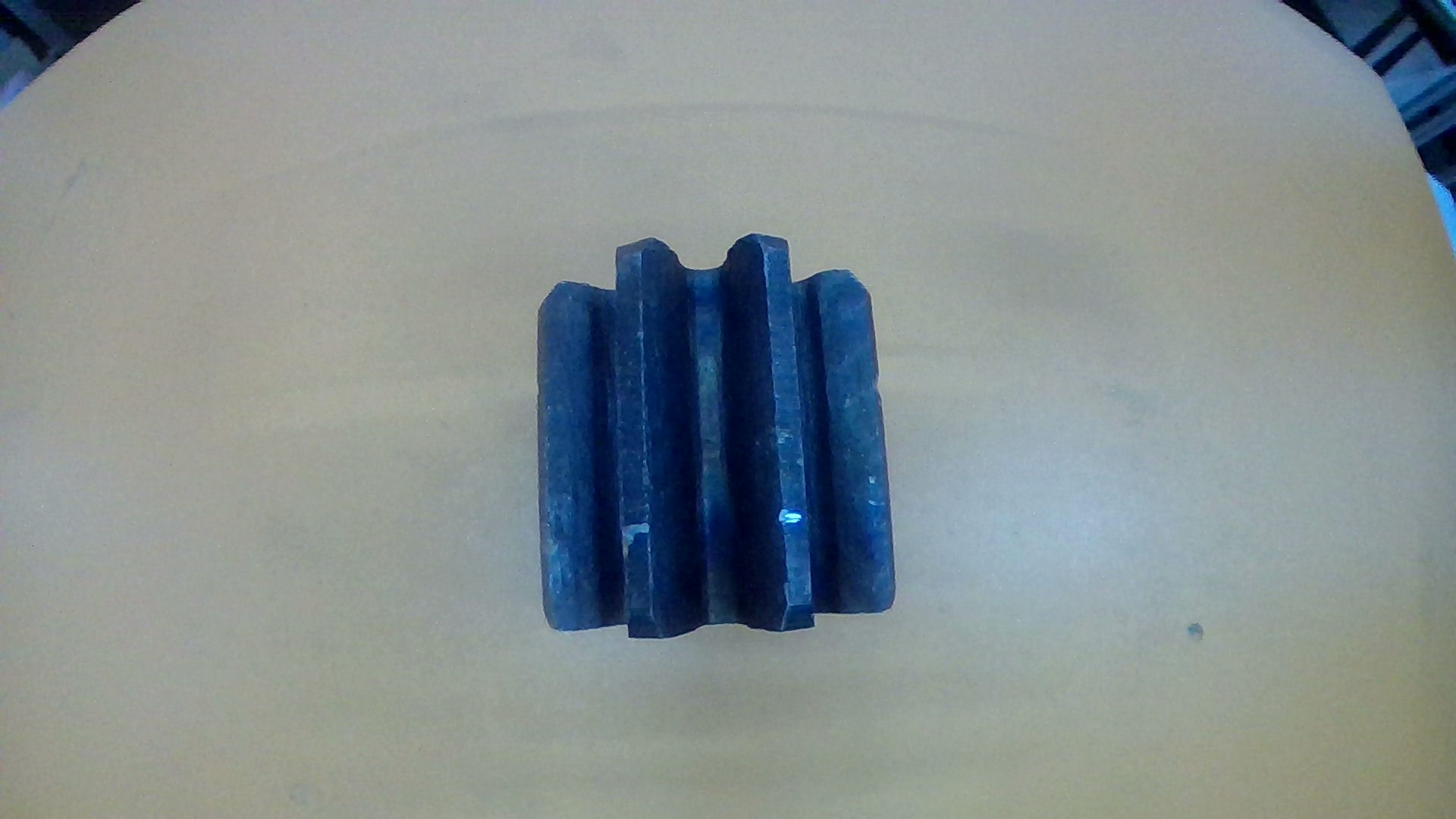

Supplement: S1 Data — (ZIP) [file pone.0322217.s001.zip › dataset/1/WIN_20250112_14_38_31_Pro.jpg]

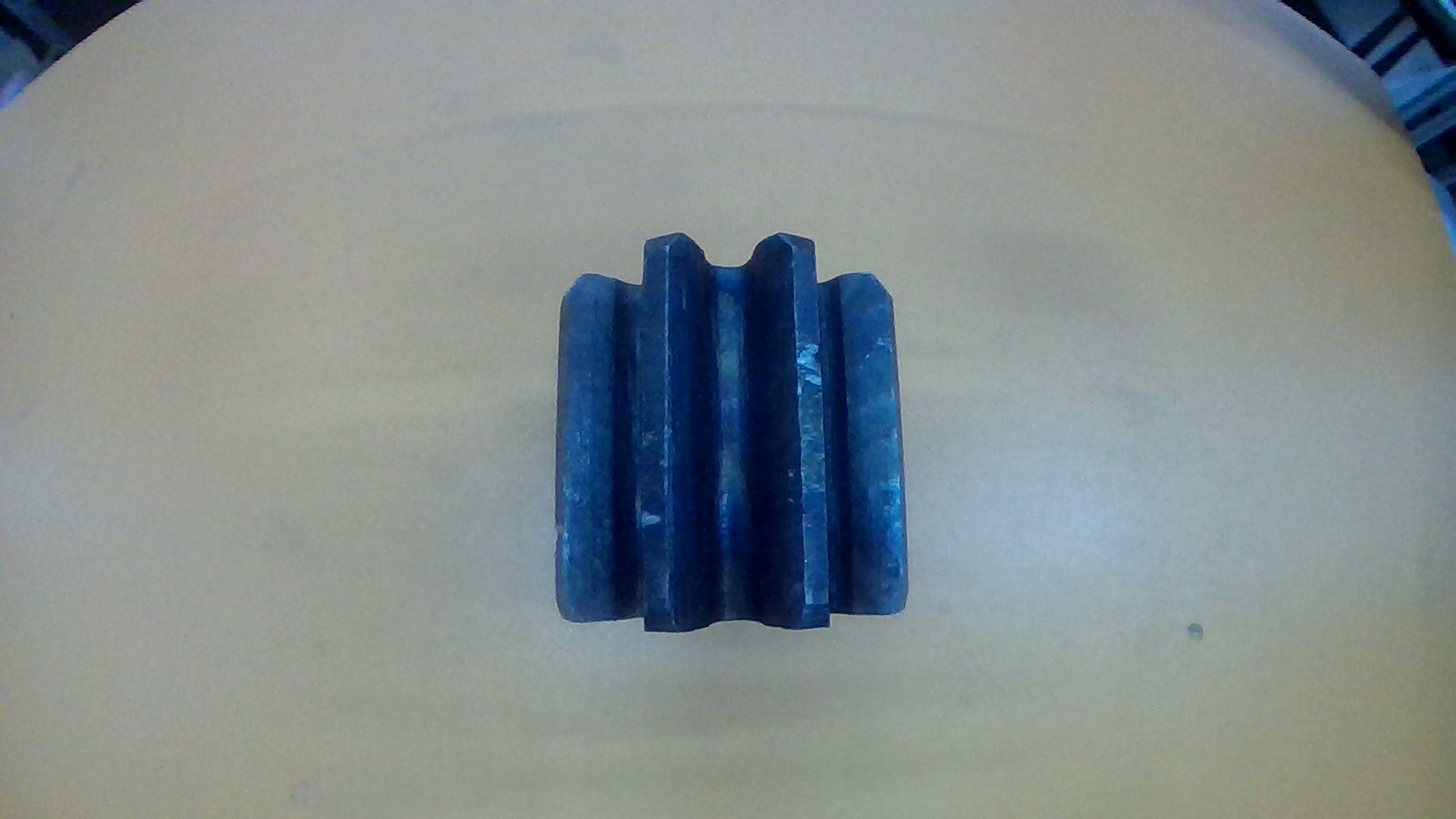

Supplement: S1 Data — (ZIP) [file pone.0322217.s001.zip › dataset/1/WIN_20250112_14_38_36_Pro.jpg]

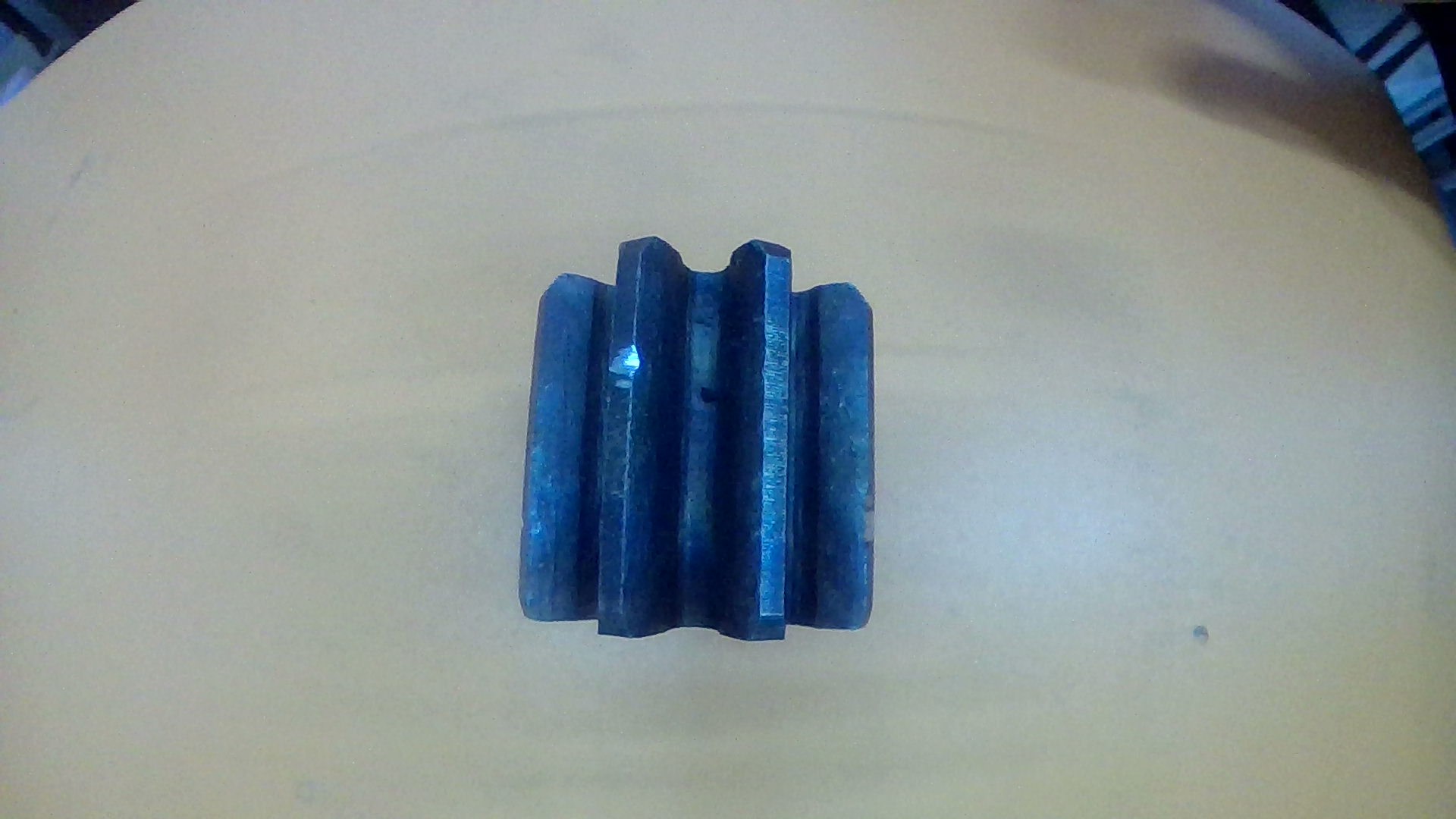

Supplement: S1 Data — (ZIP) [file pone.0322217.s001.zip › dataset/1/WIN_20250112_14_38_39_Pro.jpg]

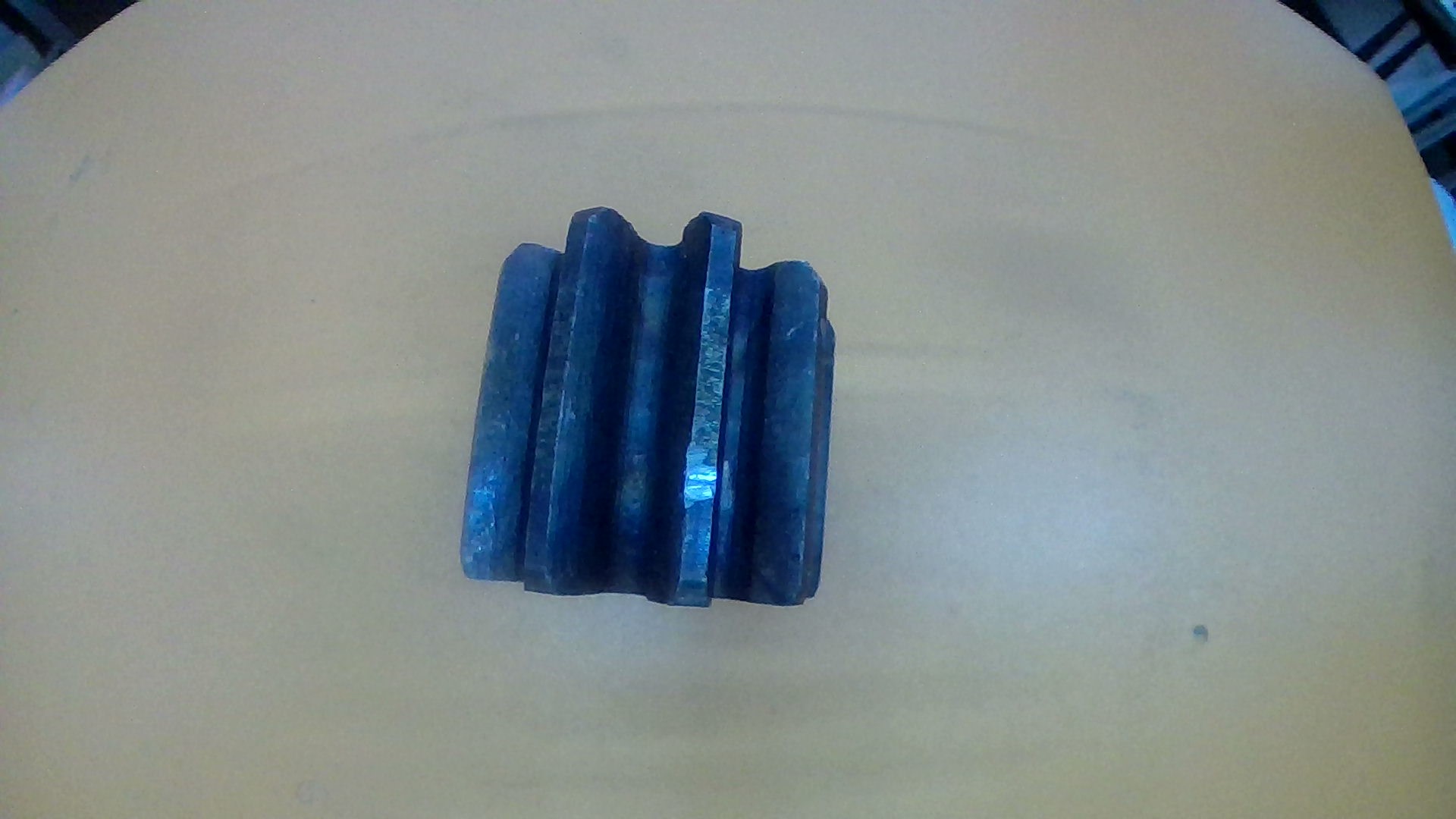

Supplement: S1 Data — (ZIP) [file pone.0322217.s001.zip › dataset/1/WIN_20250112_14_38_43_Pro.jpg]

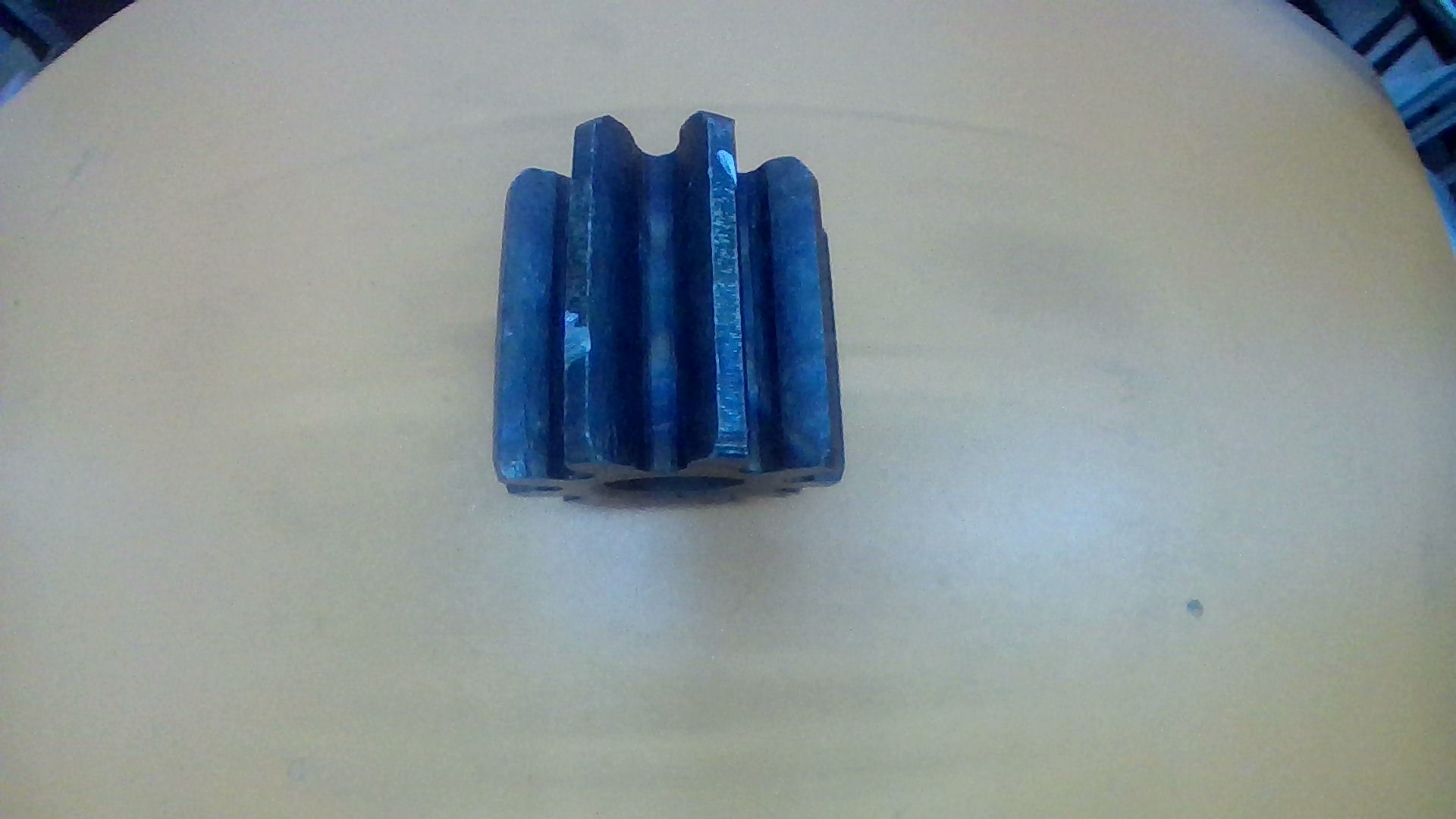

Supplement: S1 Data — (ZIP) [file pone.0322217.s001.zip › dataset/1/WIN_20250112_14_38_46_Pro.jpg]

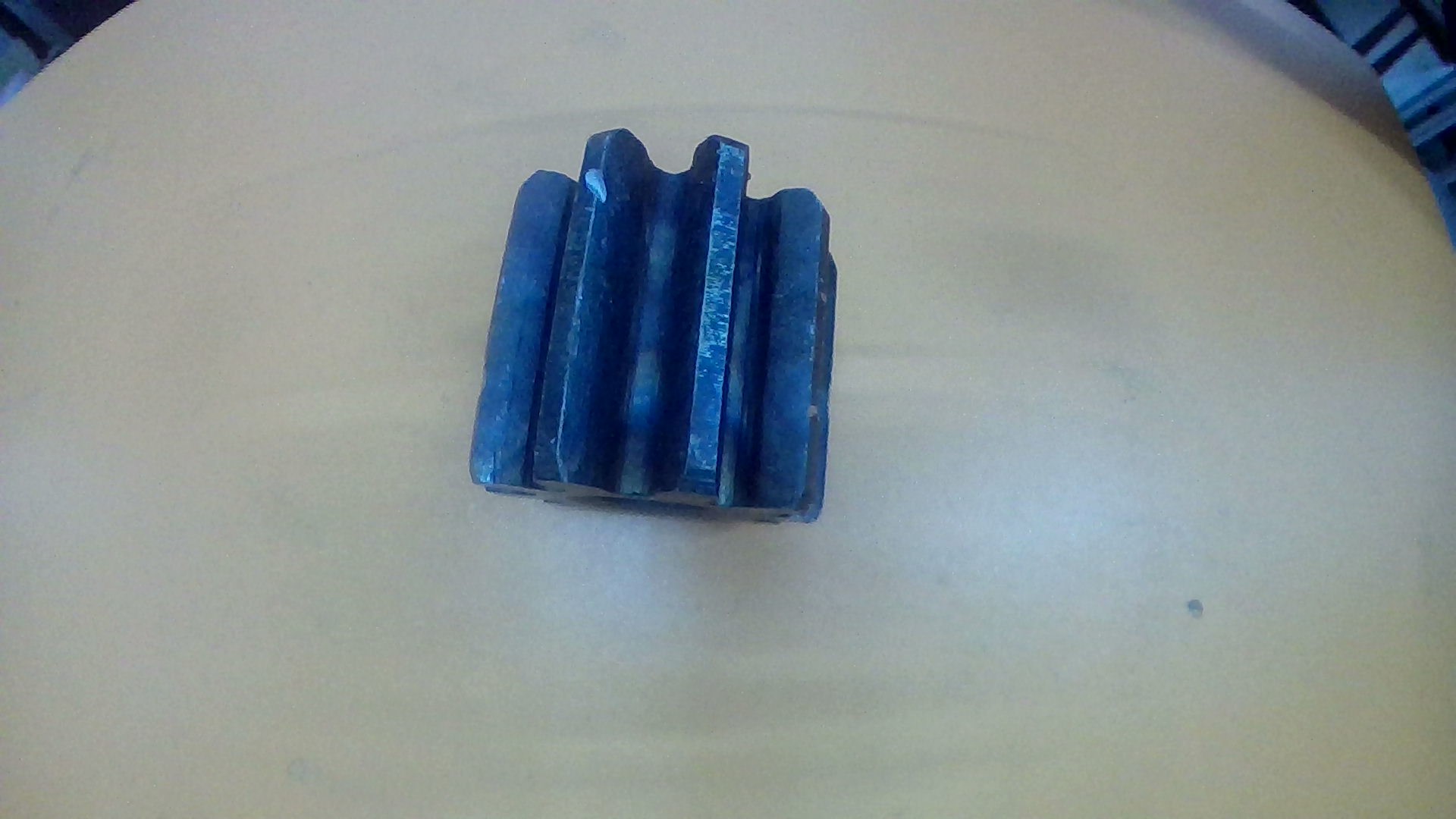

Supplement: S1 Data — (ZIP) [file pone.0322217.s001.zip › dataset/1/WIN_20250112_14_38_49_Pro.jpg]

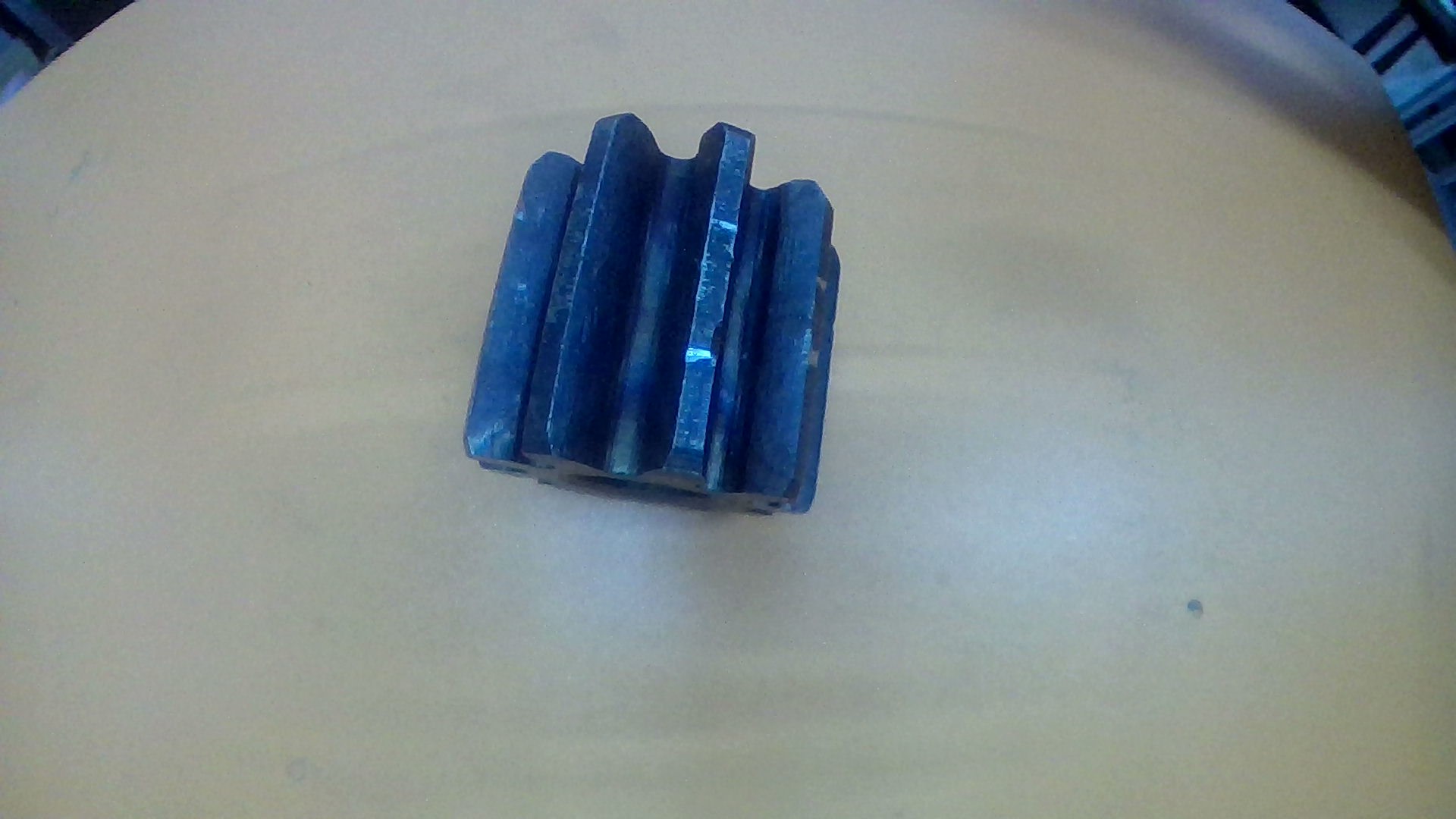

Supplement: S1 Data — (ZIP) [file pone.0322217.s001.zip › dataset/1/WIN_20250112_14_38_53_Pro.jpg]

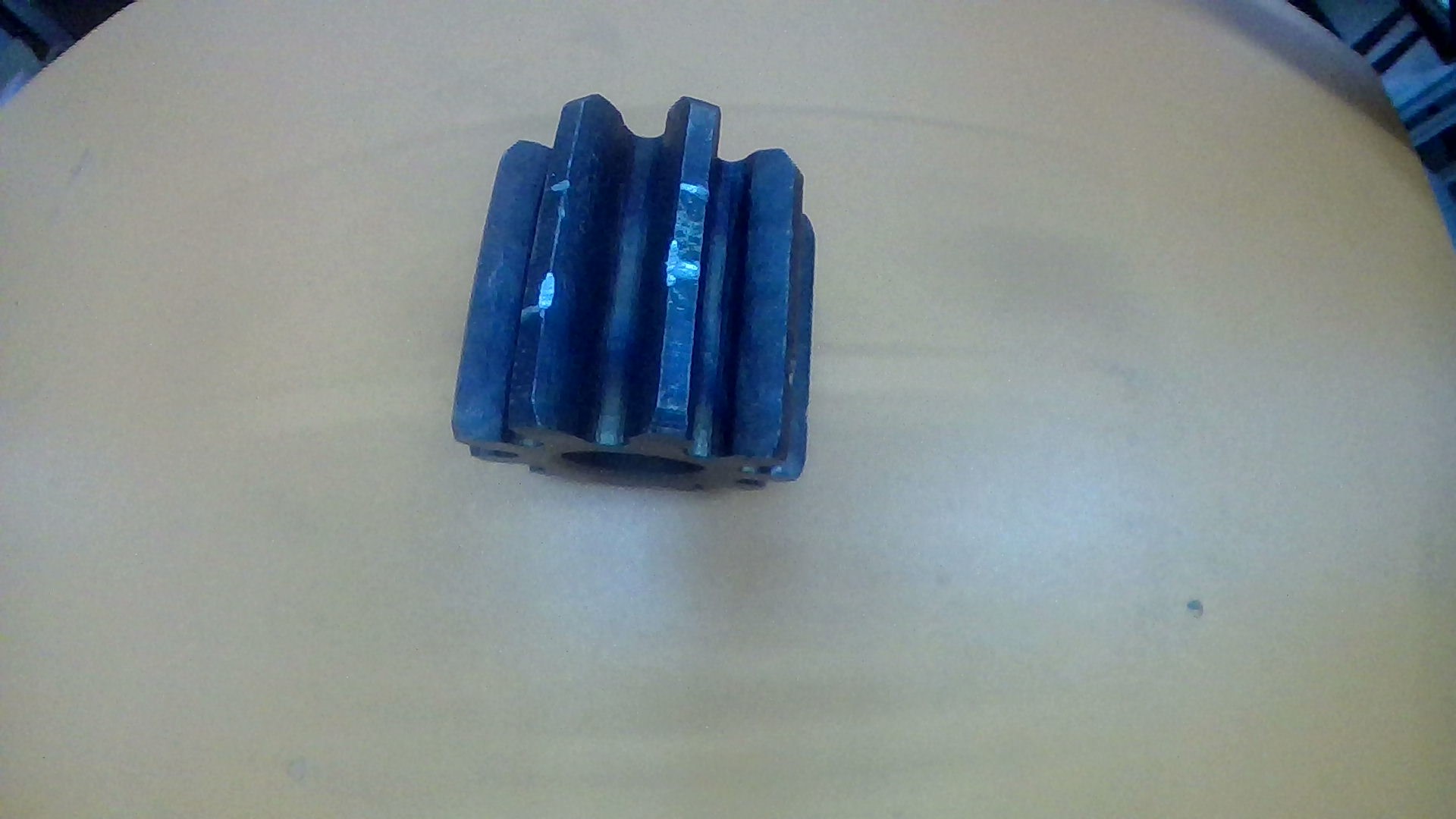

Supplement: S1 Data — (ZIP) [file pone.0322217.s001.zip › dataset/1/WIN_20250112_14_38_56_Pro.jpg]

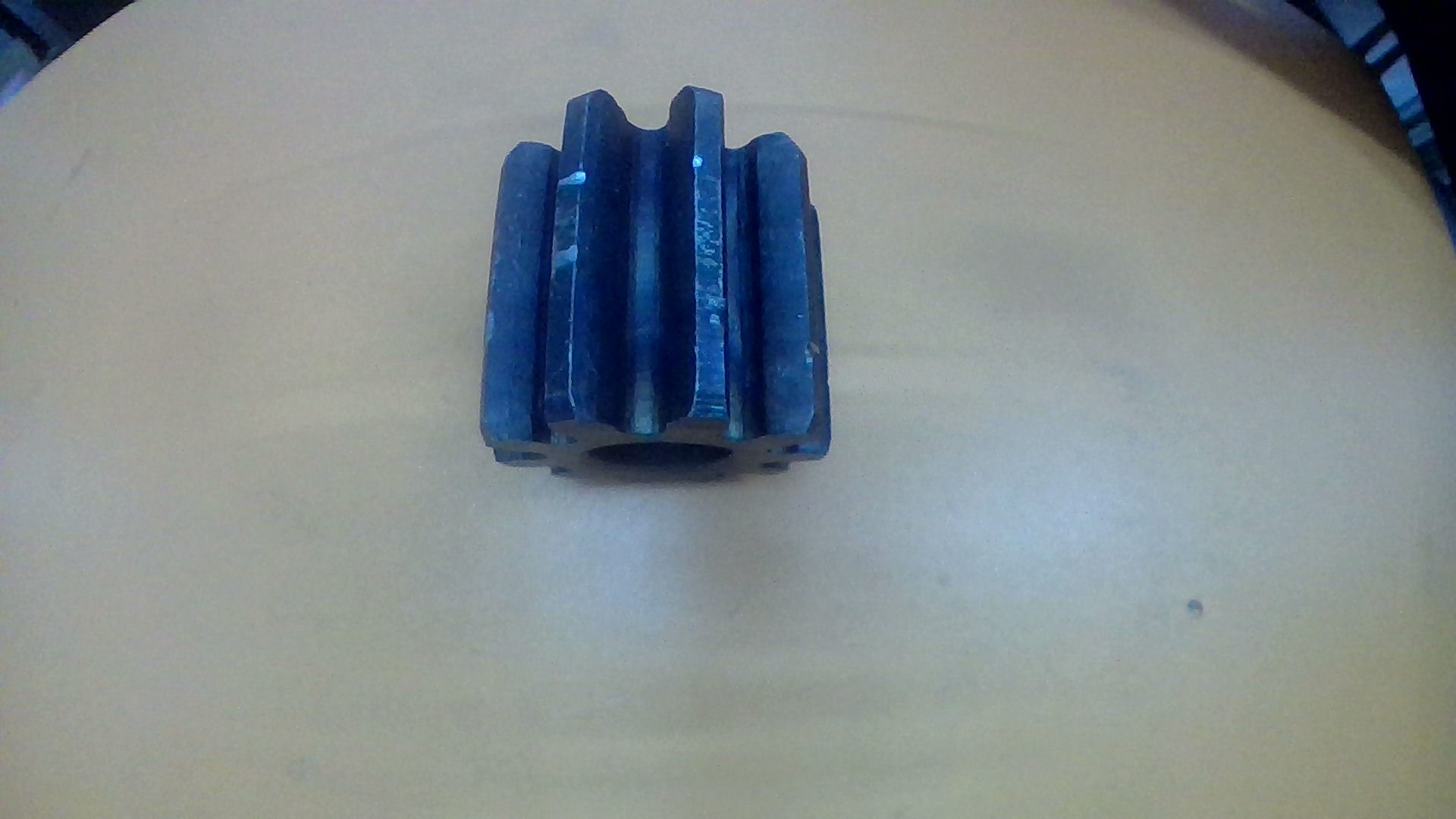

Supplement: S1 Data — (ZIP) [file pone.0322217.s001.zip › dataset/1/WIN_20250112_14_38_59_Pro.jpg]

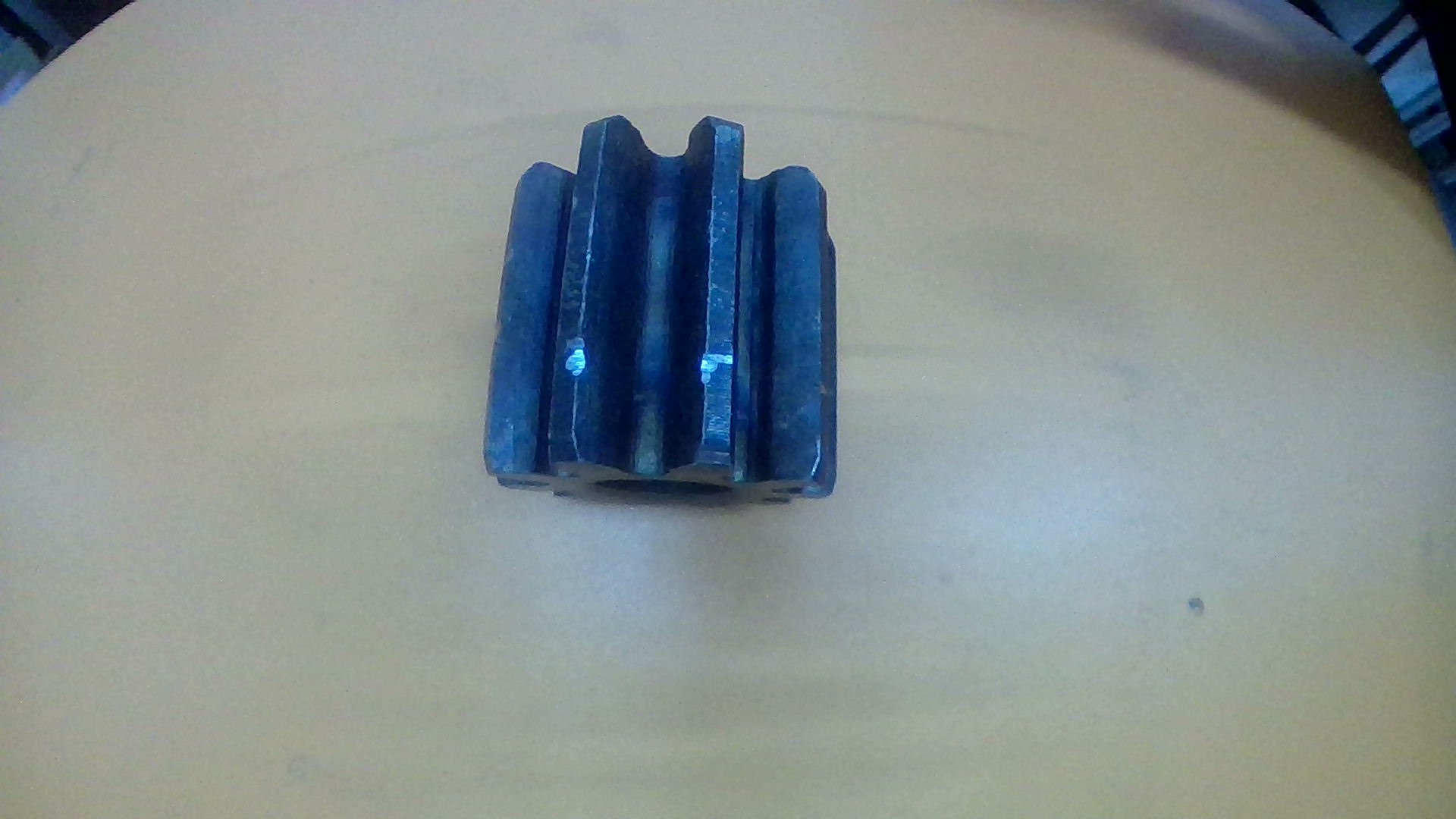

Supplement: S1 Data — (ZIP) [file pone.0322217.s001.zip › dataset/1/WIN_20250112_14_39_02_Pro.jpg]

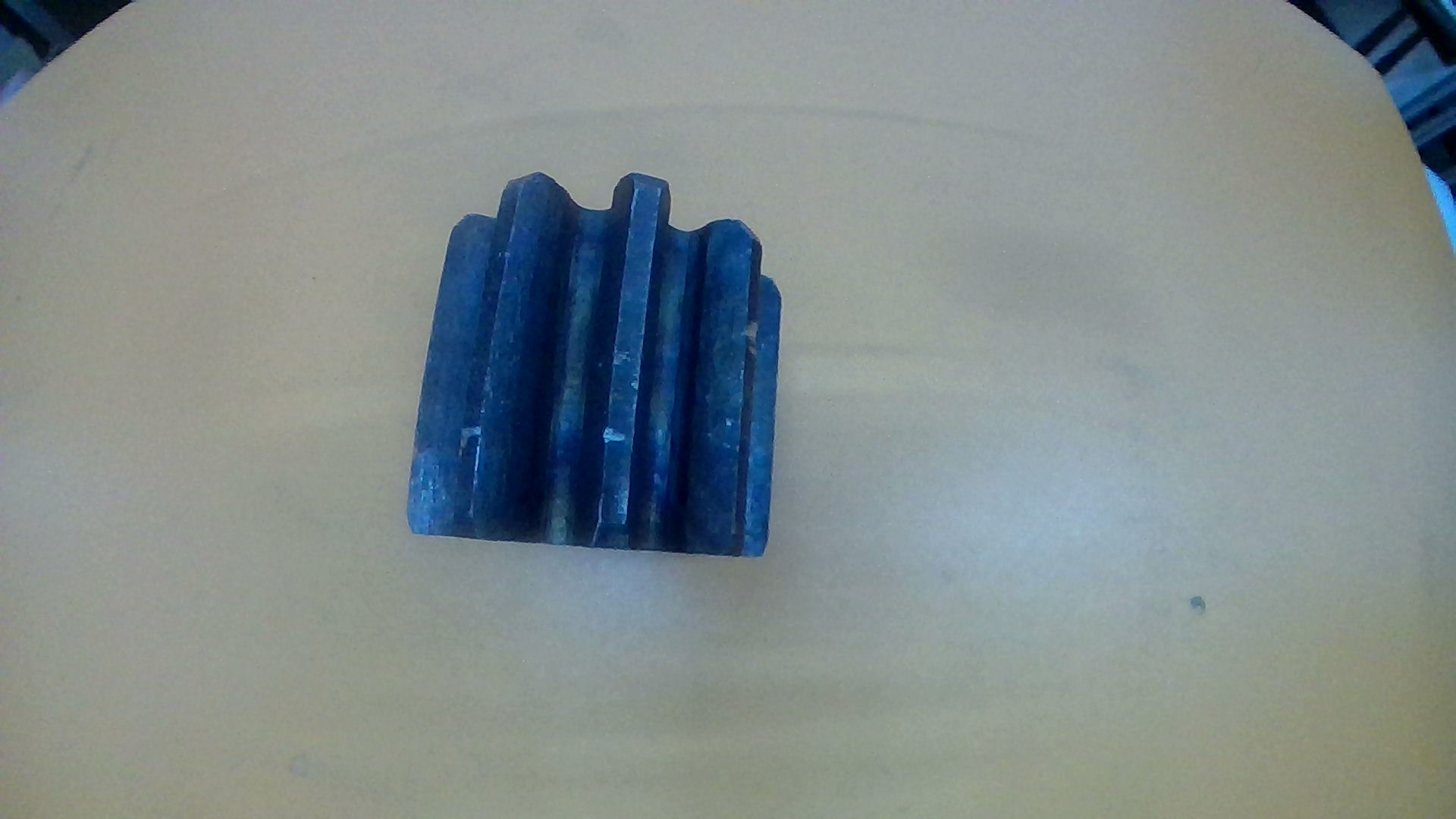

Supplement: S1 Data — (ZIP) [file pone.0322217.s001.zip › dataset/1/WIN_20250112_14_39_05_Pro.jpg]

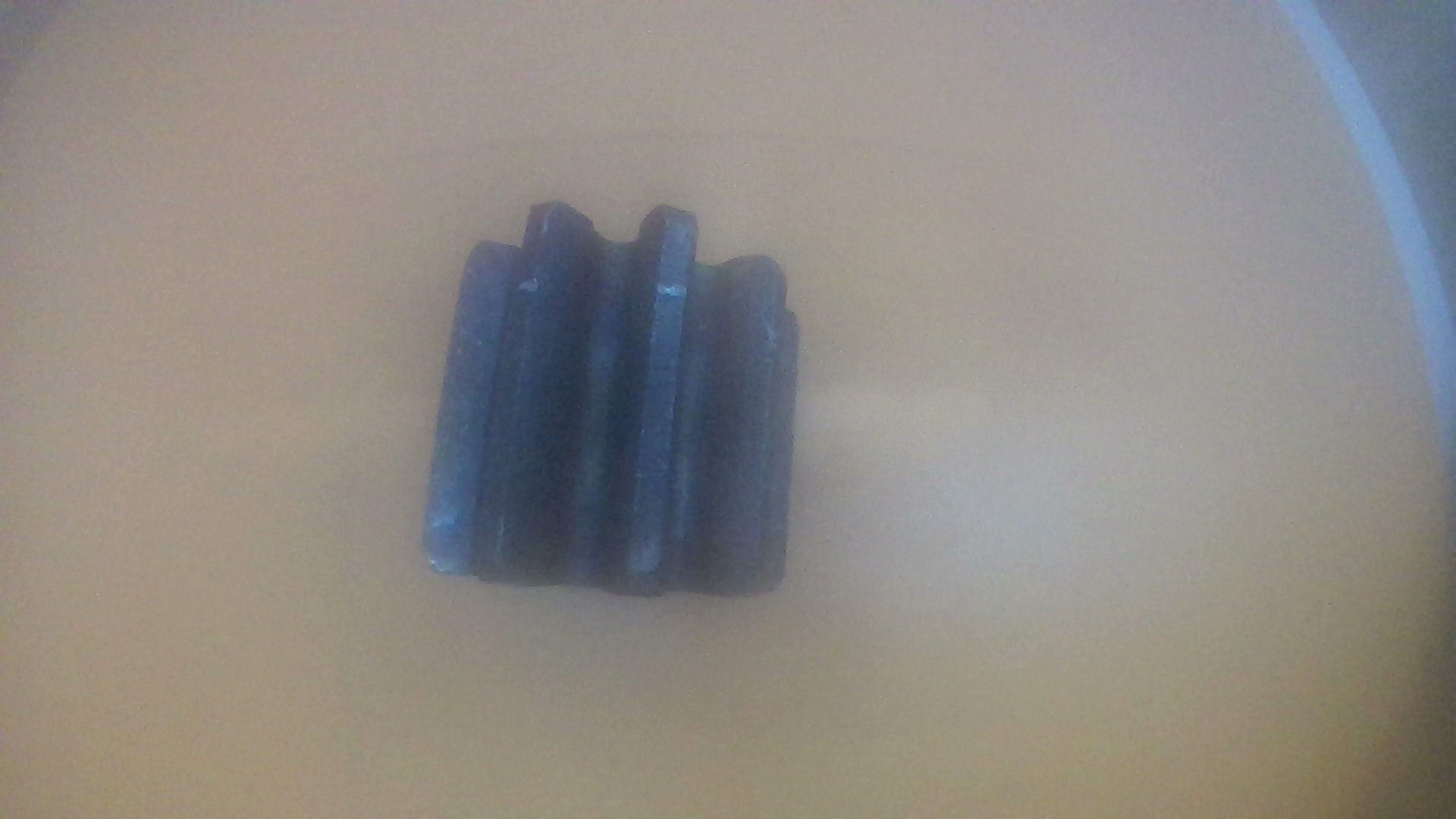

Supplement: S1 Data — (ZIP) [file pone.0322217.s001.zip › dataset/1/WIN_20250112_14_40_13_Pro.jpg]

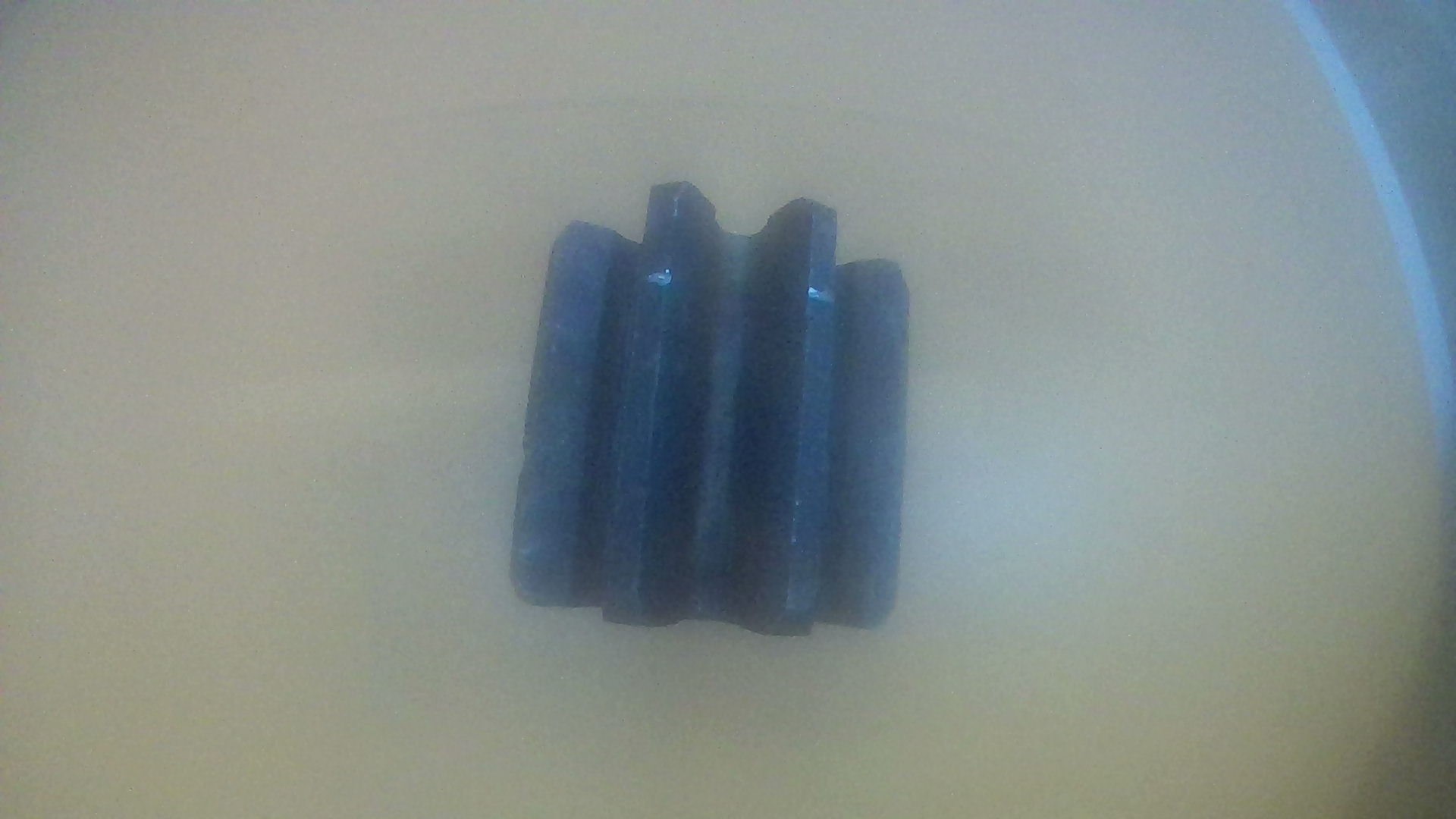

Supplement: S1 Data — (ZIP) [file pone.0322217.s001.zip › dataset/1/WIN_20250112_14_40_14_Pro.jpg]

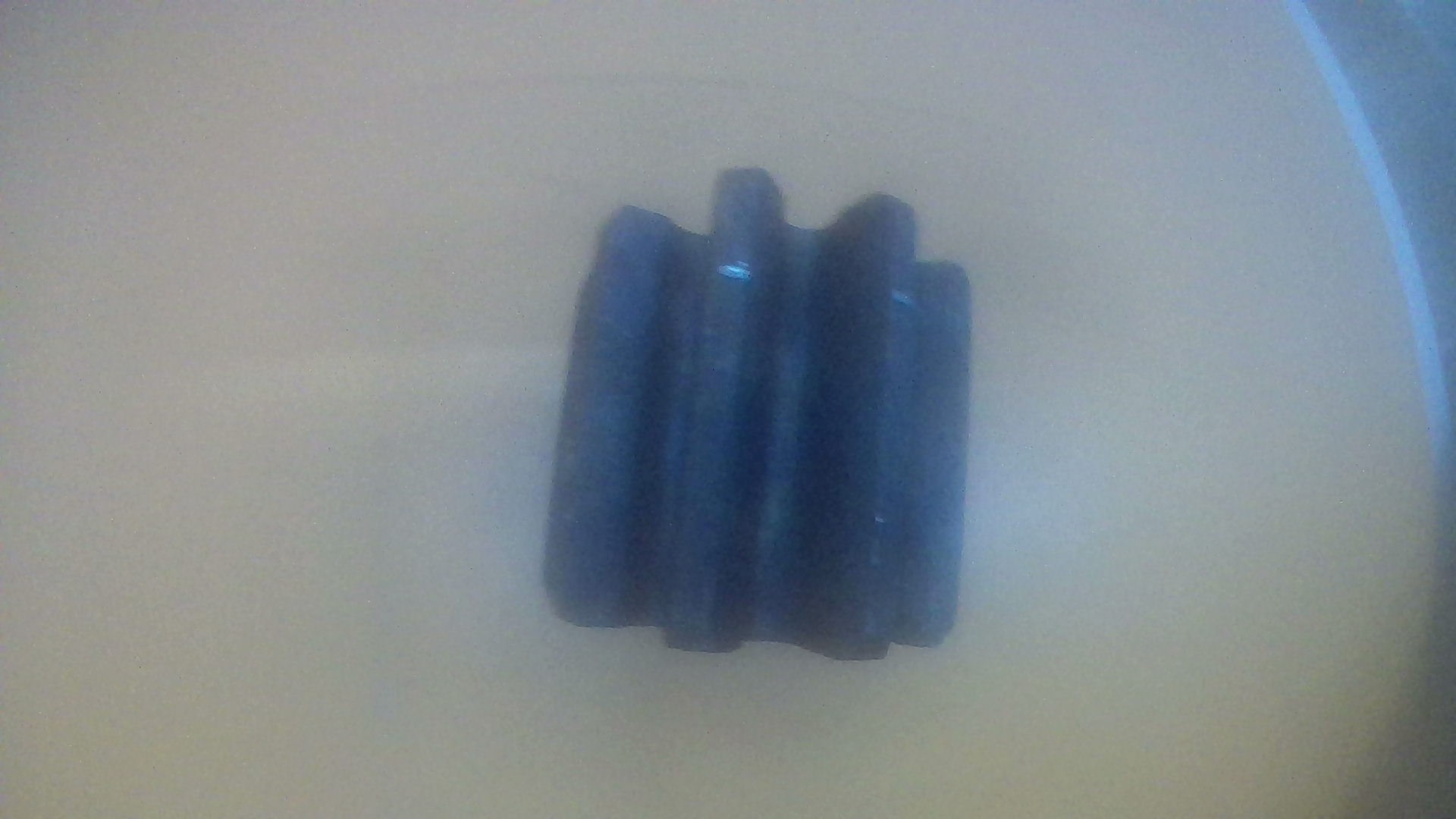

Supplement: S1 Data — (ZIP) [file pone.0322217.s001.zip › dataset/1/WIN_20250112_14_40_16_Pro.jpg]

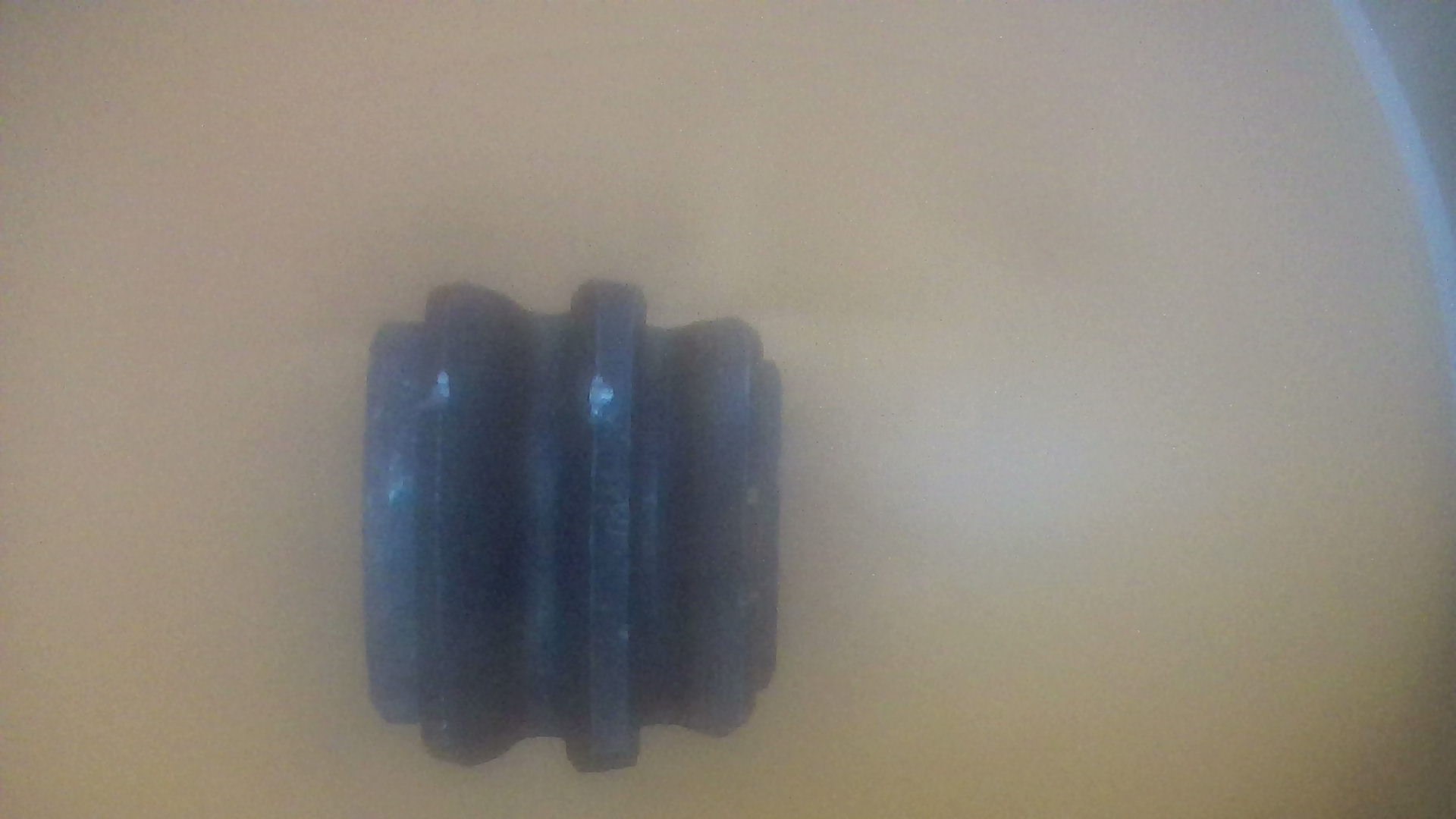

Supplement: S1 Data — (ZIP) [file pone.0322217.s001.zip › dataset/1/WIN_20250112_14_40_22_Pro.jpg]

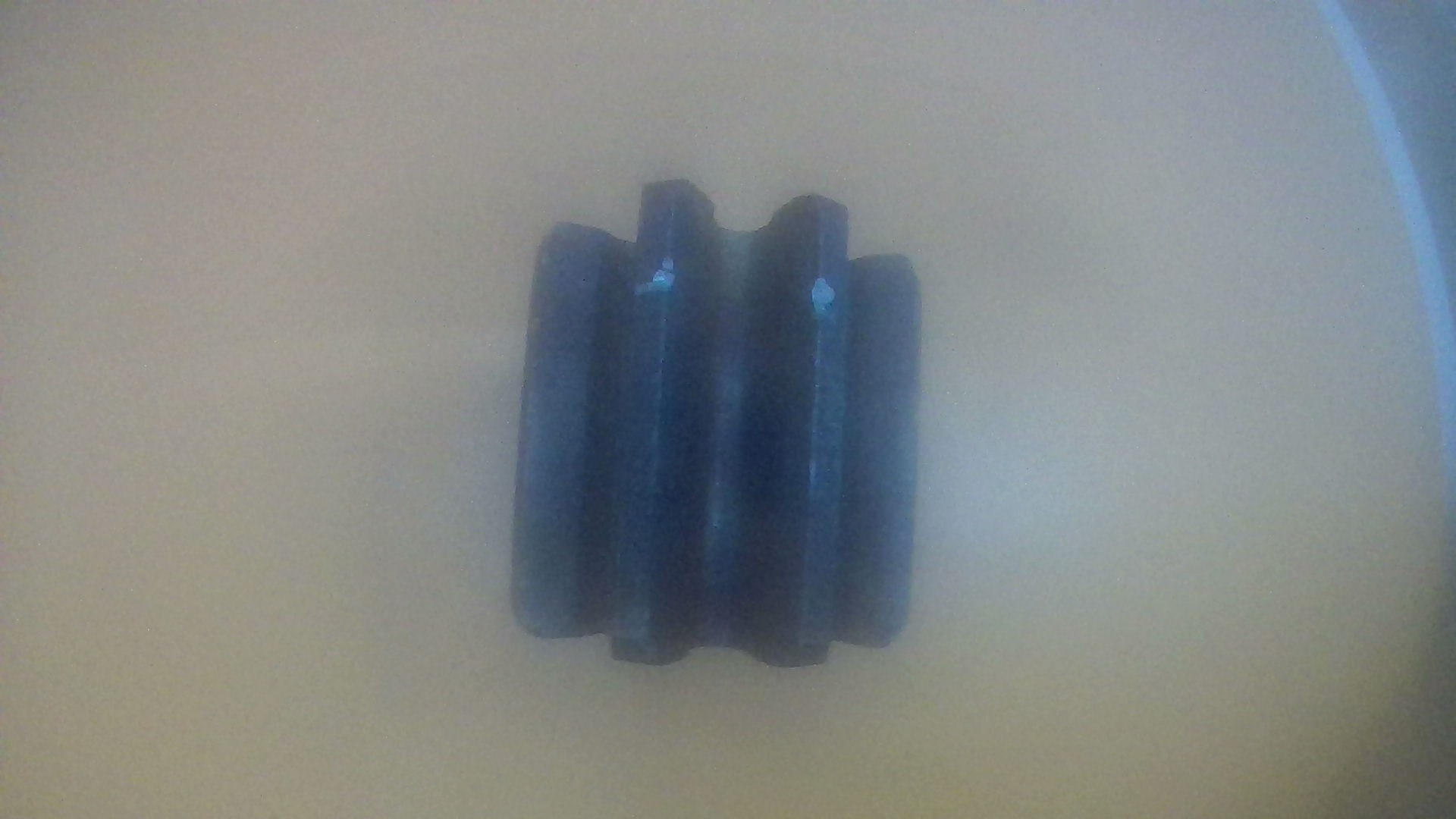

Supplement: S1 Data — (ZIP) [file pone.0322217.s001.zip › dataset/1/WIN_20250112_14_40_23_Pro.jpg]

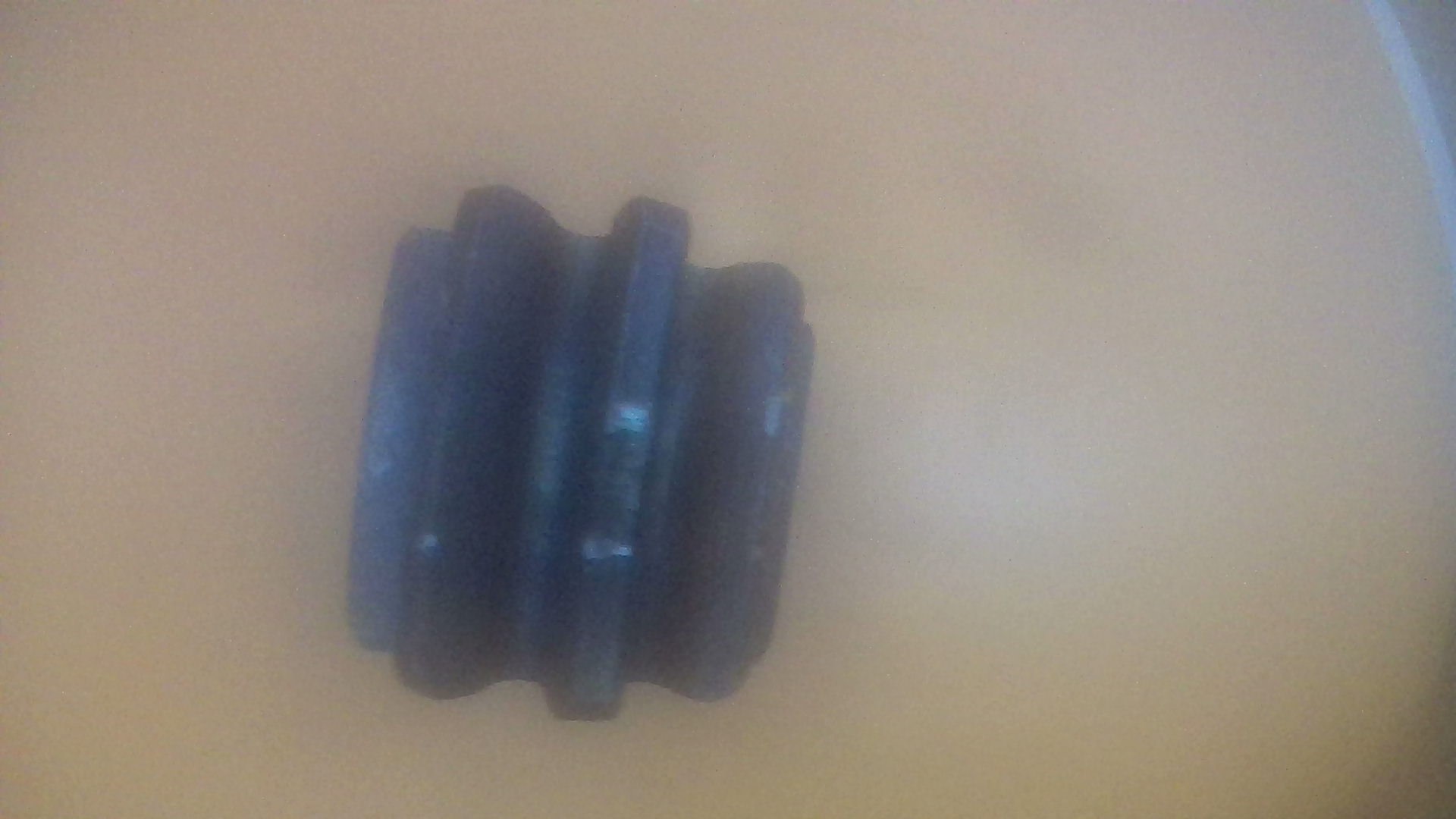

Supplement: S1 Data — (ZIP) [file pone.0322217.s001.zip › dataset/1/WIN_20250112_14_40_29_Pro.jpg]

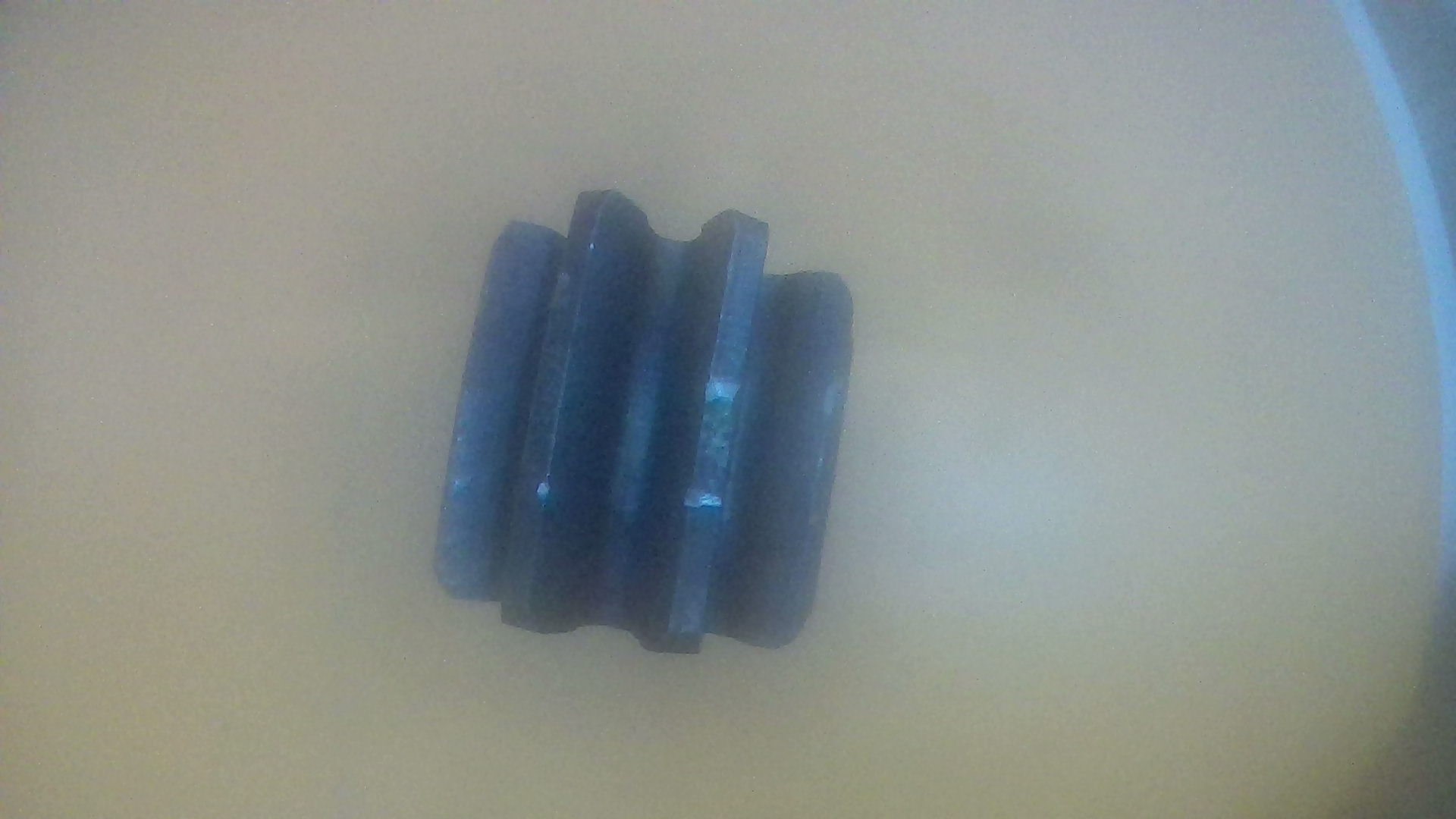

Supplement: S1 Data — (ZIP) [file pone.0322217.s001.zip › dataset/1/WIN_20250112_14_40_30_Pro.jpg]

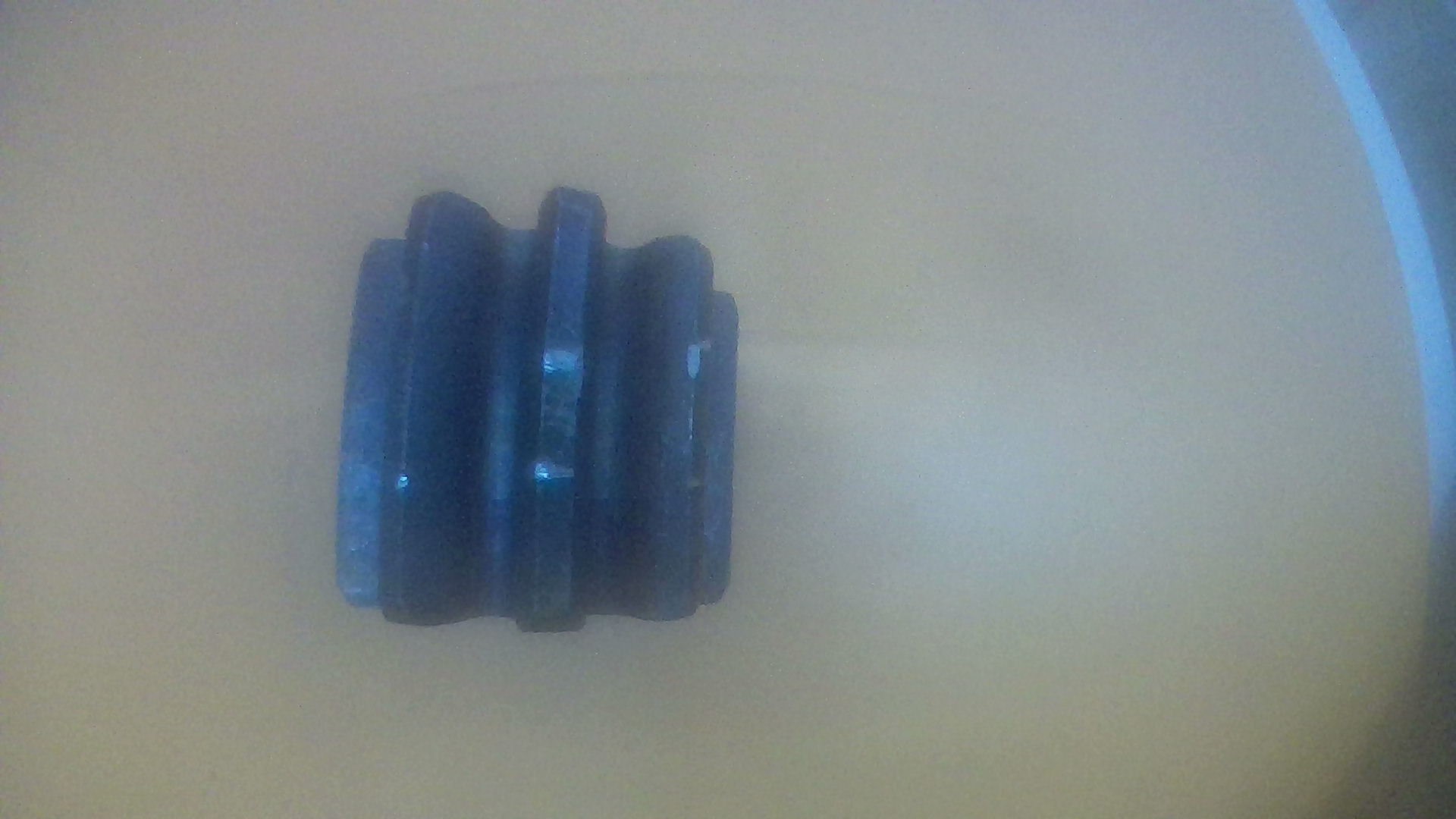

Supplement: S1 Data — (ZIP) [file pone.0322217.s001.zip › dataset/1/WIN_20250112_14_40_32_Pro.jpg]

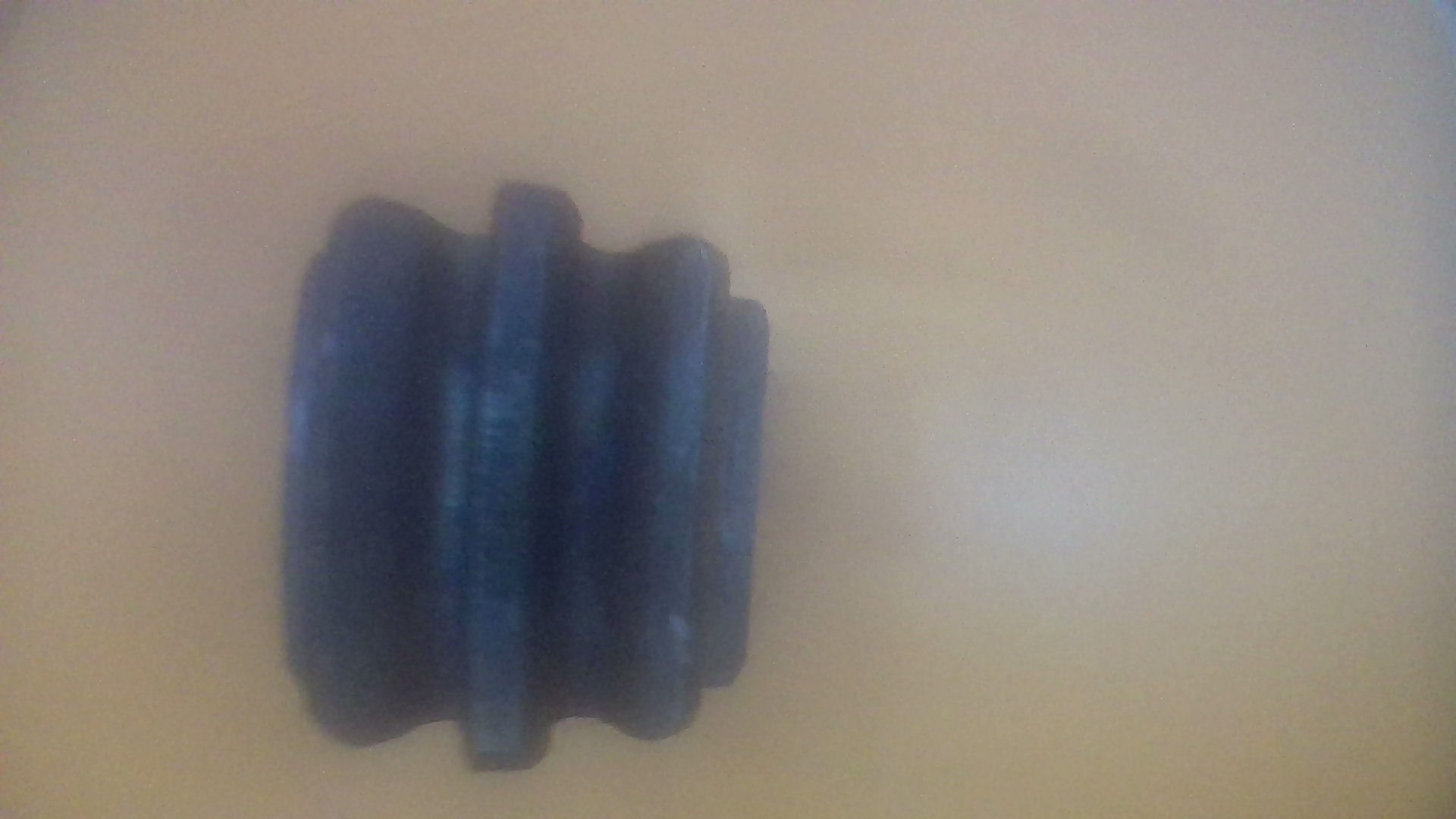

Supplement: S1 Data — (ZIP) [file pone.0322217.s001.zip › dataset/1/WIN_20250112_14_40_38_Pro.jpg]

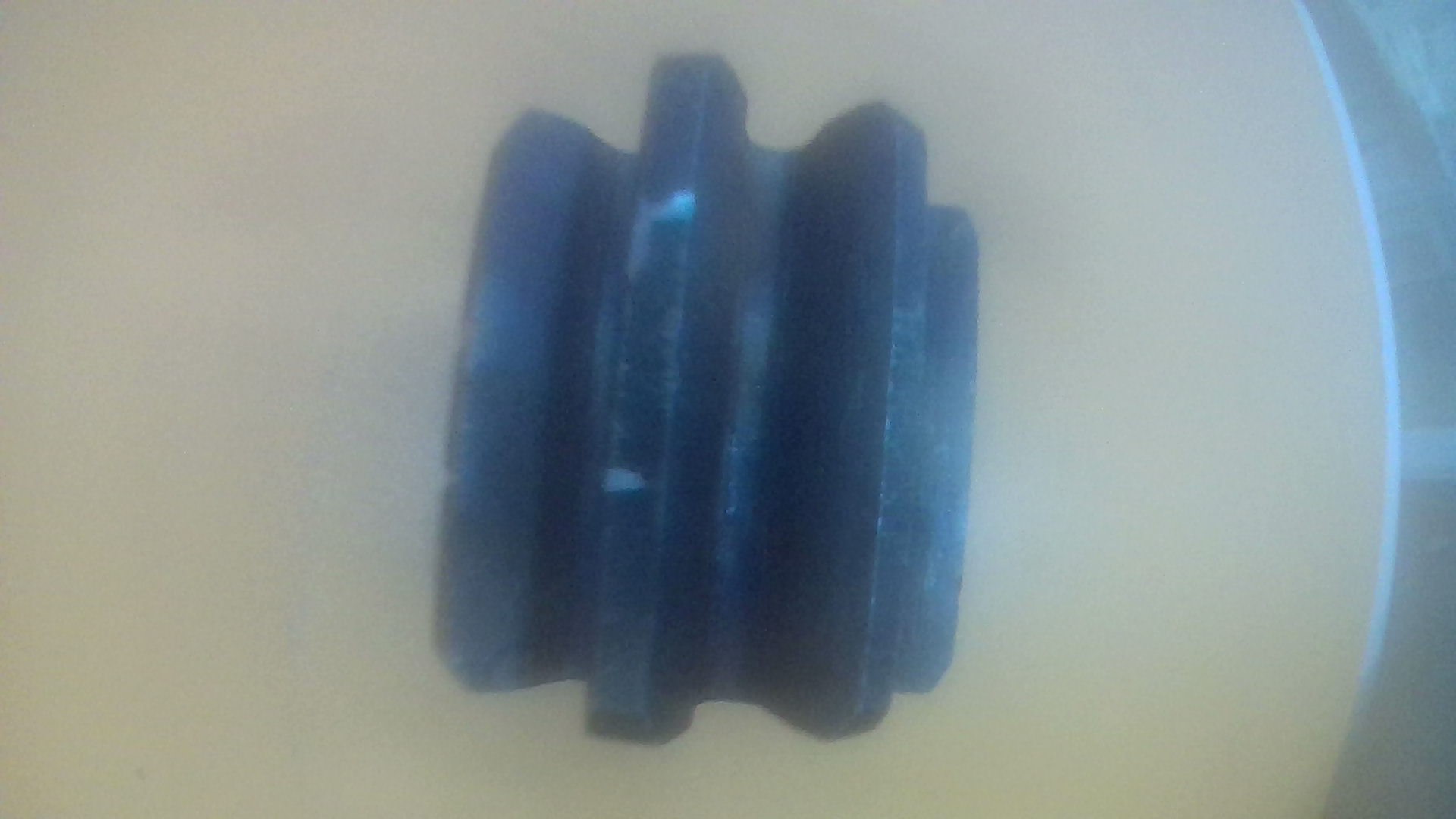

Supplement: S1 Data — (ZIP) [file pone.0322217.s001.zip › dataset/1/WIN_20250112_14_40_40_Pro.jpg]

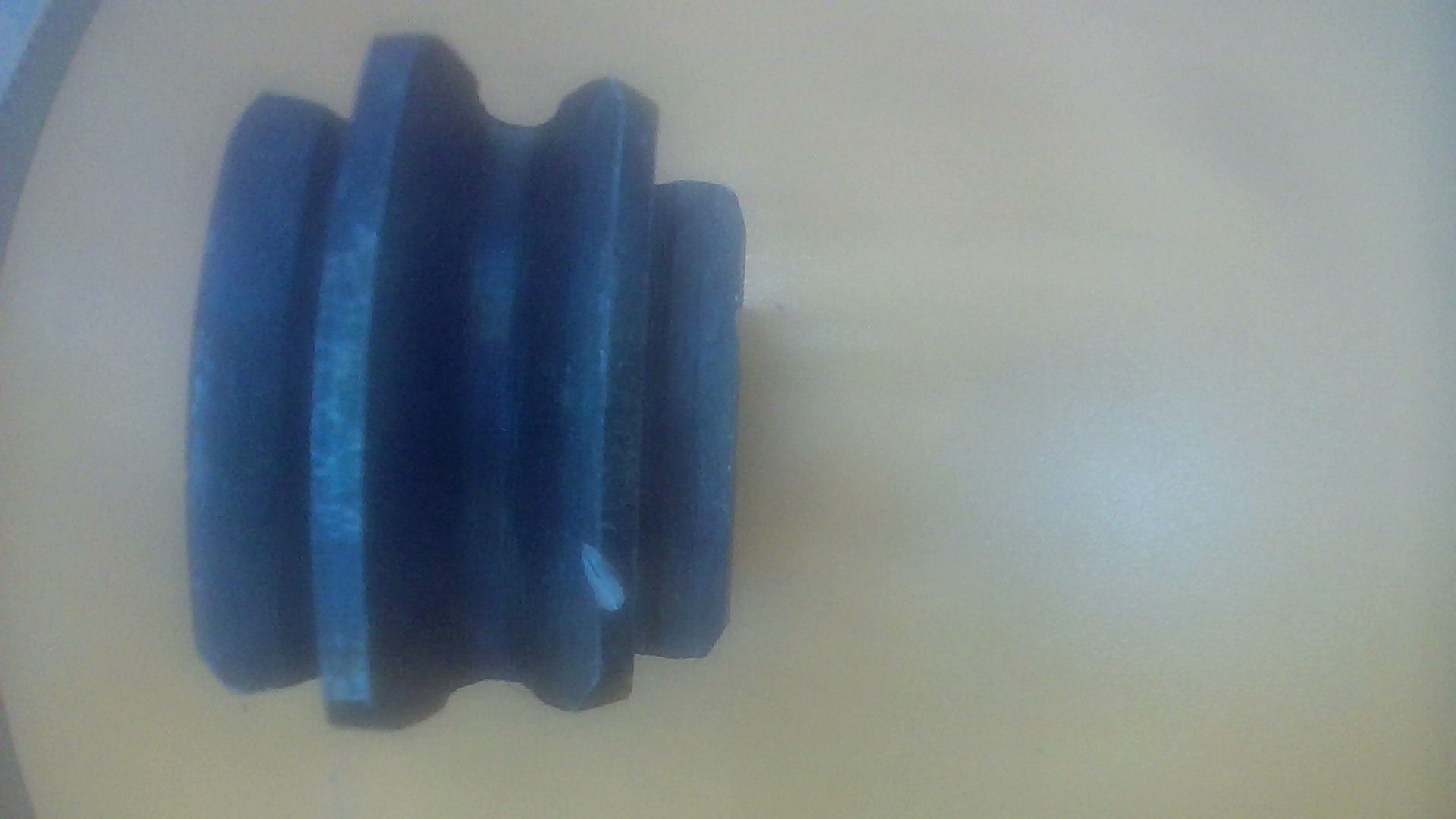

Supplement: S1 Data — (ZIP) [file pone.0322217.s001.zip › dataset/1/WIN_20250112_14_40_42_Pro.jpg]

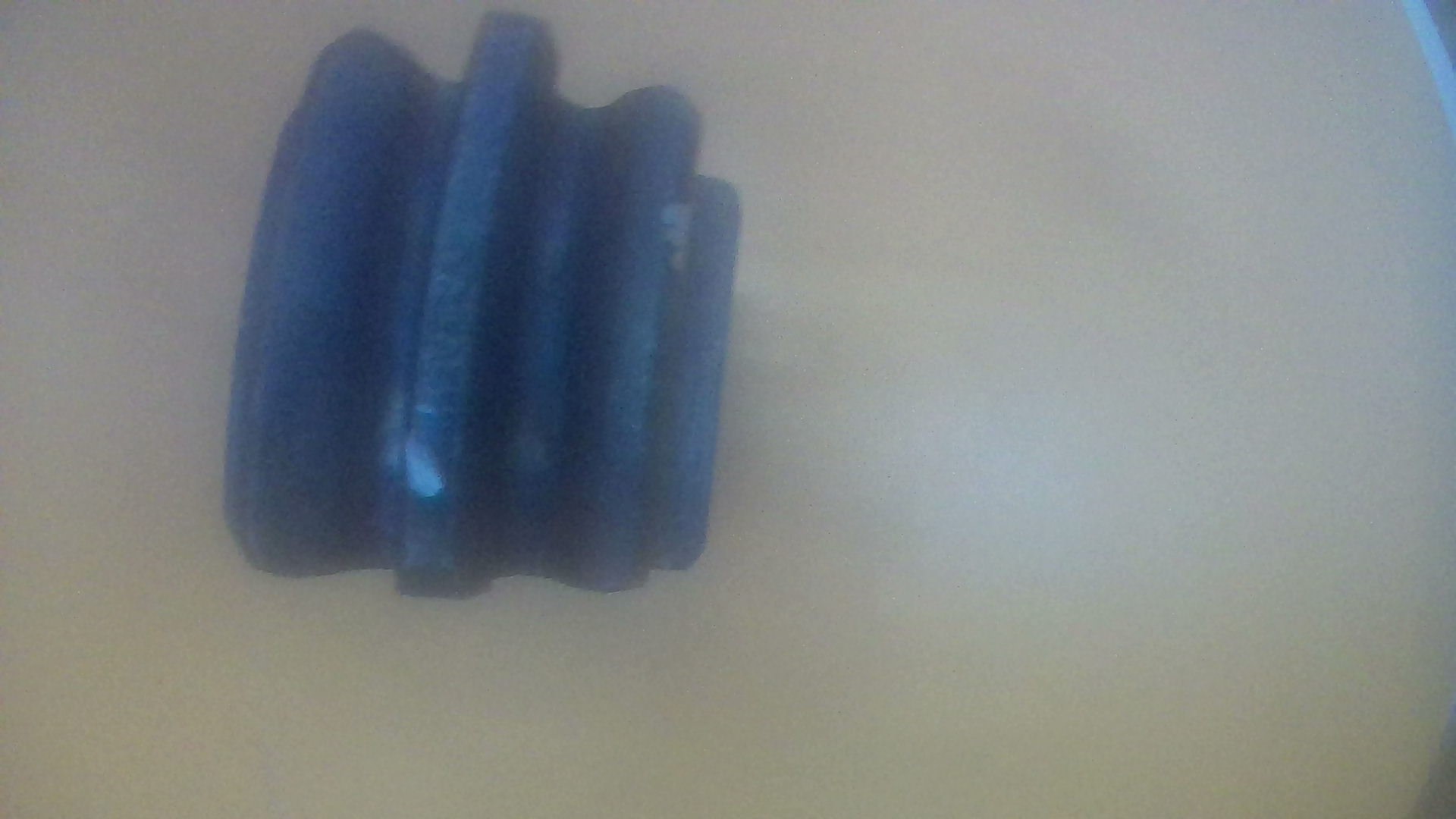

Supplement: S1 Data — (ZIP) [file pone.0322217.s001.zip › dataset/1/WIN_20250112_14_40_49_Pro.jpg]

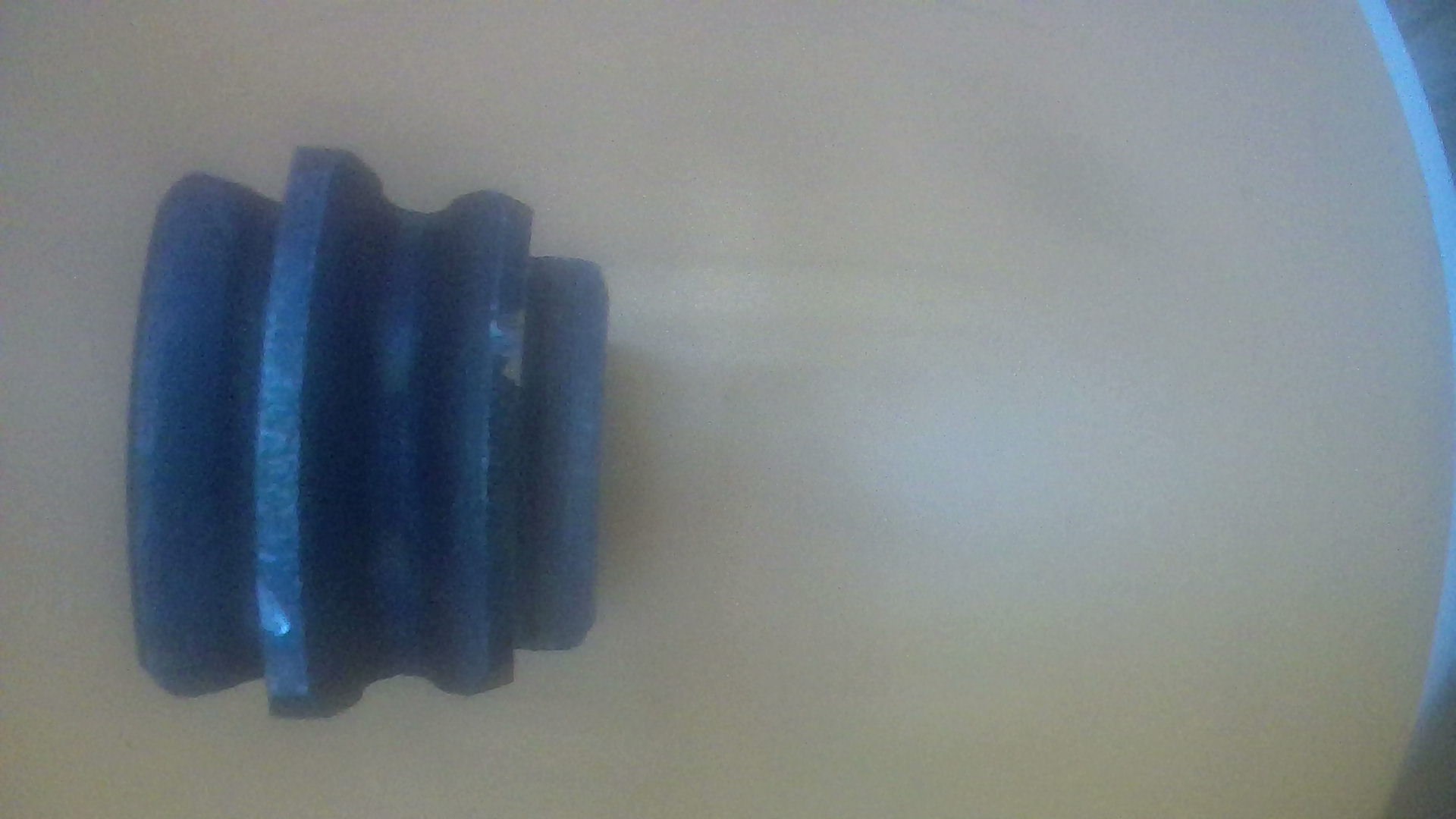

Supplement: S1 Data — (ZIP) [file pone.0322217.s001.zip › dataset/1/WIN_20250112_14_40_52_Pro.jpg]

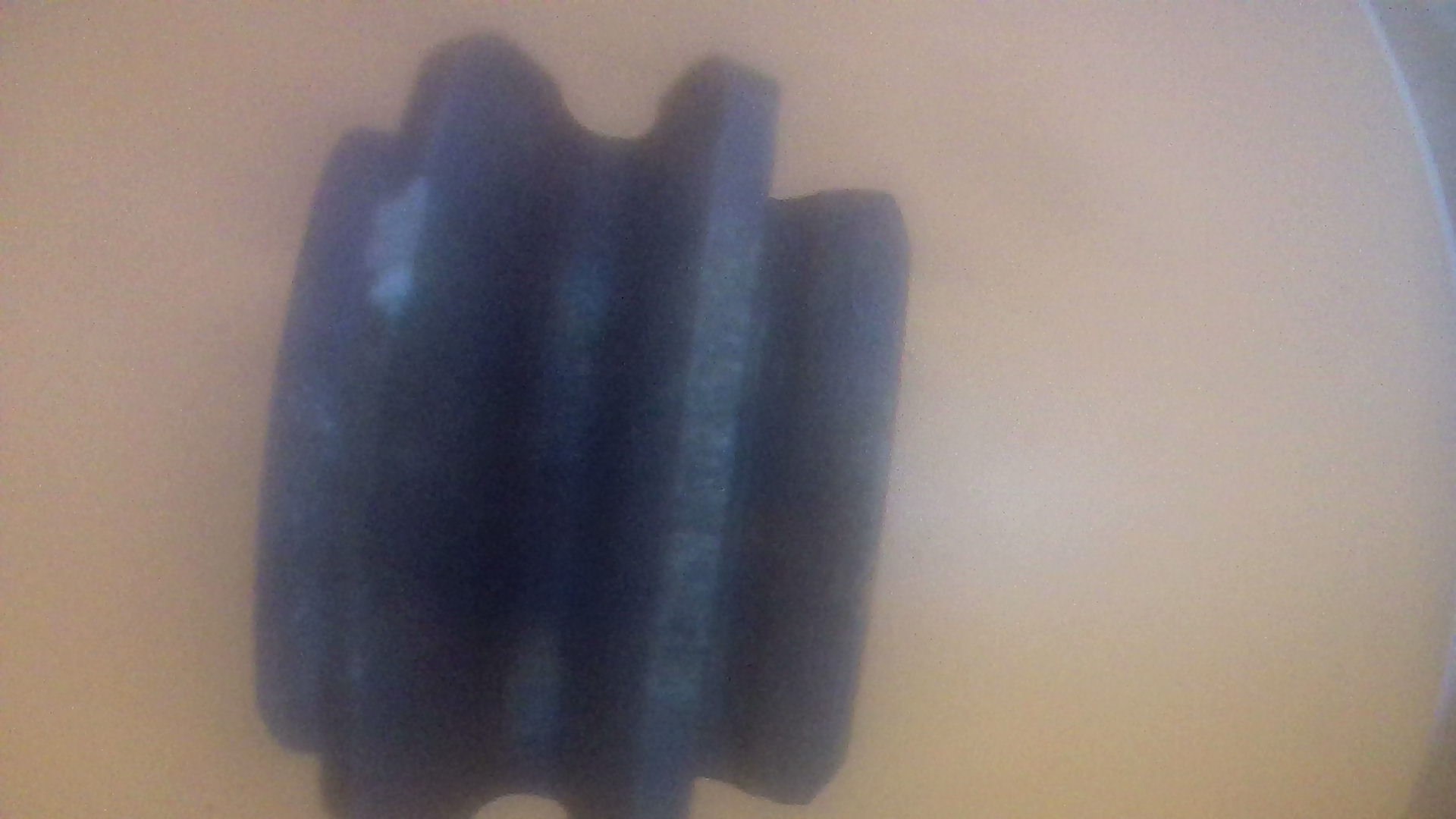

Supplement: S1 Data — (ZIP) [file pone.0322217.s001.zip › dataset/1/WIN_20250112_14_40_58_Pro.jpg]

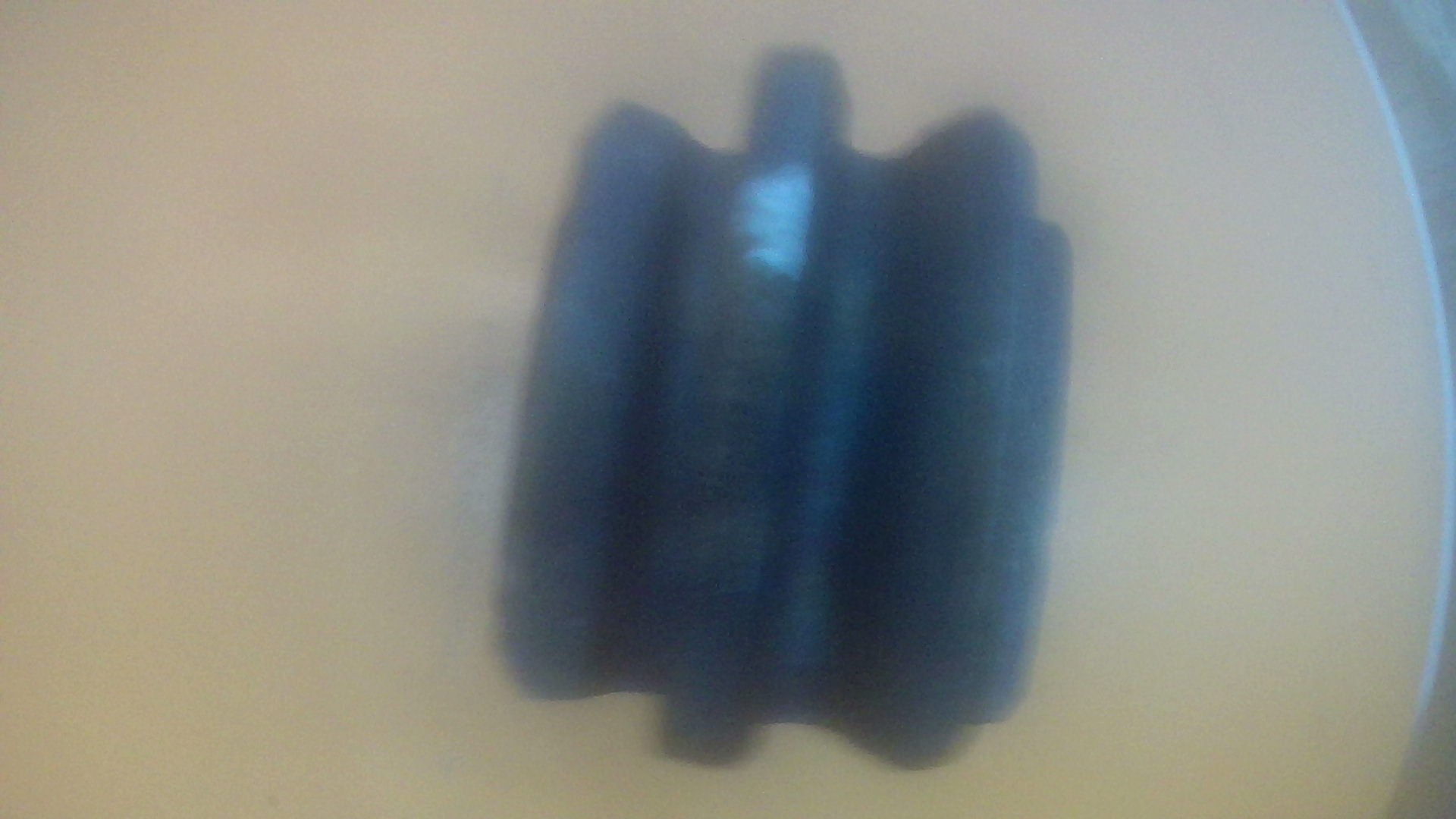

Supplement: S1 Data — (ZIP) [file pone.0322217.s001.zip › dataset/1/WIN_20250112_14_40_59_Pro.jpg]

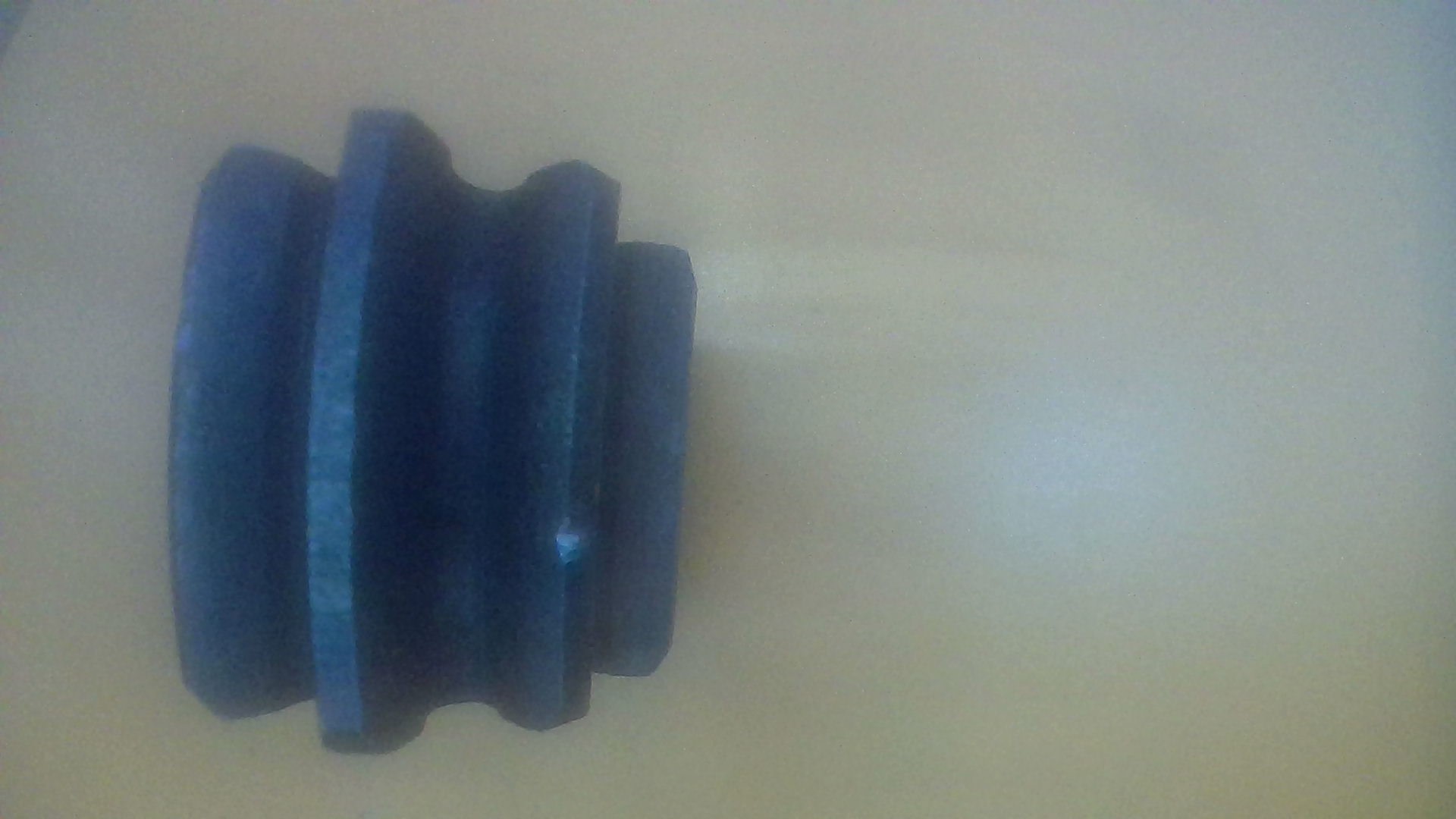

Supplement: S1 Data — (ZIP) [file pone.0322217.s001.zip › dataset/1/WIN_20250112_14_41_01_Pro.jpg]

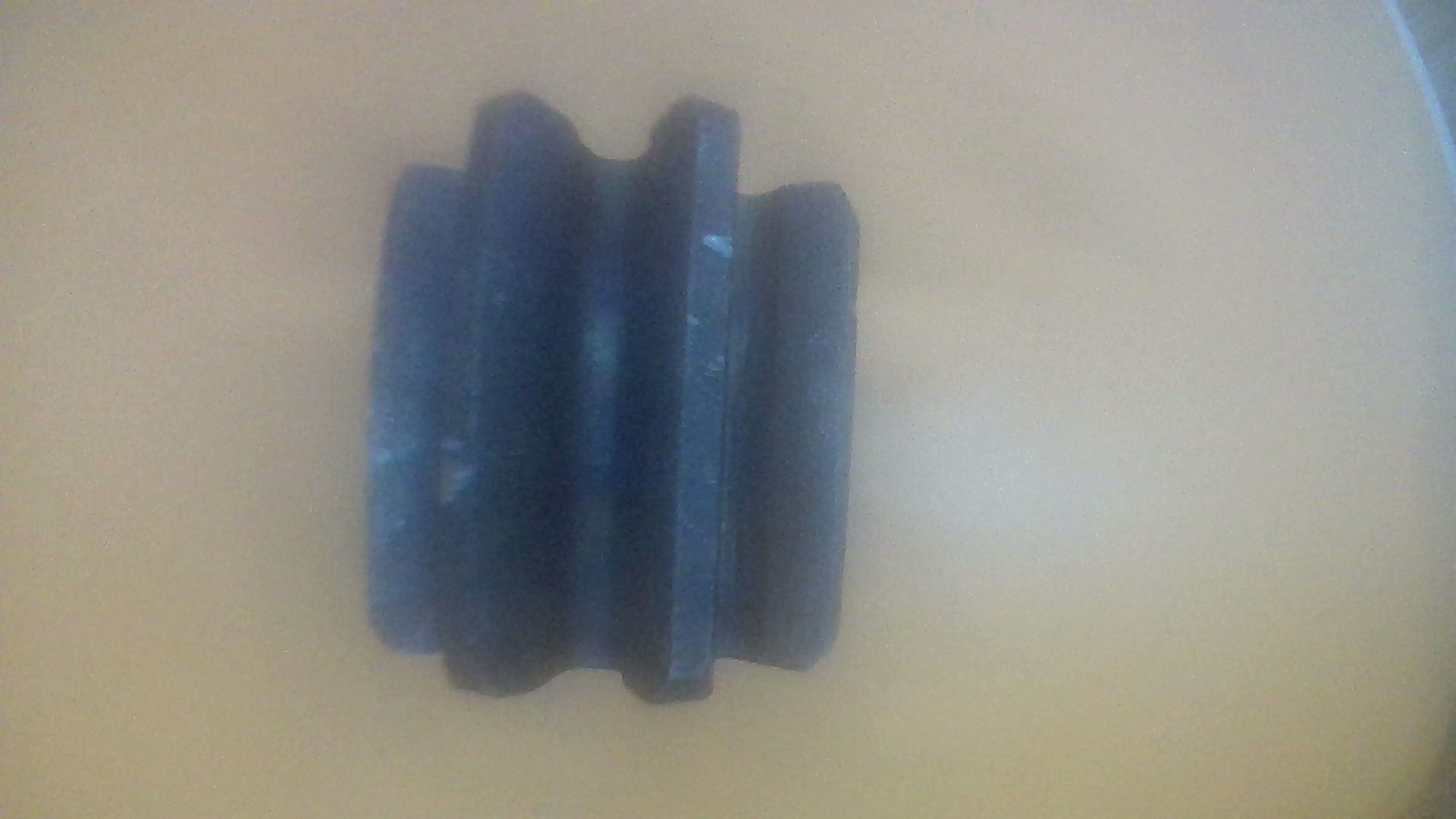

Supplement: S1 Data — (ZIP) [file pone.0322217.s001.zip › dataset/1/WIN_20250112_14_41_08_Pro.jpg]

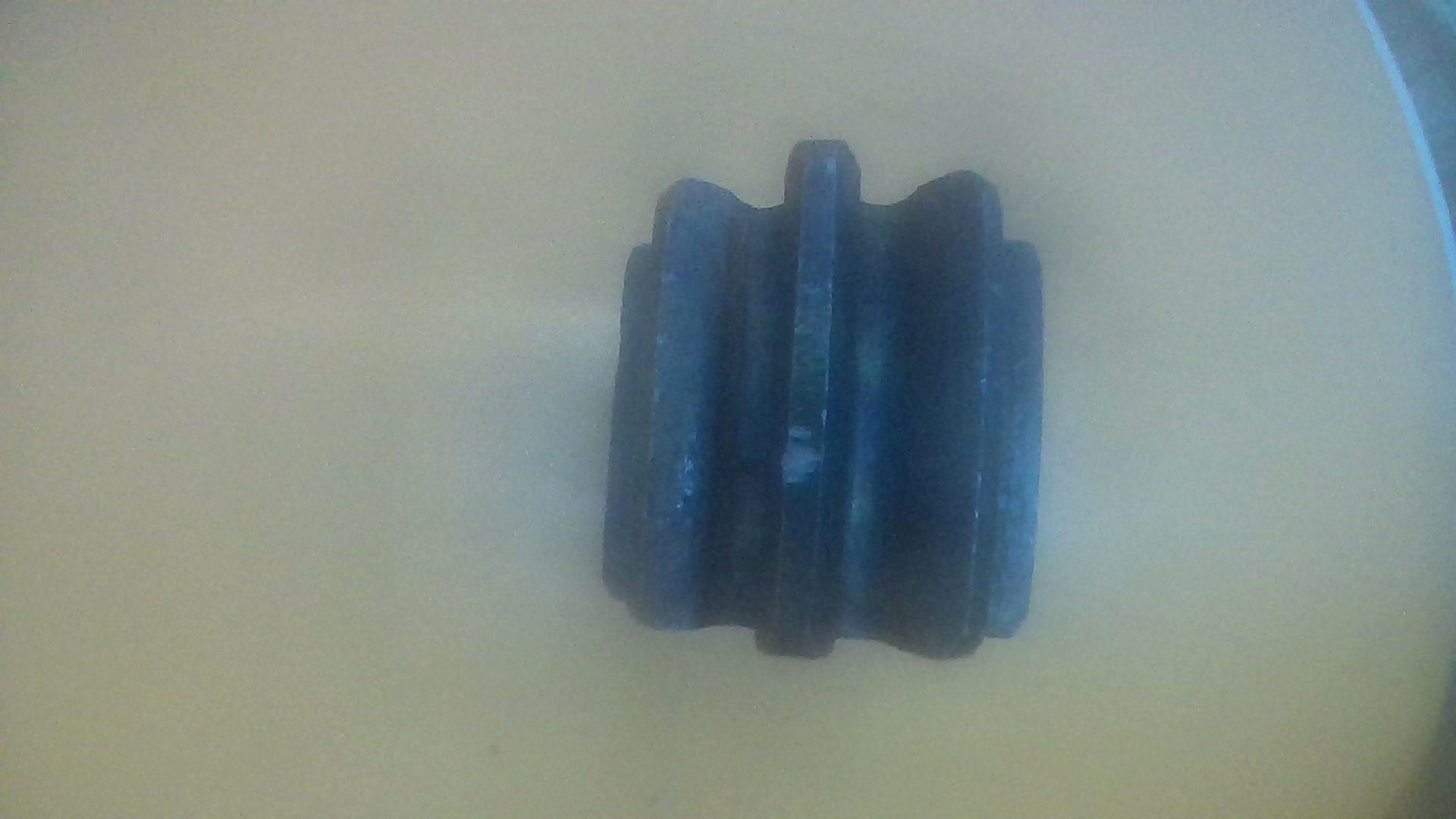

Supplement: S1 Data — (ZIP) [file pone.0322217.s001.zip › dataset/1/WIN_20250112_14_41_09_Pro.jpg]

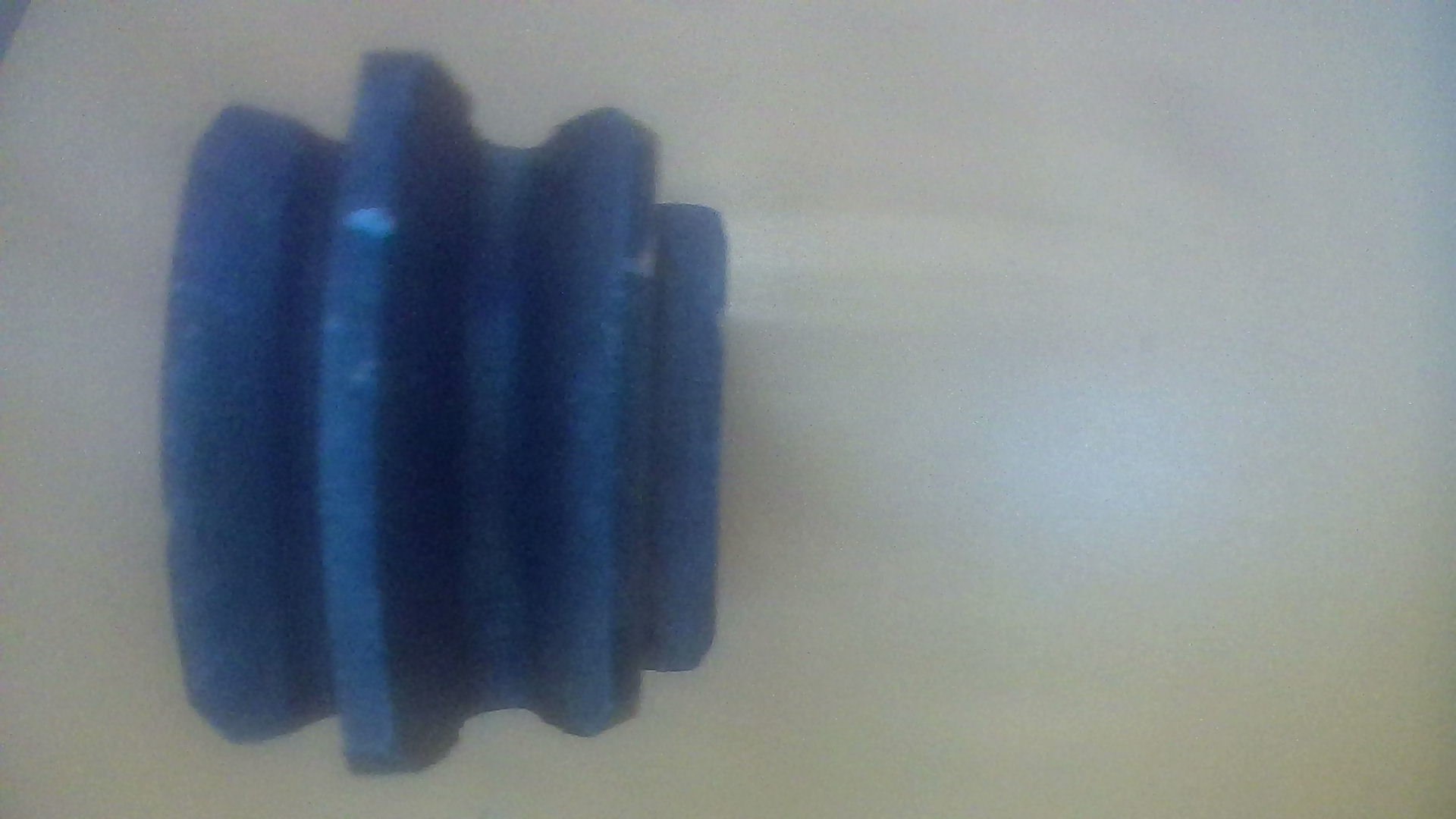

Supplement: S1 Data — (ZIP) [file pone.0322217.s001.zip › dataset/1/WIN_20250112_14_41_11_Pro.jpg]

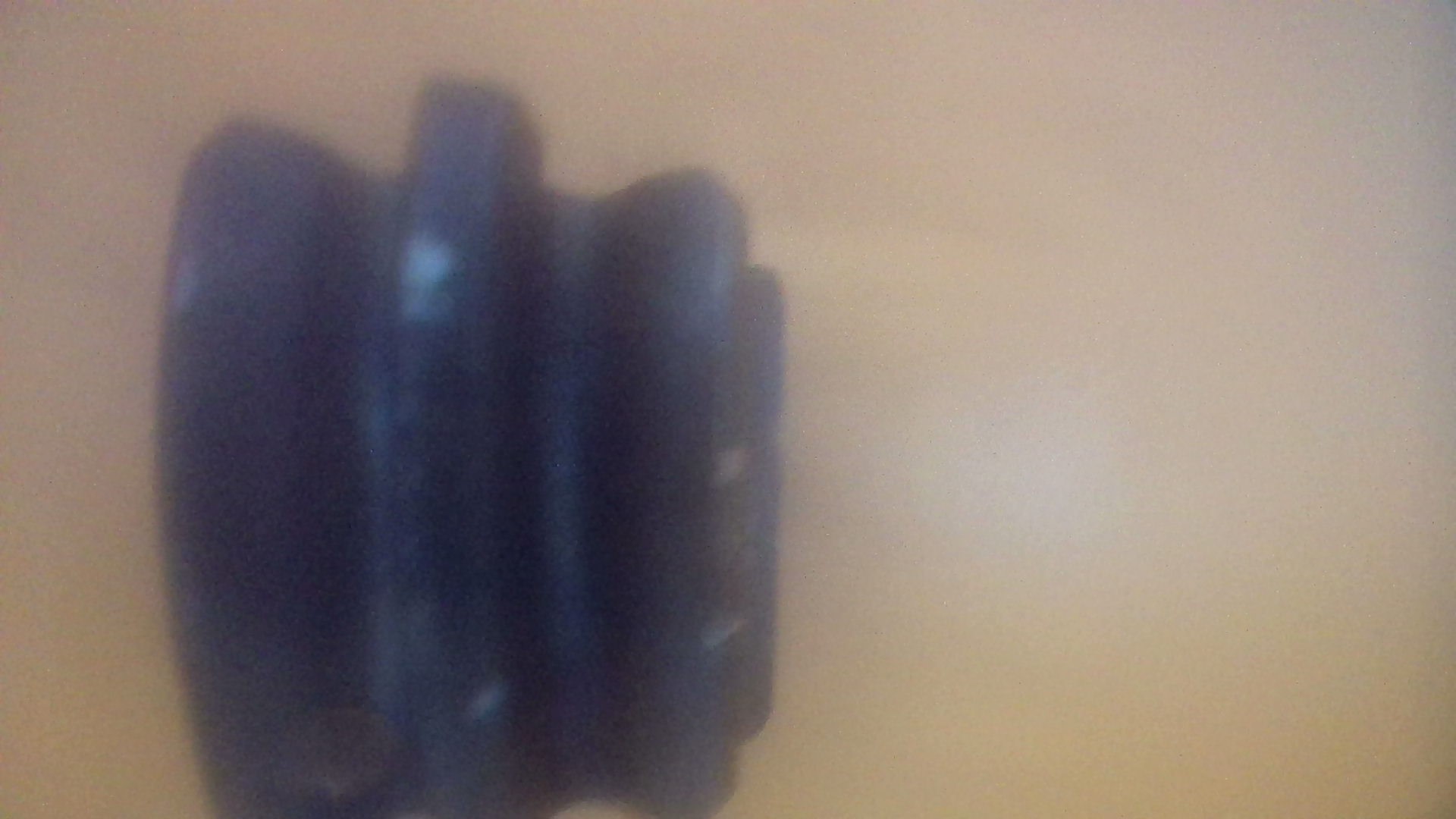

Supplement: S1 Data — (ZIP) [file pone.0322217.s001.zip › dataset/1/WIN_20250112_14_41_17_Pro.jpg]

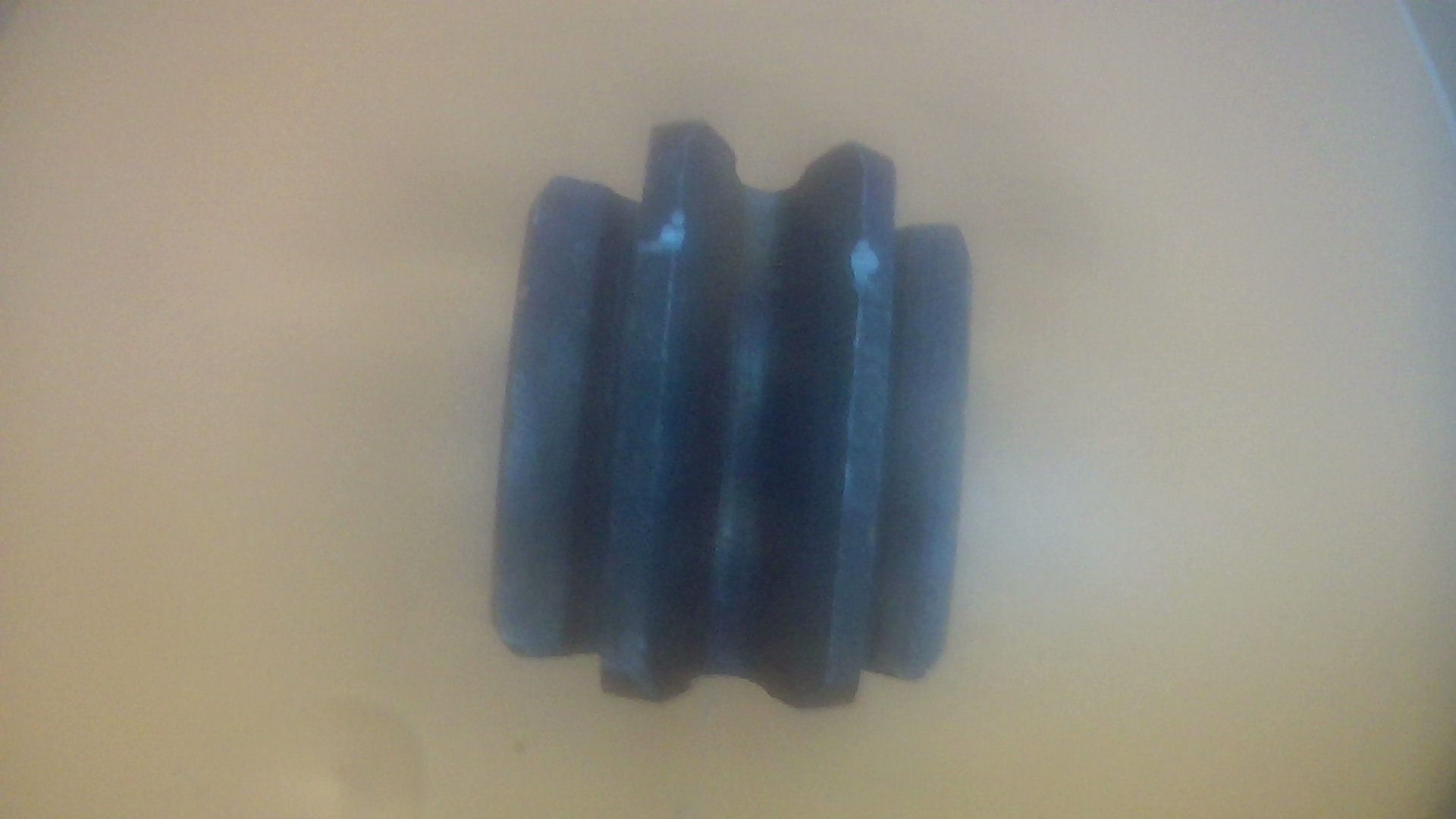

Supplement: S1 Data — (ZIP) [file pone.0322217.s001.zip › dataset/1/WIN_20250112_14_41_18_Pro.jpg]

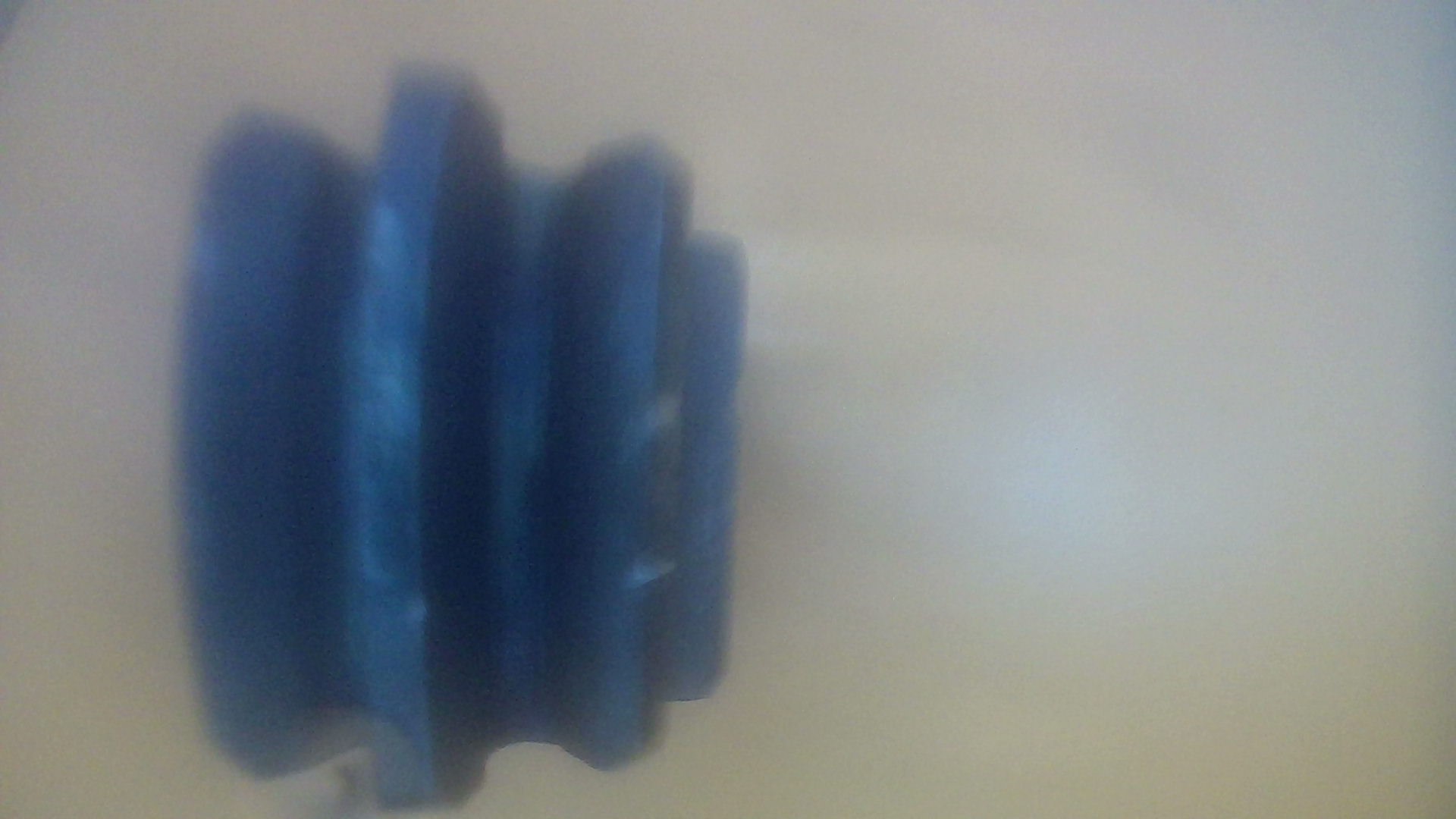

Supplement: S1 Data — (ZIP) [file pone.0322217.s001.zip › dataset/1/WIN_20250112_14_41_19_Pro.jpg]

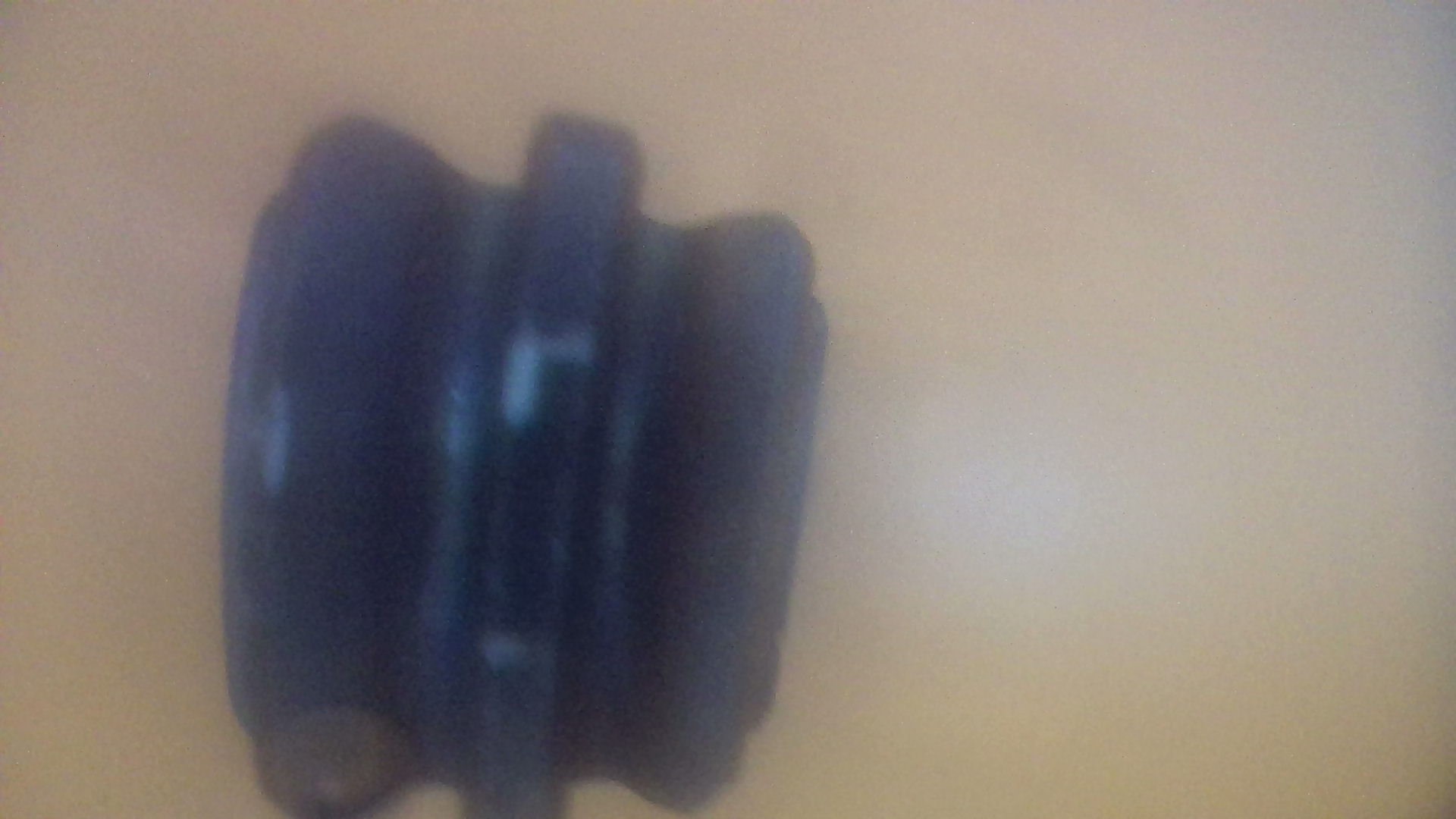

Supplement: S1 Data — (ZIP) [file pone.0322217.s001.zip › dataset/1/WIN_20250112_14_41_24_Pro.jpg]

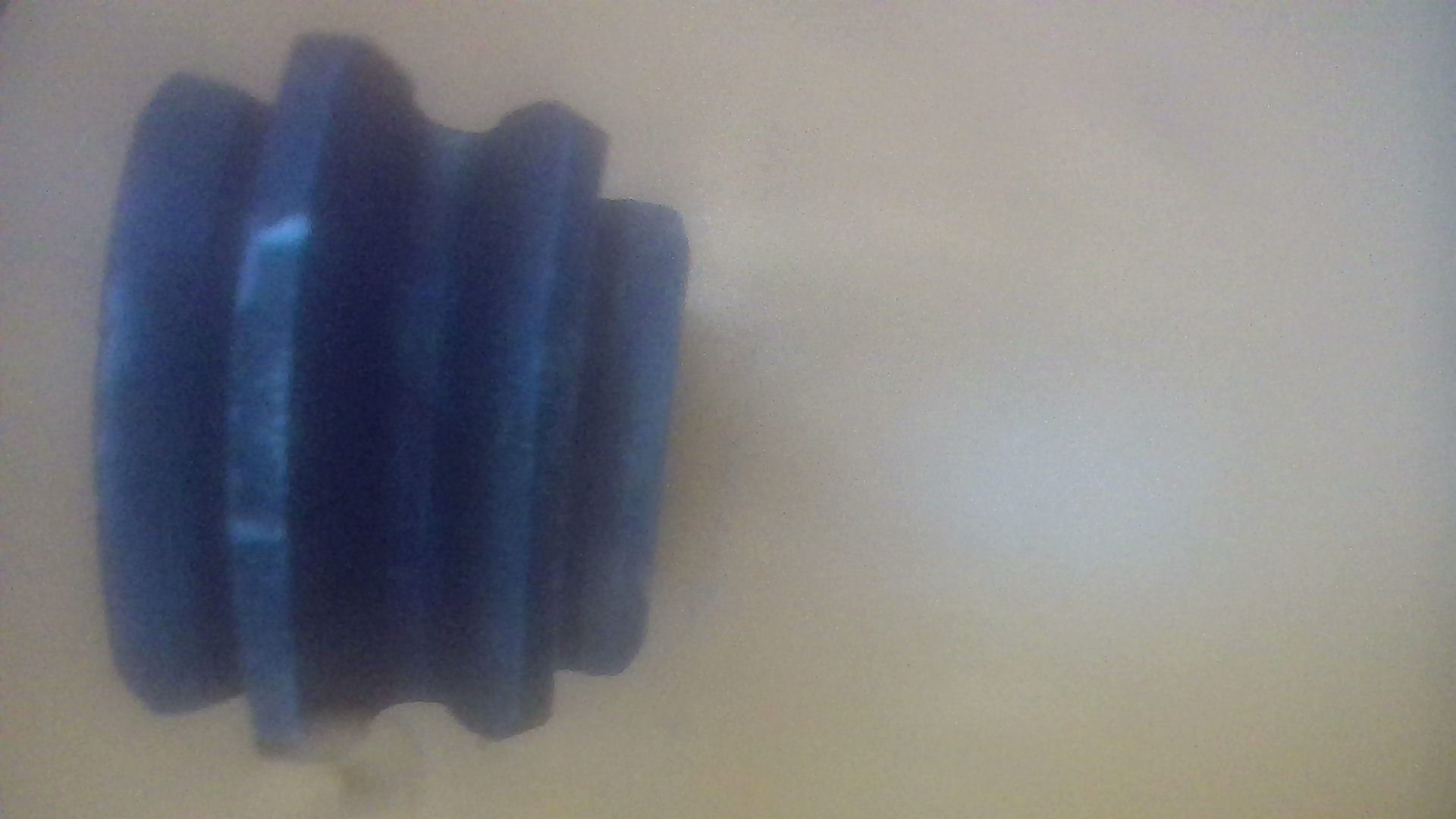

Supplement: S1 Data — (ZIP) [file pone.0322217.s001.zip › dataset/1/WIN_20250112_14_41_25_Pro.jpg]

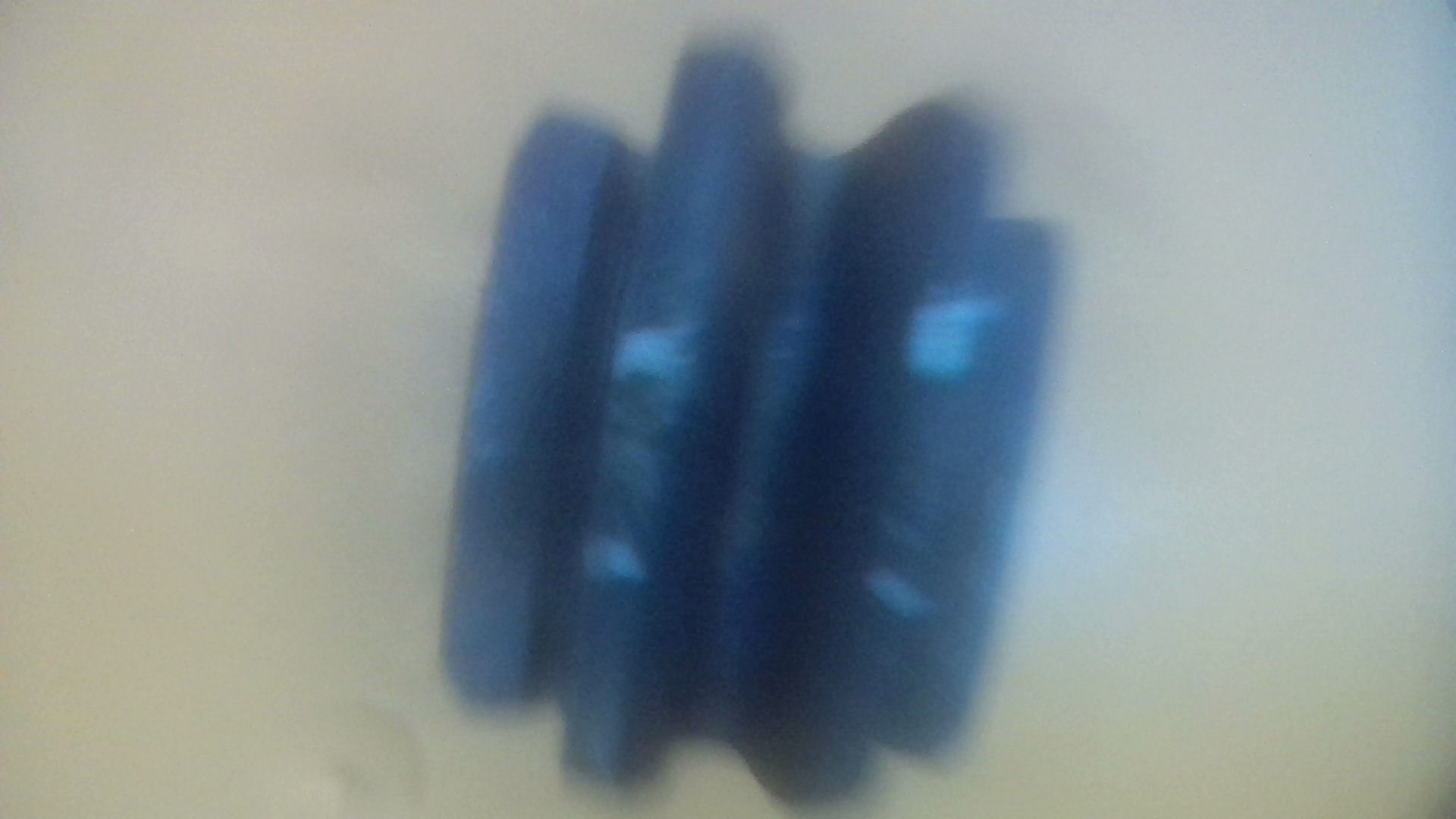

Supplement: S1 Data — (ZIP) [file pone.0322217.s001.zip › dataset/1/WIN_20250112_14_41_27_Pro.jpg]

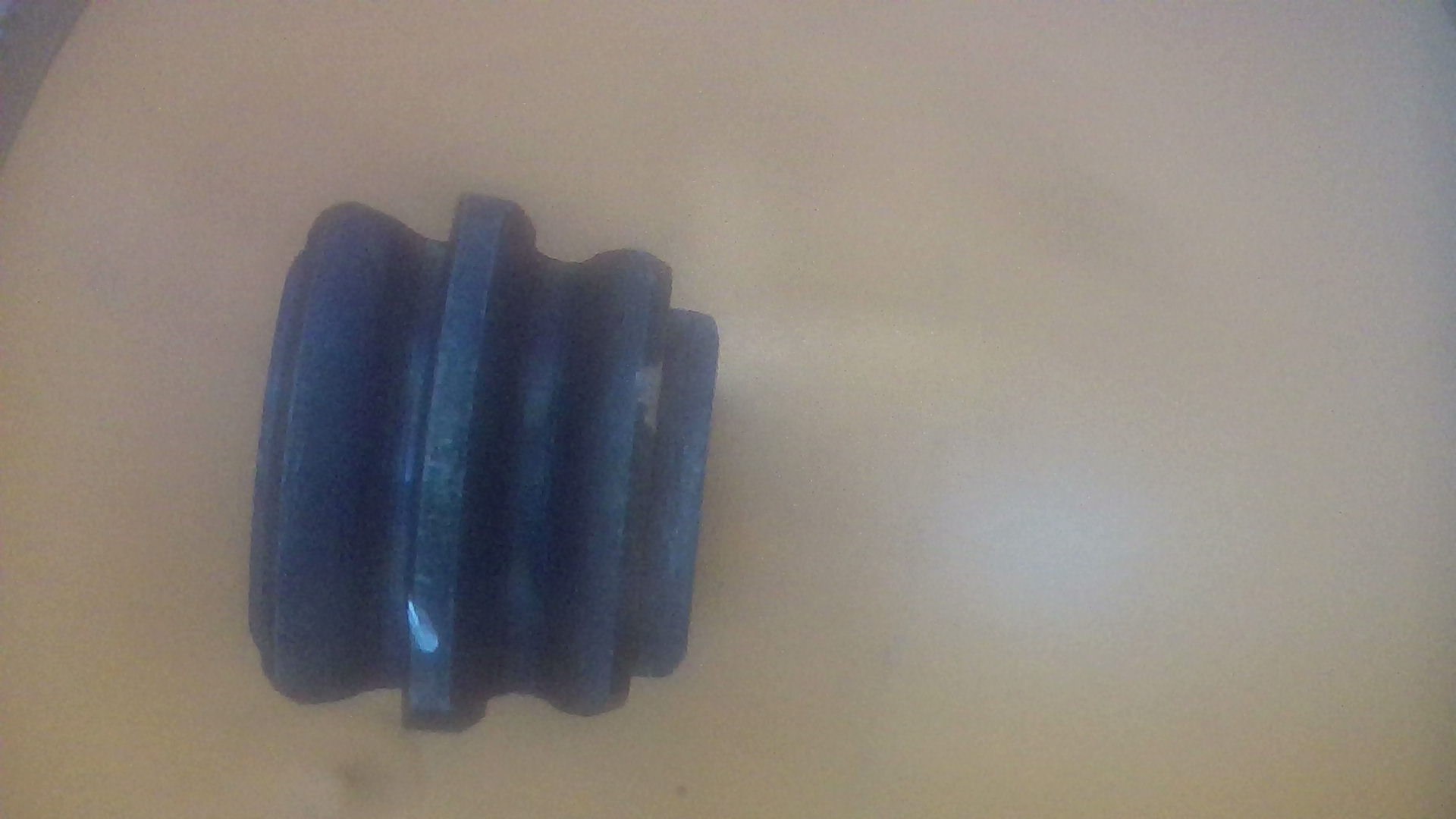

Supplement: S1 Data — (ZIP) [file pone.0322217.s001.zip › dataset/1/WIN_20250112_14_41_32_Pro.jpg]

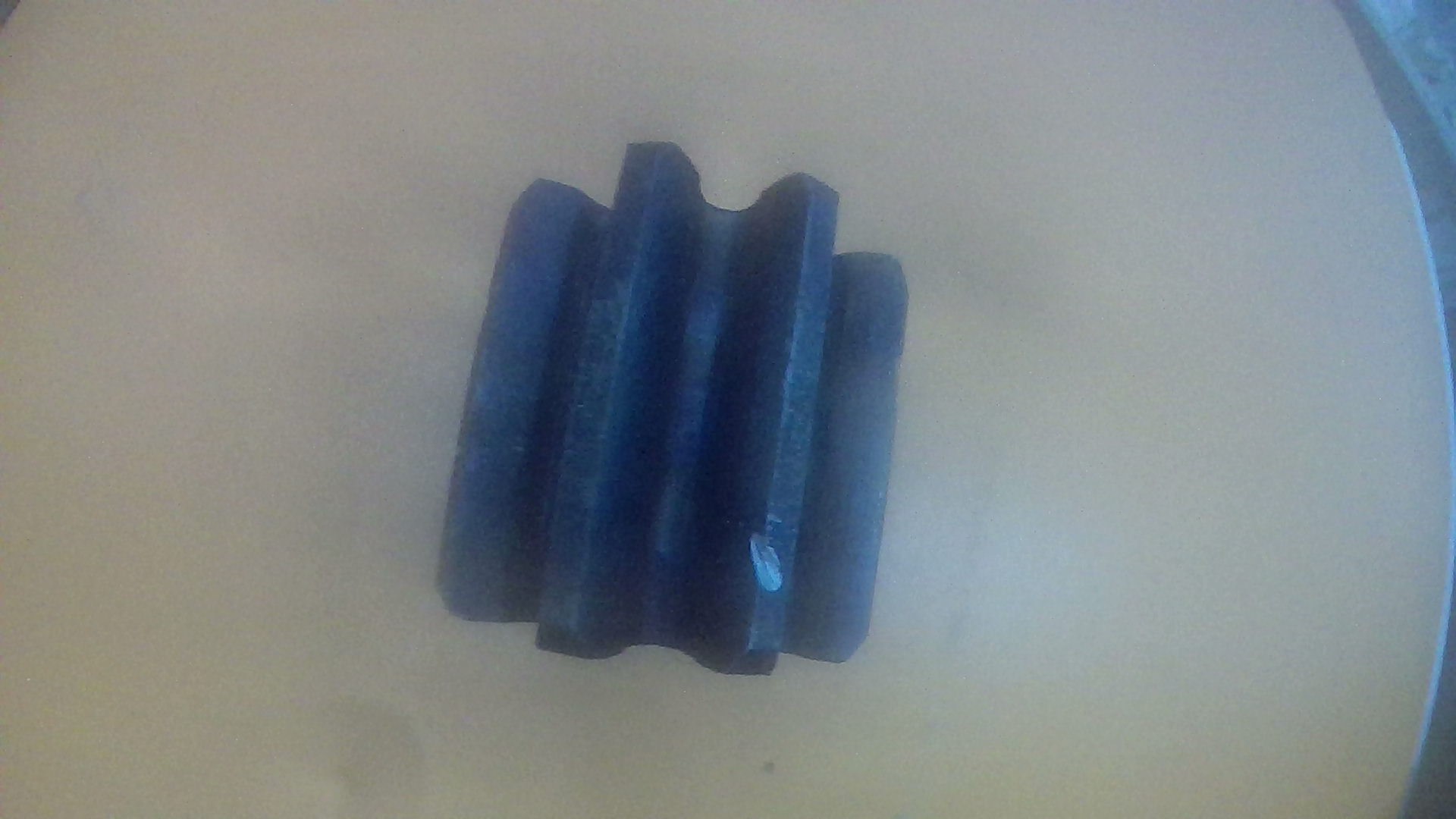

Supplement: S1 Data — (ZIP) [file pone.0322217.s001.zip › dataset/1/WIN_20250112_14_41_34_Pro.jpg]

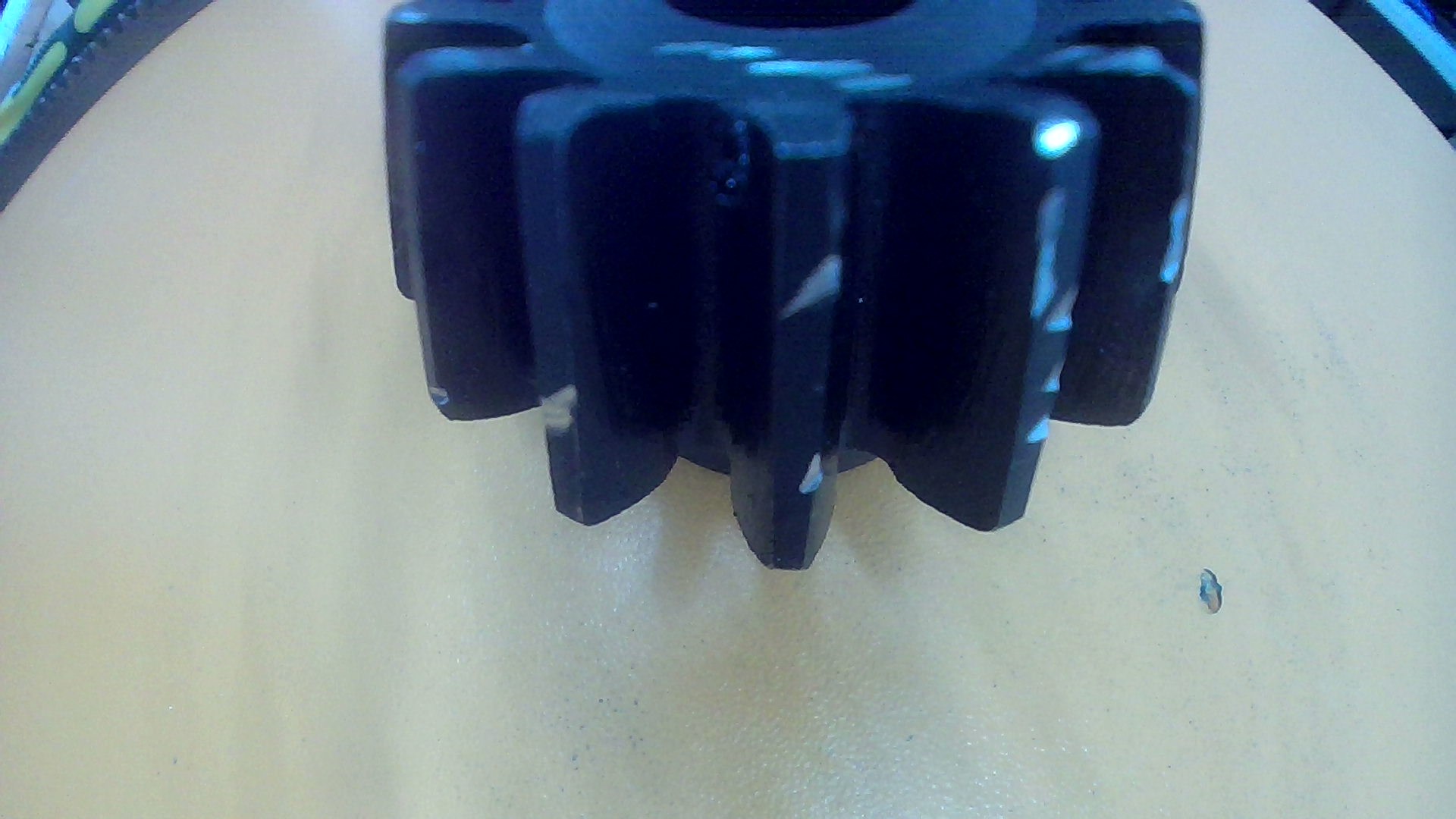

Supplement: S1 Data — (ZIP) [file pone.0322217.s001.zip › dataset/2/WIN_20250112_14_51_47_Pro.jpg]

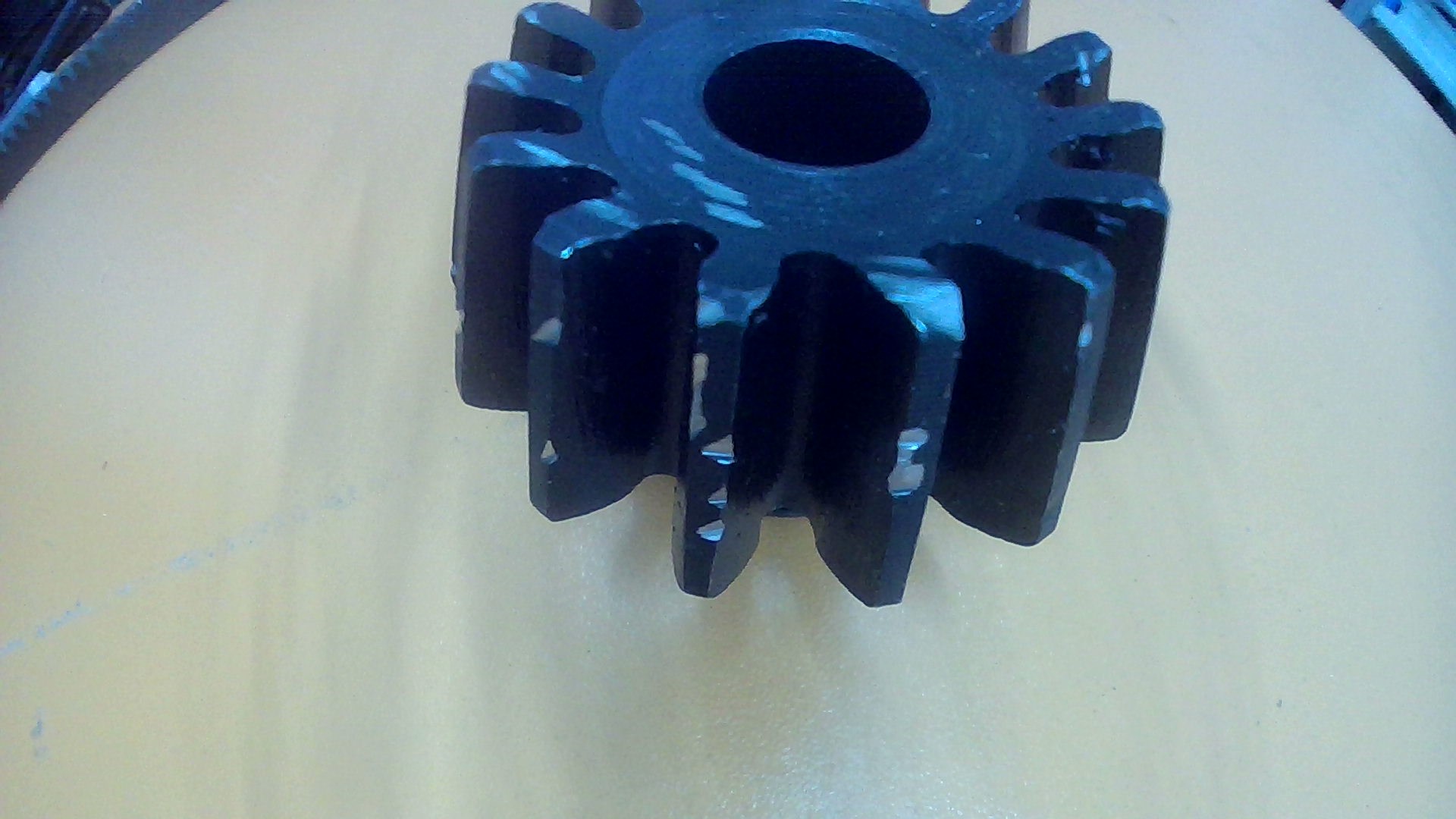

Supplement: S1 Data — (ZIP) [file pone.0322217.s001.zip › dataset/2/WIN_20250112_14_51_58_Pro.jpg]

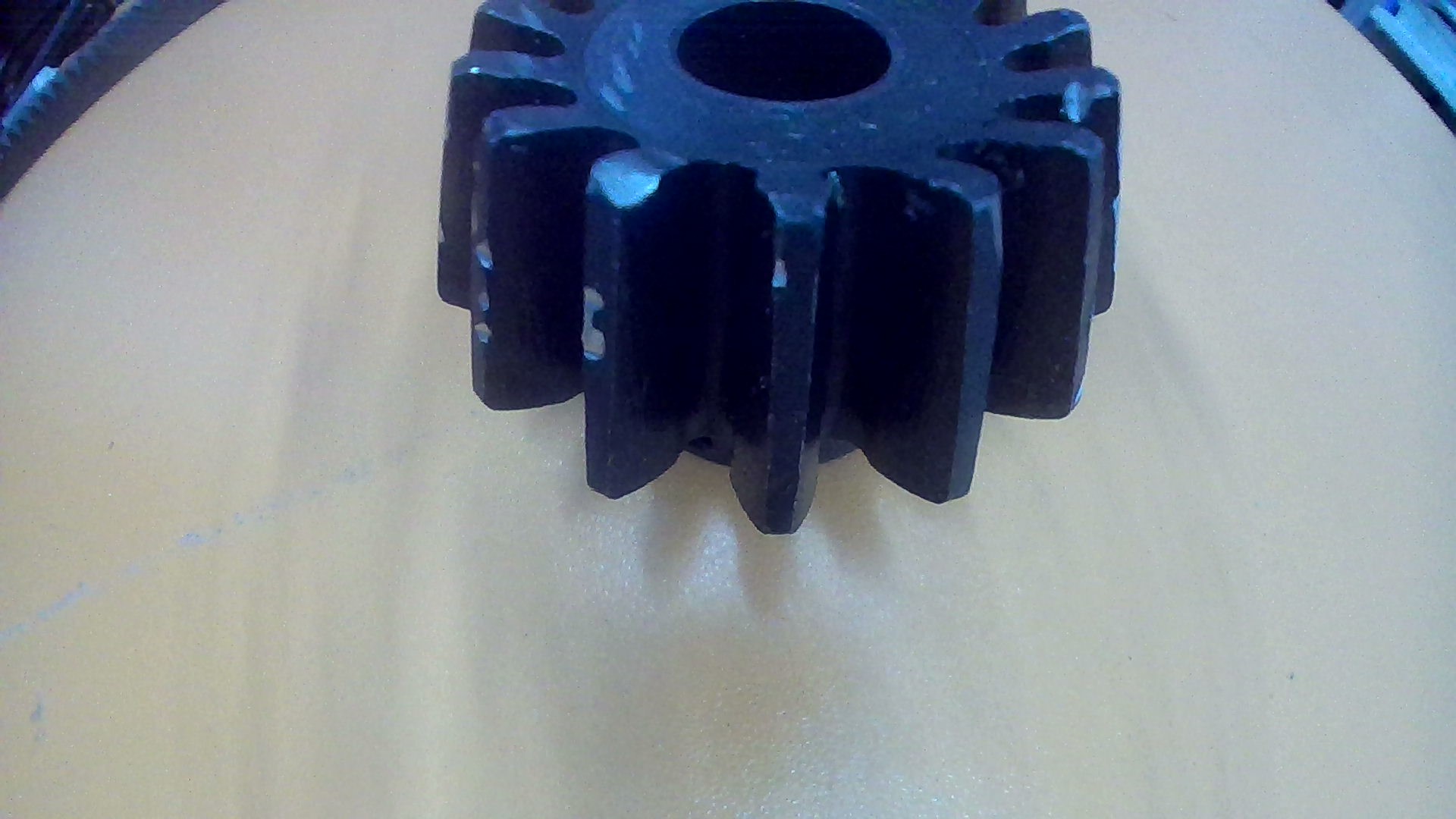

Supplement: S1 Data — (ZIP) [file pone.0322217.s001.zip › dataset/2/WIN_20250112_14_52_03_Pro.jpg]

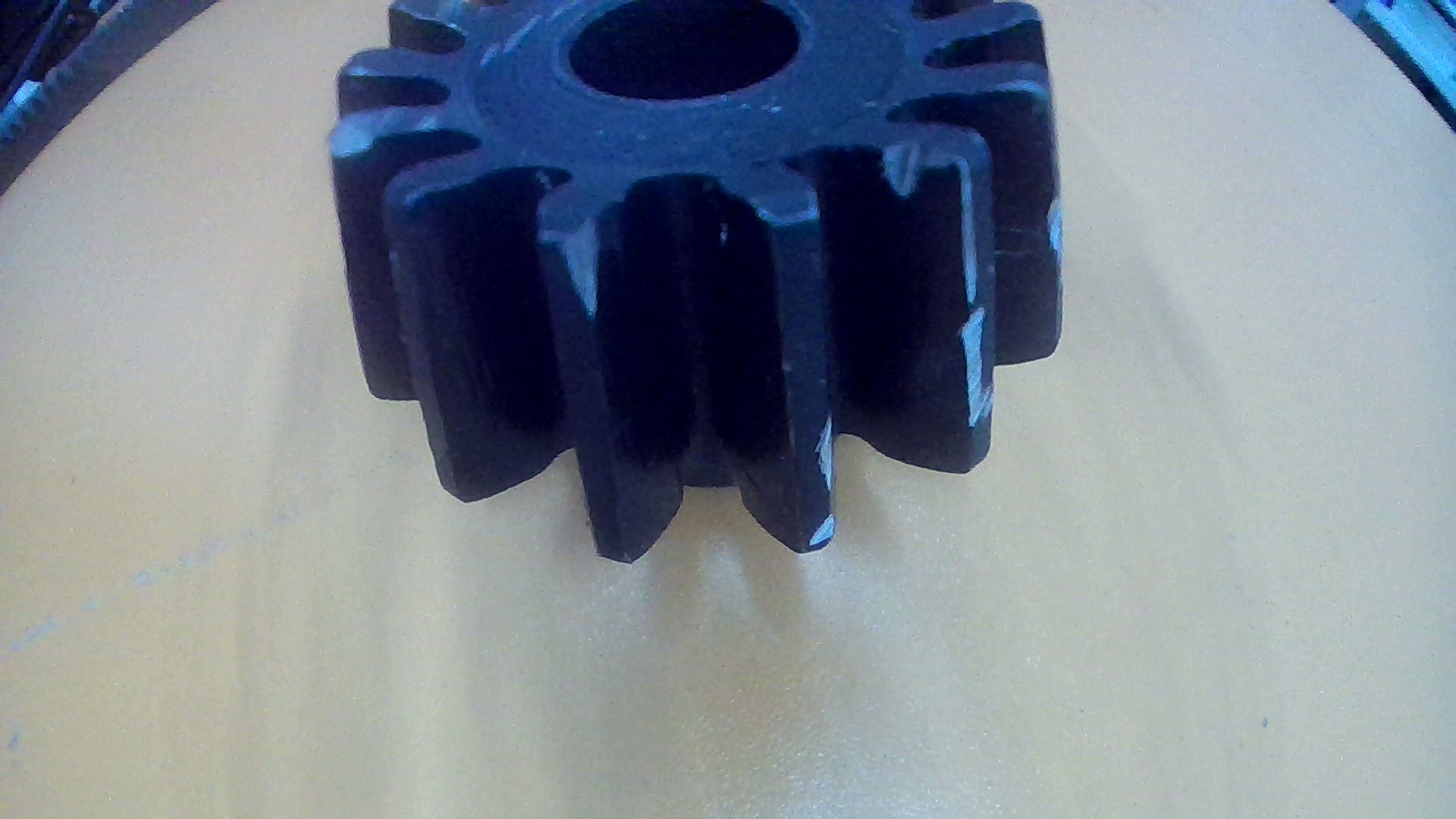

Supplement: S1 Data — (ZIP) [file pone.0322217.s001.zip › dataset/2/WIN_20250112_14_52_08_Pro.jpg]

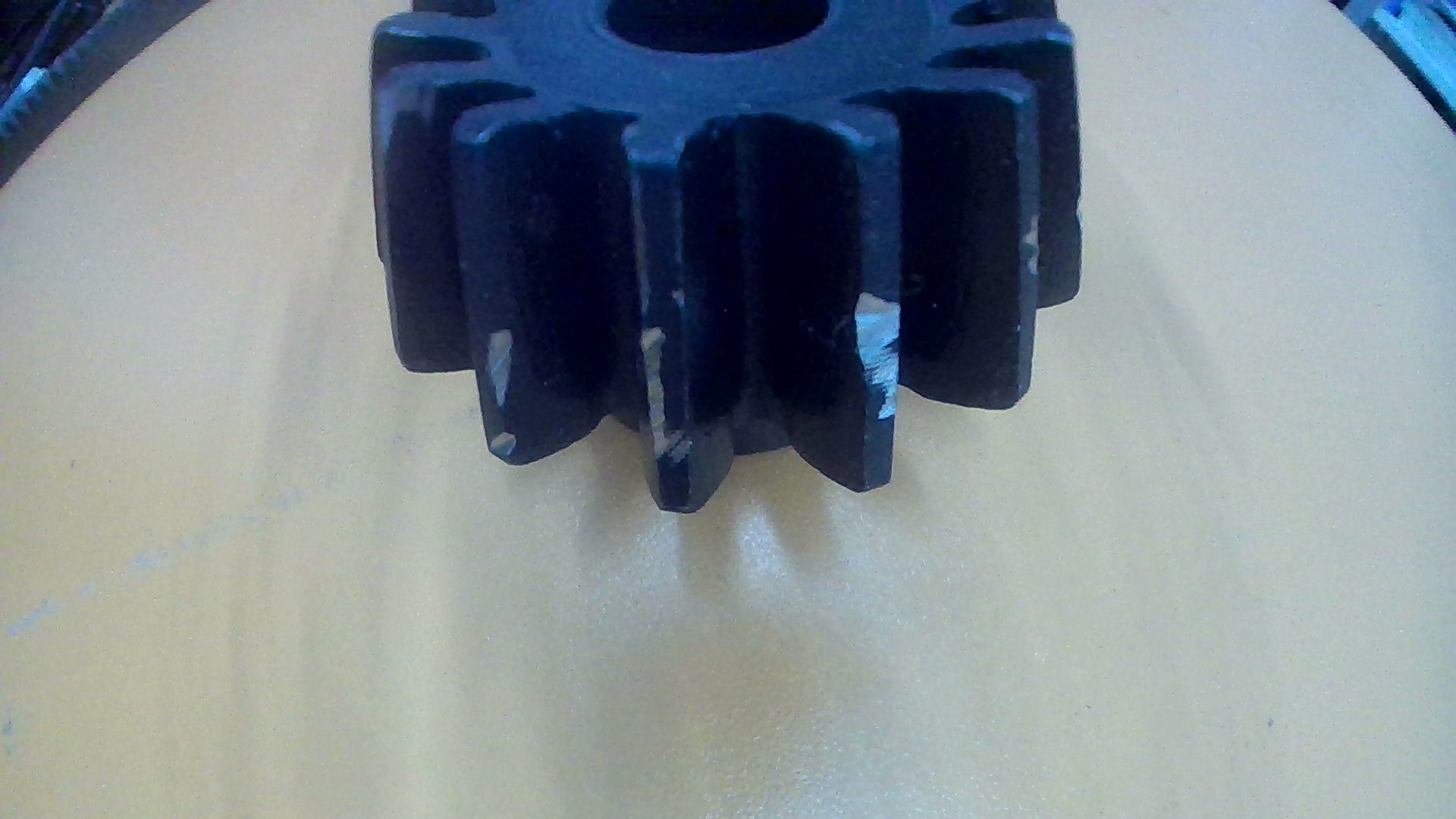

Supplement: S1 Data — (ZIP) [file pone.0322217.s001.zip › dataset/2/WIN_20250112_14_52_11_Pro.jpg]

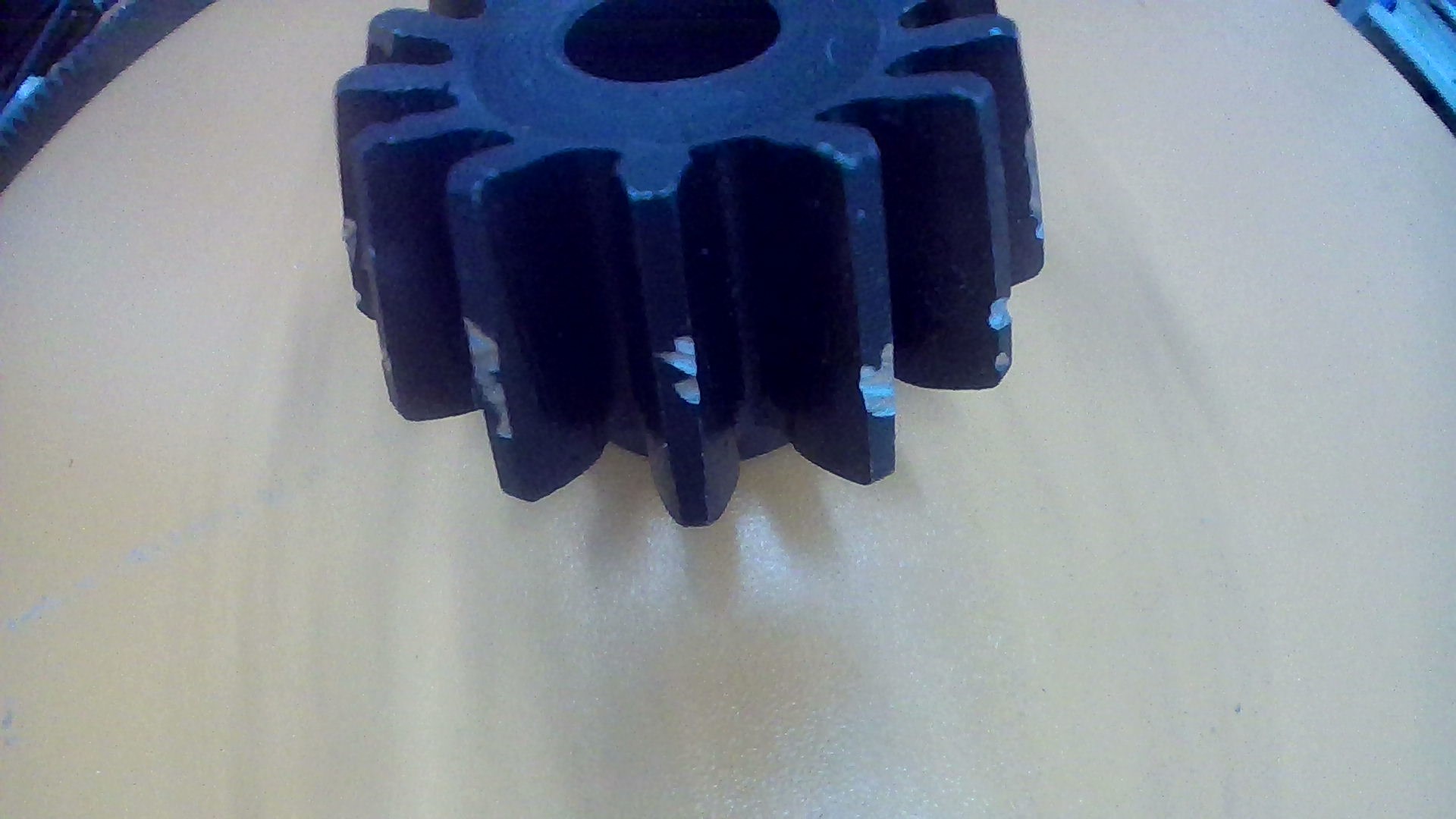

Supplement: S1 Data — (ZIP) [file pone.0322217.s001.zip › dataset/2/WIN_20250112_14_52_16_Pro.jpg]

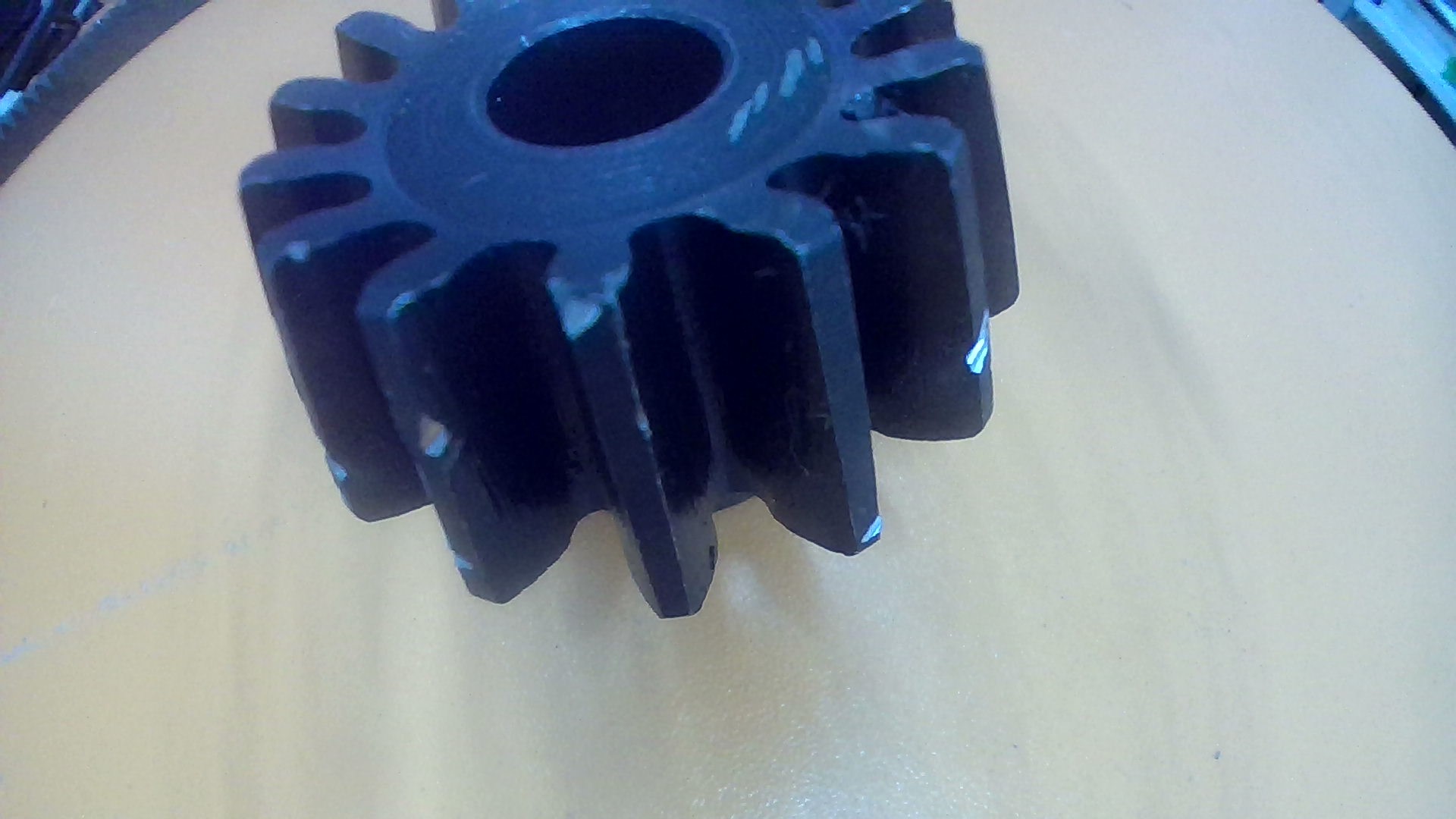

Supplement: S1 Data — (ZIP) [file pone.0322217.s001.zip › dataset/2/WIN_20250112_14_52_24_Pro.jpg]

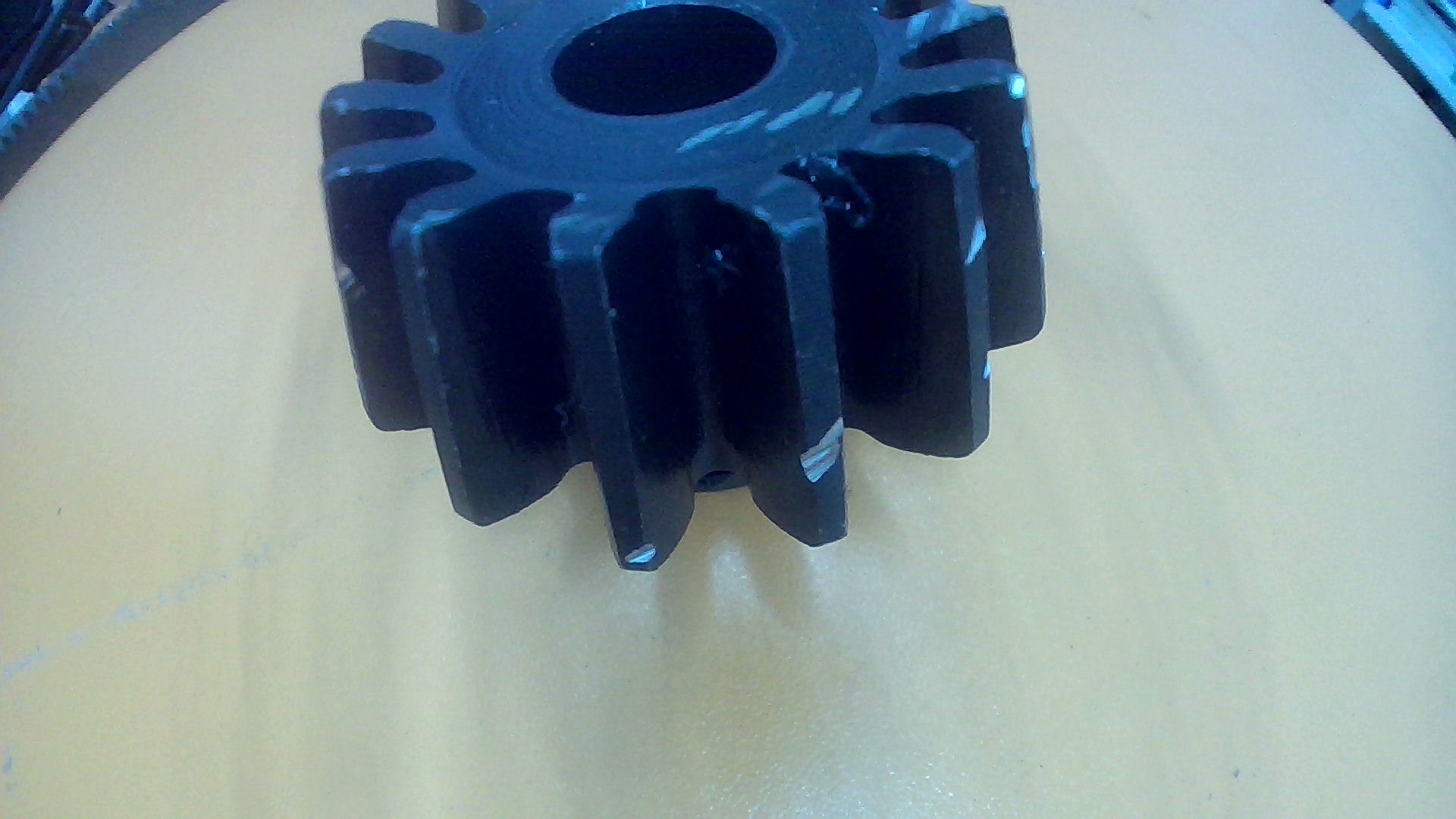

Supplement: S1 Data — (ZIP) [file pone.0322217.s001.zip › dataset/2/WIN_20250112_14_52_28_Pro.jpg]

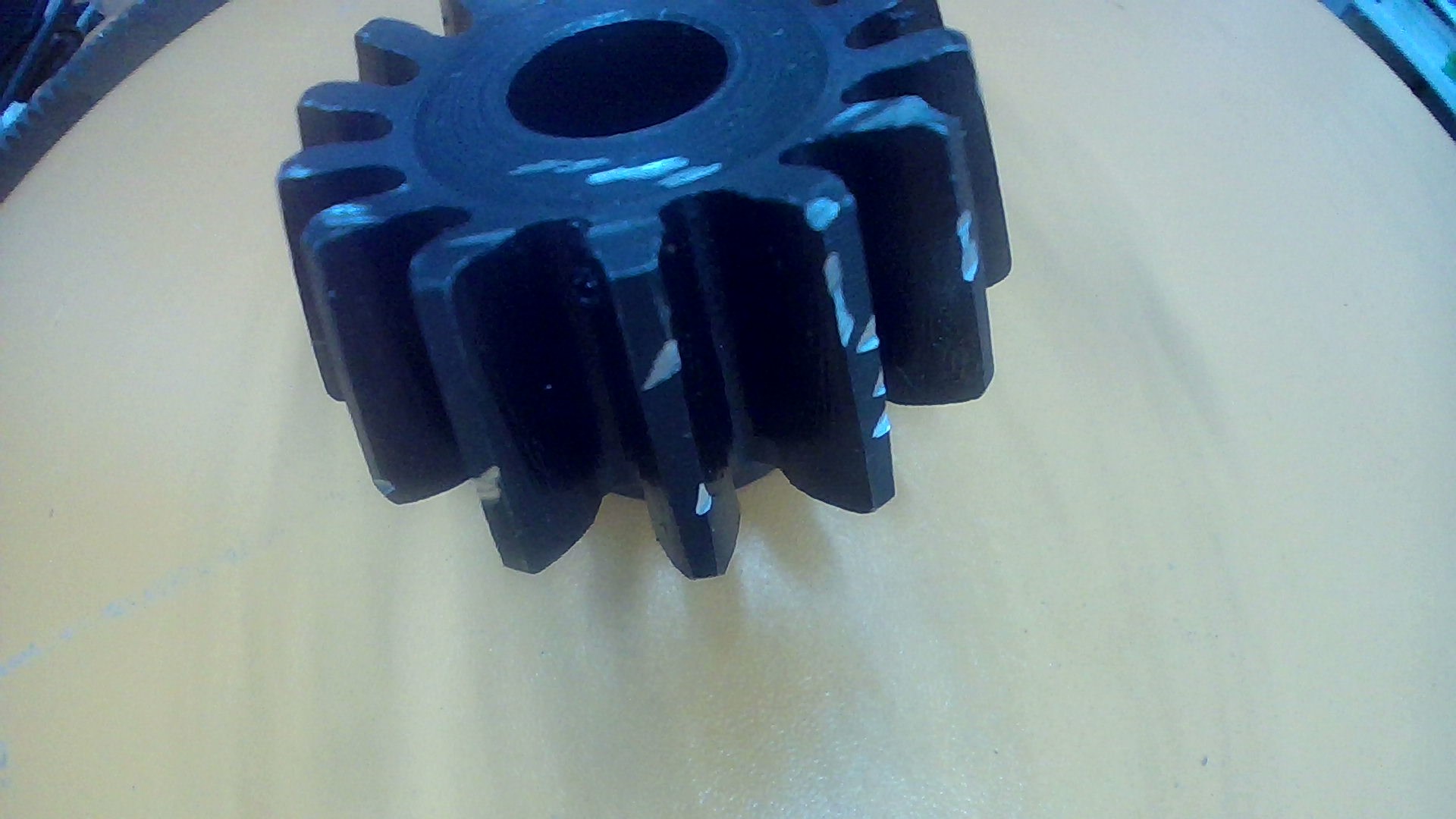

Supplement: S1 Data — (ZIP) [file pone.0322217.s001.zip › dataset/2/WIN_20250112_14_52_32_Pro.jpg]

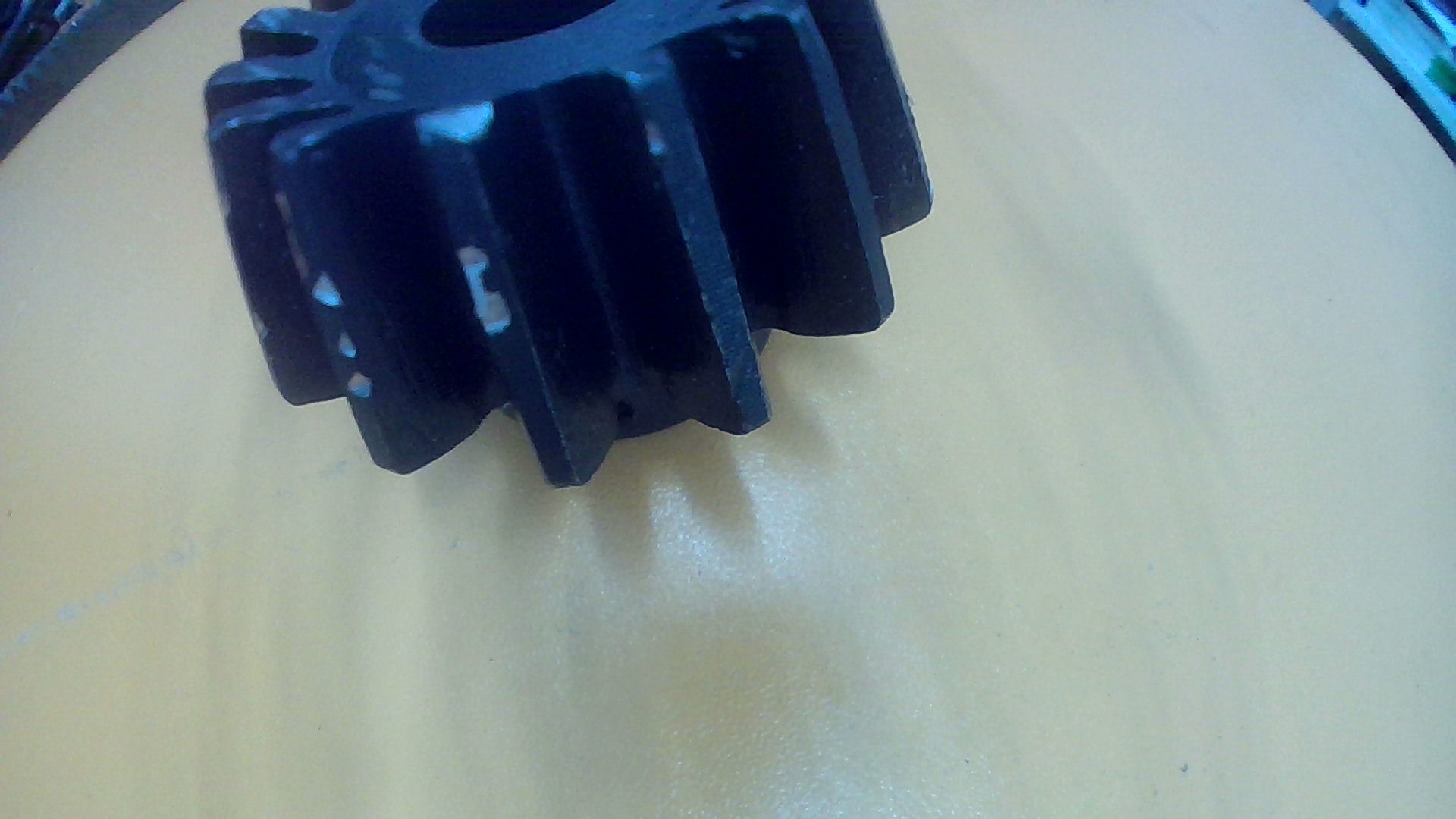

Supplement: S1 Data — (ZIP) [file pone.0322217.s001.zip › dataset/2/WIN_20250112_14_52_37_Pro.jpg]

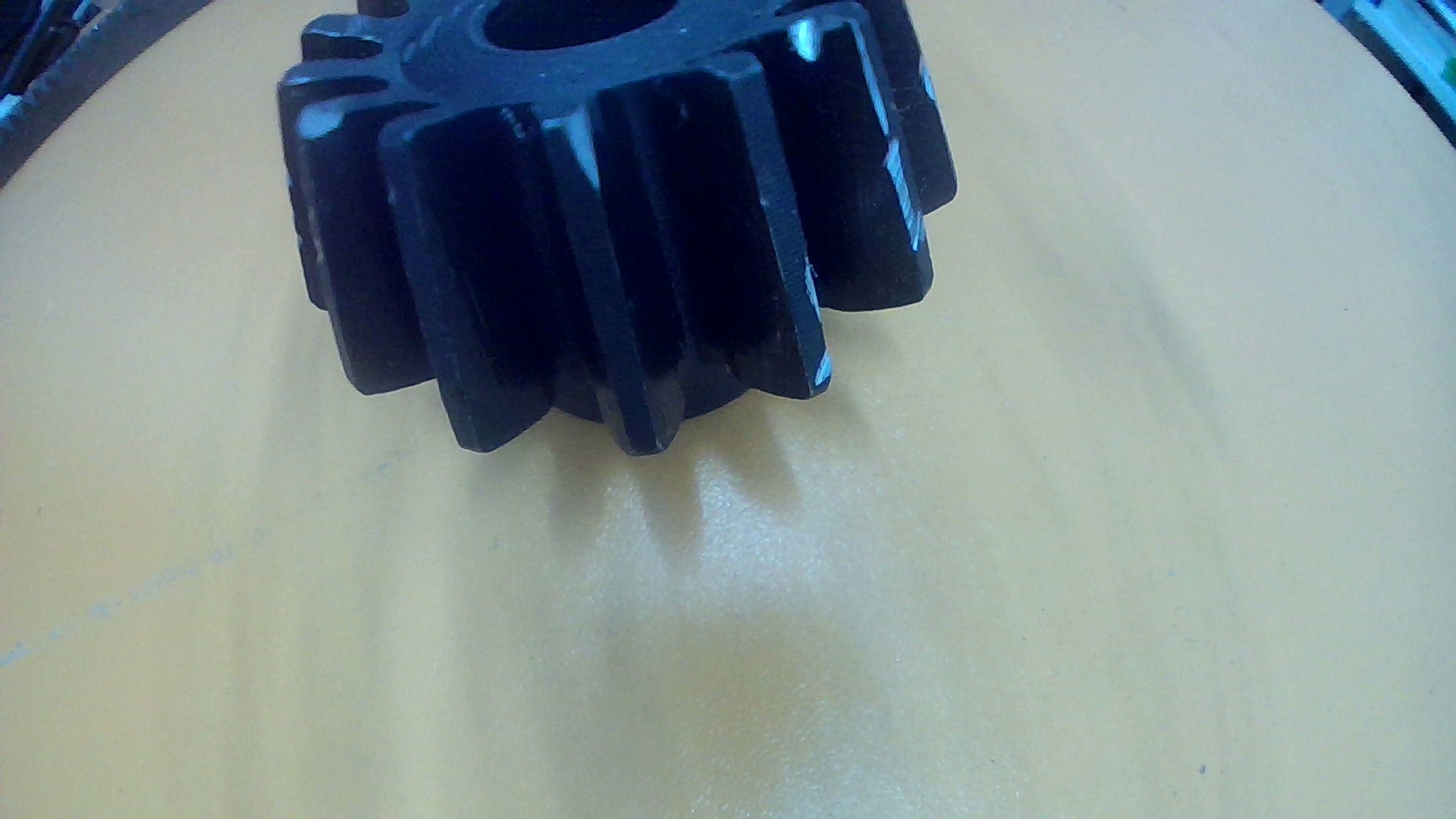

Supplement: S1 Data — (ZIP) [file pone.0322217.s001.zip › dataset/2/WIN_20250112_14_52_40_Pro.jpg]

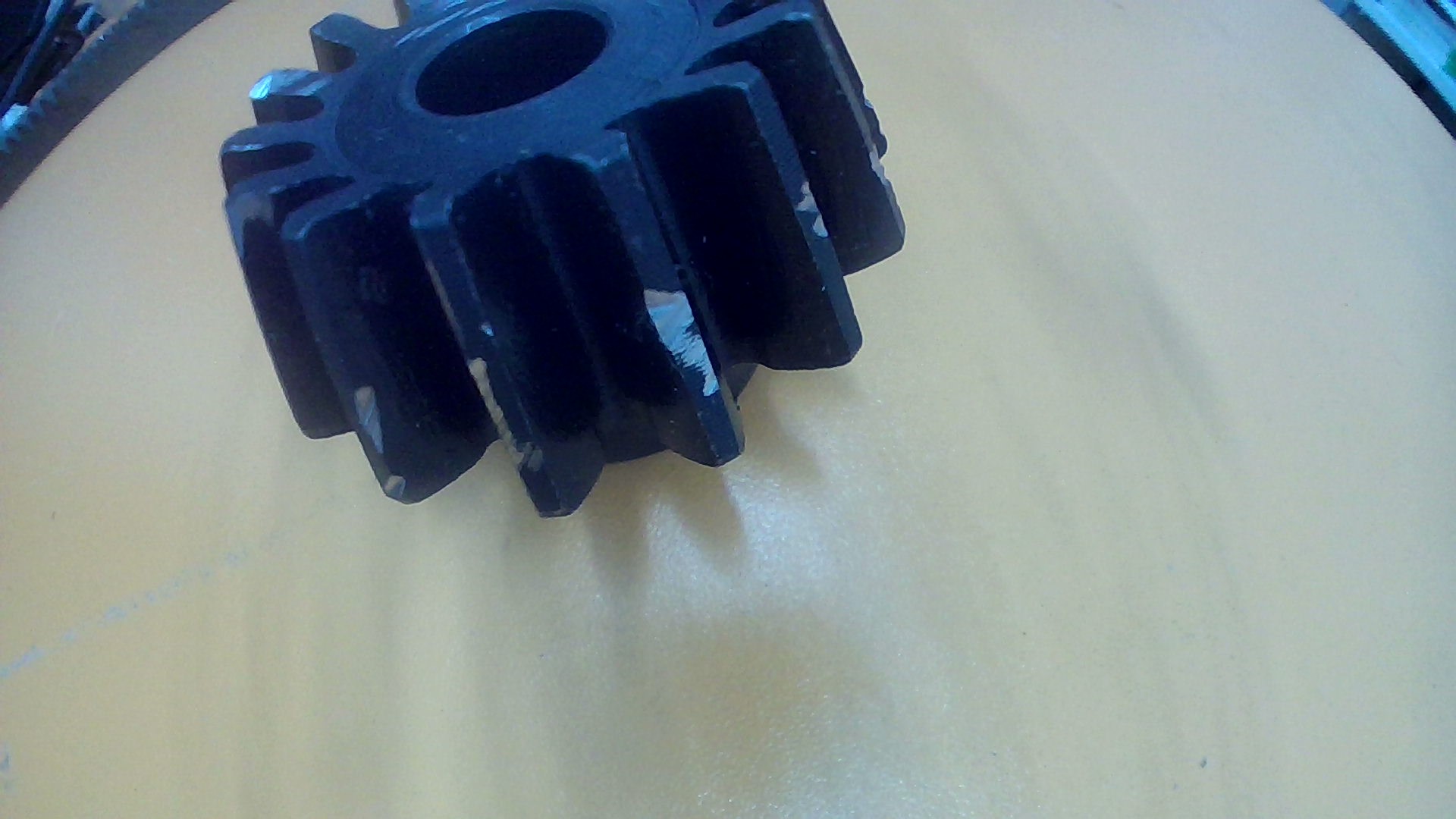

Supplement: S1 Data — (ZIP) [file pone.0322217.s001.zip › dataset/2/WIN_20250112_14_52_44_Pro.jpg]

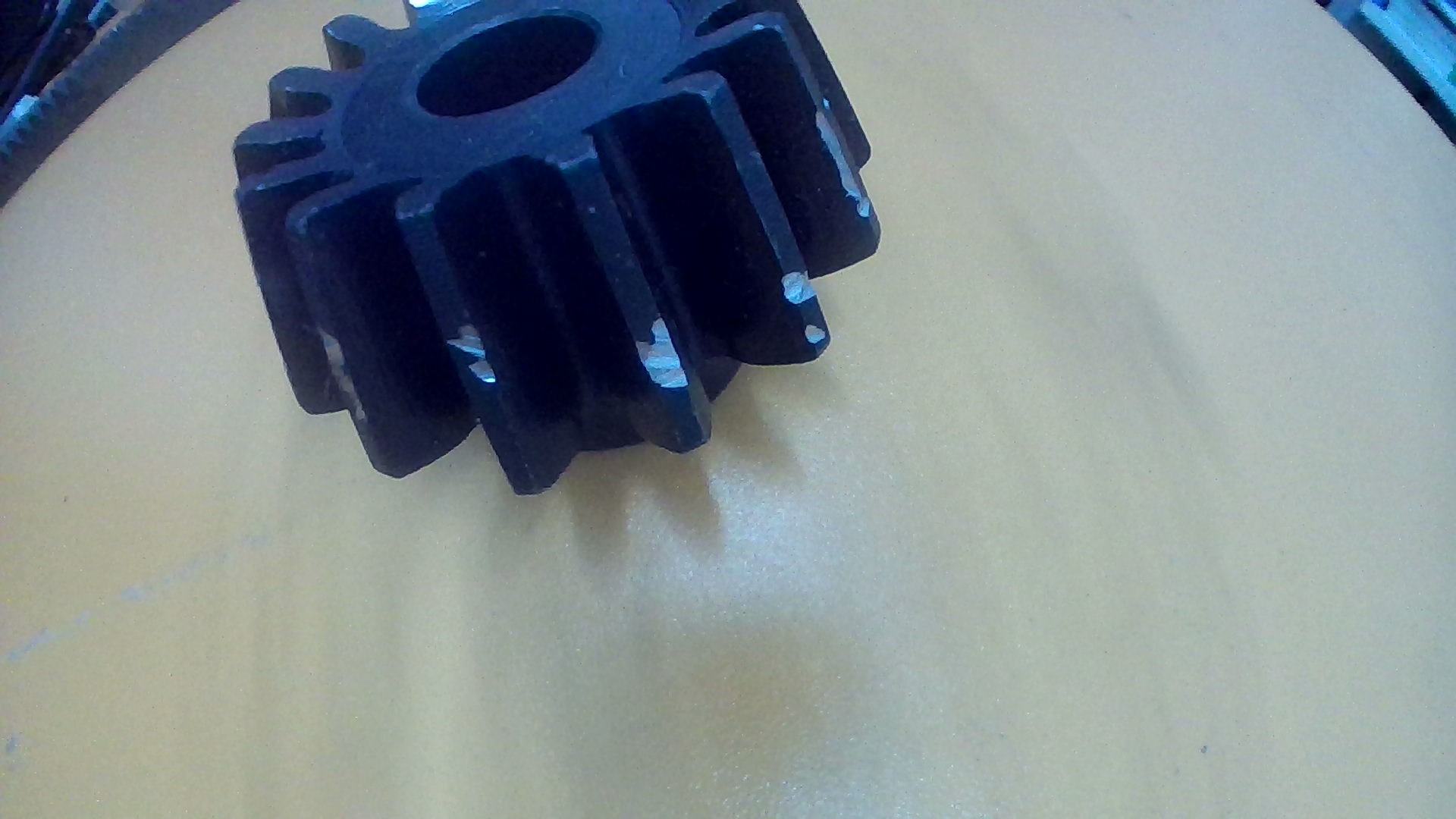

Supplement: S1 Data — (ZIP) [file pone.0322217.s001.zip › dataset/2/WIN_20250112_14_52_47_Pro.jpg]

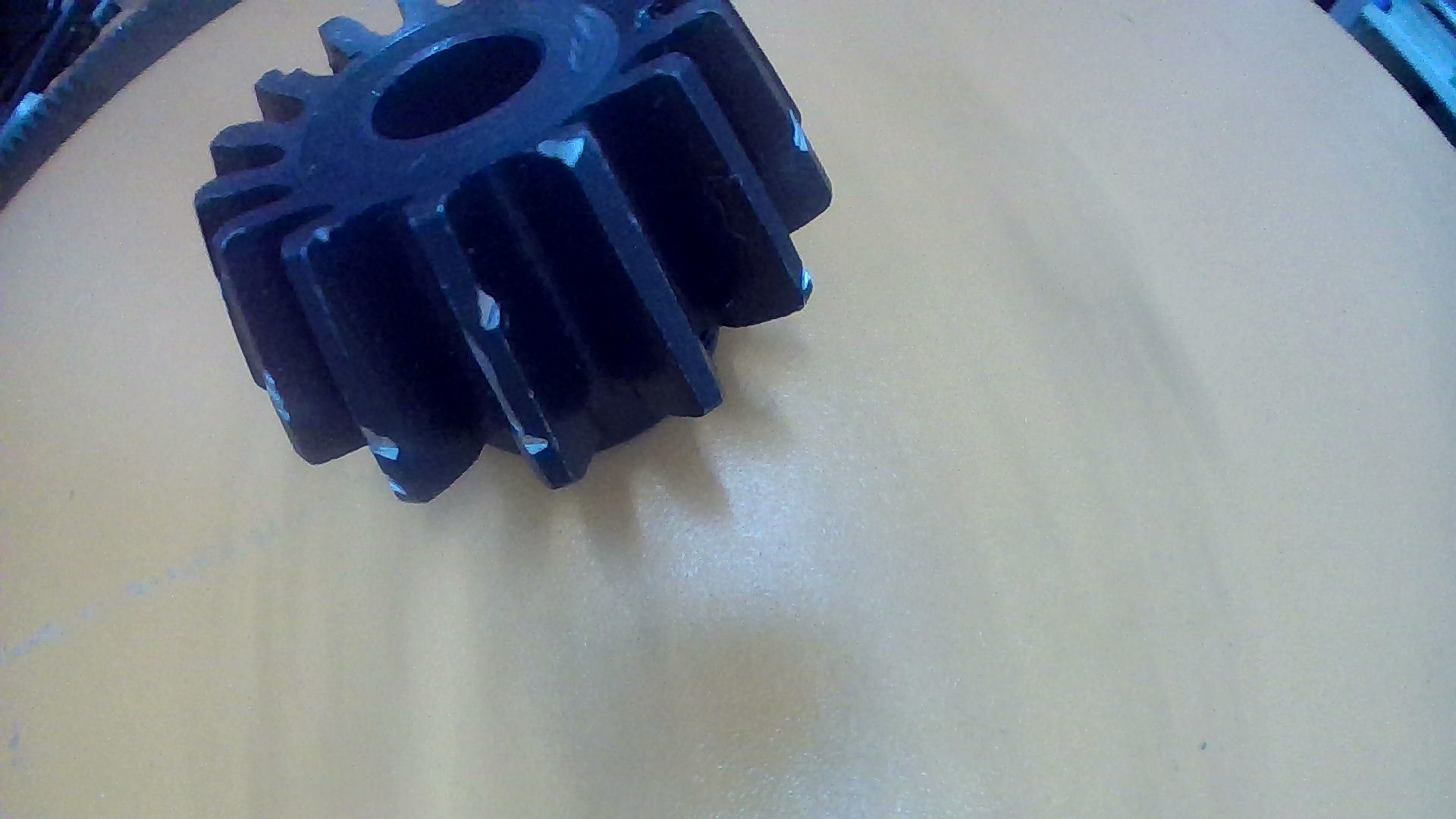

Supplement: S1 Data — (ZIP) [file pone.0322217.s001.zip › dataset/2/WIN_20250112_14_52_50_Pro.jpg]

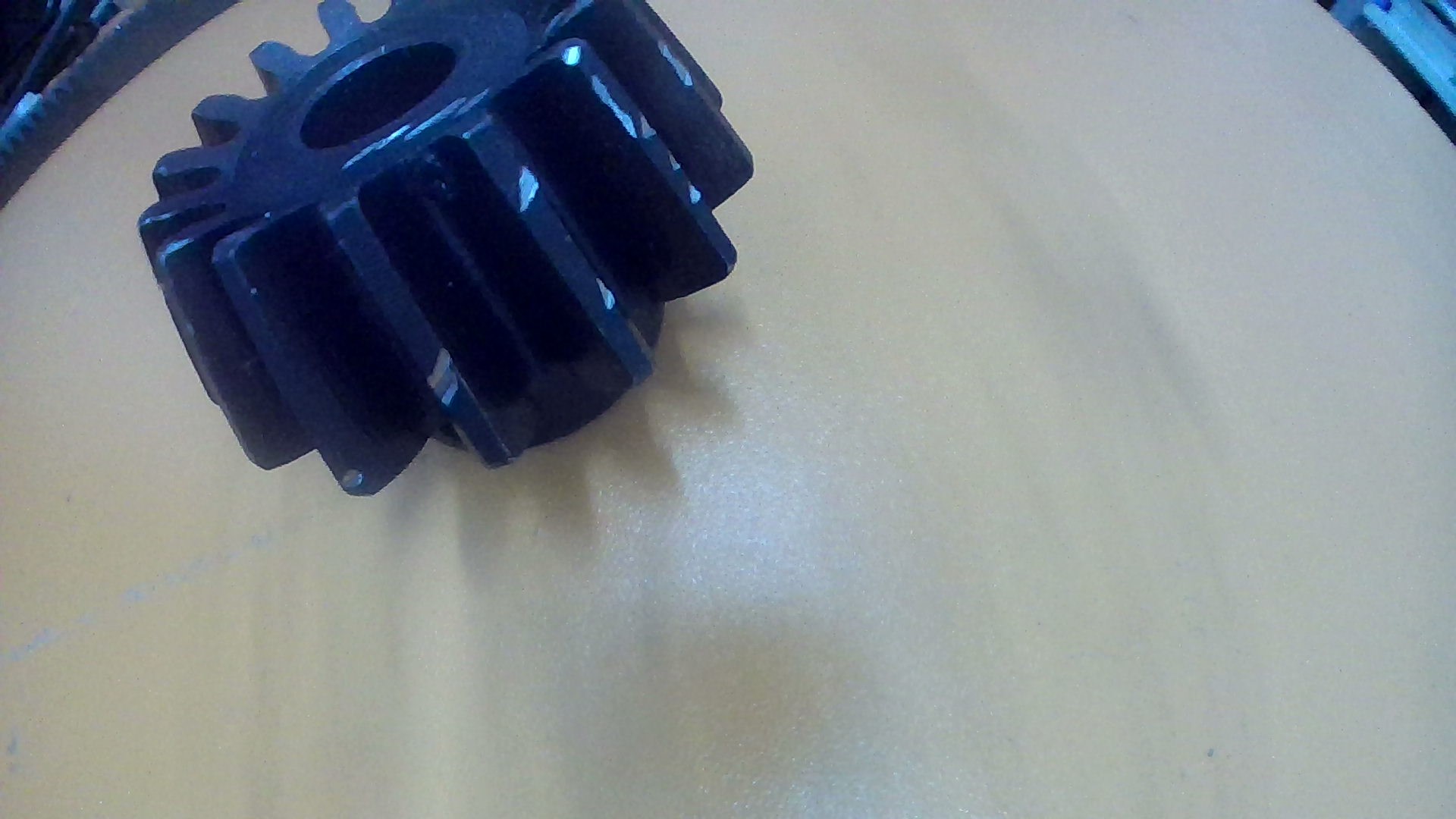

Supplement: S1 Data — (ZIP) [file pone.0322217.s001.zip › dataset/2/WIN_20250112_14_52_54_Pro.jpg]

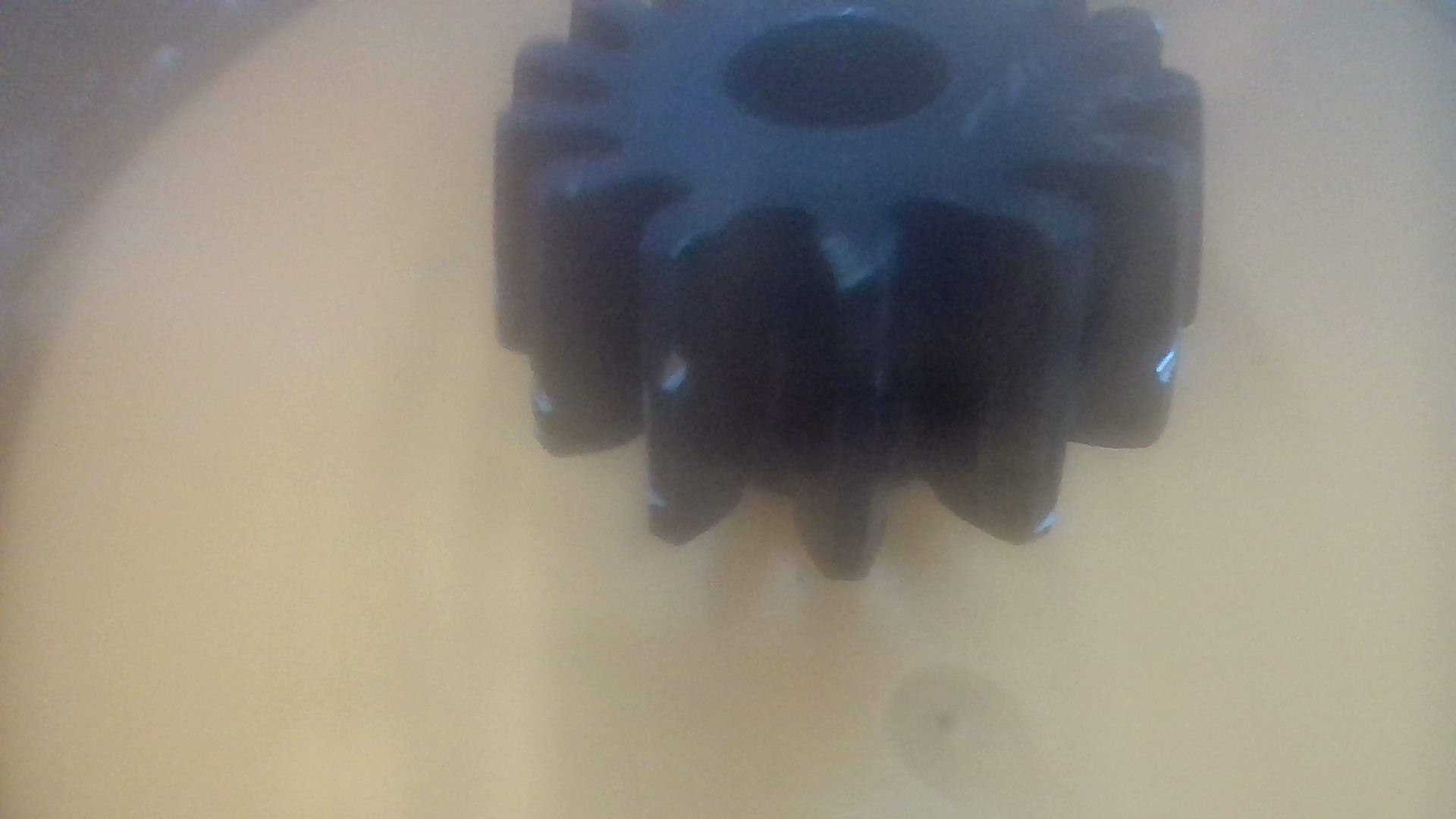

Supplement: S1 Data — (ZIP) [file pone.0322217.s001.zip › dataset/2/WIN_20250112_14_53_03_Pro.jpg]

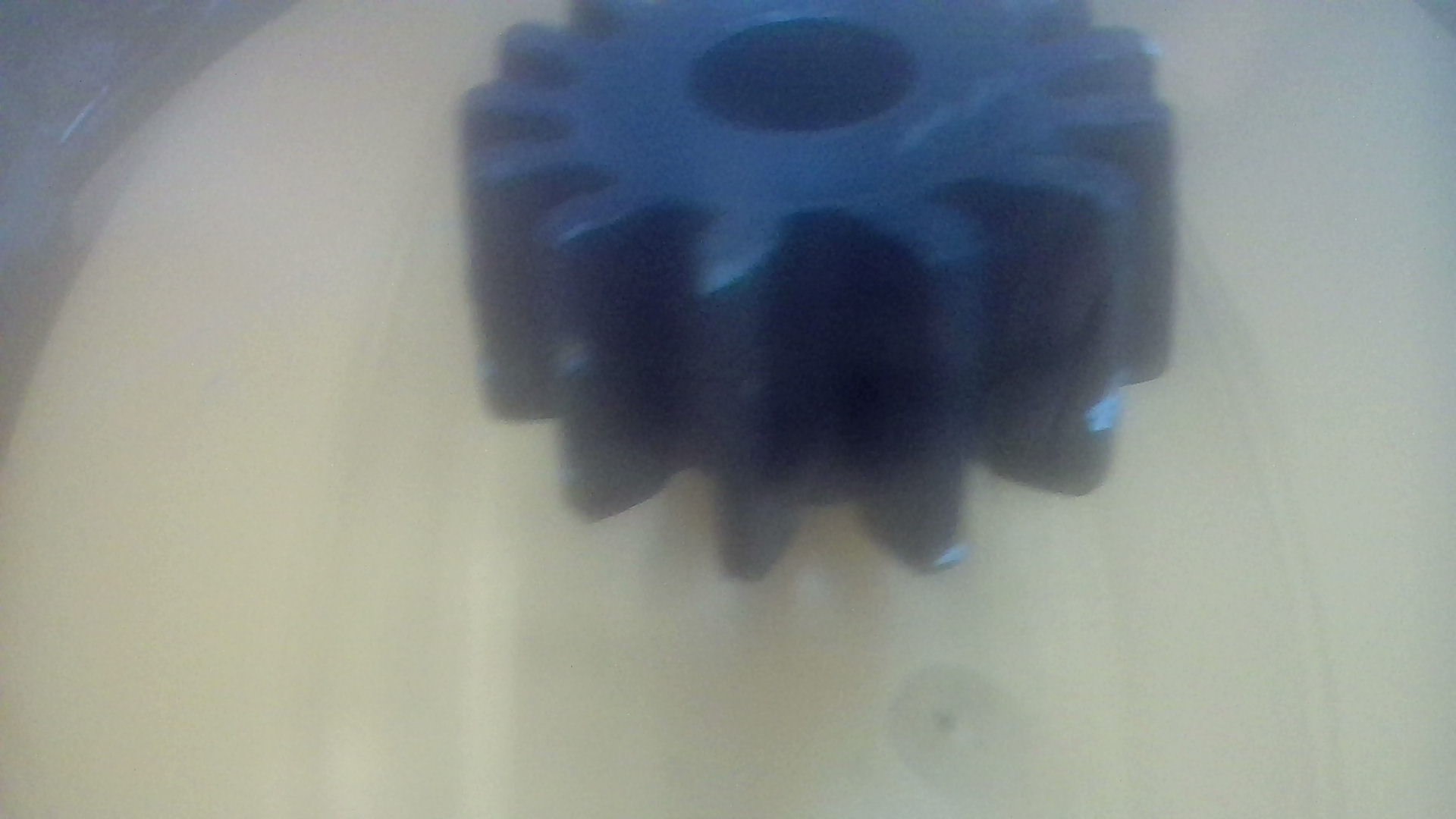

Supplement: S1 Data — (ZIP) [file pone.0322217.s001.zip › dataset/2/WIN_20250112_14_53_04_Pro.jpg]

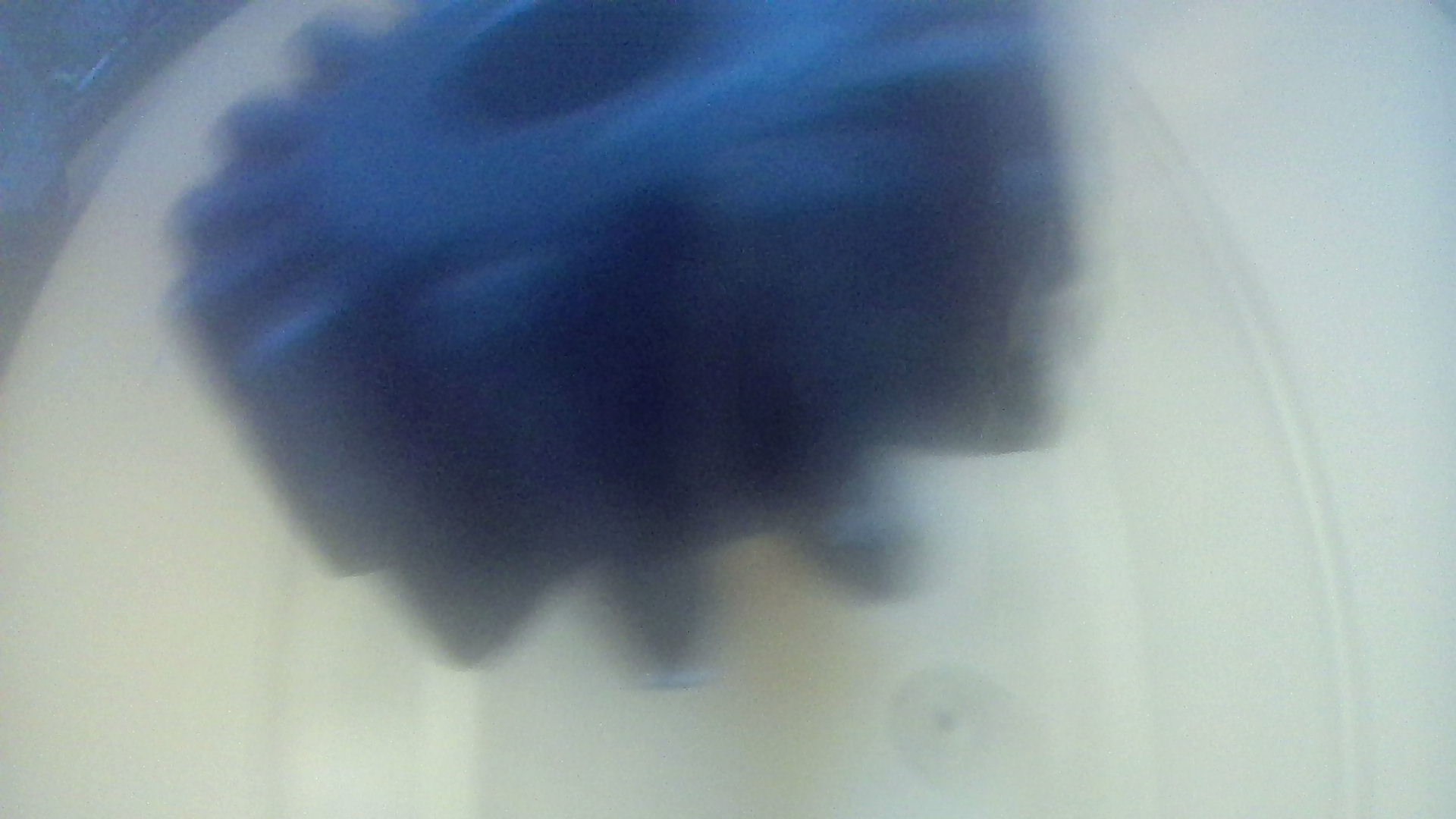

Supplement: S1 Data — (ZIP) [file pone.0322217.s001.zip › dataset/2/WIN_20250112_14_53_06_Pro.jpg]

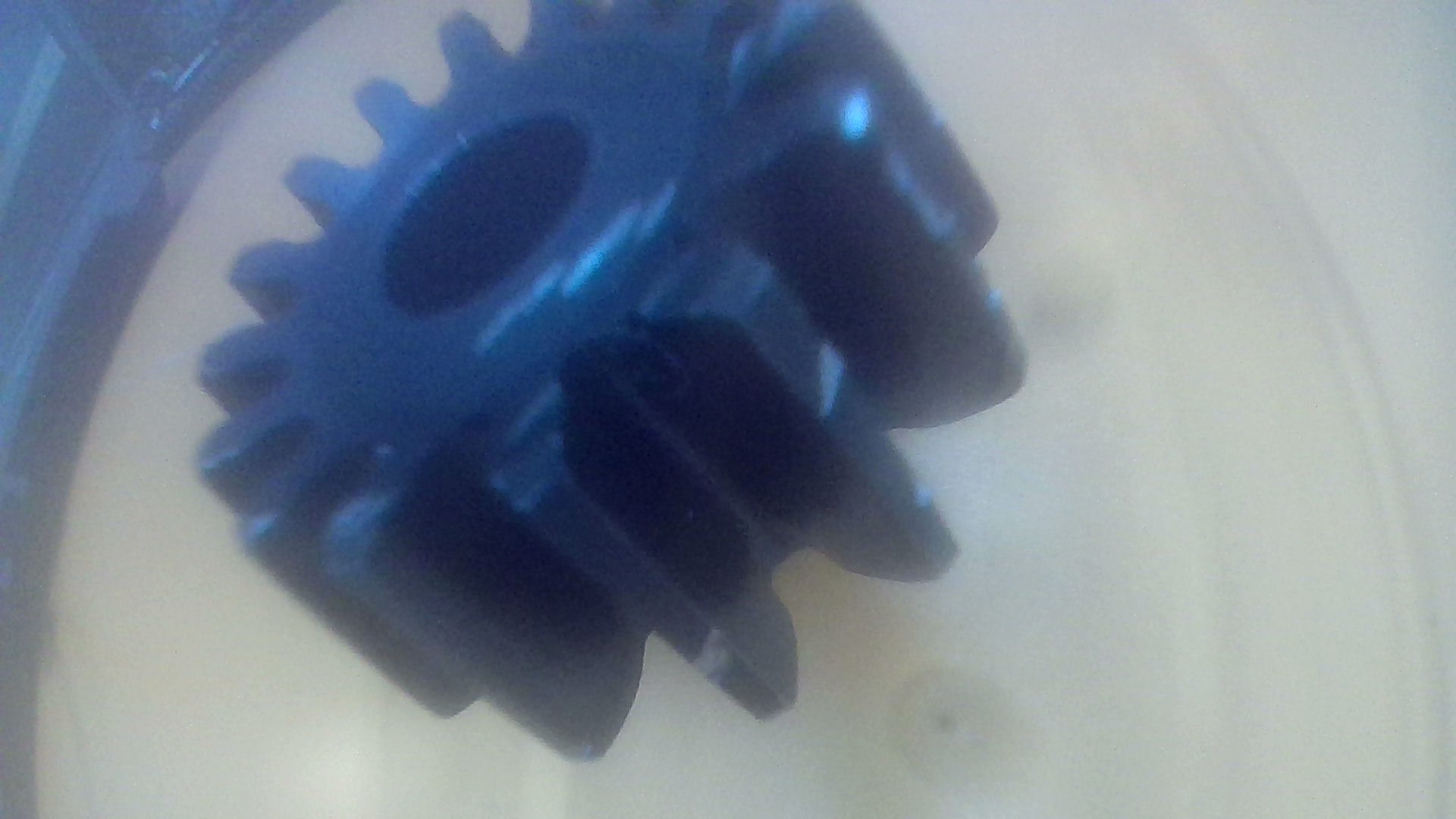

Supplement: S1 Data — (ZIP) [file pone.0322217.s001.zip › dataset/2/WIN_20250112_14_53_07_Pro.jpg]

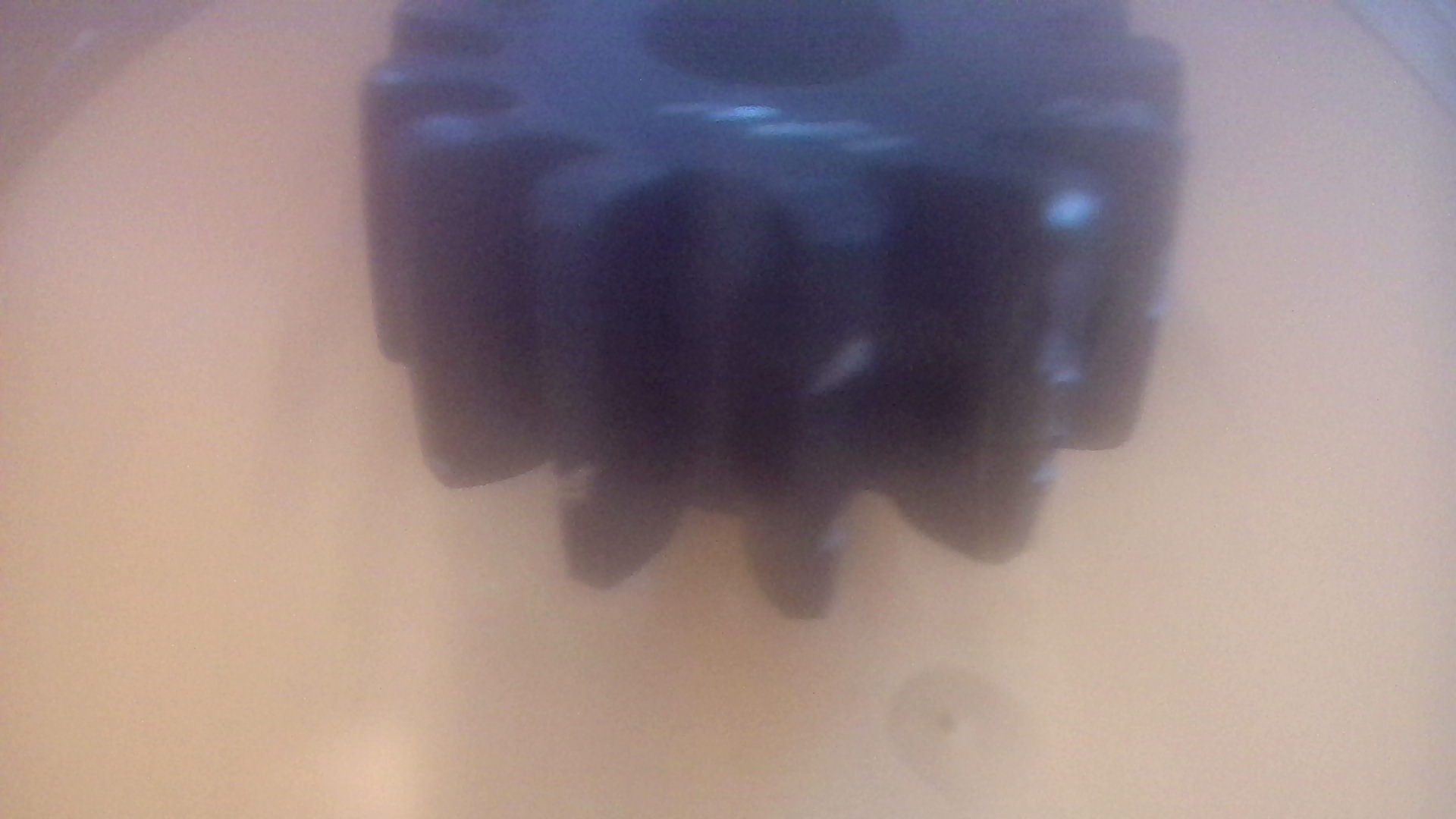

Supplement: S1 Data — (ZIP) [file pone.0322217.s001.zip › dataset/2/WIN_20250112_14_53_12_Pro.jpg]

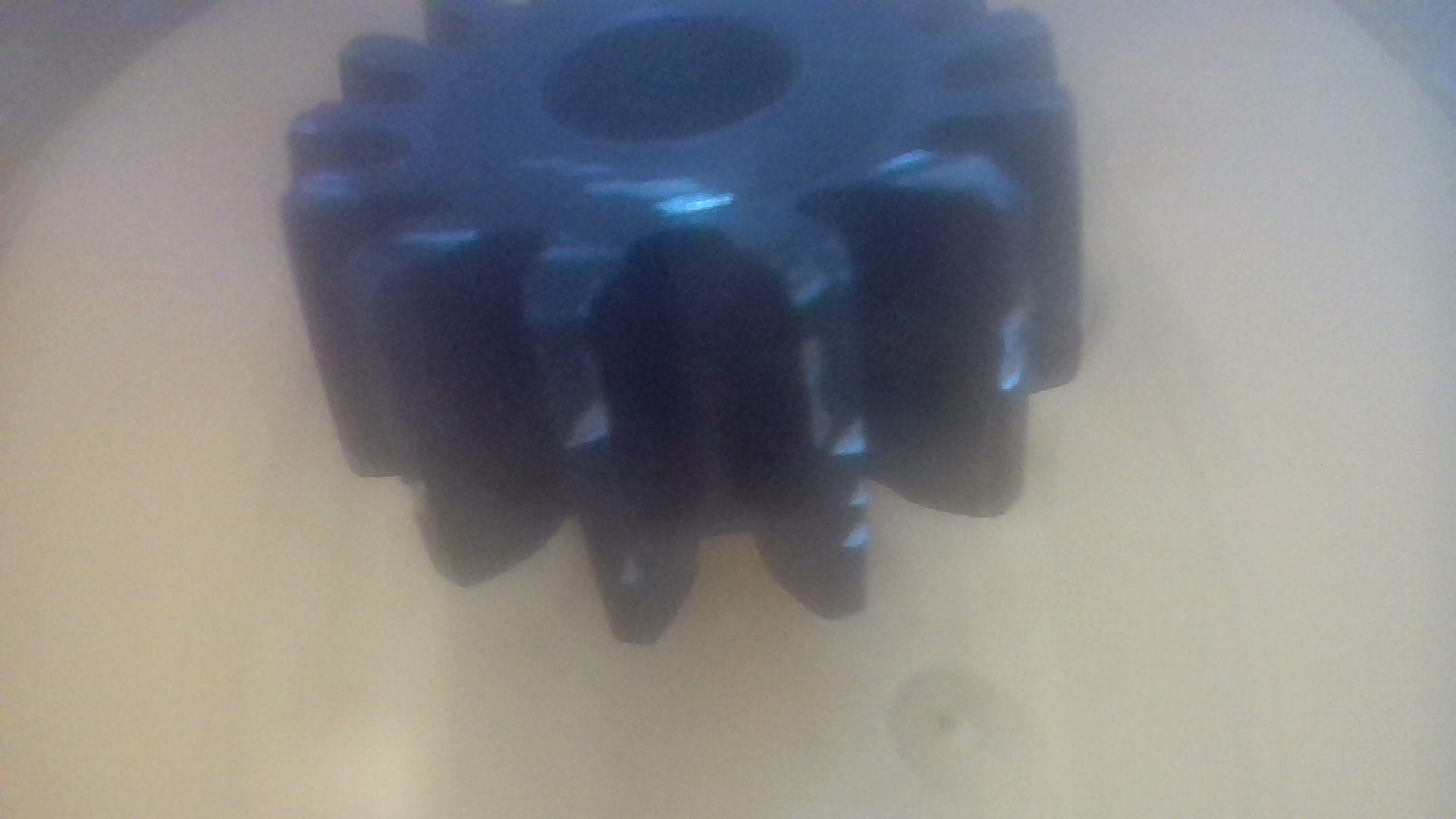

Supplement: S1 Data — (ZIP) [file pone.0322217.s001.zip › dataset/2/WIN_20250112_14_53_13_Pro.jpg]

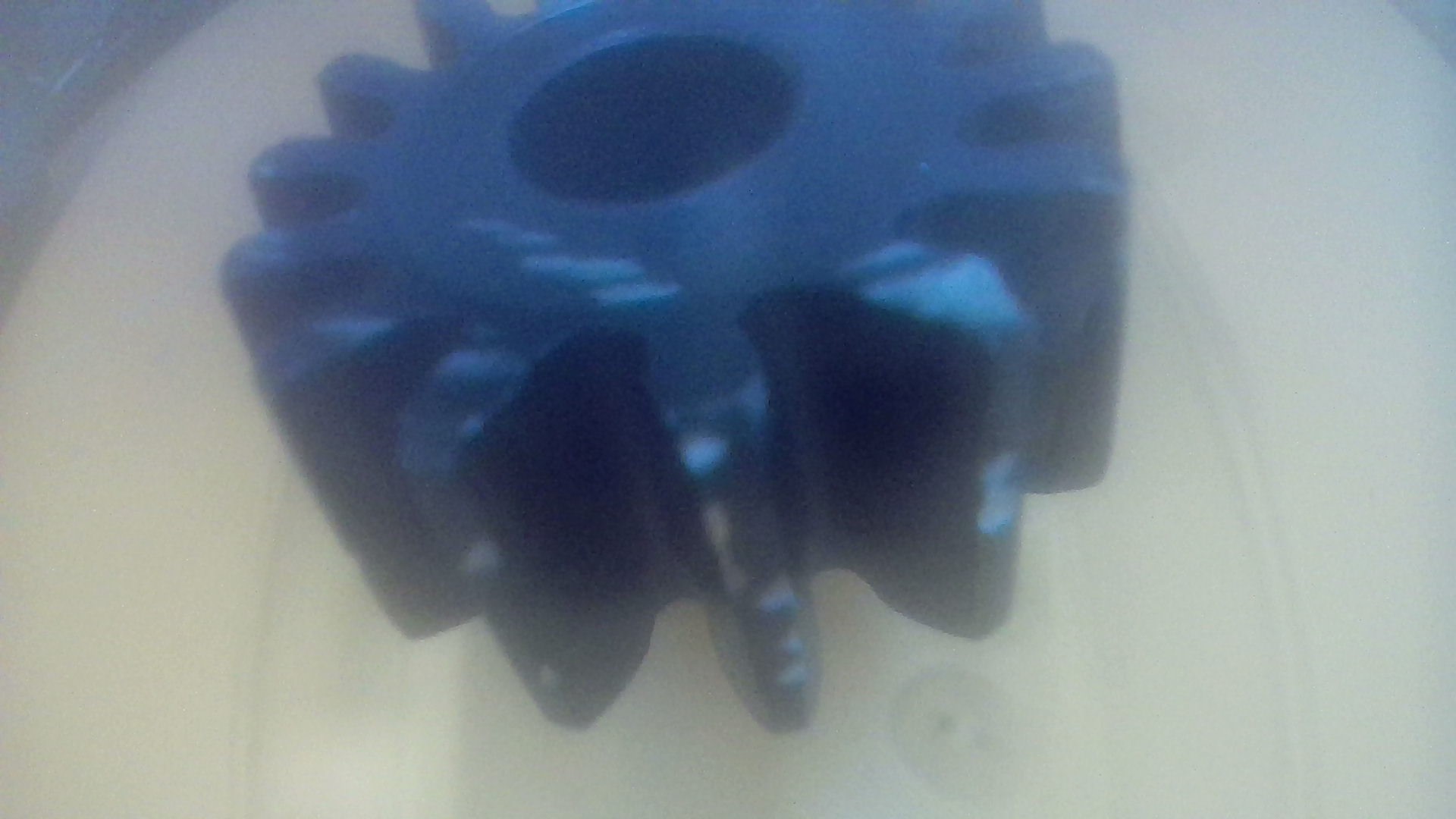

Supplement: S1 Data — (ZIP) [file pone.0322217.s001.zip › dataset/2/WIN_20250112_14_53_15_Pro.jpg]

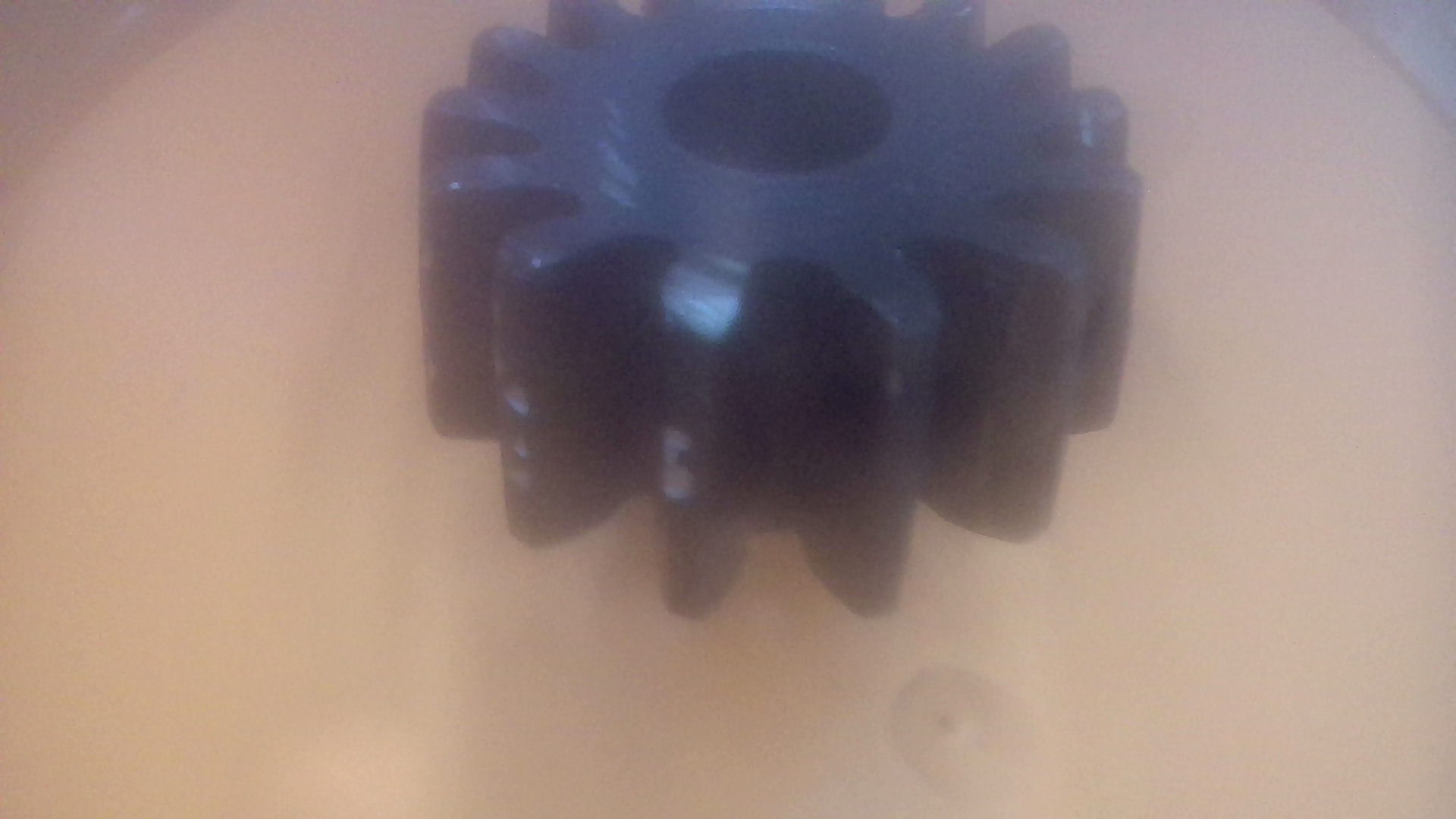

Supplement: S1 Data — (ZIP) [file pone.0322217.s001.zip › dataset/2/WIN_20250112_14_53_19_Pro.jpg]

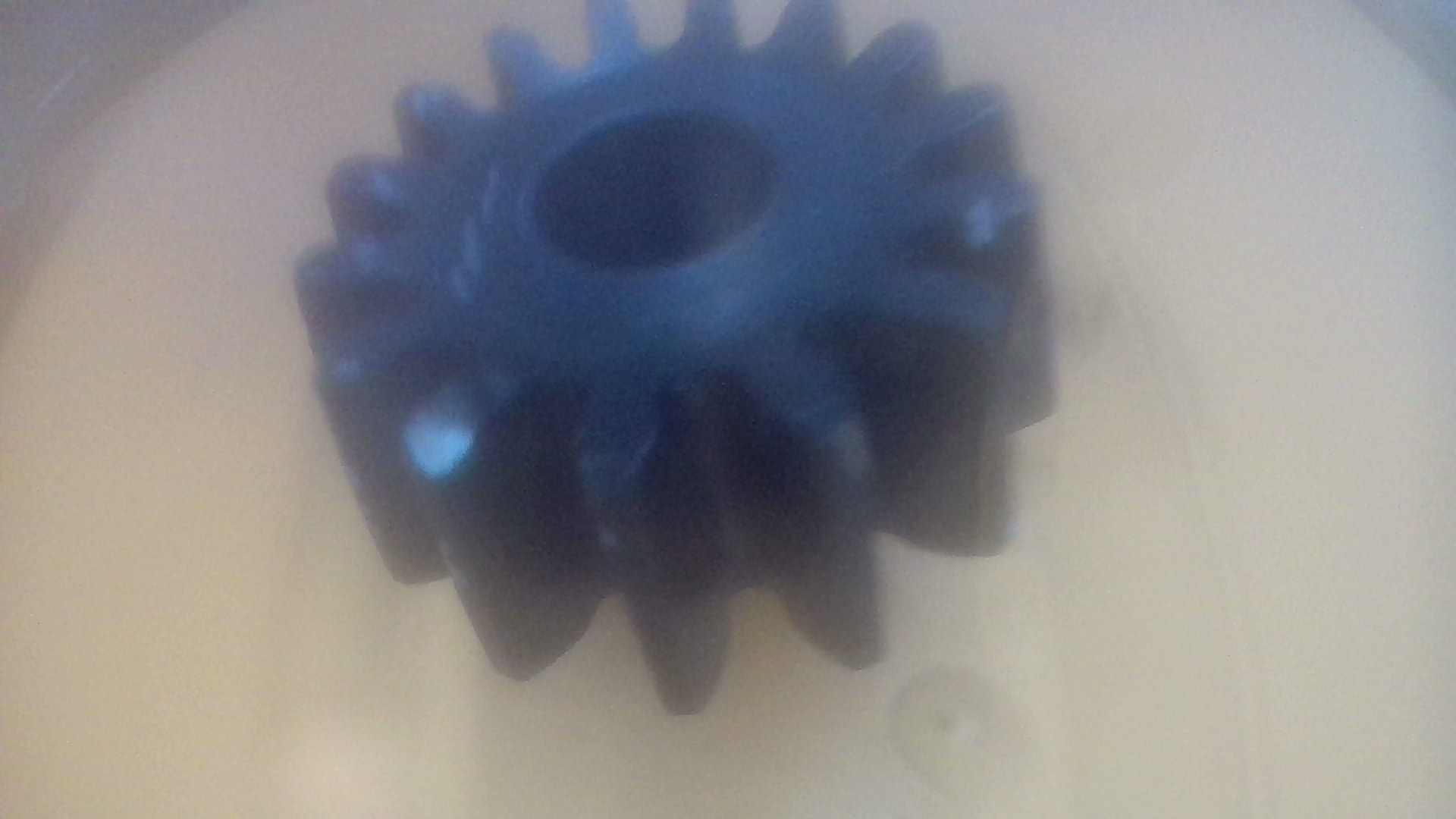

Supplement: S1 Data — (ZIP) [file pone.0322217.s001.zip › dataset/2/WIN_20250112_14_53_20_Pro.jpg]

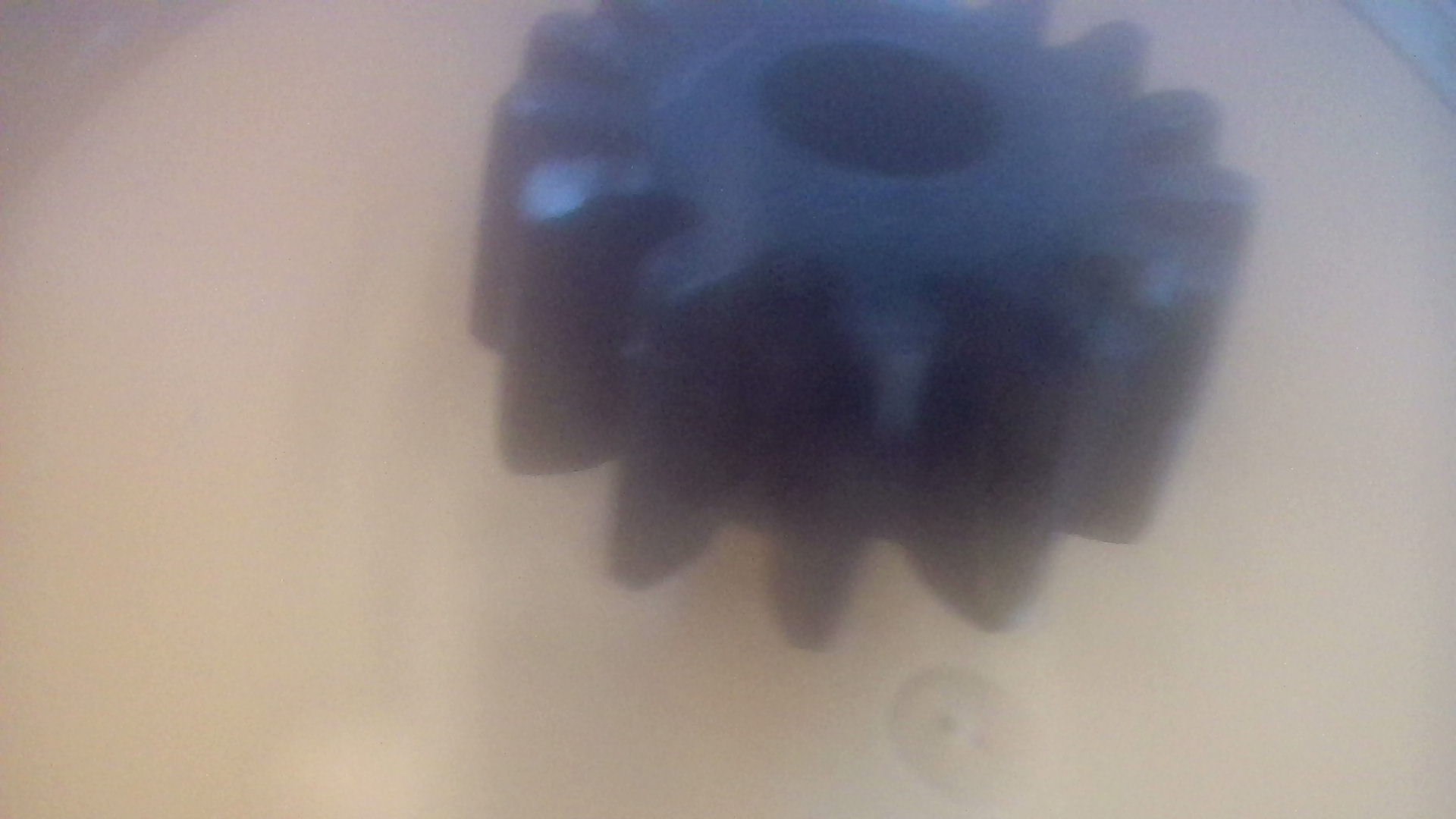

Supplement: S1 Data — (ZIP) [file pone.0322217.s001.zip › dataset/2/WIN_20250112_14_53_25_Pro.jpg]

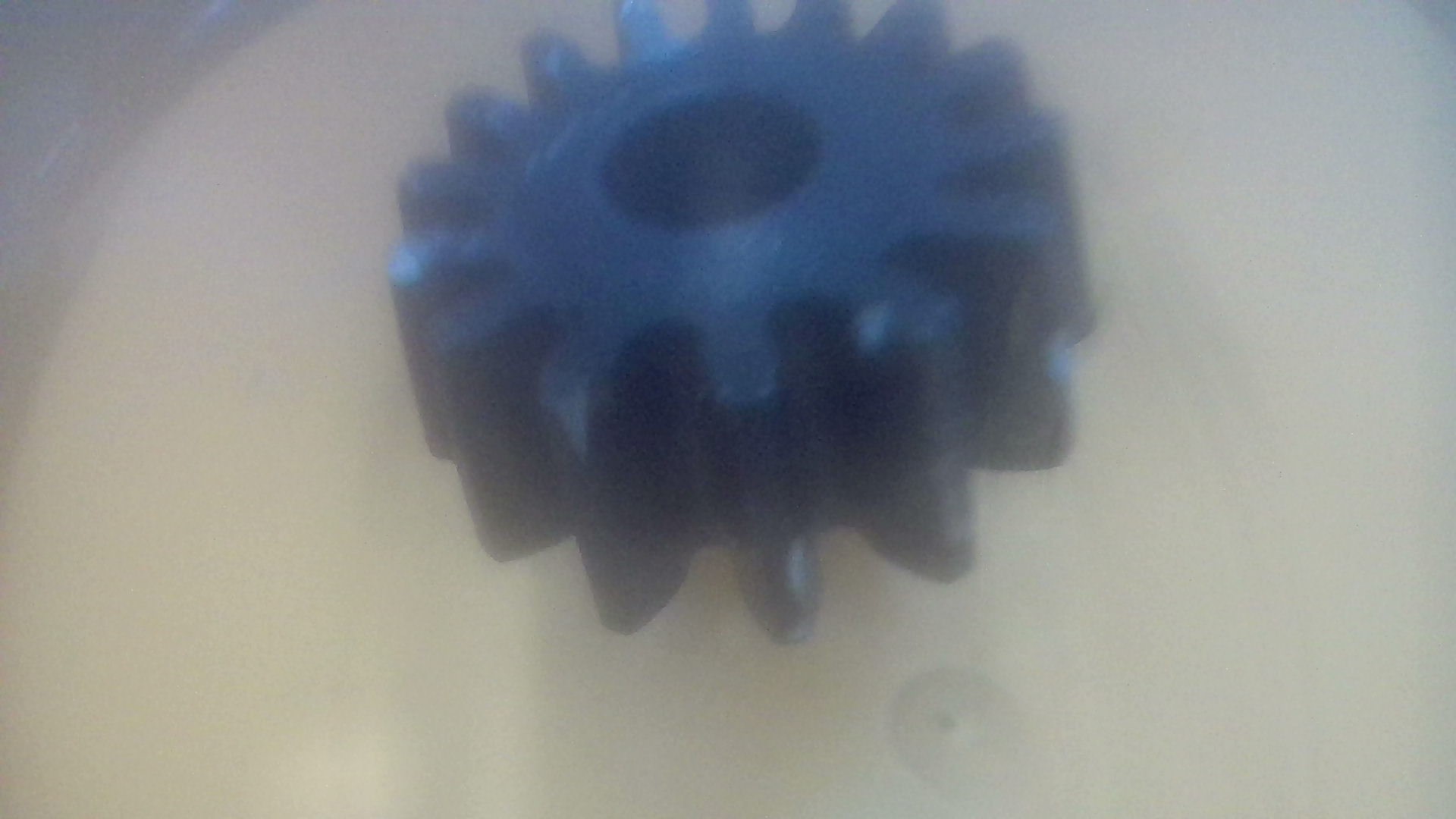

Supplement: S1 Data — (ZIP) [file pone.0322217.s001.zip › dataset/2/WIN_20250112_14_53_26_Pro.jpg]

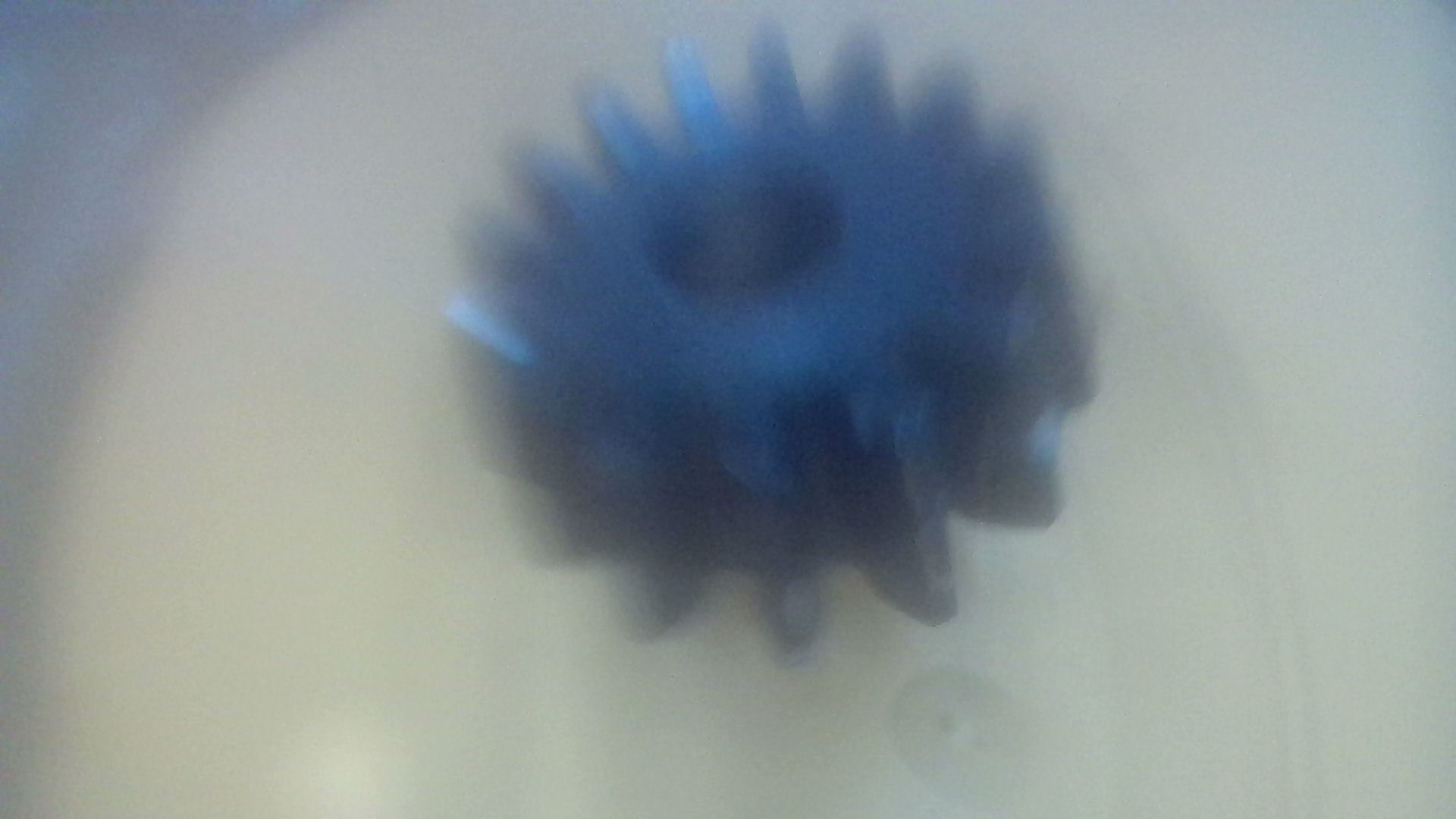

Supplement: S1 Data — (ZIP) [file pone.0322217.s001.zip › dataset/2/WIN_20250112_14_53_27_Pro.jpg]

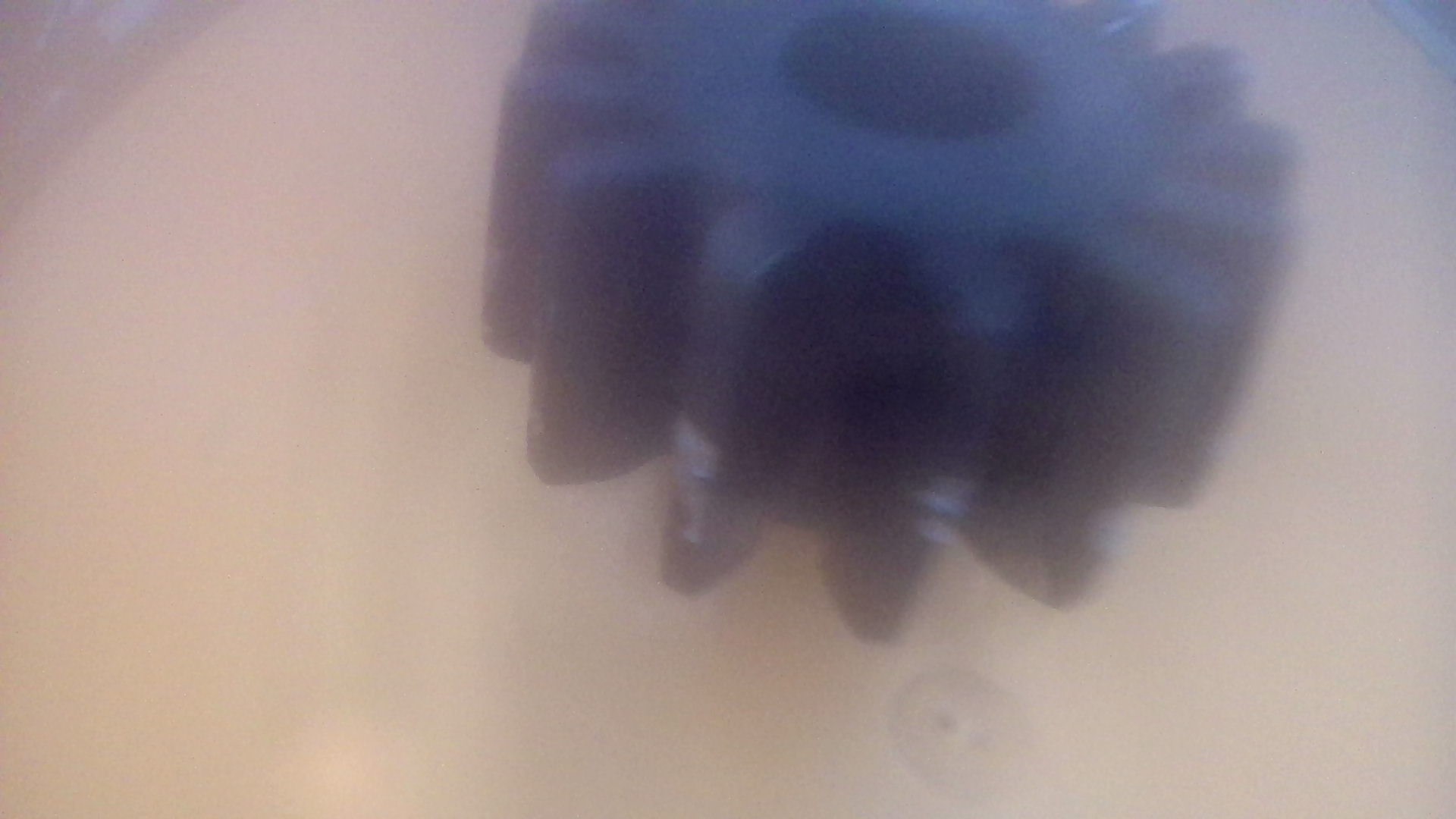

Supplement: S1 Data — (ZIP) [file pone.0322217.s001.zip › dataset/2/WIN_20250112_14_53_31_Pro.jpg]

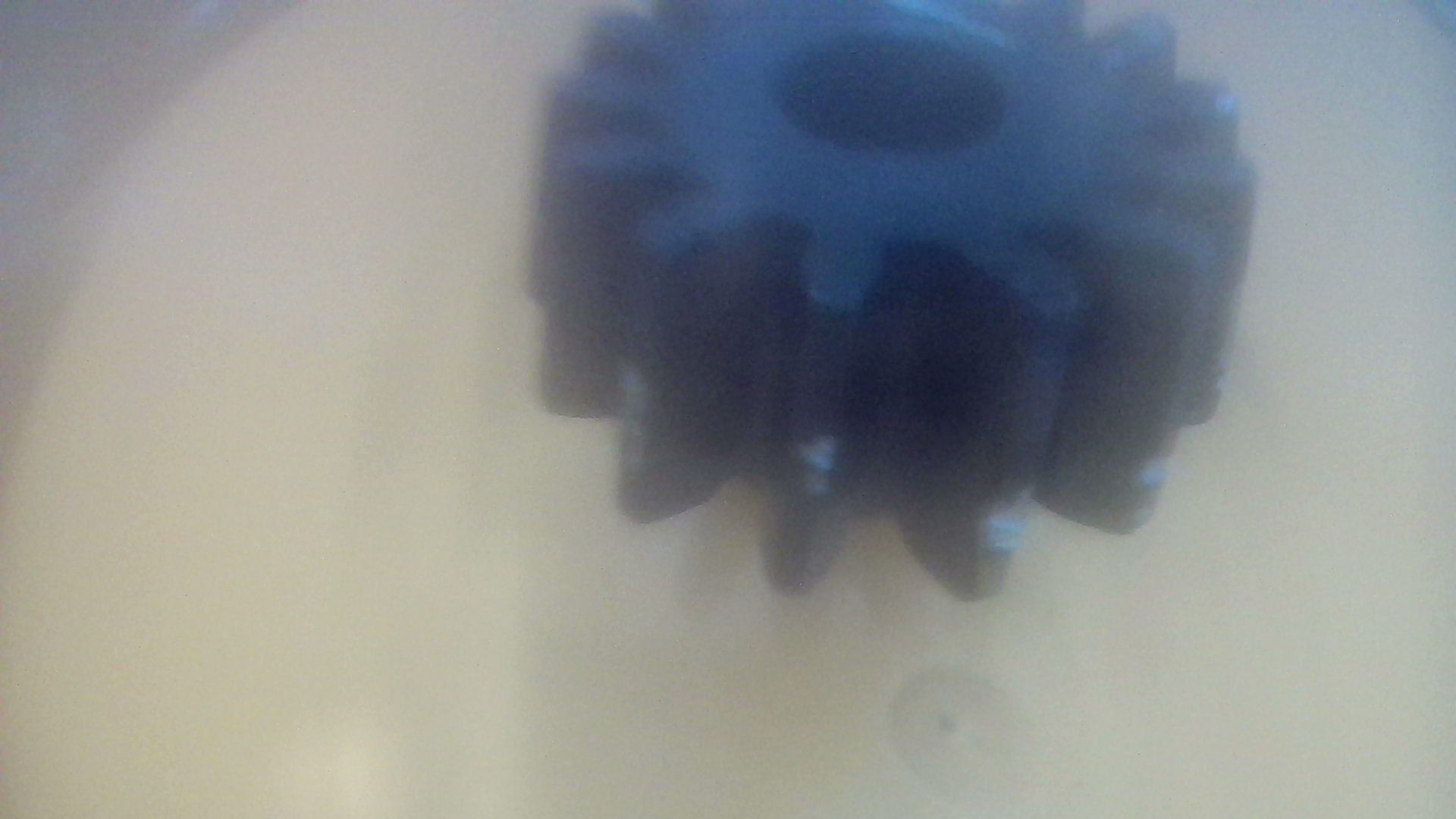

Supplement: S1 Data — (ZIP) [file pone.0322217.s001.zip › dataset/2/WIN_20250112_14_53_33_Pro.jpg]

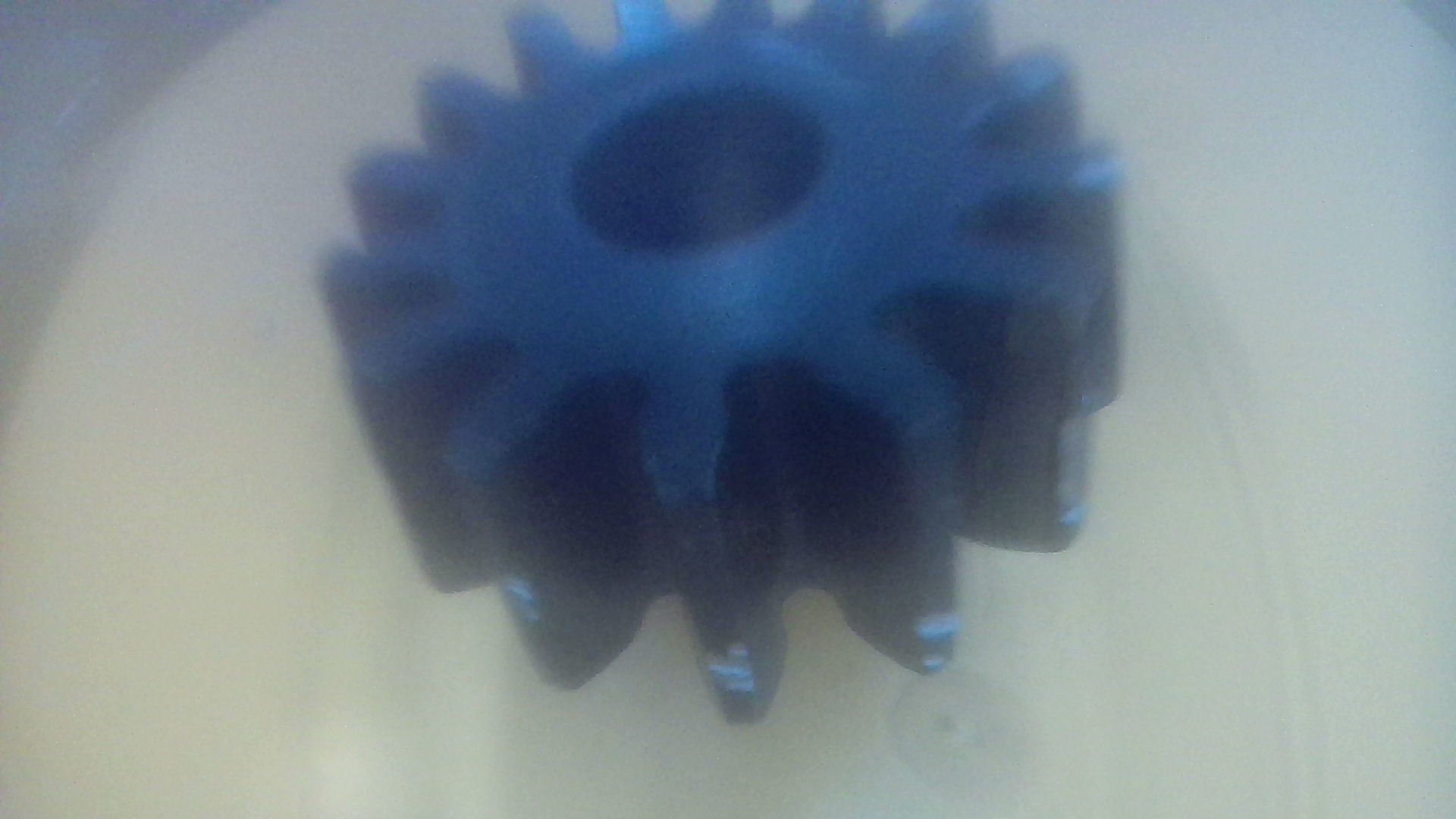

Supplement: S1 Data — (ZIP) [file pone.0322217.s001.zip › dataset/2/WIN_20250112_14_53_34_Pro.jpg]

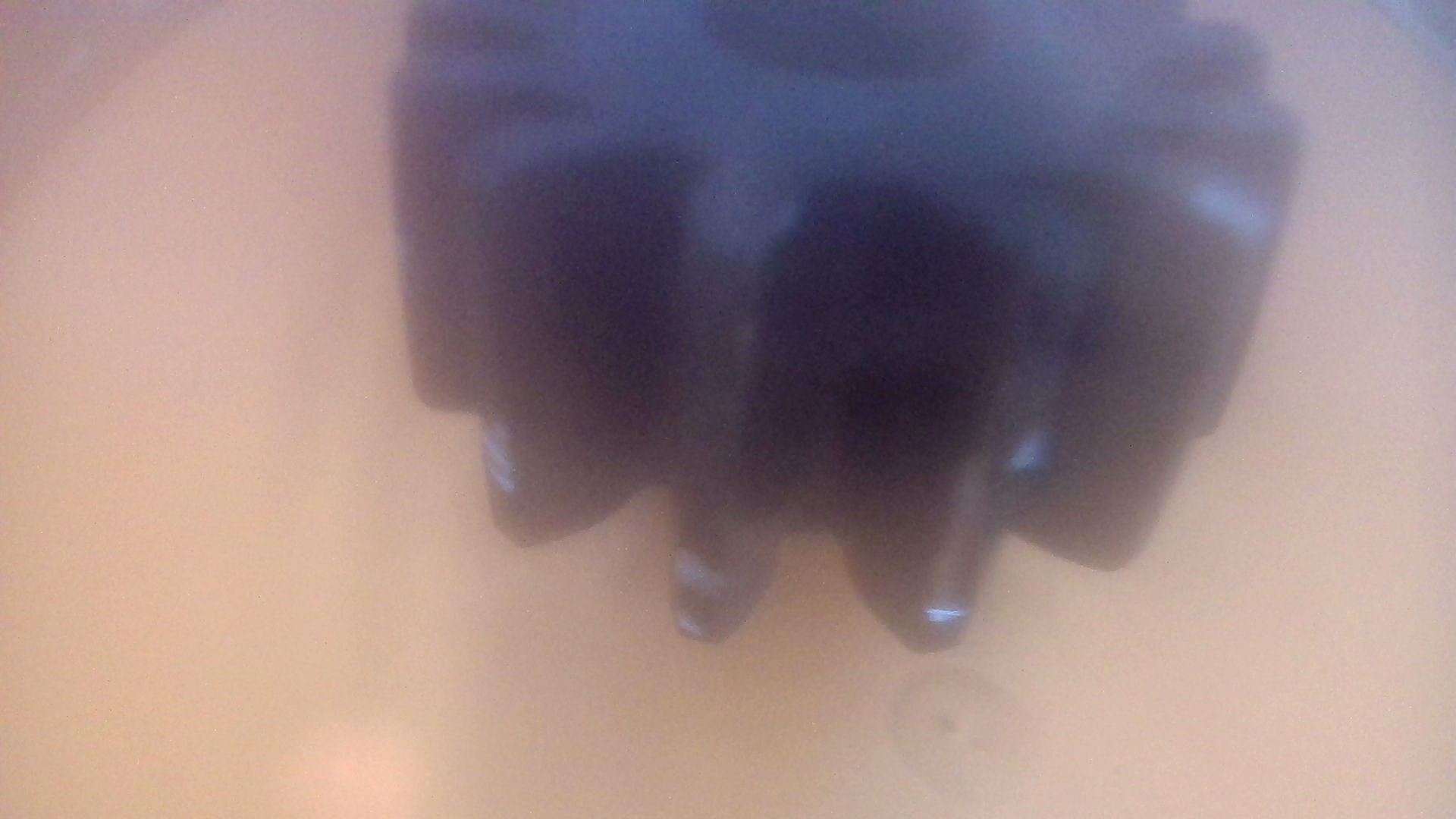

Supplement: S1 Data — (ZIP) [file pone.0322217.s001.zip › dataset/2/WIN_20250112_14_53_38_Pro.jpg]

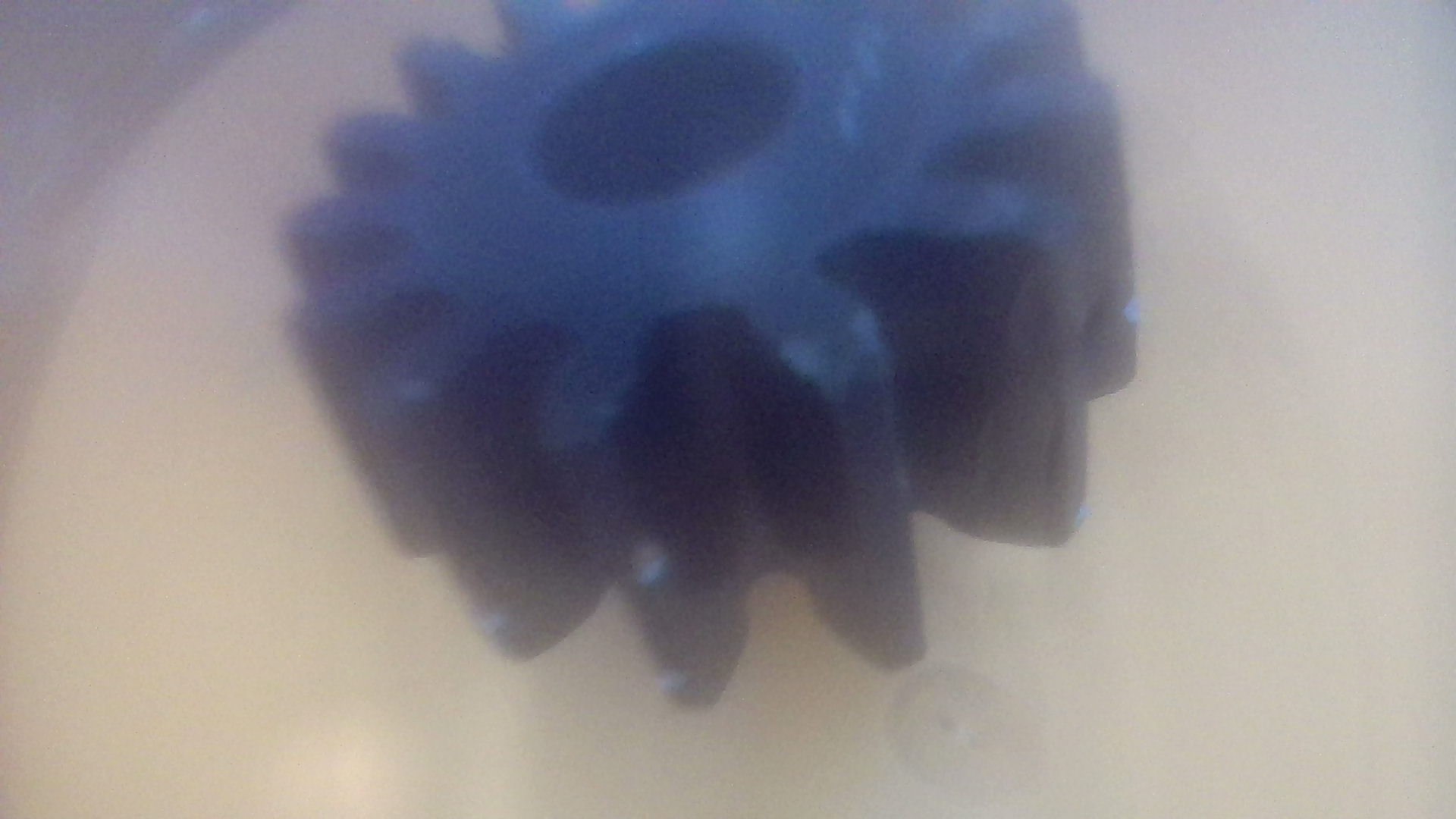

Supplement: S1 Data — (ZIP) [file pone.0322217.s001.zip › dataset/2/WIN_20250112_14_53_40_Pro.jpg]

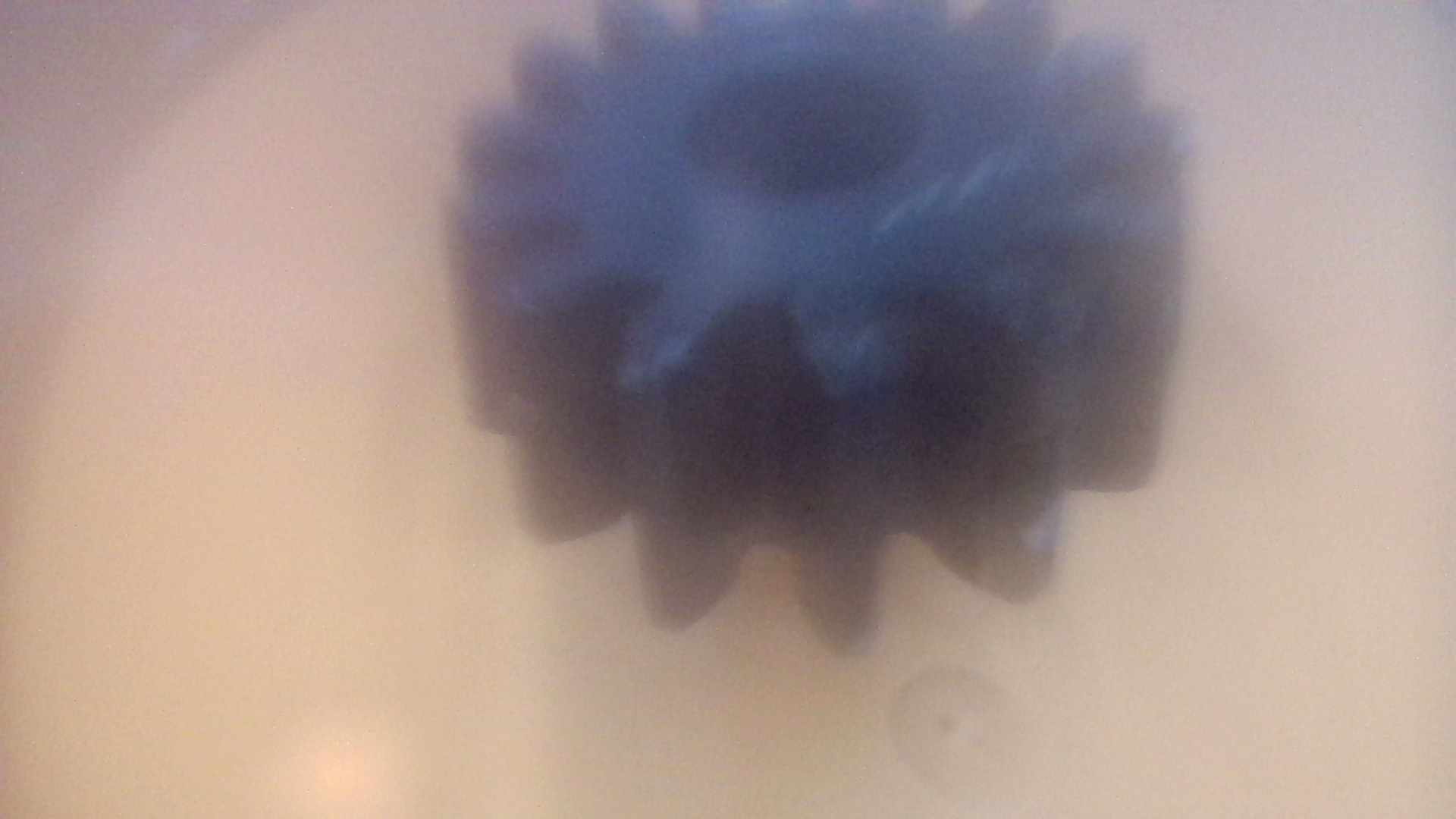

Supplement: S1 Data — (ZIP) [file pone.0322217.s001.zip › dataset/2/WIN_20250112_14_53_45_Pro.jpg]

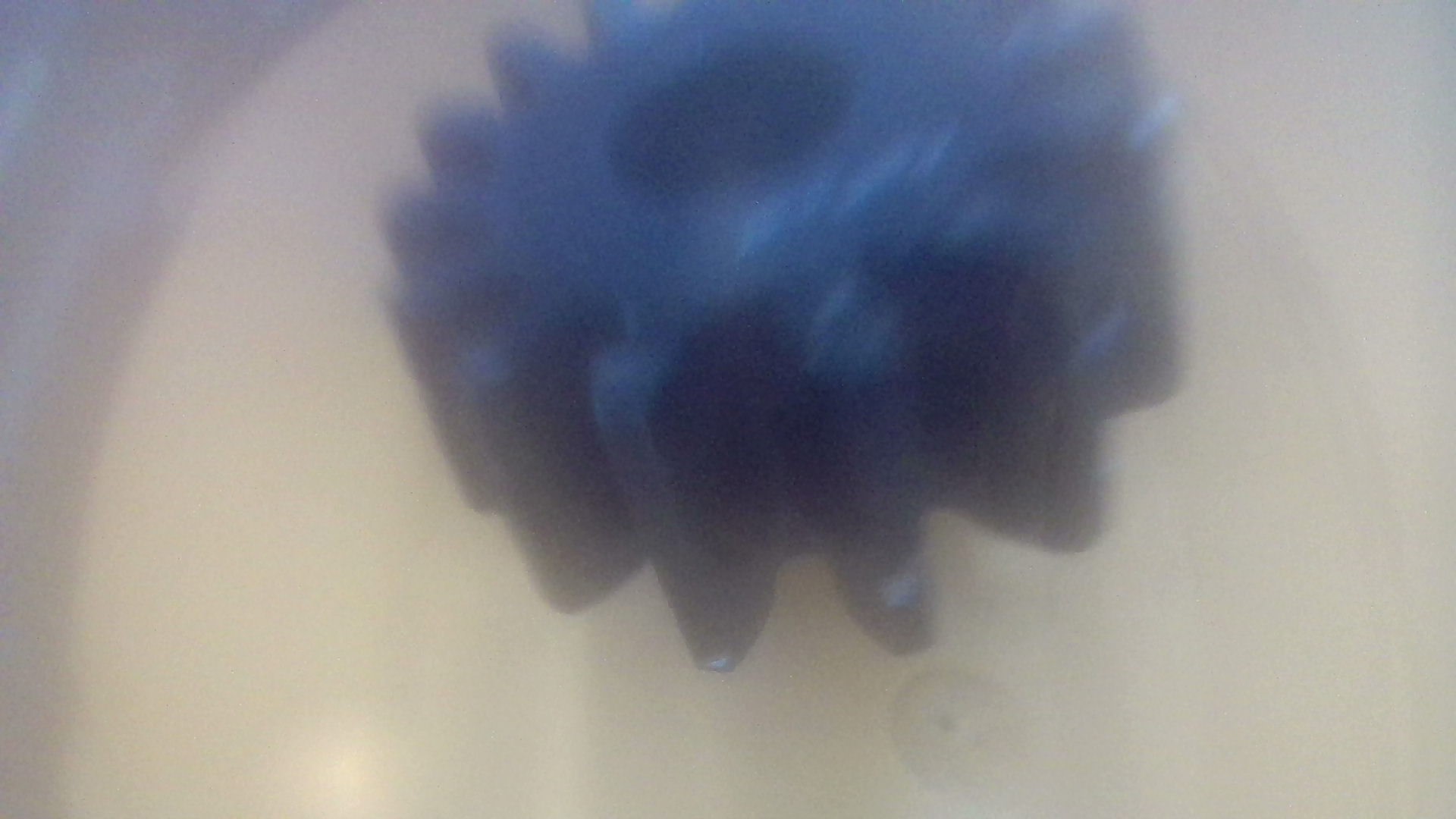

Supplement: S1 Data — (ZIP) [file pone.0322217.s001.zip › dataset/2/WIN_20250112_14_53_46_Pro.jpg]

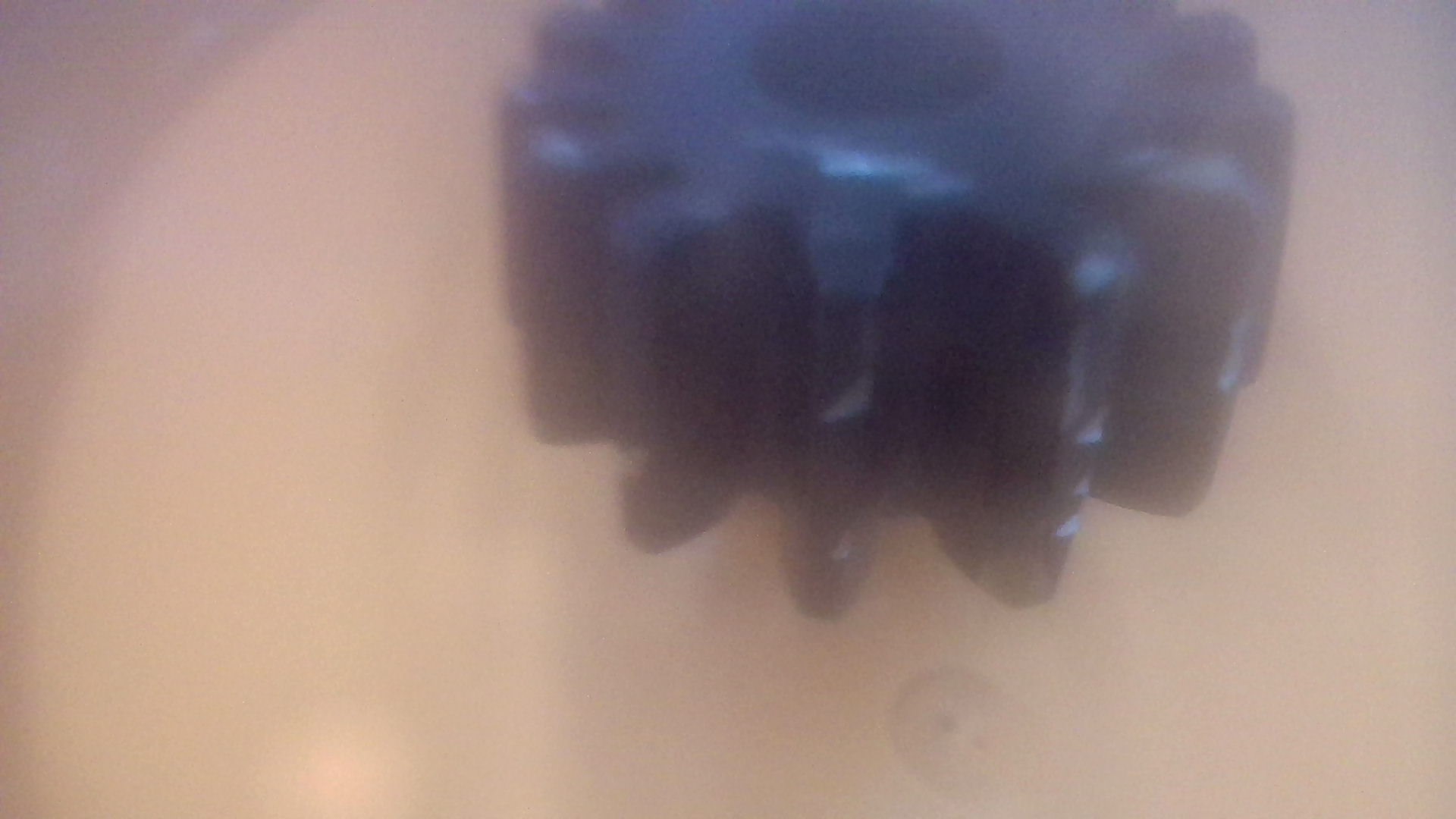

Supplement: S1 Data — (ZIP) [file pone.0322217.s001.zip › dataset/2/WIN_20250112_14_53_50_Pro.jpg]

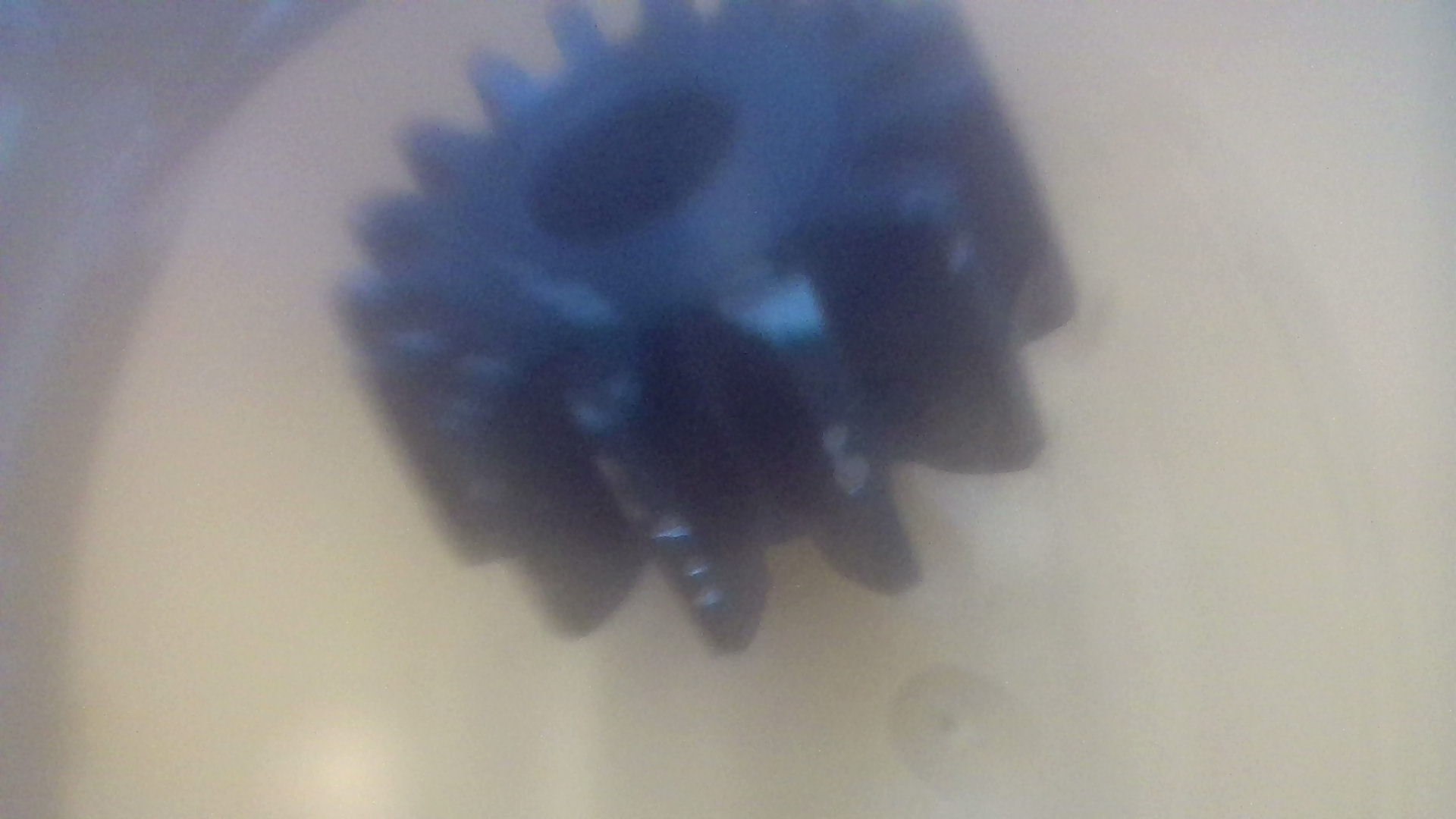

Supplement: S1 Data — (ZIP) [file pone.0322217.s001.zip › dataset/2/WIN_20250112_14_53_52_Pro.jpg]

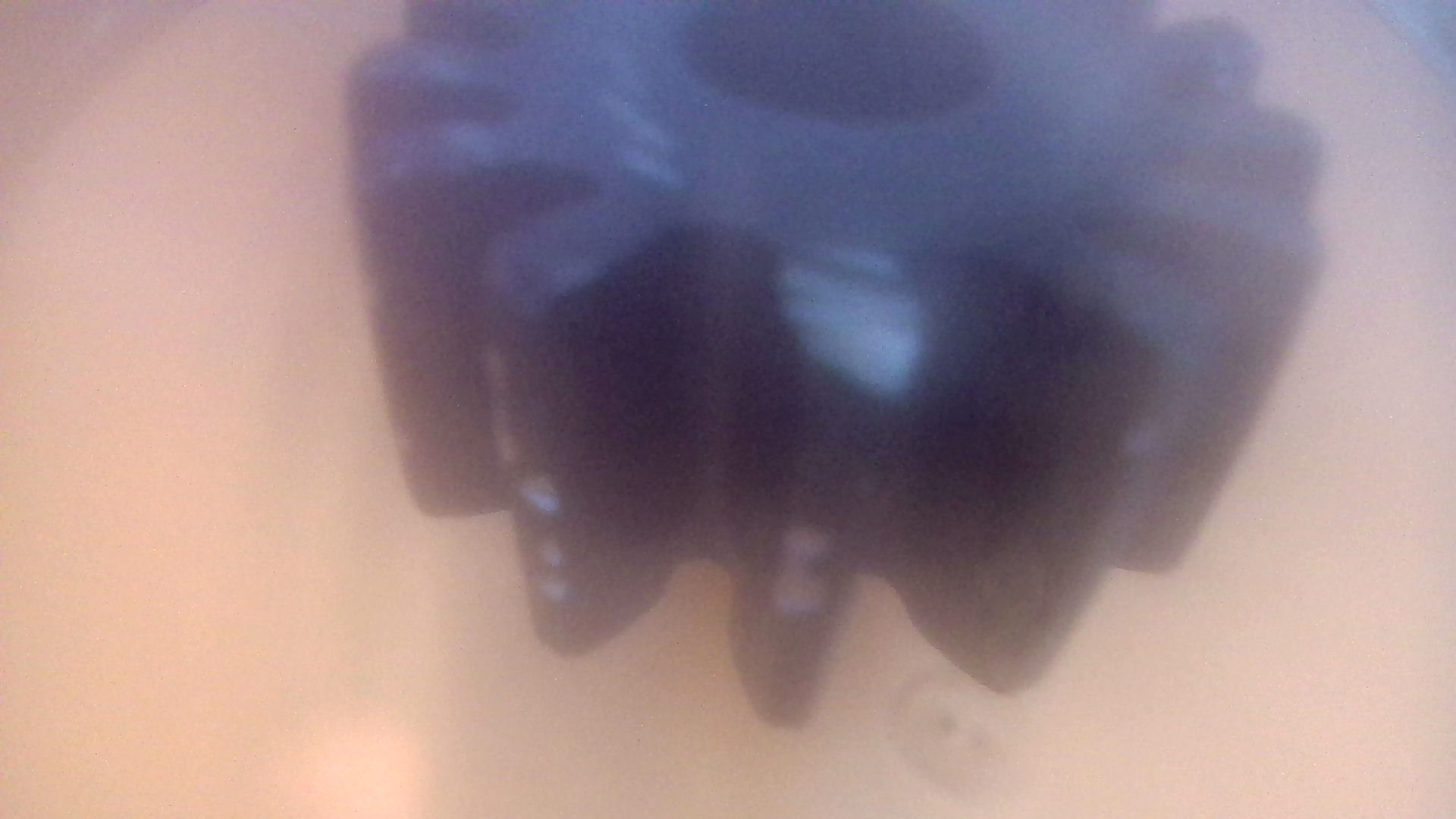

Supplement: S1 Data — (ZIP) [file pone.0322217.s001.zip › dataset/2/WIN_20250112_14_53_56_Pro.jpg]

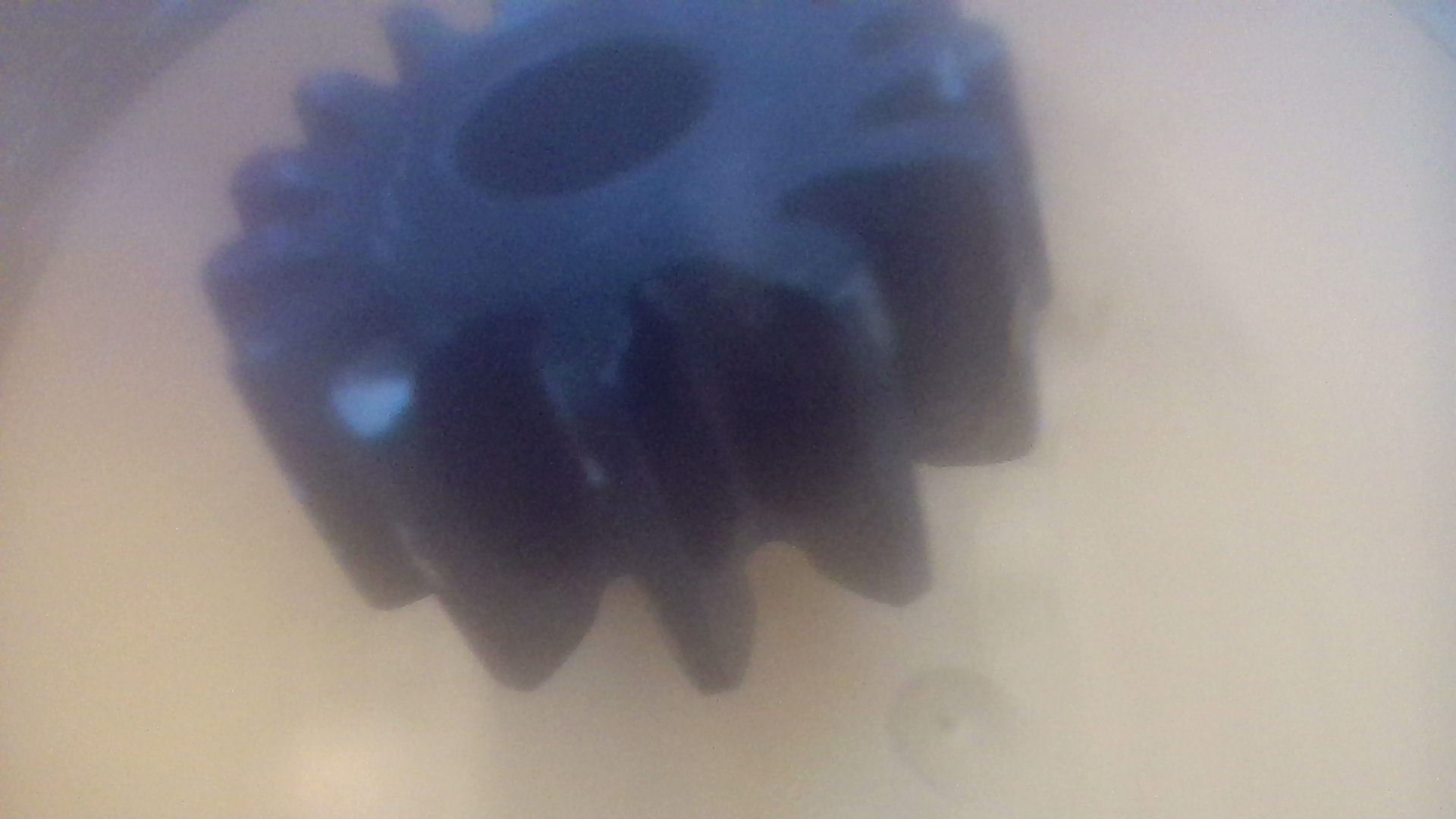

Supplement: S1 Data — (ZIP) [file pone.0322217.s001.zip › dataset/2/WIN_20250112_14_53_57_Pro.jpg]

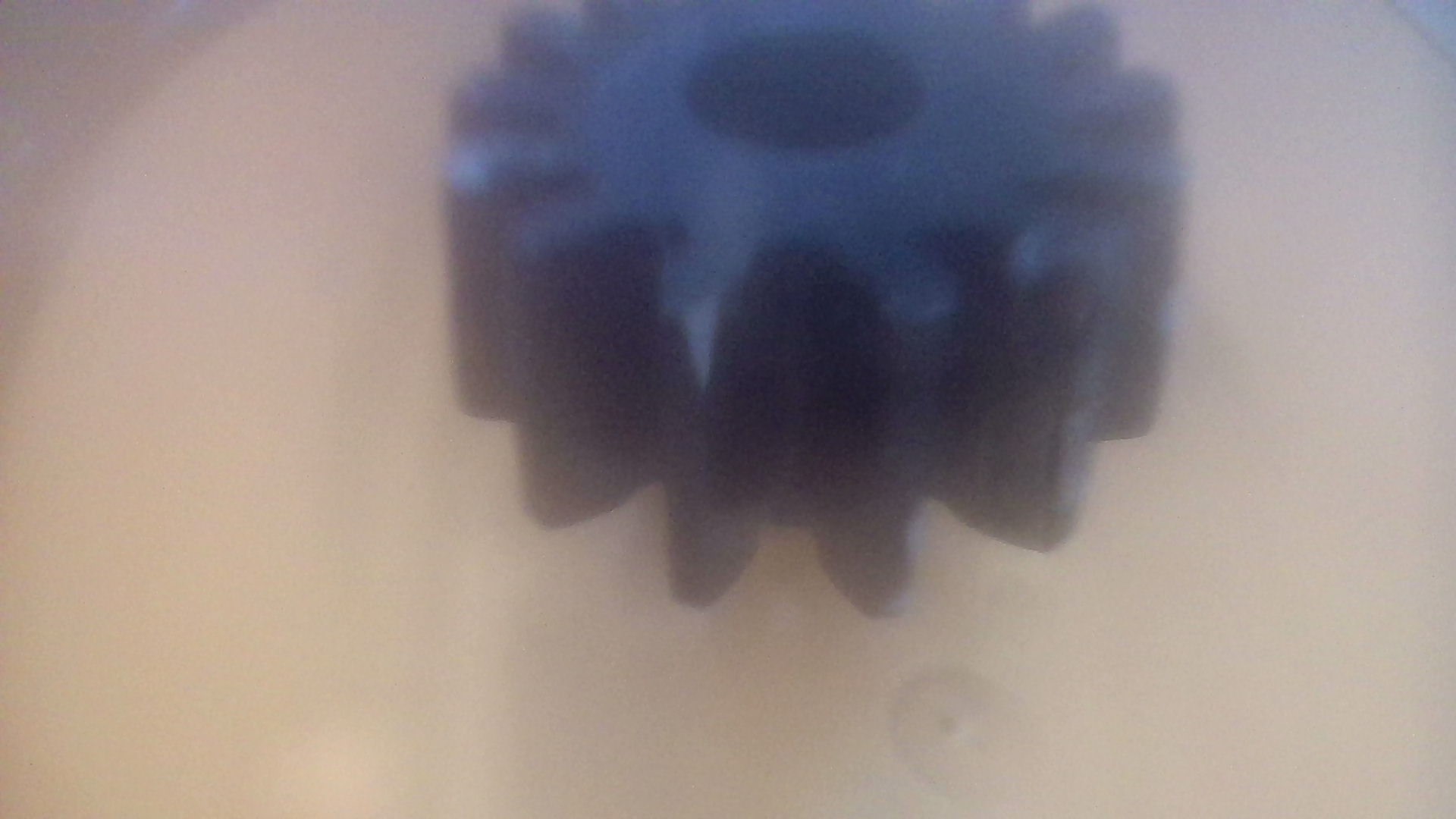

Supplement: S1 Data — (ZIP) [file pone.0322217.s001.zip › dataset/2/WIN_20250112_14_54_01_Pro.jpg]

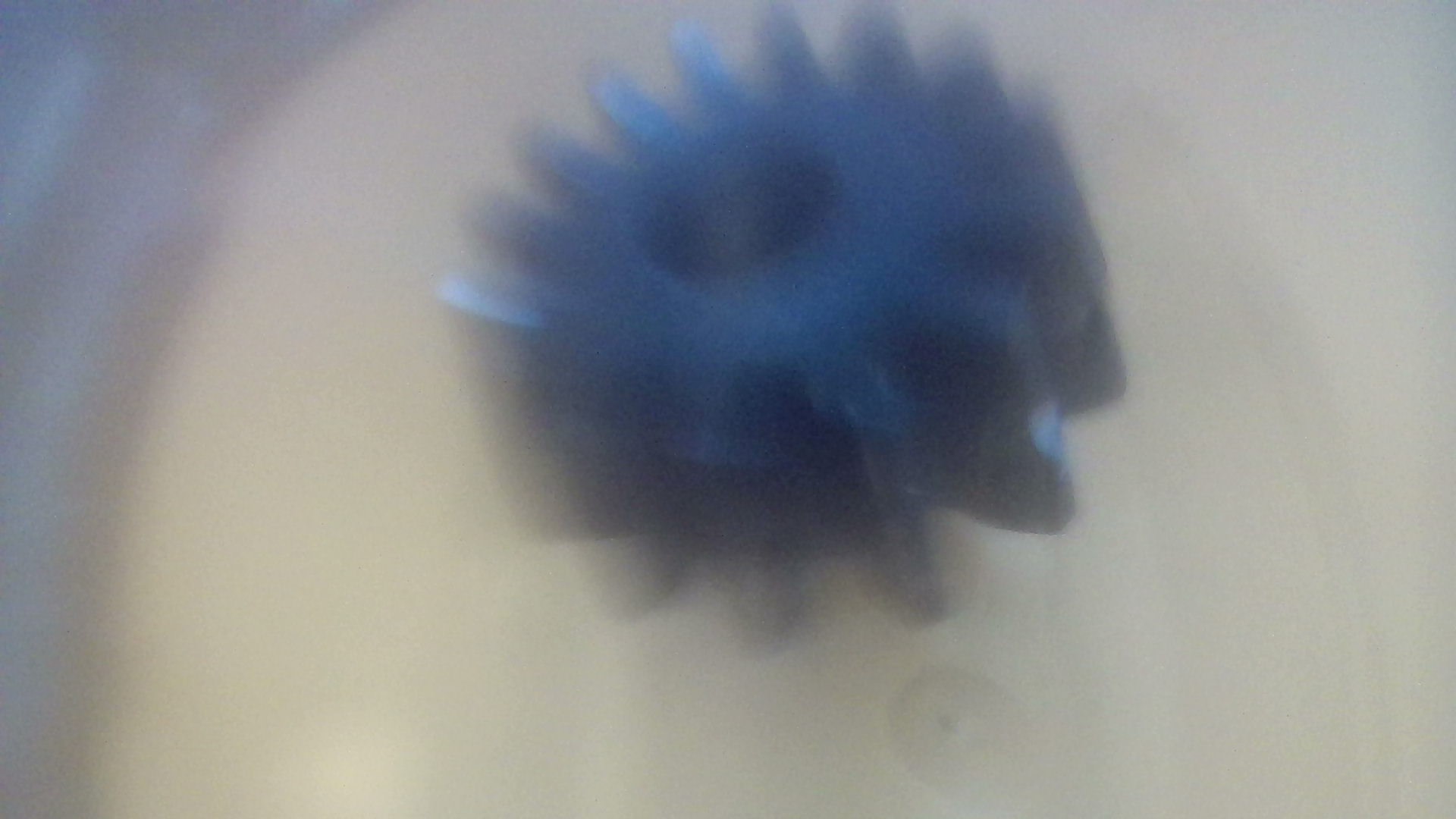

Supplement: S1 Data — (ZIP) [file pone.0322217.s001.zip › dataset/2/WIN_20250112_14_54_03_Pro.jpg]

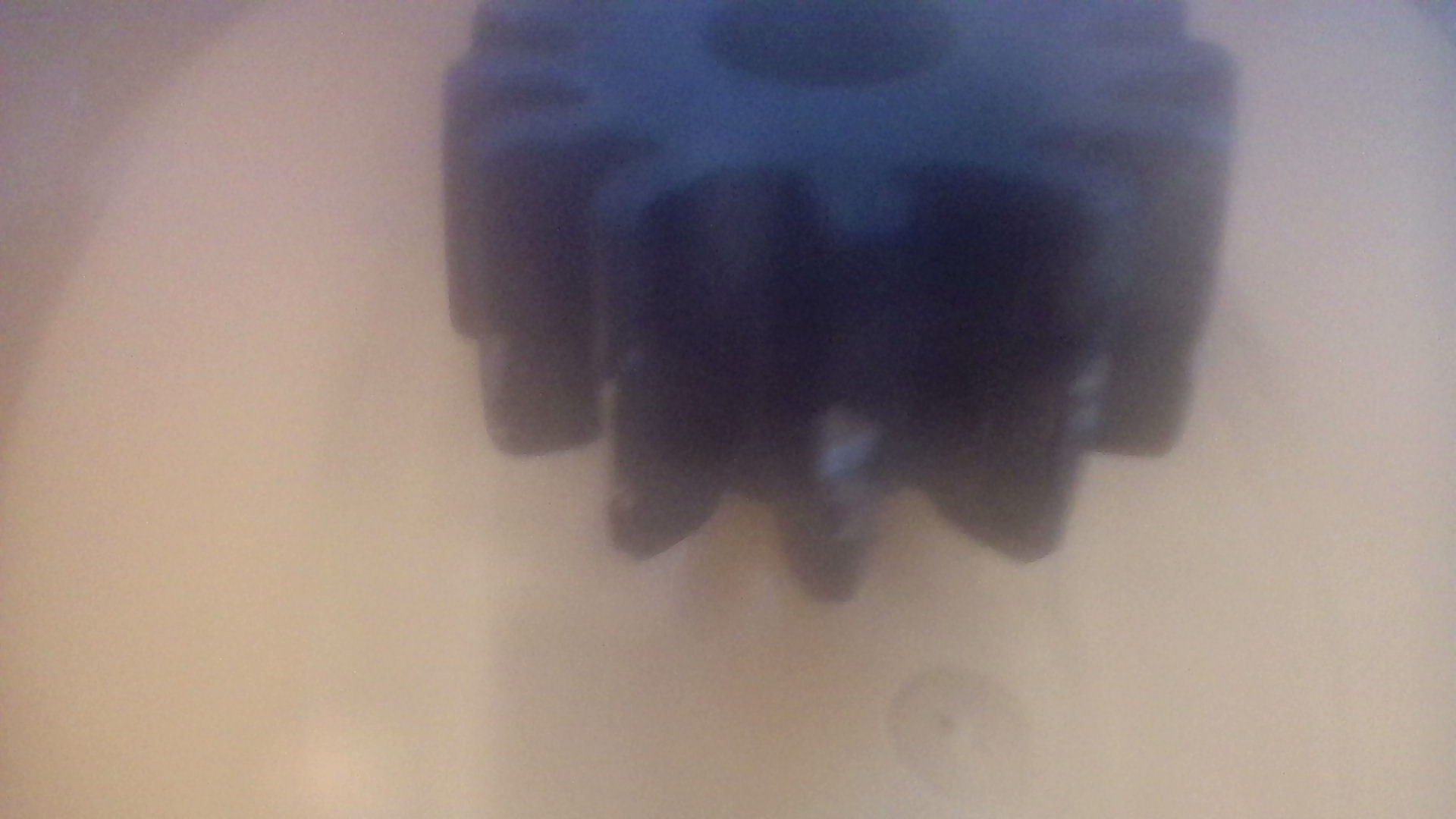

Supplement: S1 Data — (ZIP) [file pone.0322217.s001.zip › dataset/2/WIN_20250112_14_54_07_Pro.jpg]

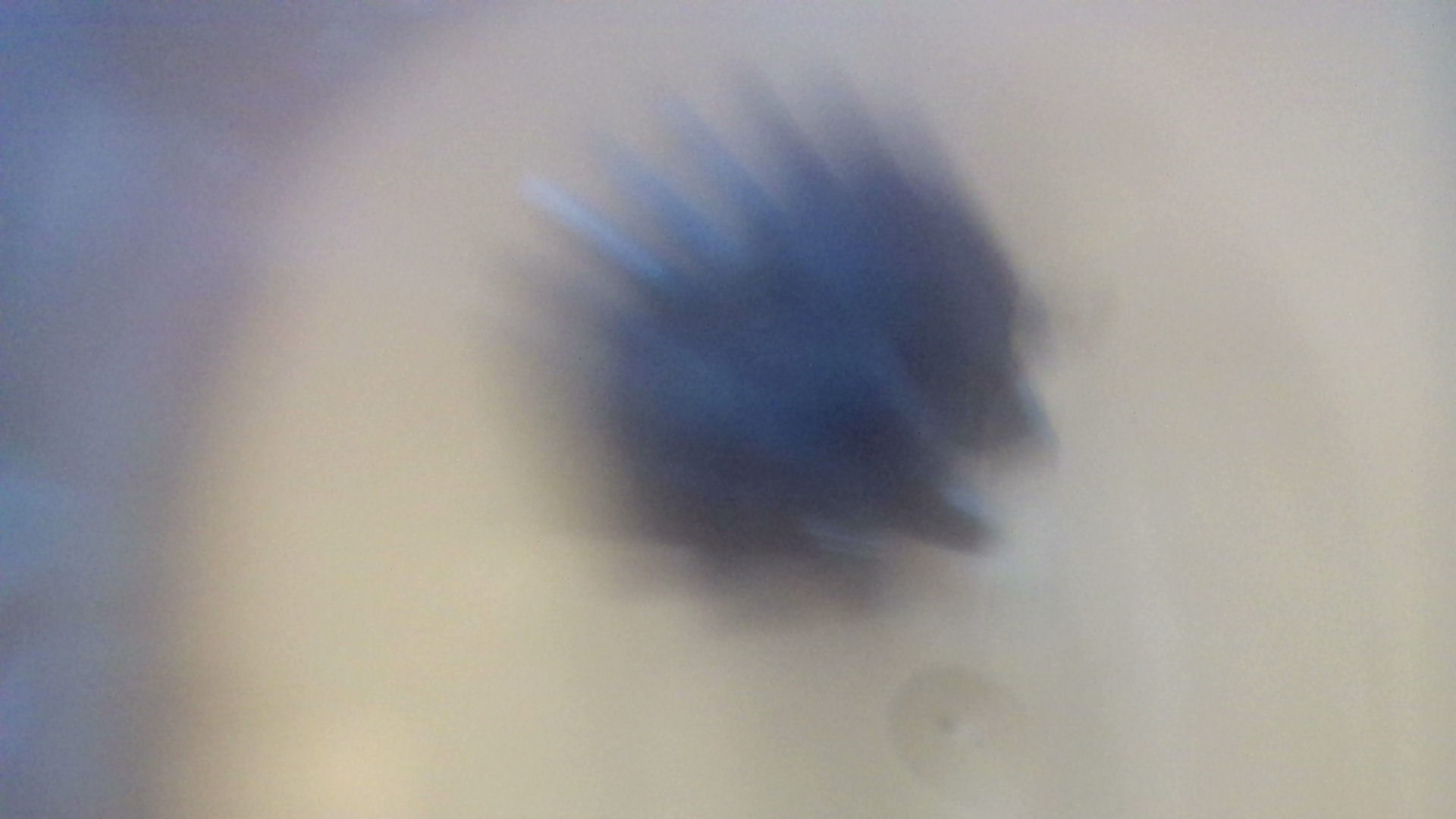

Supplement: S1 Data — (ZIP) [file pone.0322217.s001.zip › dataset/2/WIN_20250112_14_54_08_Pro.jpg]

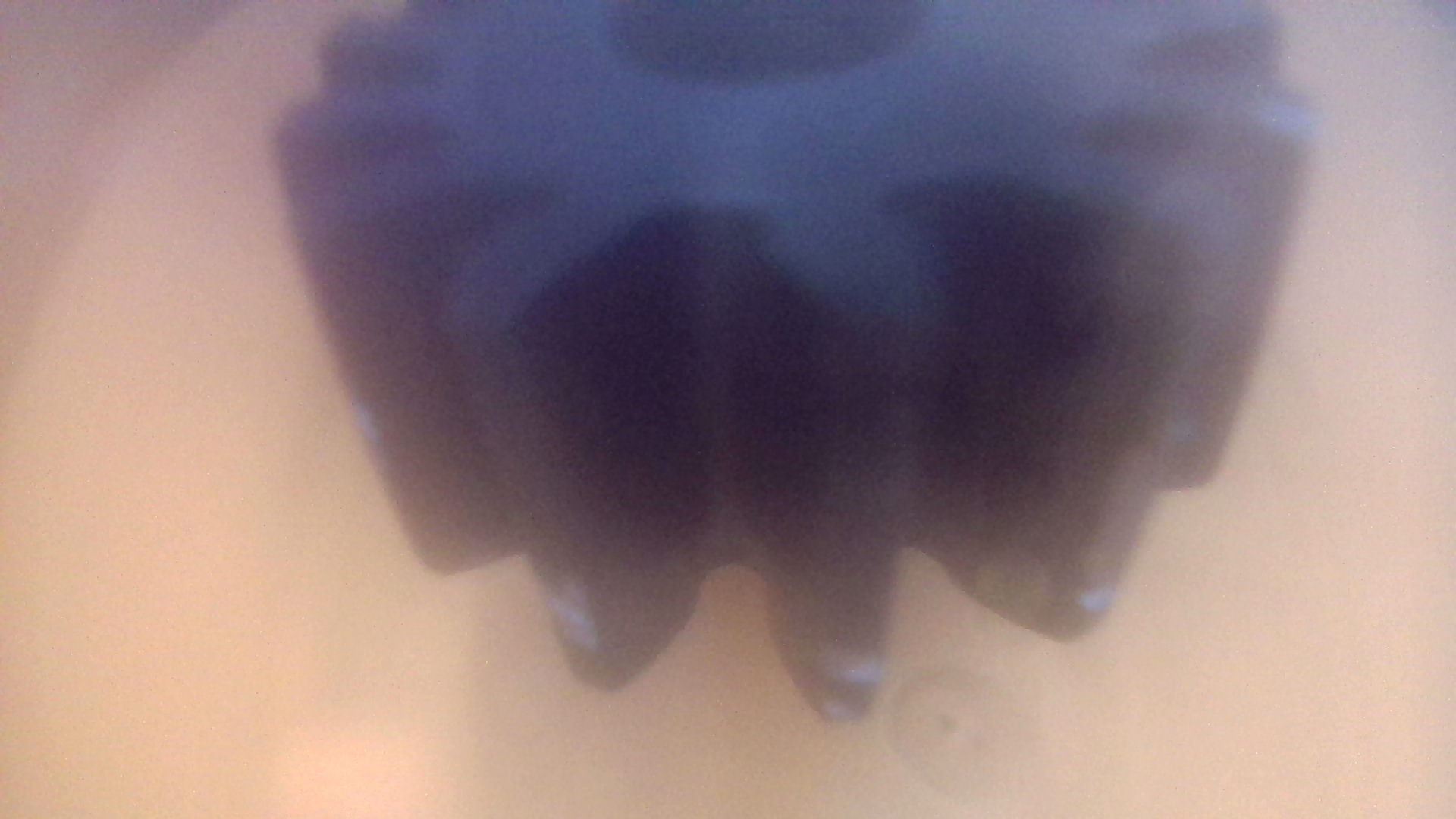

Supplement: S1 Data — (ZIP) [file pone.0322217.s001.zip › dataset/2/WIN_20250112_14_54_11_Pro.jpg]

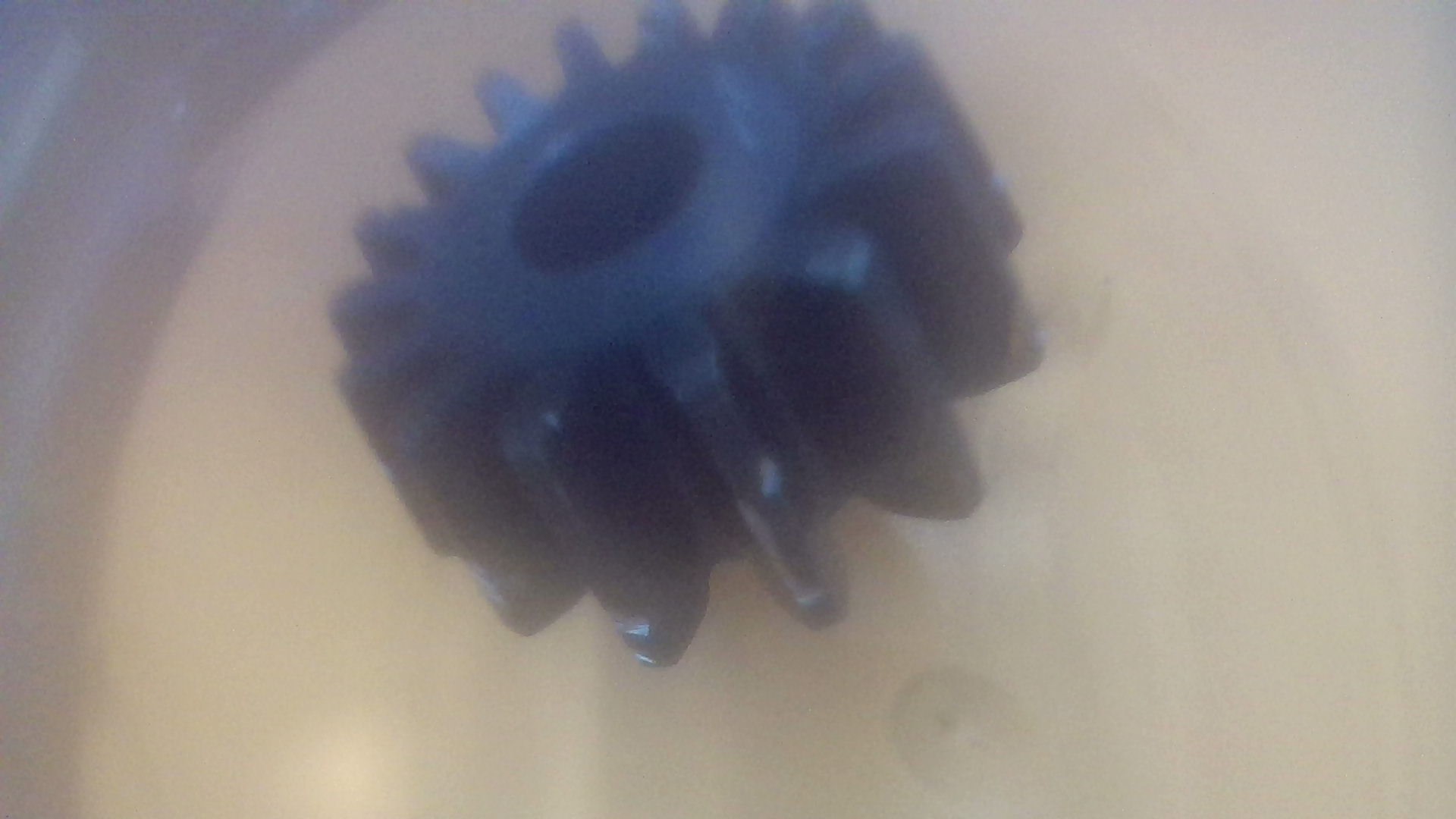

Supplement: S1 Data — (ZIP) [file pone.0322217.s001.zip › dataset/2/WIN_20250112_14_54_12_Pro.jpg]

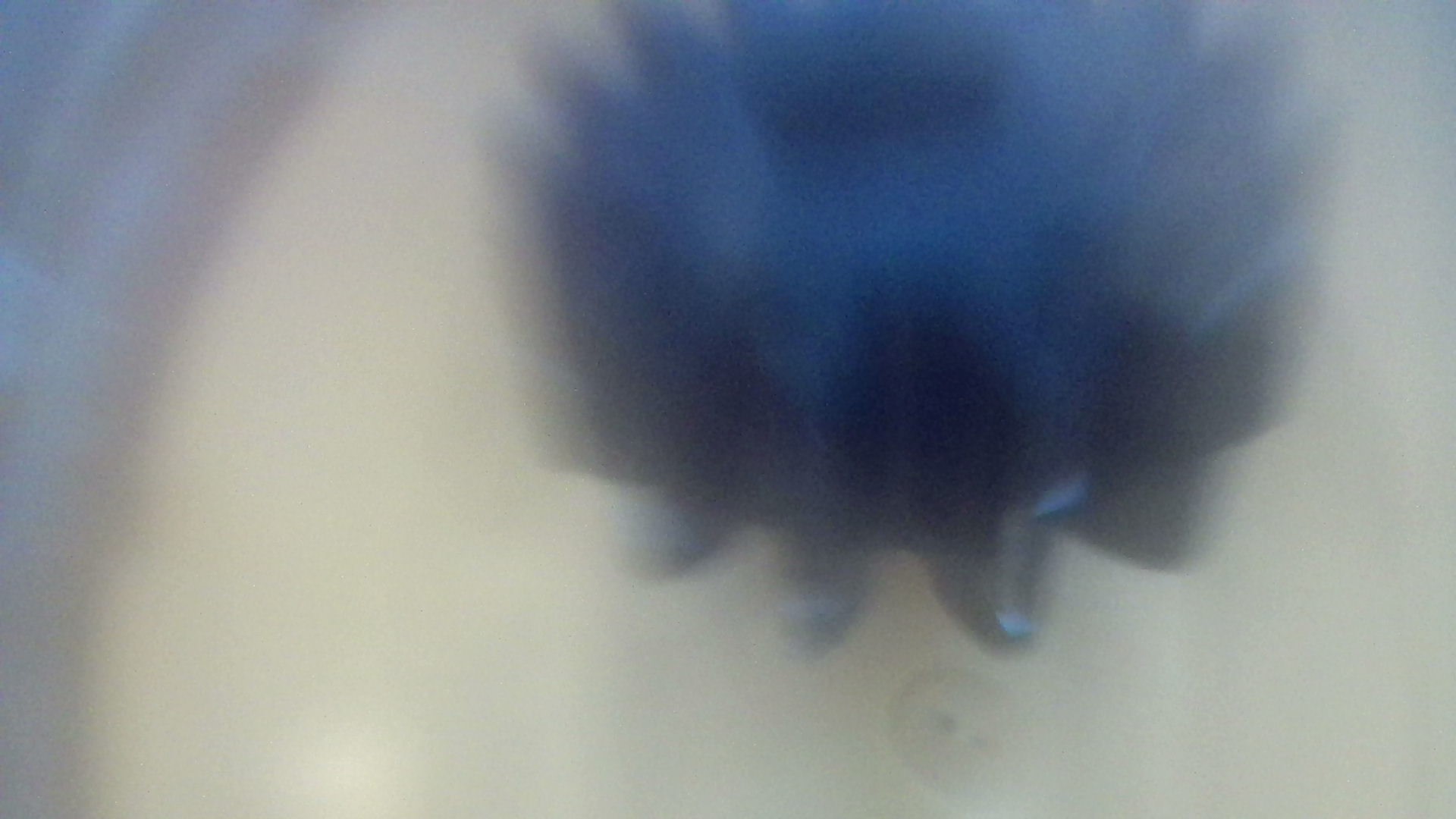

Supplement: S1 Data — (ZIP) [file pone.0322217.s001.zip › dataset/2/WIN_20250112_14_54_14_Pro.jpg]

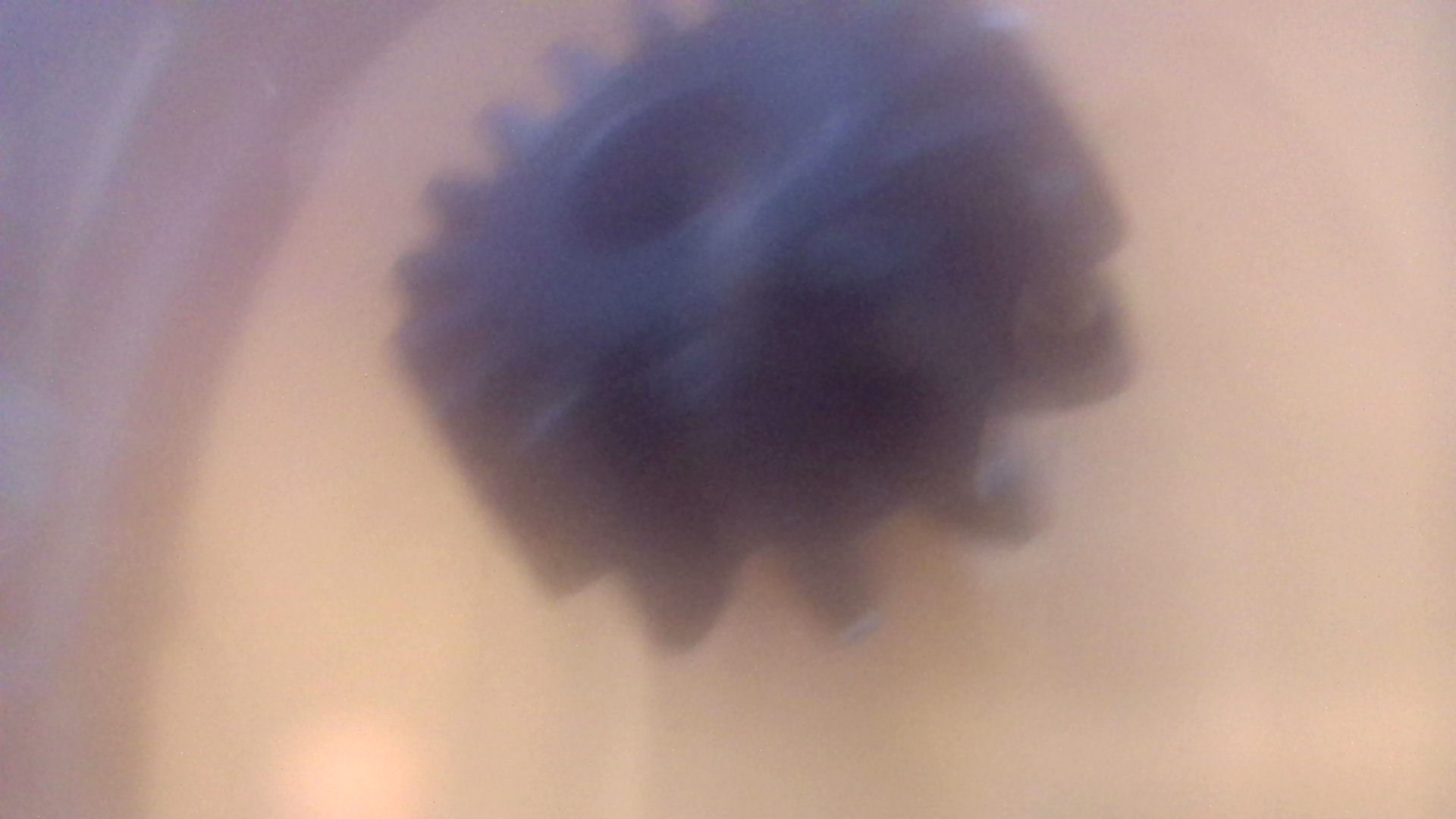

Supplement: S1 Data — (ZIP) [file pone.0322217.s001.zip › dataset/2/WIN_20250112_14_54_18_Pro.jpg]

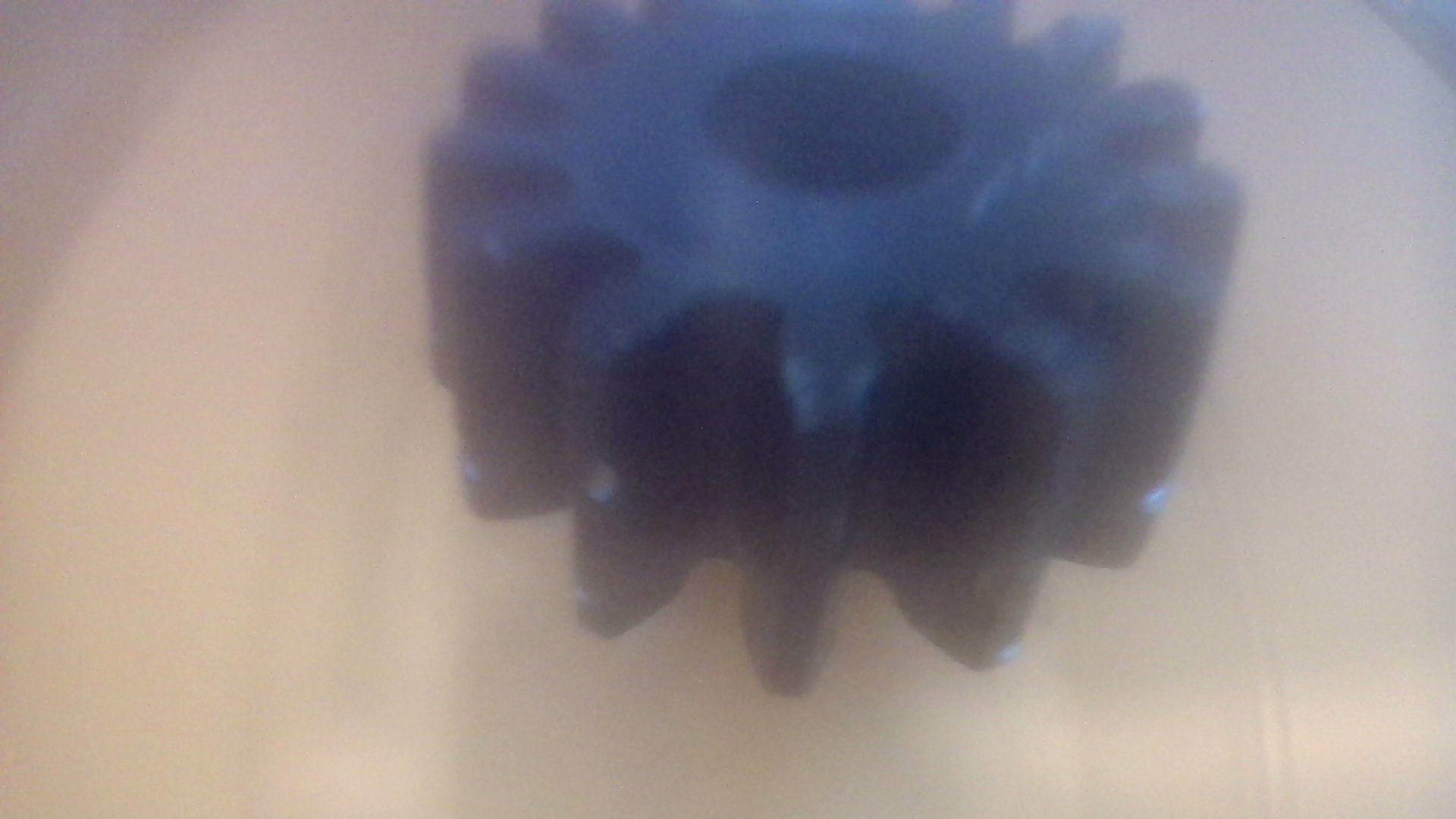

Supplement: S1 Data — (ZIP) [file pone.0322217.s001.zip › dataset/2/WIN_20250112_14_54_19_Pro.jpg]

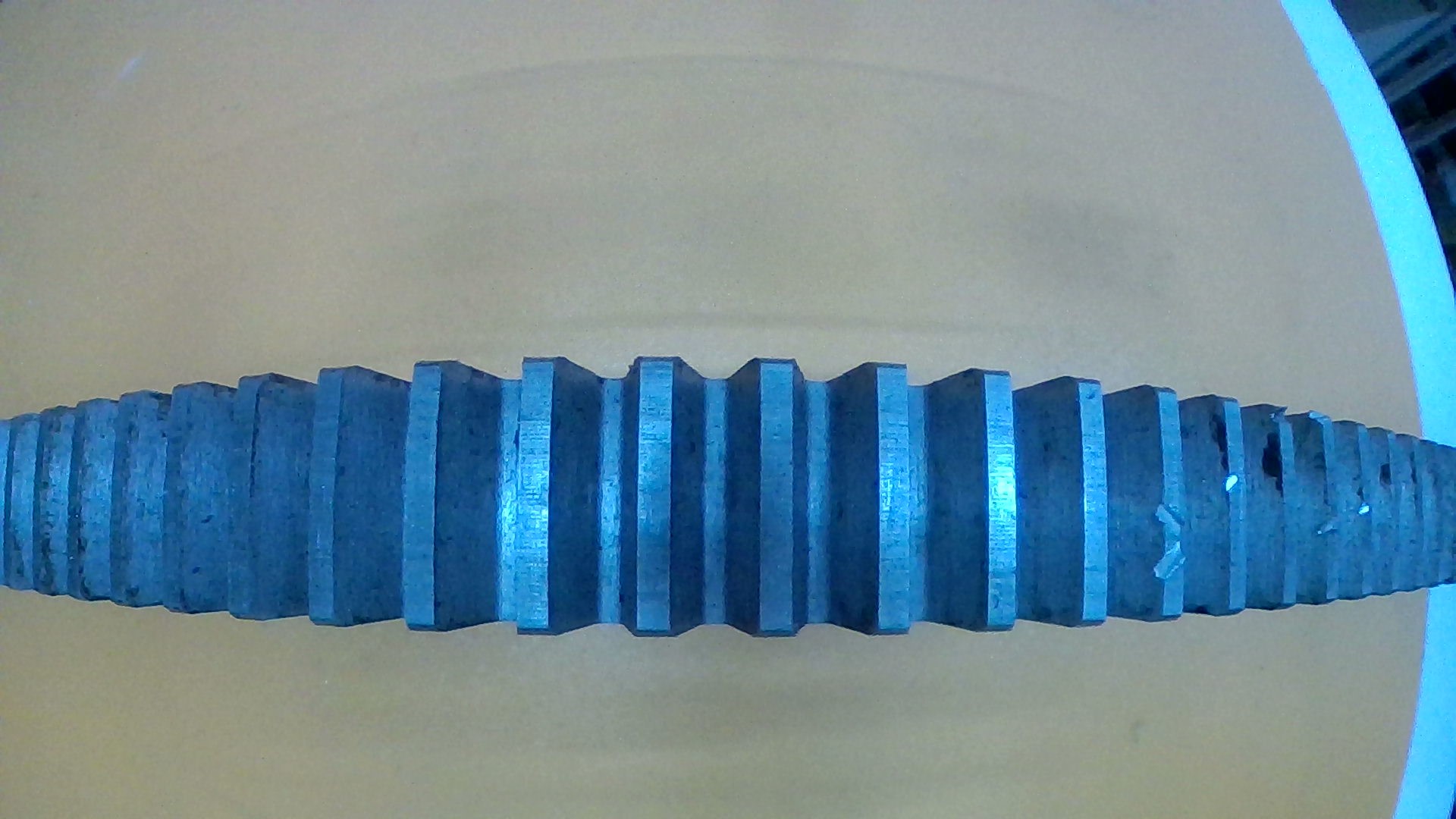

Supplement: S1 Data — (ZIP) [file pone.0322217.s001.zip › dataset/3/WIN_20250111_19_16_08_Pro.jpg]

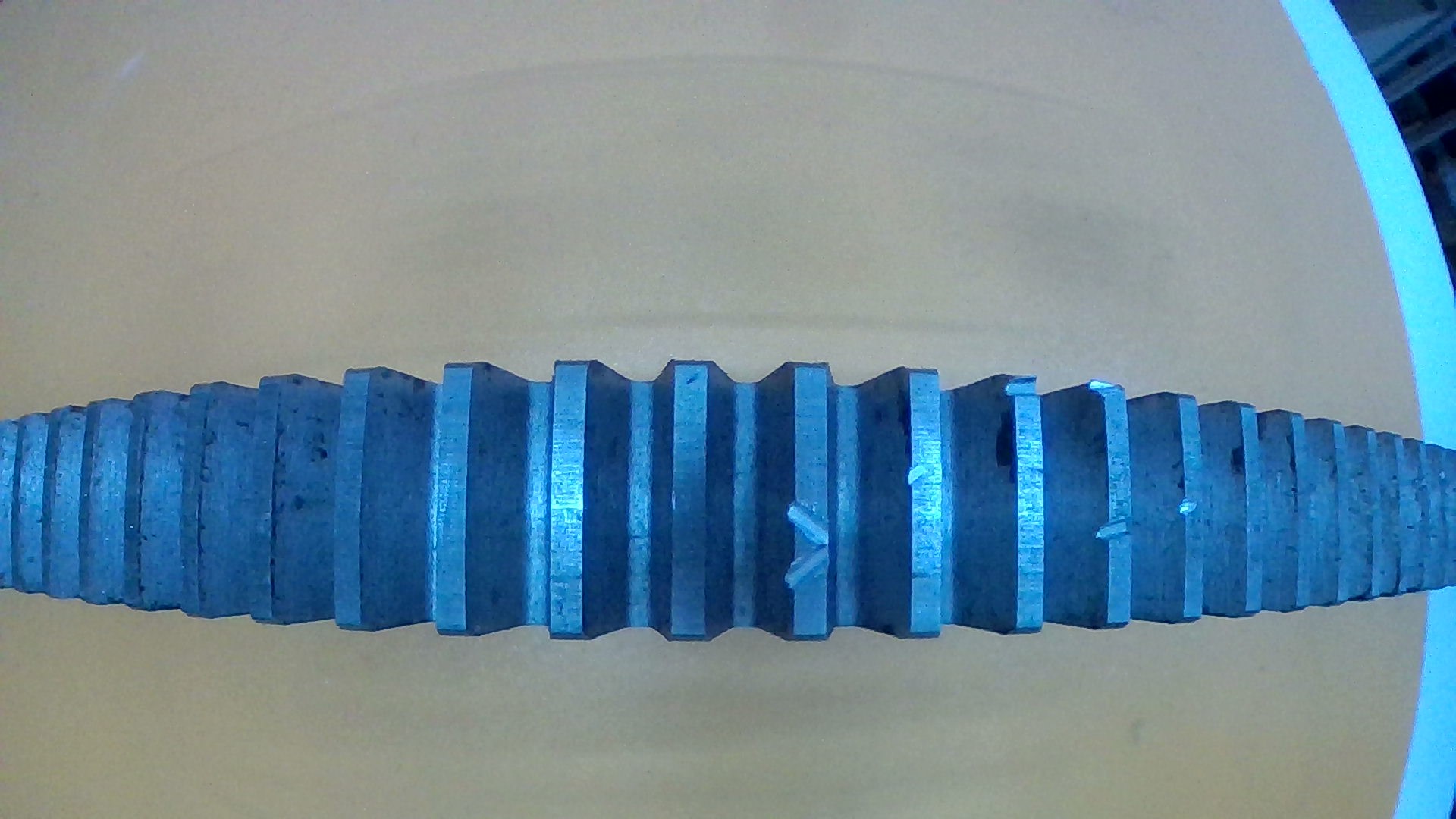

Supplement: S1 Data — (ZIP) [file pone.0322217.s001.zip › dataset/3/WIN_20250111_19_16_17_Pro.jpg]

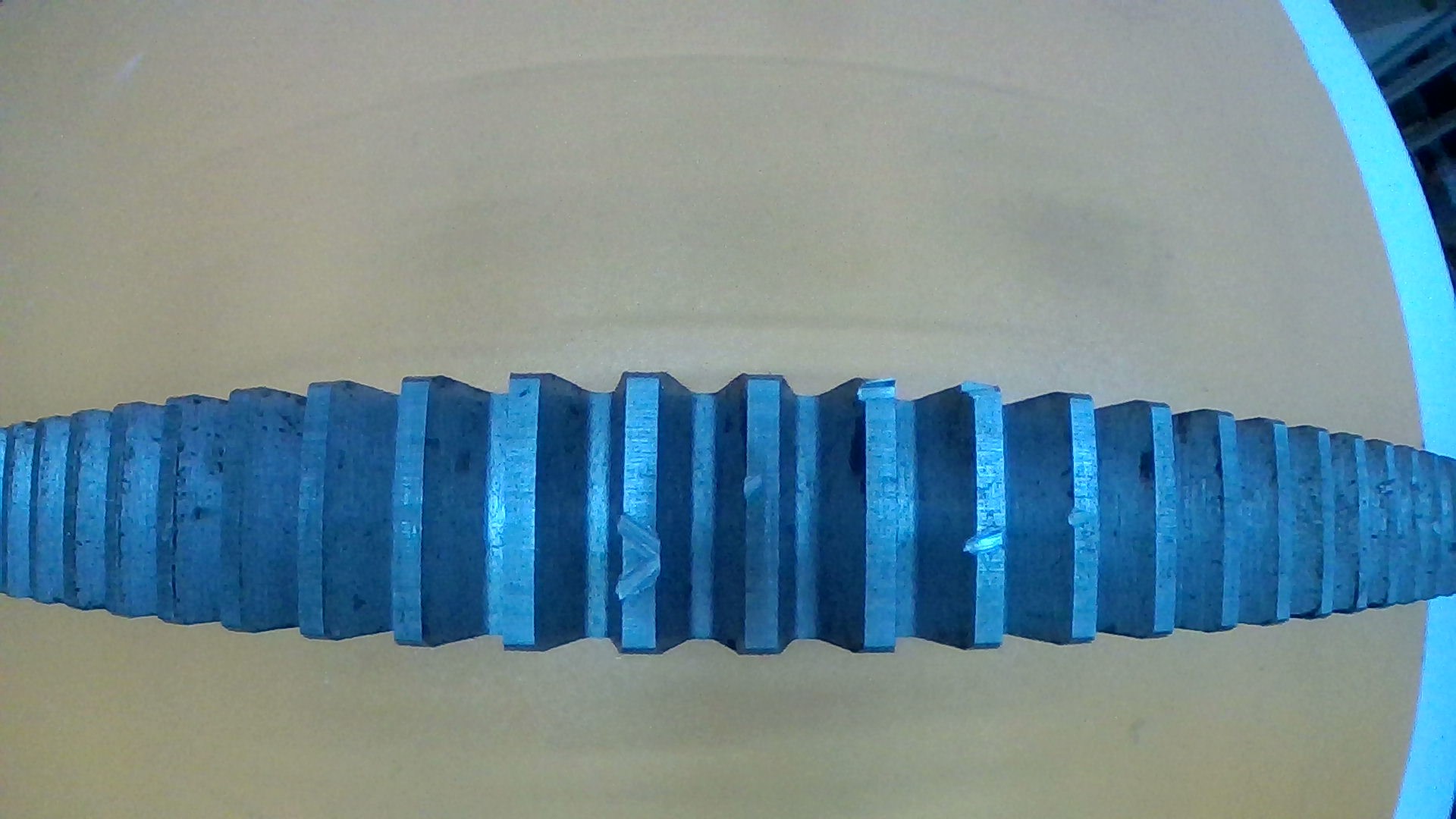

Supplement: S1 Data — (ZIP) [file pone.0322217.s001.zip › dataset/3/WIN_20250111_19_16_20_Pro.jpg]

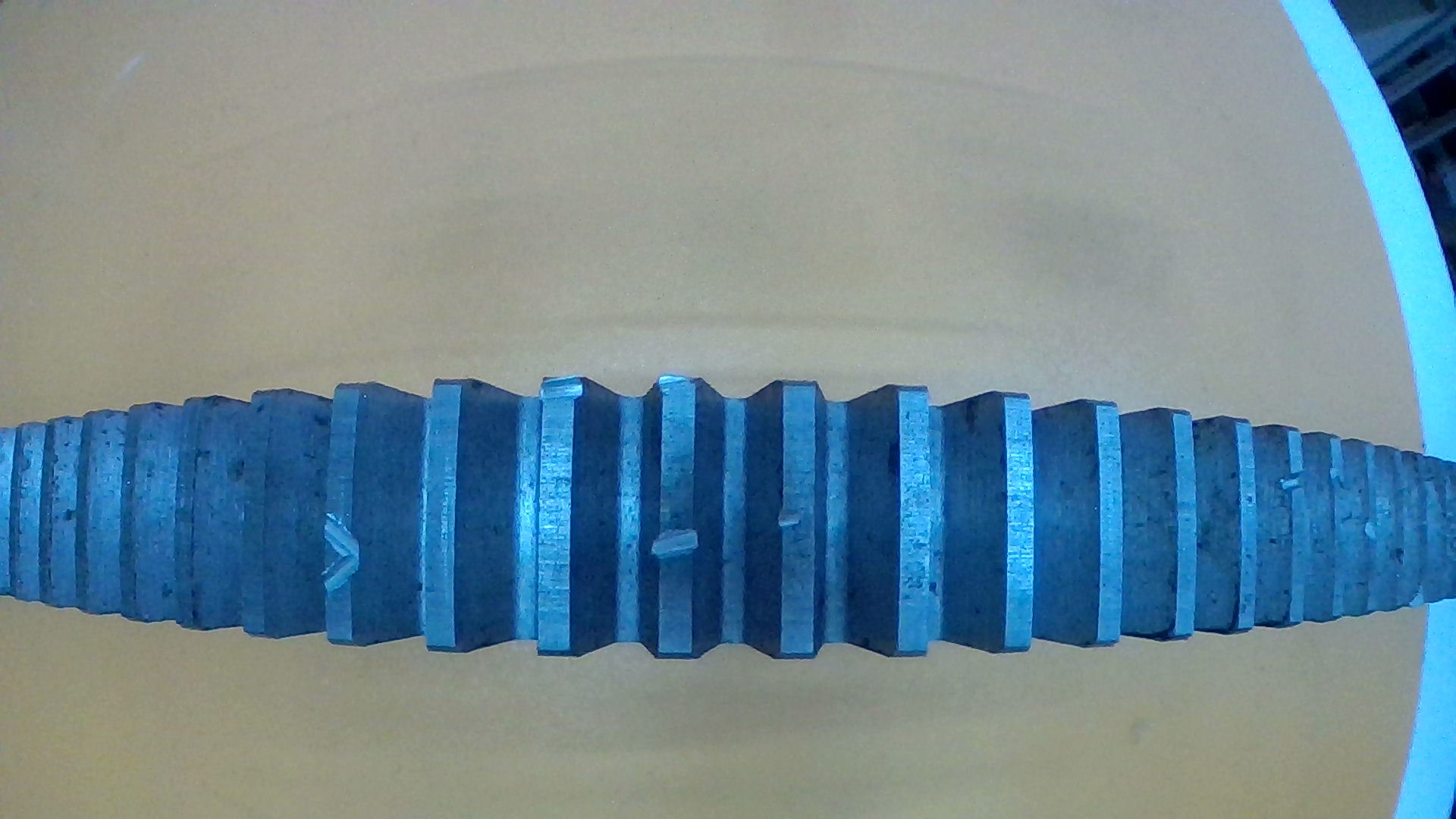

Supplement: S1 Data — (ZIP) [file pone.0322217.s001.zip › dataset/3/WIN_20250111_19_16_24_Pro.jpg]

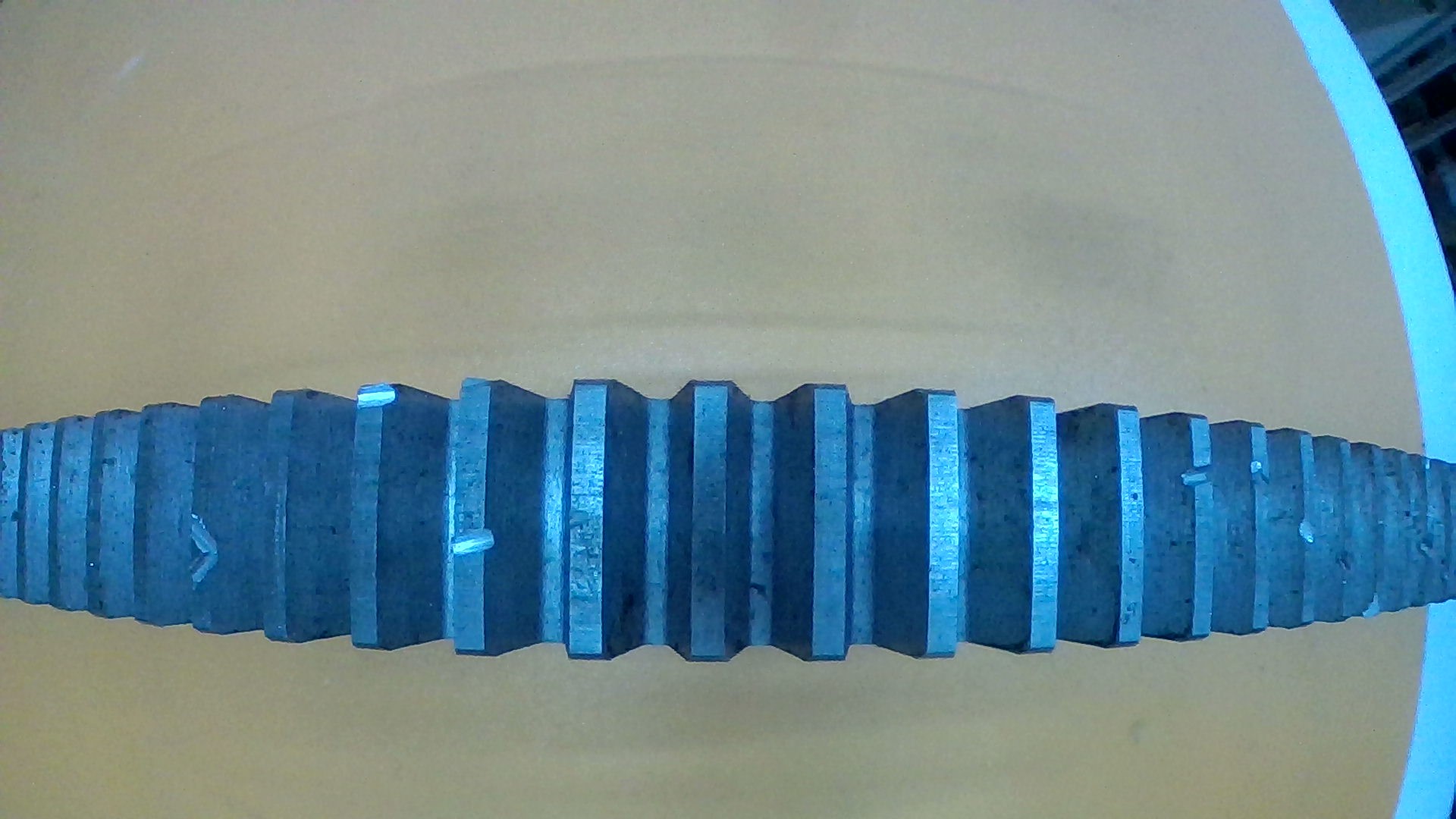

Supplement: S1 Data — (ZIP) [file pone.0322217.s001.zip › dataset/3/WIN_20250111_19_16_27_Pro.jpg]

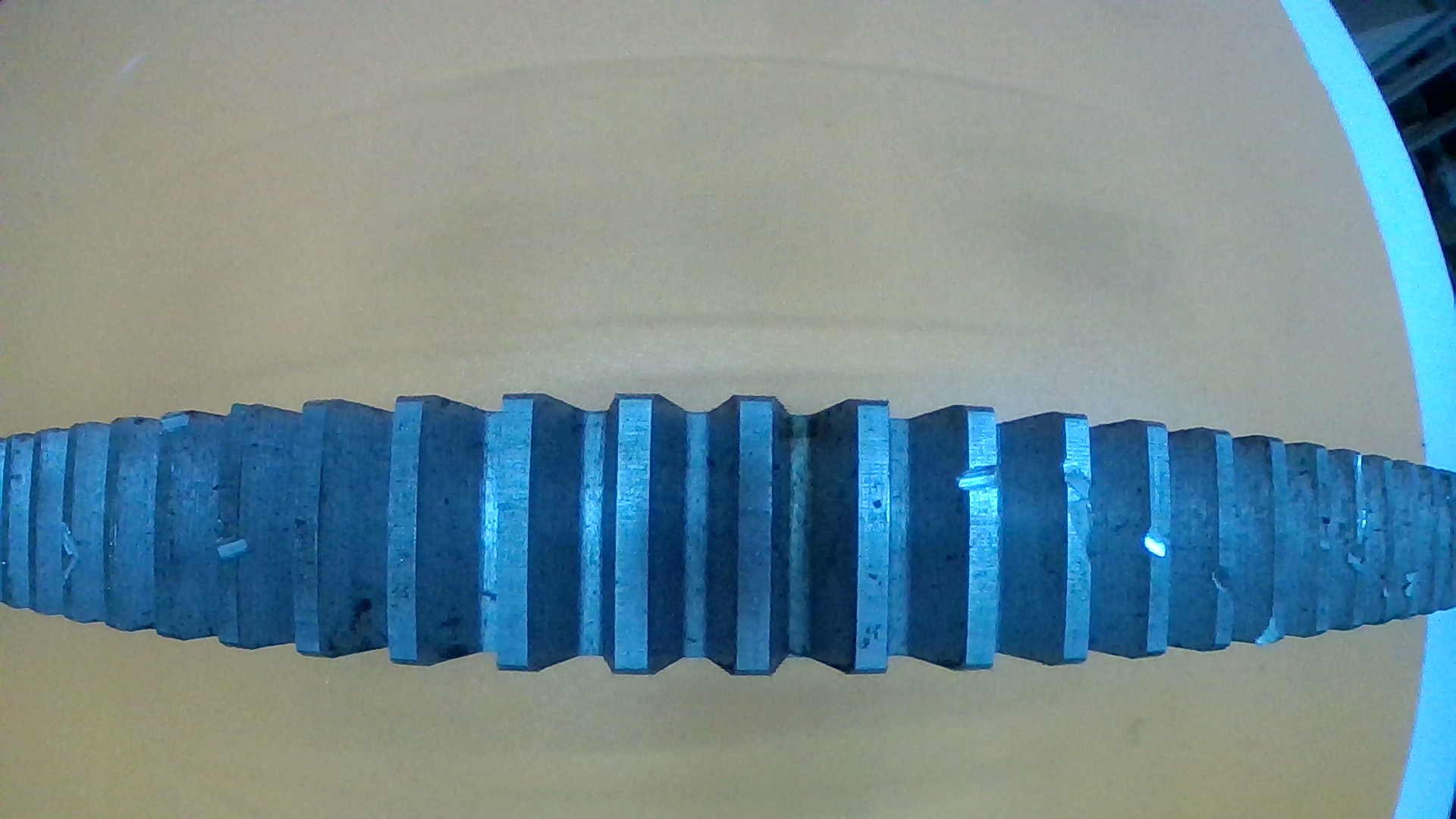

Supplement: S1 Data — (ZIP) [file pone.0322217.s001.zip › dataset/3/WIN_20250111_19_16_30_Pro.jpg]

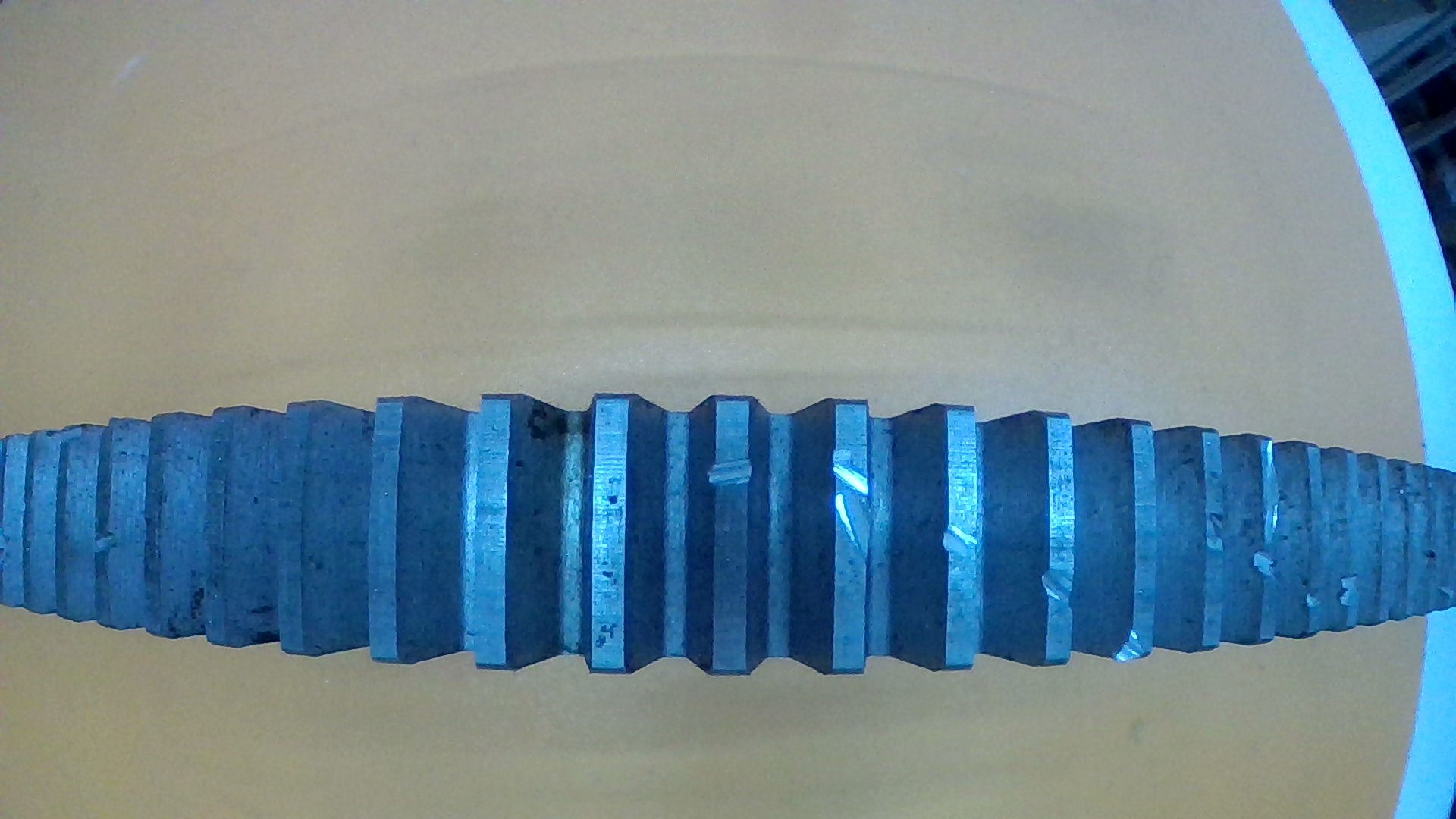

Supplement: S1 Data — (ZIP) [file pone.0322217.s001.zip › dataset/3/WIN_20250111_19_16_33_Pro.jpg]

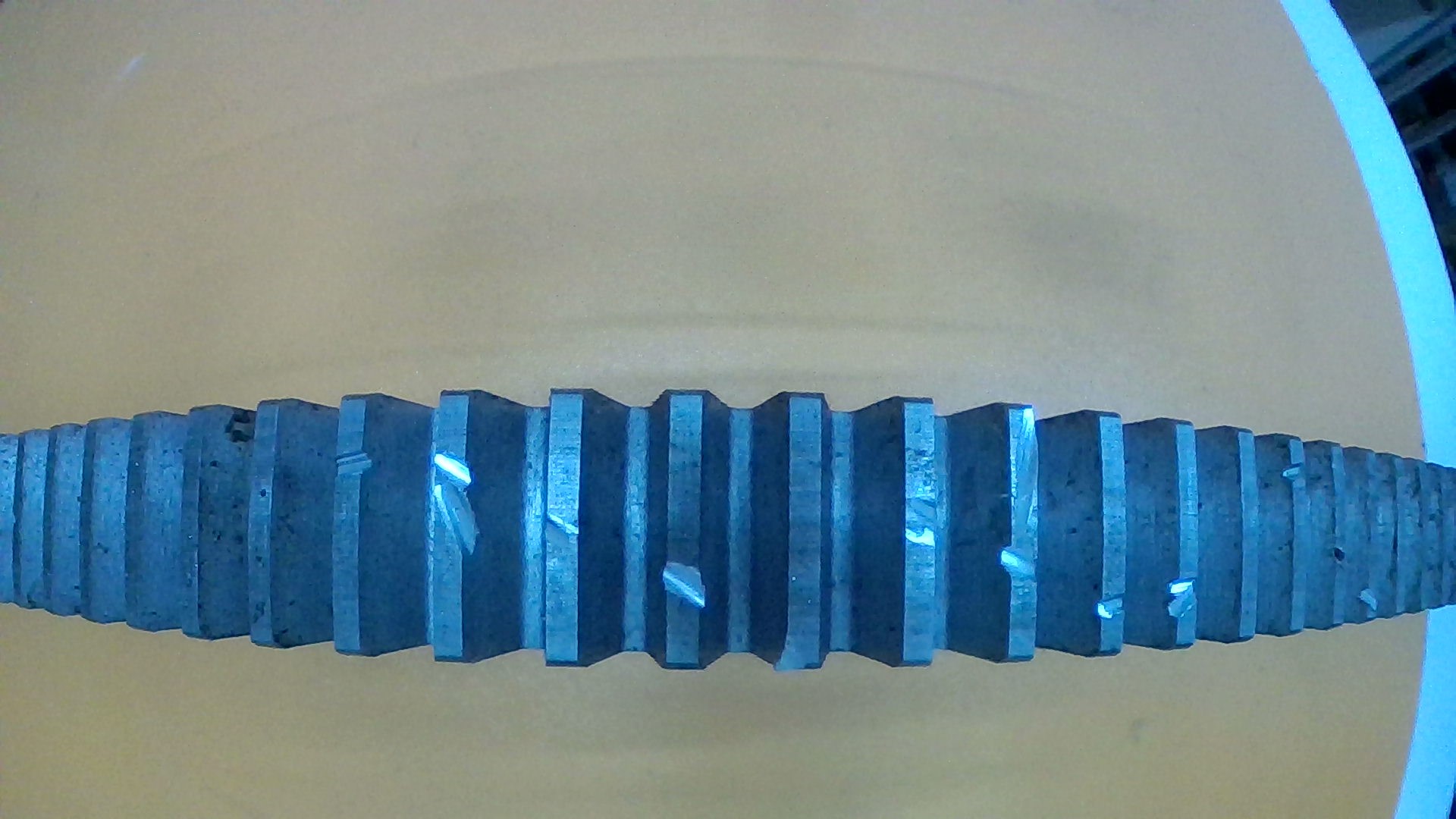

Supplement: S1 Data — (ZIP) [file pone.0322217.s001.zip › dataset/3/WIN_20250111_19_16_37_Pro.jpg]

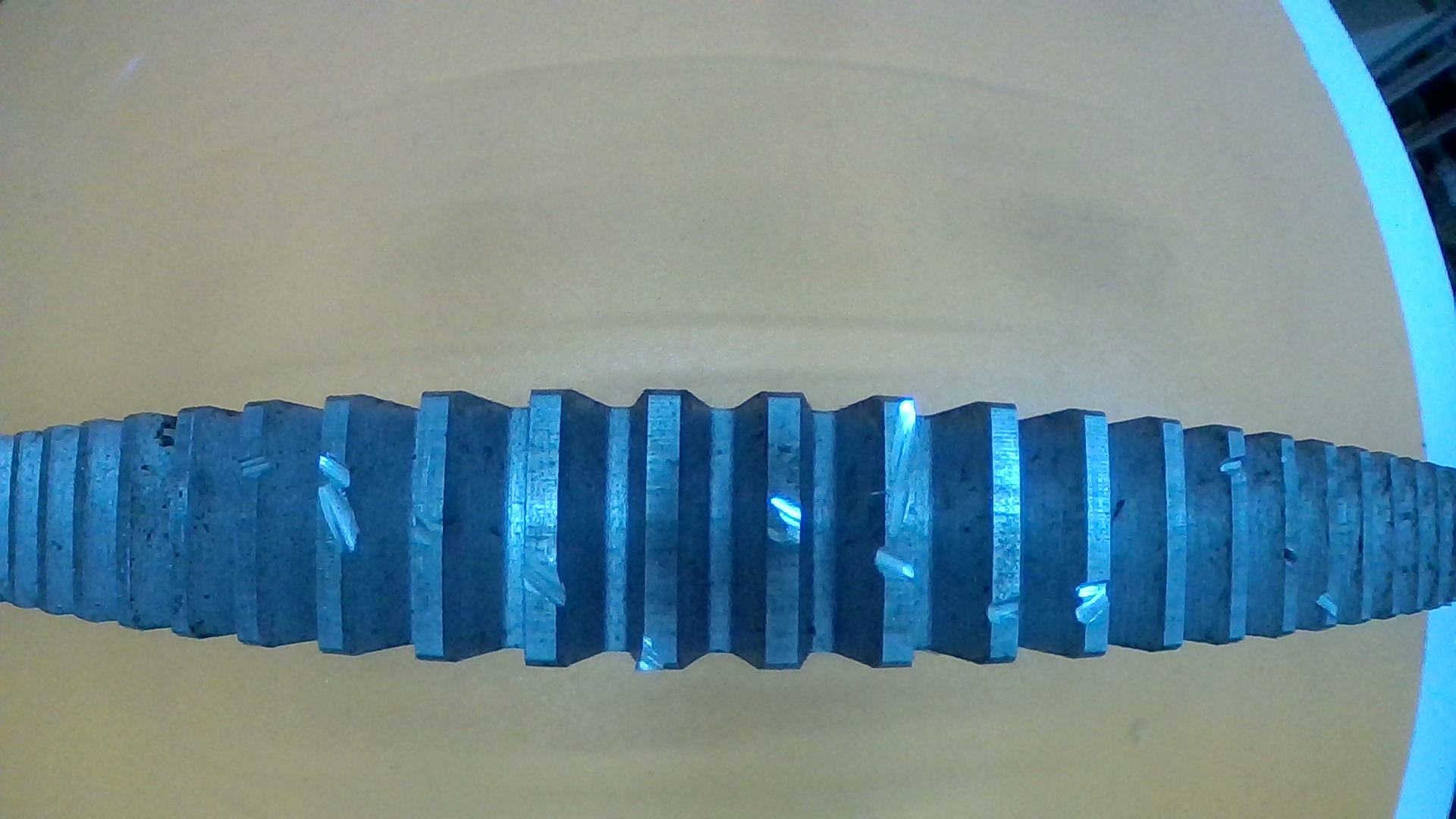

Supplement: S1 Data — (ZIP) [file pone.0322217.s001.zip › dataset/3/WIN_20250111_19_16_40_Pro.jpg]
