# Supplementary material for: An image processing technique for optimizing industrial defect detection using dehazing algorithms
Source: PLoS One. 2025 May 2;20(5):e0322217. doi: 10.1371/journal.pone.0322217 (PMC12047806; doi:10.1371/journal.pone.0322217)
Supplement: Attachment — Submitted filename: Response to Reviewers.docx [file pone.0322217.s003.docx]

Reviewer #1: The study addresses a significant problem in industrial defect detection, particularly the impact of environmental
factors like water fog on image quality. The proposed solution is highly practical and applicable to real-world industrial settings, making it valuable for automated quality control systems. The integration of dehazing, denoising, and illumination enhancement is a strong aspect of the research, as it provides a holistic solution rather than focusing solely on one aspect of image enhancement. The use of an improved YOLOv8 model further strengthens defect detection accuracy.
The paper compares CNN, MADNet, and the improved YOLOv8 model, it lacks a strong justification for why these specific models were chosen. There are many other state-of-the-art dehazing and object detection models (e.g. Transformer-based architectures) that could have been considered.
The authors should conduct statistical tests (e.g., t-tests or ANOVA) to determine whether the improvements in PSNR, SSIM, and detection confidence are statistically significant compared to baseline models (CNN, MADNet, etc.). Further a subset of data should be available publicly for validation.

Response：Thanks to the comments of the reviewers, this paper does lack a justification for the choice of a specific model. This modification adds a detailed justification, and adds a statistical test to determine whether the improvement in PSNR, SSIM and detection confidence is statistically significant compared with the baseline model. I understand the importance of datasets, which are only partially available for copyright and other privacy reasons. Thank you for your hard work on this article.

Reviewer #2: The paper is good as it addresses the underlined objectives it seek to achieve. However, there are just a few things that needs to be done regarding the consistency of the word "Angle iron" in Figure 6, line 393, figure 7 line 427, and then table 1, line 504 and table 2, line 538 compared to the other words "Rngle iron". This should be cross checked and corrected.

Response：I would like to thank the reviewers for their careful inspection. There is indeed a conflict problem during the process, and I have made the modification. Thank you again for your hard work on this paper.
